# Risk Factors Associated With Sarcopenia in Patients With Chronic Kidney Disease: A Systematic Review and Meta‐Analysis

**DOI:** 10.1002/jcsm.70166

**Published:** 2025-12-28

**Authors:** Kaili Jin, Xiaoyan Li, Yiqin Ma, Dan Yang, Xiaoxue Tan, Qiuhua Sun, Rongyun Wang

**Affiliations:** ^1^ School of Nursing Zhejiang Chinese Medical University Hangzhou China; ^2^ Department of Orthopeadics and Traumatology The First Affiliated Hospital of Zhejiang Chinese Medical University Hangzhou China

**Keywords:** chronic kidney disease, meta‐analysis, risk factors, sarcopenia

## Abstract

**Background:**

Sarcopenia is an age‐related degenerative disorder characterized by a progressive decline in skeletal muscle mass, strength, and function with high prevalence in chronic kidney disease (CKD). Identifying clinical and epidemiological factors of sarcopenia in patients with CKD is essential to enable early recognition and appropriate clinical interventions.

**Methods:**

We conducted a systematic search for resources from PubMed, Embase, Web of Science, Wangfang, VIP (China Science and Technology Journal Database), CNKI (National Knowledge Infrastructure), CMAJD (Chinese Medical Association Journal Database), and SinoMed databases until 21 May 2025. We included studies that reported risk factors for sarcopenia in patients with CKD. All data were extracted independently by two reviewers using a standardized data collection form. The odds ratio (OR) for each risk factor was combined from the included studies. Sensitivity analyses and additional subgroup analyses were conducted.

**Results:**

Finally 58 studies involving a total of 15 425 participants were included. Risk factors with a significant association with sarcopenia in patients with CKD included diabetes (OR = 1.96; 95% CI: 1.51–2.54; *p* < 0.001). In contrast, higher BMI (per 1 kg/m^2^) (OR = 0.76; 95% CI: 0.65–0.88; *p* < 0.001) was associated with a lower risk. In addition, for non‐dialysis‐dependent CKD (NDD‐CKD) patients, older age (per 1 year), diabetes, and higher C‐reactive protein (per 1 mg/L) were associated with an increased risk of sarcopenia. In contrast, higher BMI (per 1 kg/m^2^), higher carbon dioxide binding capacity (per 1 mmol/L), and an increase in body protein content (per 1 kg) were protective in this group. In haemodialysis (HD) patients, diabetes and higher body protein content (per 1 kg) were associated with an increased risk of sarcopenia. While higher BMI (per 1 kg/m^2^), higher carbon dioxide binding capacity (per 1 mmol/L), and regular exercise were protective in this group. In renal transplant recipients (RTR), longer dialysis vintage (per 1 month) was identified as a protective factor.

**Conclusion:**

This study comprehensively illustrated that the development of sarcopenia in patients with CKD is influenced by a variety of risk factors across various domains. The identification of patients at a high risk of sarcopenia who could benefit from enhanced prophylaxis and treatment can be facilitated by the knowledge of risk factors that have a strong association with sarcopenia in patients with CKD. It is imperative to prioritize the identification of modifiable risk factors in order to enhance the effectiveness of prevention and treatment.

## 1 Introduction

Sarcopenia is an age‐related degenerative condition marked by a reduction in skeletal muscle mass, strength, and/or function, and is linked to heightened risks of unfavourable outcomes including falls, functional decline, frailty, and mortality [1, 2]. Chronic kidney disease (CKD) represents a significant public health problem [3]. Recent studies have found that CKD promotes sarcopenia, and symptoms of sarcopenia are more likely to be observed in CKD patients than in their peers [4, 5]. The prevalence of CKD combined with sarcopenia ranged from 4% to 42% [6, 7]. In addition to the symptoms of kidney disease, patients with CKD combined with sarcopenia also experience weight loss, slowed walking speed, and decreased mobility. These factors significantly impact the quality of life of patients and increase the risk of falls, fractures, infections, loss of independence, and mortality [8, 9, 10]. Therefore, emphasizing CKD combined with sarcopenia and intervening as early as possible has positive significance in improving the prognosis of CKD patients.

According to the revised criteria of the European Working Group on Sarcopenia in Older People (EWGSOP2) [2], sarcopenia is defined not only by a reduction in muscle mass but also by decreased muscle strength, with low muscle strength considered the primary determinant of probable sarcopenia. In patients with CKD, the progressions of muscle mass and muscle strength during disease progression are not strongly associated. Although muscle mass may remain stable, muscle strength tends to decline significantly with increasing CKD severity [11]. The etiologies of muscle disorders resulting in skeletal muscle loss in CKD are varied, including renal disease, dialysis, and the low‐grade chronic inflammation characteristic of CKD patients. These variables collectively enhance protein breakdown, diminish protein synthesis, and result in a negative protein balance [12, 13, 14, 15, 16, 17]. Numerous studies have suggested that various factors, such as age, gender, BMI, and osteocalcin, may be associated with sarcopenia in individuals with CKD [18, 19, 20, 21]. However, the findings of different studies remain inconsistent.

This study aimed to systematically review and synthesize published evidence on clinical and epidemiological factors associated with sarcopenia in patients with CKD. Among the numerous risk factors identified, some are modifiable, meaning they can be altered or controlled to reduce the risk of sarcopenia. In addition, this study sought to identify potential new risk factors. It may also aid in identifying individuals with CKD who are at high risk for sarcopenia and may require closer monitoring as well as enhanced prevention and treatment strategies.

## 2 Materials and Methods

Our review protocol is registered in PROSPERO (CRD42024551344). This study was exempt from ethical review due to its reliance on previously published data; consequently, informed consent was waived. The study adhered to the Preferred Reporting Items for Systematic Reviews and Meta‐Analyses (PRISMA) reporting guideline [22] and the Meta‐analysis Of Observational Studies in Epidemiology (MOOSE) checklist [23].

### 2.1 Databases and Search Strategy

Four reviewers (K.J., X.L., Y.M. and D.Y.) independently searched a computer database for literature up to 21 May 2025. We conducted a search of PubMed, Web of Science, Embase, CNKI, Wanfang Data, SinoMed, VIP, the Chinese Medical Association Journal Database, and publicly available literature using EndNote X9. We also reviewed citations in the retrieved articles to identify additional relevant studies on sarcopenia‐associated factors in individuals with CKD. These English search phrases derive from combinations of associated MeSH terms, such as Chronic Kidney Diseases/Chronic Renal Insufficiencies/Chronic Renal Insufficiency/Chronic Kidney Insufficiency, Sarcopenias/Muscular Atrophy/muscle mass/muscle strength, Risk Factors/Social Risk Factor. To maximize our literature search, we incorporated methodological traceability into the review process. Detailed results were presented in Data S1.

### 2.2 Inclusion and Exclusion Criteria

The inclusion criteria are as follows: (1) including cross‐sectional studies, prospective and retrospective cohort studies, randomized controlled studies, and case–control studies. (2) Study subjects: Age > 18 years, with a clinical diagnosis of CKD, including non‐dialysis‐dependent CKD (NDD‐CKD), haemodialysis (HD), peritoneal dialysis (PD) and renal transplant recipients (RTR) patients. The diagnosis of kidney disease is based on the Improving Global Outcomes (KDIGO) diagnostic criteria [24]. Sarcopenia must be assessed according to the diagnostic guidelines for sarcopenia, including guidelines from the European Working Group on Sarcopenia in Older People (EWGSOP), Asian Working Group for Sarcopenia (AWGS), Foundation for the National Institutes of Health (FNIH), and Chinese Society of Osteoporosis and Bone Mineral Research Guidelines (CSOBMR). (3) The study reported at least one risk factor for sarcopenia in patients with CKD.

The exclusion criteria are as follows: (1) Studies not published in Chinese or English. (2) Duplicate publications. (3) Studies for which outcome data could not be extracted. (4) Animal experiments.

### 2.3 Literature Selection and Data Extraction

Three researchers (K.J., X.L. and X.T.) independently screened the titles and abstracts according to the inclusion and exclusion criteria. The full text of the studies was then rescreened to determine the final included studies. Disagreements were resolved through discussion or consultation with two senior reviewers (R.W. and Q.S.). If necessary, contact the author of the study for data confirmation. Data were extracted using a standardized form template that included the first author, publication year, study country, geographical classification, study design, sample size, prevalence by sex, diagnostic criteria and measurement methods for sarcopenia, CKD treatment modality, prevalence of sarcopenia and associated risk factors, CKD subtype and stage, and comorbidities.

### 2.4 Quality Assessment

The quality of the included articles was independently assessed by two reviewers (X.T. and Q.S.). Any disagreements were resolved by consensus or, if necessary, by consulting a third reviewer (R.W.). Joanna Briggs Institute (JBI) Critical Appraisal tool [25] was used to assess the quality of the cross‐sectional studies. It consists of 8 items, each of which can be assessed as yes, no, unclear, and not applicable. All entries are YES with a research quality rating of A, and some entries are YES with a research quality rating of B (1–3 entries are ‘no’) [26]. The Newcastle‐Ottawa Scale (NOS) Quality Assessment Tool [27] was used to evaluate the quality of cohort and case–control studies. These two measures assess three aspects: selection, comparability, and exposure/outcome. The maximum score is 9, with scores ranging from 0 to 4 classified as low‐quality studies, and scores from 5 to 9 classified as high‐quality studies [26, 28]. The Cochrane Handbook for Systematic Reviews of Interventions [29] was used to assess the quality of the literature on randomized controlled trials, with three levels: low risk of bias, unclear risk of bias and high risk of bias.

### 2.5 Statistical Analysis

We used Stata 17.0 for statistical analyses, with odds ratios (ORs) and 95% confidence intervals (CIs) as the primary measures. We used R software version 4.4.2 to establish the forests. Adjusted ORs were preferentially extracted and used as the main effect estimates. Crude ORs were presented when the pertinent adjusted ORs are unavailable. For risk factors with a significant crude OR (*p* < 0.05) but a non‐significant adjusted OR, we provide both the crude and adjusted OR in the text. For studies that did not report odds ratios (ORs) and their confidence intervals but provided mean ± standard deviation data, we converted standardized mean differences (SMDs) into log odds ratios (log ORs) as recommended by the Cochrane Handbook [30] (Version 6.5, Chapter 9.4.6 ‘Combining dichotomous and continuous outcomes’). The standard errors of these log ORs were adjusted using the constant $\sqrt{3}$/π ≈ 0.5513 to enable consistent estimation and facilitate pooling of continuous and dichotomous data within the same meta‐analysis. The *I* ^2^ statistic was employed to evaluate statistical heterogeneity. If *I* ^2^ < 50% signifies minimal heterogeneity among studies, a fixed‐effect model was employed, conversely, if *I* ^2^ > 50% indicates substantial heterogeneity, a random‐effects model was utilized. Employ subgroup analysis to explore the sources of heterogeneity. Subgroup analyses were conducted according to CKD treatment modality, categorized into NDD‐CKD patients, PD, HD, and RTR patients, to explore potential differences in the associations between risk factors and sarcopenia across different stages of disease management. Sensitivity analysis was conducted using the Leave‐One‐Out (LOO) method, in which each study was sequentially excluded to assess the robustness and stability of the pooled results. Publication bias was evaluated using funnel plots and Egger's test. Publication bias was also assessed using funnel plots and the Egger's test. For analyses showing potential publication bias, we applied the trim‐and‐fill method to estimate the adjusted effect size by imputing hypothetical missing studies to balance the asymmetry of the funnel plot. For each risk factor, we extracted effect estimates as reported in the included studies. Continuous variables, such as age and BMI, were treated as continuous measures (e.g., per 1‐year increase, per 1 kg/m^2^ increase). Categorical variables were analysed as dichotomous exposures according to the definitions provided in each study (e.g., female vs. male for sex, presence vs. absence of diabetes). Where studies reported the same variable in both continuous and categorical forms, we conducted separate meta‐analyses to avoid methodological heterogeneity.

## 3 Results

**FIGURE 1:**
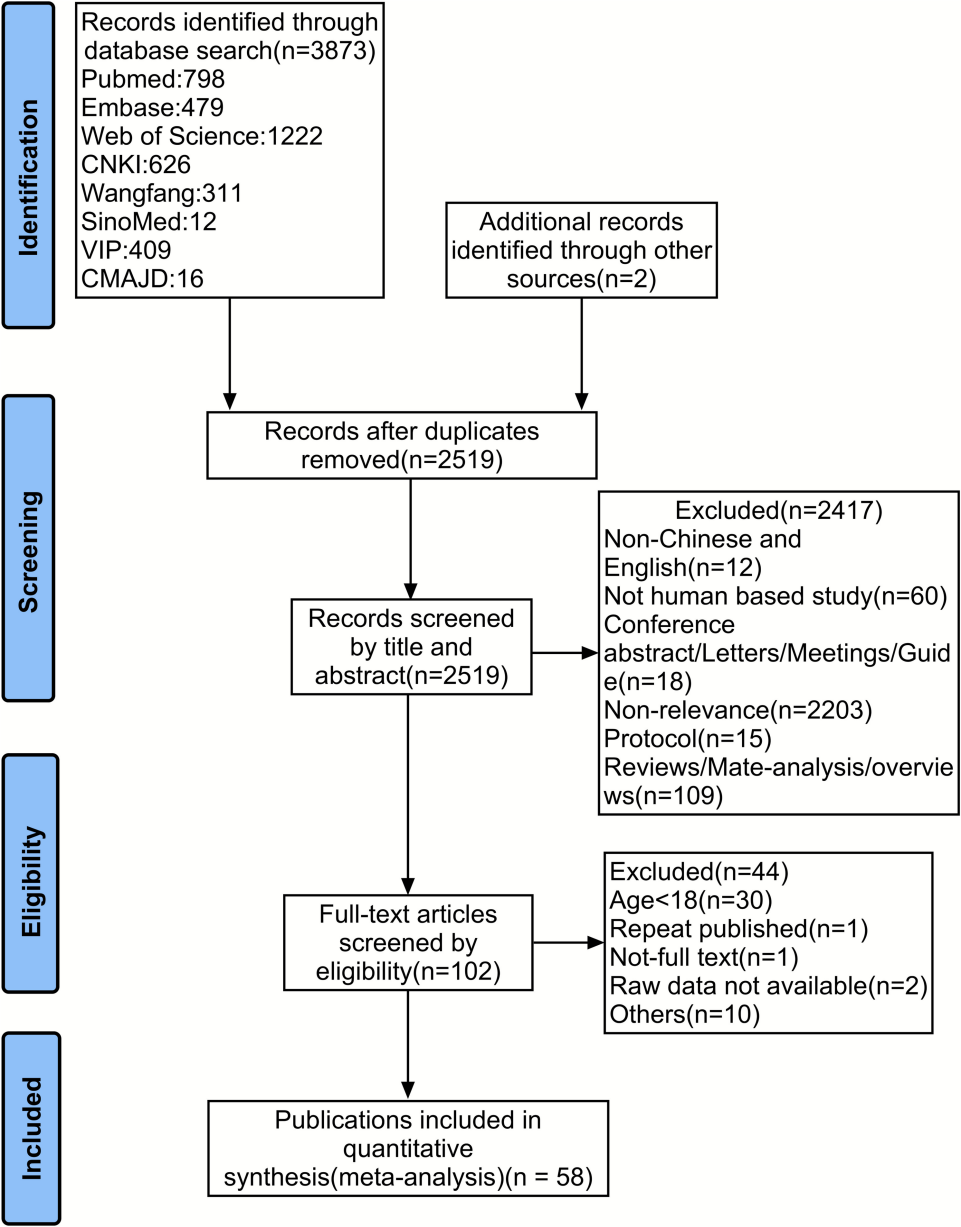
Flowchart of the meta‐analysis.

Initially, 3875 articles were screened for this study. After the removal of duplicate records, 2519 articles remained. Following the preliminary review of titles and abstracts, 102 articles were selected. After reading the full text, 58 studies [5, 19, 20, 21, 31, 32, 33, 34, 35, 36, 37, 38, 39, 40, 41, 42, 43, 44, 45, 46, 47, 48, 49, 50, 51, 52, 53, 54, 55, 56, 57, 58, 59, 60, 61, 62, 63, 64, 65, 66, 67, 68, 69, 70, 71, 72, 73, 74, 75, 76, 77, 78, 79, 80, 81, 82, 83, 84] were included in this review finally (Figure 1). The characteristics of the included studies were shown in Table 1.

**TABLE 1:** Characteristics of included studies.

Study	Study design	Country	The crowd source	Sample (*N*) (total/male/female)	Prevalence (total‐male–female), %	Age, year	Definition of sarcopenia	Assessment method of muscle mass	CKD treatment modality	Influencing factor
Han [31] 2011	Cross‐sectional study	China	Outpatients	101/51/50	NR	35–85	EWGSOP (2019)	BIA	HD	Age, female gender, serum myostatin level, dialysis status and the type of dialyzer; (the grip strength was negatively related to age, female gender, muscle mass, myostatin levels and haemodialysis, but positively to the use of high‐flux dialyzer in linear regression.
Leal [32] 2011	Cross‐sectional study	Brazil	Outpatients	43/25/18	55.8–27.9‐27.9	54.5 ± 12.2	NR	DXA	HD	HD patient
Kojo [33] 2014	Cross‐sectional study	Japan	Outpatients	60/60/NR	NR	41–89	NR	CT	HD	Serum total testosterone and age in male
Ozkayar [34] 2014	Cross‐sectional study	Turkey	Outpatients	166/68/98	20.5–13.3‐7.2	37.9 ± 11.9	Cardiovascular Health Study (CHS)	BIA	RTR	Age
Souza [35] 2017	Cross‐sectional study	Brazil	Outpatients	100/NR/NR	EWGSOP:11.9–31‐27;FNIH:28.7‐NR‐NR	73.59 ± 9.22	EWGSOP + FNIH	DXA	NDD‐CKD	Walking speed, BMI
Tufan [36] 2017	Cross‐sectional study	Turkey	Community patients	209/NR/NR	29.7–29.7‐NR	67.8 ± 6.4	Cardiovascular Health Study (CHS)	Other	NDD‐CKD	Higher age
Yanishi [37] 2017	Cross‐sectional study	Japan	Outpatients	51/36/15	11.8–7.8‐3.9	46.2 ± 12.8	AWGS	DXA + BIA	RTR	Age, duration of dialysis before transplantation
As'habi [38] 2018	Cross‐sectional study	Iran	Outpatients	79/35/44	11.5‐NR‐NR	52.1 ± 17.22	Other	BIA	PD	Genderthe prevalence of dynapenia and the age of patients, physical activitylevel, and the presence of diabetes mellitus
D'Alessandro [39] 2018	Cross‐sectional study	Italy	Outpatients	80/NR/NR	33.75‐NR‐NR	73.7 ± 7.2	EWGSOP	BIA	NDD‐CKD	Age, physical capacity
Ishikawa [40] 2018	Cross‐sectional study	Japan	Outpatients	260/169/91	25.0–18.5‐6.5	69–80	AWGS	DXA	NDD‐CKD	Age, male gender, body mass index, diabetes mellitus, loop diuretic use
Yoowannakul [41] 2018	Cross‐sectional study	NR	Outpatients	600/373/227	The prevalence of sarcopenia was FNIH 68.3% for Asian, 27.1% for Black and 36.6% for White women byand 59.6% Asian, 21.3% Black and 39.9% White men by EWGS criteria	51.9–81.4	FNIH + EWGSOP + AWGS	BIA	HD	Gender, ethnicity
Guida [42] 2019	Cross‐sectional study	Italy	Outpatients	88/59/29	44.3‐NR‐NR	53.4 ± 13.1	FMI + SM/BW cutoff values	BIA + BIVA	PD	Obesity, diabetes
Quiñónez Olivas [43] 2019	Cross‐sectional study	Mexico	Outpatients	84/32/52	51–20.5‐30.7	76 ± 7.5	EWGSOP	BIA	NDD‐CKD	Age, FM, LBI, BMI
Shen [44] 2019	Cross‐sectional study	China	Outpatients	207/122/85	13.0–9.7‐3.4	55.3 ± 13.7	AWGS	BIA	PD	Male, longer PD duration, higher ECW/ICW
Hortegal [45] 2020	Cross‐sectional study	Brazil	Outpatients	209/124/85	29.2–41.9‐10.6	51.9 ± 15.0	EWGSOP2	DXA	HD	Being inflamed, presence of DM, being male, increasing age, body fat, BMI
Kang [46] 2020	Cross‐sectional study	South Korea	Outpatients	84/44/40	NR	≥20	AWGS	DXA	HD	Serum vitamin D level
Saito [47] 2020	Cross‐sectional study	Japan	Outpatients	231/159/72	/	75.9 ± 6.1	NR	Other	NDD‐CKD	Serum active vitamin D level
Song [48] 2022	Case controlled study	China	Outpatients	72/33/39	/	56.80 ± 10.86	NR	BIA	NDD‐CKD	Age, serum 25(OH)D, dialysis vintage, NT‐proBNP
Visser [49] 2020	Cohort study	Netherlands	Outpatients	54/38/16	/	52–74	EWGSOP2	BIA	HD	Male sex, inflammation
Zhang Qi [51] 2020	Cohort study	China	Outpatients	135/89/46	62.9‐NR‐NR	70.6 ± 7.7	AWGS	BIA	HD	Advanced age, low BMI
Zhang Qian [50] 2020	Cross‐sectional study	China	Outpatients	174/93/81	/	63.05 ± 12.29	NR	BIA	HD	Gender, daily steps, muscle mass, 25(OH)D level and IL‐6 in young group, and muscle mass, 25(OH)D, daily steps, and NT‐proBNP in elderly group
Zhu [52] 2020	Cross‐sectional study	China	Outpatients	113/56/57	23–19.6‐26.3	56.1 ± 13.8	AWGS	BIA	PD	Decreased BMI, increased total body water, decreased protein content
Abdala [53] 2021	Cross‐sectional study	Argentina	Outpatients	100/60/40	16–11.1‐25	55.6	EWGSOP2	DXA	HD	Gender;
An [54] 2021	Cohort study	Korea	Outpatients	892/523/369	28.1–16.4‐11.8	56–77	AWGS	BIA	NDD‐CKD	Age, BMI, diabetes, hypertension, blood pressure, eGFR, haemoglobin, serum levels of total cholesterol, protein, albumin, total CO_2_, total calcium
Du [55] 2021	Cross‐sectional study	China	Outpatients	125/68/57	31.2–18.4‐12.8	59.4 ± 14.9	EWGSOP2	DXA	HD	Low CO_2_CP, high vWF, no regular exercise
Hsu [56] 2021	Cross‐sectional study	China	Outpatients	118/61/57	NR	63.2 ± 13.2	Other	BIA	HD	Higher serum leptin levels in male
Dubey [57] 2021	RCT	India	Outpatients	188/134/54	69.1‐NR‐NR	48.5–51.9	AWGS	DXA	NDD‐CKD	Age, low BMI, eGFR, lower bicarbonate levels
Mattera [58] 2021	Cross‐sectional study	Italy	Outpatients	77/49/28	53.1–40.2‐13.0	62.7 ± 13.8	EWGSOP	DXA	HD	Low serum albumin, low serum phosphorus level, female sex, low BMI
Umakanthan [5] 2021	Cross‐sectional study	Australia	Outpatients	39/28/11	18–7.7‐10.3	54–77	EWGSOP	BIS	HD	Female sex, low serum albumin, low serum phosphorus level, age, female sex
Yu [59] 2021	Cohort study	China	Inpatients	180/87/93	NR‐55.7‐41.9	59.43 ± 13.70	AWGS	DXA	NDD‐CKD	Age, CKD progression
Amorim [60] 2022	Cross‐sectional study	Brazil	Outpatients	139/NR/NR	20.9–27.0‐13.8	57 ± 13.5	EWGSOP2	BIA	NDD‐CKD	Lower PhA values, higher IL‐6 levels, lower serum creatinine levels
Nam [61] 2022	Cohort study	Korea	Outpatients	517/342/175	25.5‐NR‐NR	56–77	AWGS(2019)	BIA	NDD‐CKD	The serum cystatin C/Cr ratio
Song [62] 2020	Cross‐sectional study	China	Outpatients	105/105/NR	31.4–31.4‐NR	68–77	Consensus on Sarcopenia by the Chinese Society of Osteoporosis and Bone Mineral Research (CSOBMR Consensus on Sarcopenia)	DXA	HD	Deterioration of renal function: eGFR based on serum creatinine, Cys‐C (eGFRscr‐cys) lower than 45 mL·min^−^1·(1.73 m^2^)^−1^, eGFR based on Cys‐C (eGFRcys) lower than 45 ml·min^−1^·(1.73 m^2^)^−1^
Xavier [63] 2022	Cross‐sectional study	Brazil	Outpatients	218/124/94	60.6–31.7‐28.9	58.3 ± 14.6	EWGSOP	Other	HD	Worse nutritional status
Yildirim [64] 2022	Case controlled study	Turkey	Inpatients	240/120/120	8.3% of patients in the CKD group, 3.3% in the renal transplantation group	40.42 ± 10.60	FNIH	BIA	RTR	IGF‐1 levels in renal transplant recipients
Li [65] 2023	Cross‐sectional study	China	Outpatients	182/107/75	33.5–18.7‐14.8	50.2 ± 5.3	AWGS (2019)	DXA	HD	Blood low‐density lipoprotein cholesterol ≥ 3.37 mmol/L, ejection fraction < 50%, chest CT‐PTB and suspected PTB
Zhang [66] 2023	Case controlled study	China	Outpatients	116/NR/NR	43.97–34.5‐21.6	>60	Other	BIA	NDD‐CKD	Age, poor sleep quality, poor nutritional status and negative emotions (age, as well as PSQI, MIS, SAS, and SDS scores were the risk factors for sarcopenias in CKD, while BMI, bone mass, MAC, Scr, UA and TG were protective factors
Nie [67] 2023	RCT	China	Outpatients	196/103/93	The prevalence of sarcopenia in the control group, peritoneal dialysis group and chronic kidney disease group was 3.4%(2/59), 23.0%(14/61) and 11.8%(9/76)	50.7 ± 14.1	AWGS	DXA + BIA	PD	Level of high‐sensitivity C‐reactive protein, dialysis duration (months) are independent risk factors for the development of sarcopenia in peritoneal dialysis patients;Age;low BMI are independent risk factors for the development of sarcopenia in patients with chronic kidney disease
Moreno‐González [68] 2023	Cross‐sectional study	Austria	Outpatients	1420/NR/NR	NR	79.0 ± 6.0	EWGSOP	BIA	NDD‐CKD	Age, body mass index (BMI), disability performing instrumental activities of daily living (IADL), Mini Mental State Examination (MMSE) score < 24, osteoporosis, stage 4 CKD defined by CKD‐EPIBTP‐B2M, a non‐creatinine‐based eGFR equation,
Wang [69] 2023	Case controlled study	China	Outpatients	162/89/73	24.7–15.4‐9.4	69–82	AWGS (2019)	BIA	NDD‐CKD	Age, BMI, regular exercise, dementia, haemoglobin, carbon dioxide binding capacity, eGFR, urea nitrogen, serum albumin, CRP, and administration of alpha keto acids
Hori [73] 2024	Case controlled study	Japan	Outpatients	95/74/21	23.1–18.94‐4.2	66.9 ± 11.5	AWGS (2019)	BIA	HD	Lower serum 25(OH)D levels
Ozcan [79] 2024	Cross‐sectional study	Turkey	Outpatients	93/55/38	15.0‐NR‐NR	59 ± 1.4	EWGSOP (2019)	BIA	RTR	Renal transplanth (cadaveric transplantation), Diabetes mellitus, lower albumin levels
Chen [20] 2024	Cohort study	China	Outpatients	250/142/108	48–27.2‐20.8	57.38 ± 6.46	CSOBMR	BIA	HD	Age, grip strength, body protein content, BMl, MOS.GA score, CRP level
Alirezaei [70] 2024	Cohort study	Germany	Outpatients	137/91/46	40.14–23.64‐76.36	60.77 ± 15.28	AWGS (2019)	BIA	HD	None
Álvarez [71] 2024	Cross‐sectional study	Chile	Outpatients	23/11/12	56.5–21.7‐34.8	69.1 ± 5.8	EWGSOP2	BIA	HD	Fasting glycemia, glycosylated haemoglobin, and malnutrition inflammation
Ben‐Noach [72] 2024	Cohort study	Israel	Outpatients	74/50/24	44.5–14.5‐30	69.2 ± 14.3	EWGSOP2	Other	HD	Diabetes mellitus, female sex
Hsu [74] 2024	Cross‐sectional study	China	Outpatients	420/258/162	24.5–16.9‐7.61	69.0 ± 11.8	AWGS (2019)	Other	NDD‐CKD	The average ankle–brachial index (ABI), vascular reactivity index (VRI)
Hu [75] 2024	Cross‐sectional study	China	Outpatients	386/252/134	28.5–26.42‐13.47	58.9 ± 14.3	NR	BIA	HD	Blood manganese level
Huang [76] 2024	Cross‐sectional study	China	Outpatients	3648/1701/1947	3.89–2.78‐1.20	71.9 ± 6.07	AWGS (2019)	BIA	NDD‐CKD	Male sex and the aging process
Miyasato [77] 2024	Cross‐sectional study	Japan	Outpatients	201/123/78	NR	69.8 ± 13.2	AWGS (2019)	BIA	HD	Oral Frailty
Nishihira [78] 2024	Cohort study	Japan	Outpatients	371/243/128	NR	52	AWGS (2019)	DXA + BIA	RTR	Bone mineral density
Qaisar [80] 2024	Cross‐sectional study	United Arab Emirates	Outpatients	277/277/0	NR	68.2 ± 4.2	EWGSOP2	DXA	NR	Intestinal leak
Wu [81] 2024	Cross‐sectional study	China	Outpatients	111/75/36	59.8–45.94‐20.72	62.10 ± 8.15	AWGS (2019)	BIA	HD	Age, gender, body mass index (BMI), dialysis time, economic status, marital status and pre‐dialysis creatinine
Zeng [21] 2024	Cross‐sectional study	China	Outpatients	244/122/98	9.8‐NR‐NR	53.1 ± 13.8	AWGS (2019)	BIA	HD	PhA
Zhao [19] 2024	Cohort study	China	Outpatients	165/95/70	21.82–13.93‐7.88	64.6 ± 9.5	AWGS (2019)	BIA	HD	Age, waist circumference, handgrip strength, and InBody score
Chang [82] 2025	Cohort study	China	Outpatients	196/131/65	14.8–6.12‐8.8	53.57 ± 12.43	AWGS (2019)	BIA	HD	Non leisure‐time physical activity
M [83] 2025	Cross‐sectional study	India	Outpatients	411/209/202	51–25.3‐25.54	62.72 ± 10.82	AWGS (2019)	BIA	HD	Neutrophil‐to‐lymphocyte ratio (NLR)
Mansouri [84] 2025	Cross‐sectional study	Iran	Outpatients	109/59/50	14.7‐NR‐NR	64.27	AWGS	BIA	NR	The Planetary Health Diet Index (PHDI)

Abbreviations: 25(OH)D, 25‐hydroxyvitamin D; ABI, ankle–brachial index; AWGS, Asian Working Group for Sarcopenia; B2M, beta‐2 microglobulin; BIA, bioelectrical impedance analysis; BIS, bioimpedance spectroscopy; BIVA, bioelectrical impedance vector analysis; BMI, body mass index; BTP, beta‐trace protein; CHS, Cardiovascular Health Study; CKD‐EPI, Chronic Kidney Disease Epidemiology Collaboration; CO_2_CP, carbon dioxide combining power; CRP, C‐reactive protein; CSOBMR, Chinese Society of Osteoporosis and Bone Mineral Research (guidelines); CT, computed tomography; Cys‐C, cystatin C; DM, diabetes mellitus; DXA, dual‐energy X‐ray absorptiometry; ECW/ICW, extracellular water to intracellular water ratio; eGFR, estimated glomerular filtration rate; EWGSOP, European Working Group on Sarcopenia in Older People; EWGSOP2, European Working Group on Sarcopenia in Older People (2019 consensus update); FM, fat mass; FMI, fat mass index; FNIH, Foundation for the National Institutes of Health; HD, haemodialysis; IADL, Instrumental Activities of Daily Living; IGF‐1, insulin‐like growth factor 1; IL‐6, interleukin‐6; LBI, Lawton–Brody Index; MAC, mid‐arm circumference; MIS, Malnutrition‐Inflammation Score; MMSE, Mini Mental State Examination; MOS.GA, malnutrition and overall geriatric assessment composite score; NDD‐CKD, non–dialysis‐dependent chronic kidney disease; NLR, neutrophil‐to‐lymphocyte ratio; NT‐proBNP, N‐terminal pro–B‐type natriuretic peptide; PD, peritoneal dialysis; PhA, phase angle; PHDI, Planetary Health Diet Index; PSQI, Pittsburgh Sleep Quality Index; PTB, pulmonary tuberculosis; RCT, randomized controlled trial; RTR, renal transplant recipients; SAS, Self‐Rating Anxiety Scale; Scr, serum creatinine; SDS, Self‐Rating Depression Scale; SM/BW, skeletal muscle mass to body weight ratio; TG, triglycerides; UA, uric acid; VRI, vascular reactivity index; vWF, von Willebrand factor.

### 3.1 Quality Assessment

In the 41 cross‐sectional studies [5, 19, 21, 31, 32, 33, 34, 35, 36, 37, 38, 39, 40, 41, 42, 43, 44, 45, 46, 47, 48, 50, 52, 53, 55, 56, 58, 60, 63, 65, 66, 68, 71, 74, 75, 76, 79, 80, 81, 83, 84], 23 of them [5, 19, 31, 33, 38, 39, 40, 41, 42, 43, 46, 48, 50, 56, 66, 68, 71, 74, 76, 80, 81, 83, 84] were rated A and 18 of them [21, 32, 34, 35, 36, 37, 44, 45, 47, 52, 53, 55, 58, 60, 63, 65, 75, 79] were rated B. Eleven cohort studies [20, 49, 51, 54, 59, 61, 70, 72, 77, 78, 82] and 4 case–control studies [62, 64, 69, 73] were of high quality. Two RCT studies [57, 67] are considered to be at low risk of bias. Detailed results were presented in Data S2.

### 3.2 Meta‐Analysis Results

#### 3.2.1 General Factors

##### 3.2.1.1 Age

**FIGURE 2:**
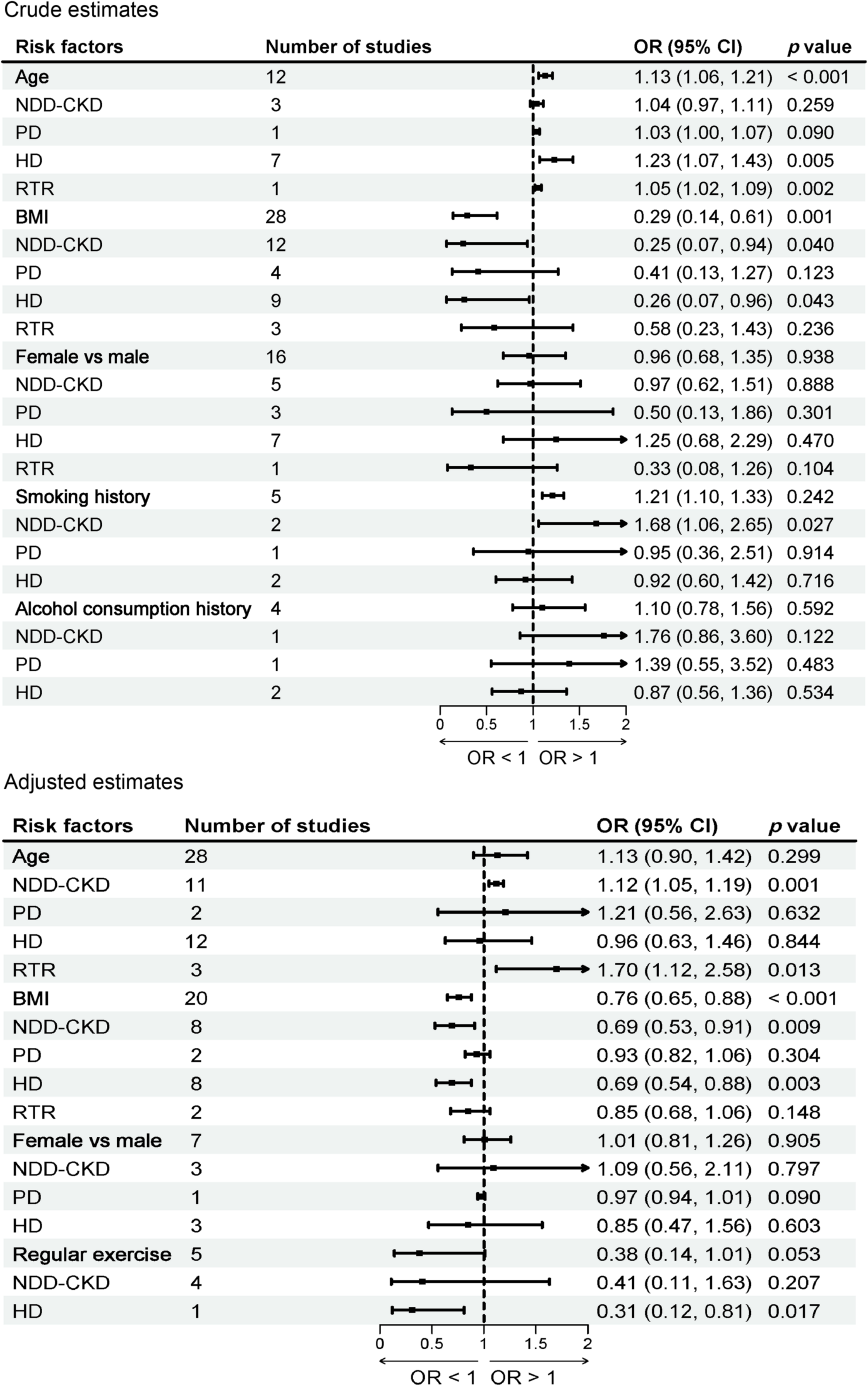
Meta‐analysis of the associations between general factors and sarcopenia in patients with CKD.

Thirty‐four studies [5, 19, 20, 21, 34, 35, 36, 37, 40, 42, 43, 44, 45, 46, 48, 53, 55, 57, 58, 59, 60, 61, 62, 63, 64, 65, 66, 67, 68, 69, 70, 71, 76, 81] reported the association between age and sarcopenia in patients with CKD. The meta‐analysis showed that each 1‐year increase in age was significantly associated with a higher risk of sarcopenia overall (crude OR = 1.13; 95% CI: 1.06–1.21; *I* ^2^ = 97.2%; *p* < 0.001; adjusted OR = 1.13; 95% CI: 0.90–1.42; *I* ^2^ = 99.8%; *p* = 0.299; Figure 2). Subgroup analysis based on treatment modality showed that a 1‐year increase in age was significantly associated with an increased risk of sarcopenia in patients with NDD‐CKD (adjusted OR = 1.12; 95% CI: 1.05–1.19; *p* = 0.001; Figure 2), RTR (adjusted OR = 1.70; 95% CI: 1.12–2.58; *p* = 0.013; Figure 2) and HD (crude OR = 1.23; 95% CI: 1.07–1.43; *p* = 0.005; adjusted OR = 0.96; 95% CI: 0.63–1.46; *p* = 0.844; Figure 2).

##### 3.2.1.2 BMI

Thirty‐three [20, 32, 34, 35, 36, 37, 39, 40, 42, 43, 44, 45, 48, 51, 53, 55, 57, 58, 59, 60, 63, 64, 66, 67, 68, 69, 70, 71, 72, 74, 81, 82, 85] reported the association between BMI and sarcopenia in patients with CKD. The meta‐analysis demonstrated that a higher BMI was significantly associated with a lower prevalence of sarcopenia (per 1 kg/m^2^ increase in BMI: adjusted OR = 0.76; 95% CI: 0.65–0.88; *I* ^2^ = 90.7%; *p* < 0.001; Figure 2). Subgroup analyses according to treatment modality showed that per 1 kg/m^2^ increase in BMI was significantly associated with a reduced risk of sarcopenia in patients with NDD‐CKD (adjusted OR = 0.69; 95% CI: 0.53–0.91; *p* = 0.009; Figure 2) and HD patients (adjusted OR = 0.69; 95% CI: 0.54–0.88; *p* = 0.003; Figure 2). In contrast, no significant associations were observed in PD patients or RTR patients.

##### 3.2.1.3 Smoking History

Five studies [20, 40, 55, 59, 67] reported the association between smoking history and sarcopenia in patients with CKD. Subgroup analyses according to treatment modality showed that in patients with NDD‐CKD, those with a history of smoking had a significantly higher risk of sarcopenia compared with those without a smoking history (crude OR = 1.68; 95% CI: 1.06–2.65; *p* = 0.027; Figure 2). In contrast, no significant associations were observed in PD patients or HD patients.

##### 3.2.1.4 Regular Exercise

Five studies [35, 40, 55, 60, 69] reported the association between regular exercise and sarcopenia in patients with CKD. Subgroup analyses according to treatment modality showed that, in HD patients, engaging in regular exercise was significantly associated with a lower risk of sarcopenia (adjusted OR = 0.31; 95% CI: 0.12–0.81; *p* = 0.017; Figure 2). No significant associations were observed in NDD‐CKD patients.

Additionally, the results of our meta‐analysis indicate that female sex, compared with male sex, was not significantly associated with the risk of sarcopenia in patients with CKD. Similarly, alcohol consumption history was also not significantly associated with sarcopenia risk. Subgroup analyses based on dialysis modality were conducted for each risk factor, and no significant associations were observed in any subgroup. Detailed results were presented in Figure 2.

#### 3.2.2 Comorbidities

##### 3.2.2.1 COPD

**FIGURE 3:**
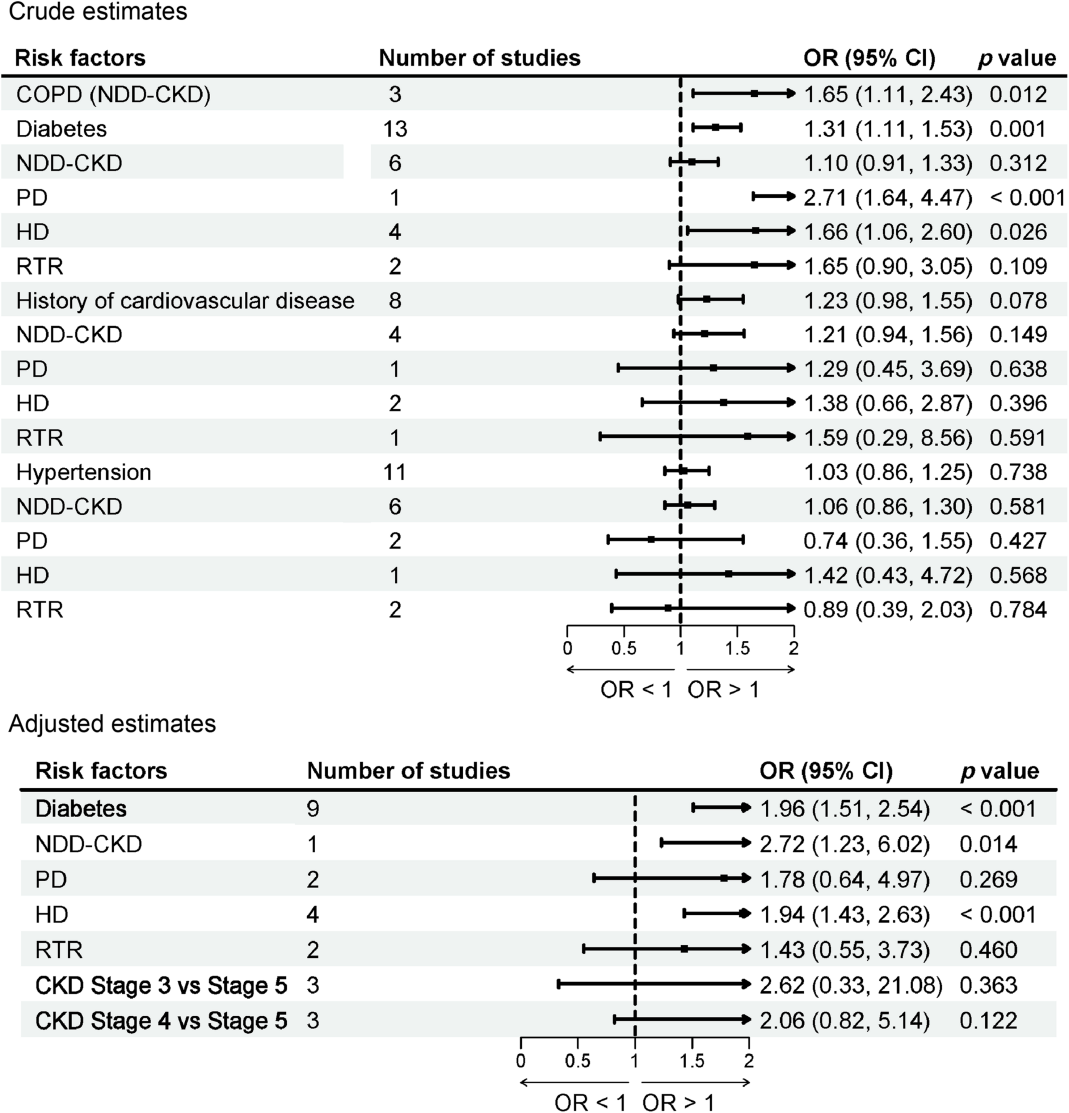
Meta‐analysis of the associations between comorbidities and sarcopenia in patients with CKD.

Three studies [48, 68, 69] reported the association between chronic obstructive pulmonary disease (COPD) and the risk of sarcopenia in patients with NDD‐CKD. The meta‐analysis demonstrated that the presence of COPD was significantly associated with a higher risk of sarcopenia (crude OR = 1.65; 95% CI: 1.11–2.43; *I* ^2^ = 0.0%; *p* = 0.012; Figure 3) in NDD‐CKD patients.

##### 3.2.2.2 Diabetes

Twenty‐two studies [5, 32, 34, 37, 40, 42, 44, 45, 48, 50, 55, 57, 58, 63, 64, 67, 68, 69, 74, 76, 79, 81] have examined the association between diabetes and the risk of sarcopenia in patients with CKD. The meta‐analysis based on adjusted ORs demonstrated that diabetes was significantly associated with an increased risk of sarcopenia (adjusted OR = 1.96; 95% CI: 1.51–2.54; *I* ^2^ = 0.0%; *p* < 0.001; Figure 3). Subgroup analyses according to treatment modality showed that diabetes was significantly associated with a higher risk of sarcopenia in NDD‐CKD patients (adjusted OR = 2.72; 95% CI: 1.23–6.02; *p* = 0.014; Figure 3), HD patients (adjusted OR = 1.94; 95% CI: 1.43–2.63; *p* < 0.001; Figure 3) and PD patients (crude OR = 2.71; 95% CI: 1.64–4.47; *p* < 0.001; adjusted OR = 1.78; 95% CI: 0.64–4.97; *p* = 0.269; Figure 3). In contrast, no significant associations were observed in RTR patients.

Additionally, results of our meta‐analysis indicate that there was no significant association between a history of cardiovascular disease, hypertension, CKD stage 3, or CKD stage 4 and the risk of sarcopenia in patients with CKD. Subgroup analyses based on dialysis modality were conducted for each risk factor, and no significant associations were observed in any subgroup. Detailed results were presented in Figure 3.

#### 3.2.3 Body Compositions

##### 3.2.3.1 Body Protein Content

**FIGURE 4:**
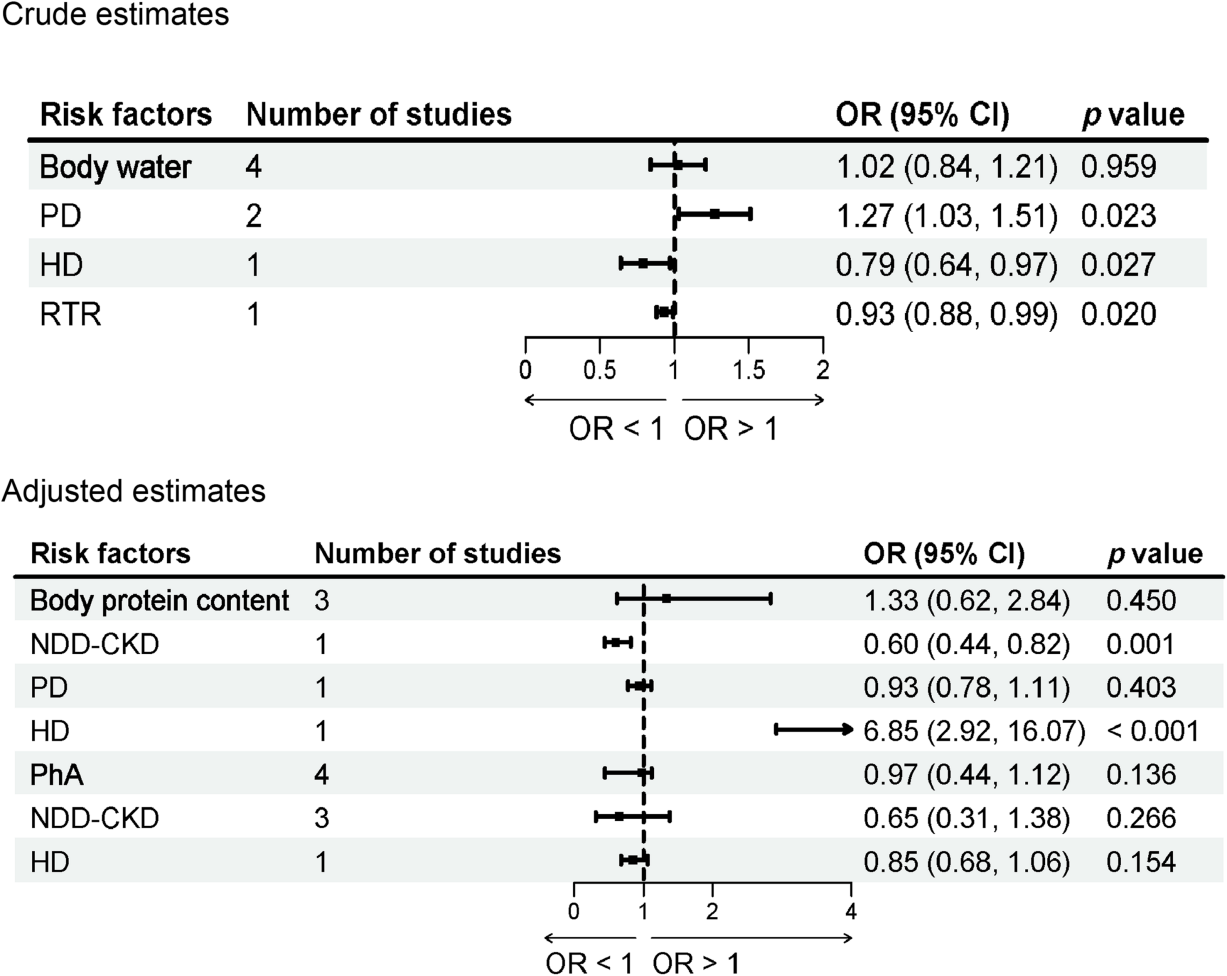
Meta‐analysis of the associations between body compositions and sarcopenia in patients with CKD.

Three studies [20, 52, 61] reported the association between body protein content and sarcopenia in patients with CKD. Subgroup analyses according to treatment modality showed that per 1 g/dL increase in body protein content was significantly associated with a lower risk of sarcopenia in NDD‐CKD patients (adjusted OR = 0.60; 95% CI: 0.44–0.82; *p* = 0.001; Figure 4). In contrast, per 1 g/dL increase in body protein content was significantly associated with a higher risk of sarcopenia in HD patients (adjusted OR = 6.85; 95% CI: 2.92–16.07; *p* < 0.001; Figure 4). No significant association was observed in PD patients.

##### 3.2.3.2 Body Water

Four studies [34, 44, 49, 52] reported on the association between body water and sarcopenia in patients with CKD. Subgroup analyses according to treatment modality showed that per 1 kg increase in body water was significantly associated with an increased risk of sarcopenia in PD patients (crude OR = 1.25; 95% CI: 1.03–1.51; *p* = 0.023; Figure 4). In contrast, per 1 kg increase in body water was significantly associated with a lower risk of sarcopenia in HD patients (crude OR = 0.79; 95% CI: 0.64–0.97; *p* = 0.027; Figure 4) and RTR patients (crude OR = 0.93; 95% CI: 0.88–0.99; *p* = 0.020; Figure 4).

Additionally, results of our meta‐analysis indicate that there was no significant association between phase angle (phA) and the risk of sarcopenia in patients with CKD. Subgroup analyses based on dialysis modality were conducted for each risk factor, and no significant associations were observed in any subgroup. Detailed results were presented in Figure 4.

#### 3.2.4 Blood‐Based Biomarkers

##### 3.2.4.1 Haemoglobin

**FIGURE 5:**
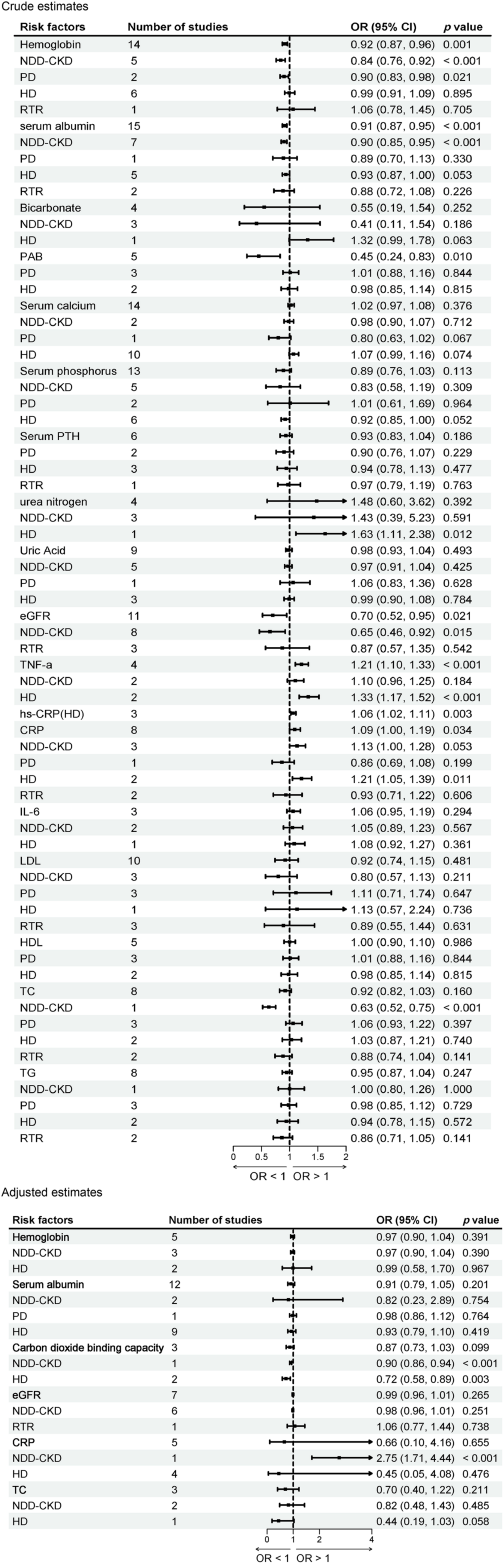
Meta‐analysis of the associations between blood‐based biomarkers and sarcopenia in patients with CKD.

Nineteen studies [32, 36, 39, 40, 48, 50, 51, 53, 54, 55, 60, 62, 67, 68, 69, 72, 79, 82, 85] reported the association between haemoglobin and sarcopenia in patients with CKD. The meta‐analysis showed that per 1 g/L increase in haemoglobin was significantly associated with a reduced risk of sarcopenia overall (crude OR = 0.915; 95% CI: 0.87–0.96; *I* ^2^ = 11.0%; *p* = 0.001; adjusted OR = 0.97; 95% CI: 0.90–1.04; *I* ^2^ = 0.0%; *p* = 0.391; Figure 5). Subgroup analyses demonstrated significant associations in NDD‐CKD patients (crude OR = 0.84; 95% CI: 0.76–0.92; *p* < 0.001; adjusted OR = 0.97; 95% CI: 0.90–1.04; *p* = 0.390; Figure 5) and PD patients (crude OR = 0.90; 95% CI: 0.83–0.98; *p* = 0.021; adjusted OR = 0.99; 95% CI: 0.58–1.90; *p* = 0.967; Figure 5), while no significant associations were observed in HD patients or RTR patients.

##### 3.2.4.2 Serum Albumin

Twenty‐seven studies [5, 19, 20, 32, 36, 39, 40, 48, 50, 51, 53, 55, 57, 60, 61, 62, 63, 64, 65, 67, 68, 72, 74, 79, 82, 83, 85] reported the association between serum albumin and sarcopenia in patients with CKD. The meta‐analysis showed that per 1 g/dL increase in serum albumin was significantly associated with a reduced risk of sarcopenia overall (crude OR = 0.91; 95% CI: 0.87–0.95; *I* ^2^ = 0.0%; *p* < 0.001; adjusted OR = 0.91; 95% CI: 0.79–1.05; *I* ^2^ = 67.5%; *p* = 0.201; Figure 5). Subgroup analyses demonstrated a significant association in NDD‐CKD patients (crude OR = 0.90; 95% CI: 0.85–0.95; *p* < 0.001; adjusted OR = 0.82; 95% CI: 0.23–2.89; *p* = 0.754; Figure 5), while no significant associations were observed in PD patients or HD patients.

##### 3.2.4.3 Carbon Dioxide Binding Capacity

Three studies [55, 61, 69] reported the association between carbon dioxide binding capacity and sarcopenia in patients with CKD. Subgroup analyses according to treatment modality showed that per 1 mmol/L increase in carbon dioxide binding capacity was significantly associated with a lower risk of sarcopenia in NDD‐CKD patients (adjusted OR = 0.90; 95% CI: 0.86–0.94; *p* < 0.001; Figure 5) and HD patients (adjusted OR = 0.72; 95% CI: 0.58–0.89; *p* = 0.003; Figure 5).

##### 3.2.4.4 Prealbumin (PAB)

Five studies [20, 44, 48, 51, 68] reported the association between serum prealbumin (PAB) and sarcopenia in patients with CKD. The meta‐analysis demonstrated that per 1 mg/L increase in PAB was significantly associated with a lower risk of sarcopenia overall (crude OR = 0.45; 95% CI: 0.24–0.83; *I* ^2^ = 97.7%; *p* = 0.010; Figure 5). Subgroup analyses according to treatment modality showed no significant associations in PD or HD patients.

##### 3.2.4.5 Urea Nitrogen

Four studies [36, 39, 55, 69] reported the association between urea nitrogen and sarcopenia in patients with CKD. The meta‐analysis demonstrated that this association was not statistically significant overall (crude OR = 1.48; 95% CI: 0.60–3.62; *I* ^2^ = 95.3%; *p* = 0.392; Figure 5). Subgroup analyses according to treatment modality showed that in HD patients, a per 1 mmol/L increase in urea nitrogen was significantly associated with an increased risk of sarcopenia (crude OR = 1.63; 95% CI: 1.11–2.38; *p* = 0.012; Figure 5). No significant associations were observed in NDD‐CKD patients.

##### 3.2.4.6 Estimated Glomerular Filtration Rate (eGFR)

Eleven studies [34, 35, 39, 40, 48, 57, 59, 61, 64, 69, 79] reported the association between eGFR and sarcopenia in patients with CKD. The meta‐analysis demonstrated that per 1 mL/min/1.73 m^2^ increase in eGFR was significantly associated with a lower risk of sarcopenia overall (crude OR = 0.70; 95% CI: 0.52–0.95; *I* ^2^ = 87.6%; *p* = 0.021; adjusted OR = 0.99; 95% CI: 0.96–1.01; *I* ^2^ = 38.5%; *p* = 0.265; Figure 5). Subgroup analyses showed that this association remained significant in patients with NDD‐CKD (crude OR = 0.65; 95% CI: 0.46–0.92; *p* = 0.015; adjusted OR = 0.98; 95% CI: 0.96–1.01; *p* = 0.251; Figure 5), while no significant associations were observed in RTR patients.

Additionally, results of our meta‐analysis indicated that there was no significant association between bicarbonate, serum calcium, serum parathyroid hormone (PTH), or uric acid and the risk of sarcopenia in patients with CKD. Subgroup analyses based on dialysis modality were conducted for each risk factor, and no significant associations were observed in any subgroup. Detailed results were presented in Figure 5.

##### 3.2.4.7 TNF‐α

Four studies [50, 55, 57, 60] reported the association between TNF‐α levels and sarcopenia in patients with CKD. The meta‐analysis demonstrated that per 1 pg./mL increase in TNF‐α was significantly associated with a higher risk of sarcopenia overall (crude OR = 1.21; 95% CI: 1.10–1.33; *I* ^2^ = 44.6%; *p* < 0.001; Figure 5). Subgroup analyses according to treatment modality showed that in HD patients, higher TNF‐α levels were significantly associated with an increased risk of sarcopenia (crude OR = 1.33; 95% CI: 1.17–1.52; *p* < 0.001; Figure 5), while no significant association was observed in NDD‐CKD patients.

##### 3.2.4.8 High‐Sensitivity C‐Reactive Protein (Hs‐CRP)

Three studies [45, 55, 62] reported the association between hs‐CRP and sarcopenia in patients with CKD. The meta‐analysis demonstrated that per 1 mg/L increase in hs‐CRP was significantly associated with a higher risk of sarcopenia overall (crude OR = 1.06; 95% CI: 1.02–1.11; *I* ^2^ = 0.0%; *p* = 0.003; Figure 5). Subgroup analysis according to treatment modality showed that this association remained significant in HD patients (crude OR = 1.06; 95% CI: 1.02–1.11; *p* = 0.003; Figure 5).

##### 3.2.4.9 C‐Reactive Protein (CRP)

Thirteen studies [20, 35, 44, 49, 50, 53, 60, 64, 66, 69, 72, 79, 82] reported the association between CRP and sarcopenia in patients with CKD. The meta‐analysis showed that a per 1 mg/L increase in CRP was significantly associated with a higher risk of sarcopenia overall (crude OR = 1.09; 95% CI: 1.00–1.19; *I* ^2^ = 29.7%; *p* = 0.034; adjusted OR = 0.66; 95% CI: 0.10–4.16; *I* ^2^ = 95.8%; *p* = 0.655; Figure 5). Subgroup analyses of adjusted ORs indicated that a per 1 mg/L increase in CRP was significantly associated with a higher risk of sarcopenia in NDD‐CKD patients (adjusted OR = 2.75; 95% CI: 1.71–4.44; *p* < 0.001; Figure 5) and HD patients (crude OR = 1.21; 95% CI: 1.05–1.39; *p* = 0.011; adjusted OR = 0.45; 95% CI: 0.05–4.08; *p* = 0.476; Figure 5). No significant associations were observed in PD or RTR patients.

Additionally, results of our meta‐analysis indicate that there was no significant association between interleukin‐6 (IL‐6) and the risk of sarcopenia in patients with CKD. Subgroup analyses based on dialysis modality were conducted for each risk factor, and no significant associations were observed in any subgroup. Detailed results were presented in Figure 5.

##### 3.2.4.10 Total Cholesterol (TC)

Ten studies [34, 37, 44, 48, 52, 55, 57, 61, 65, 67] reported the association between TC and sarcopenia in patients with CKD. Subgroup analyses according to treatment modality showed that in NDD‐CKD patients, per 1 mmol/L increase in TC was significantly associated with a lower risk of sarcopenia (crude OR = 0.63; 95% CI: 0.52–0.75; *p* < 0.001; adjusted OR = 0.41; 95% CI: 0.11–1.63; *p* = 0.207; Figure 5). No significant associations were observed in other subgroups.

Additionally, results of our meta‐analysis indicated that there was no significant association between low‐density lipoprotein cholesterol (LDL), high‐density lipoprotein cholesterol (HDL), or triglycerides (TG) and the risk of sarcopenia in patients with CKD. Subgroup analyses based on dialysis modality were conducted for each risk factor, and no significant associations were observed in any subgroup. Detailed results were presented in Figure 5.

#### 3.2.5 Treatment‐Related Factors

##### 3.2.5.1 Dialysis Vintage

**FIGURE 6:**
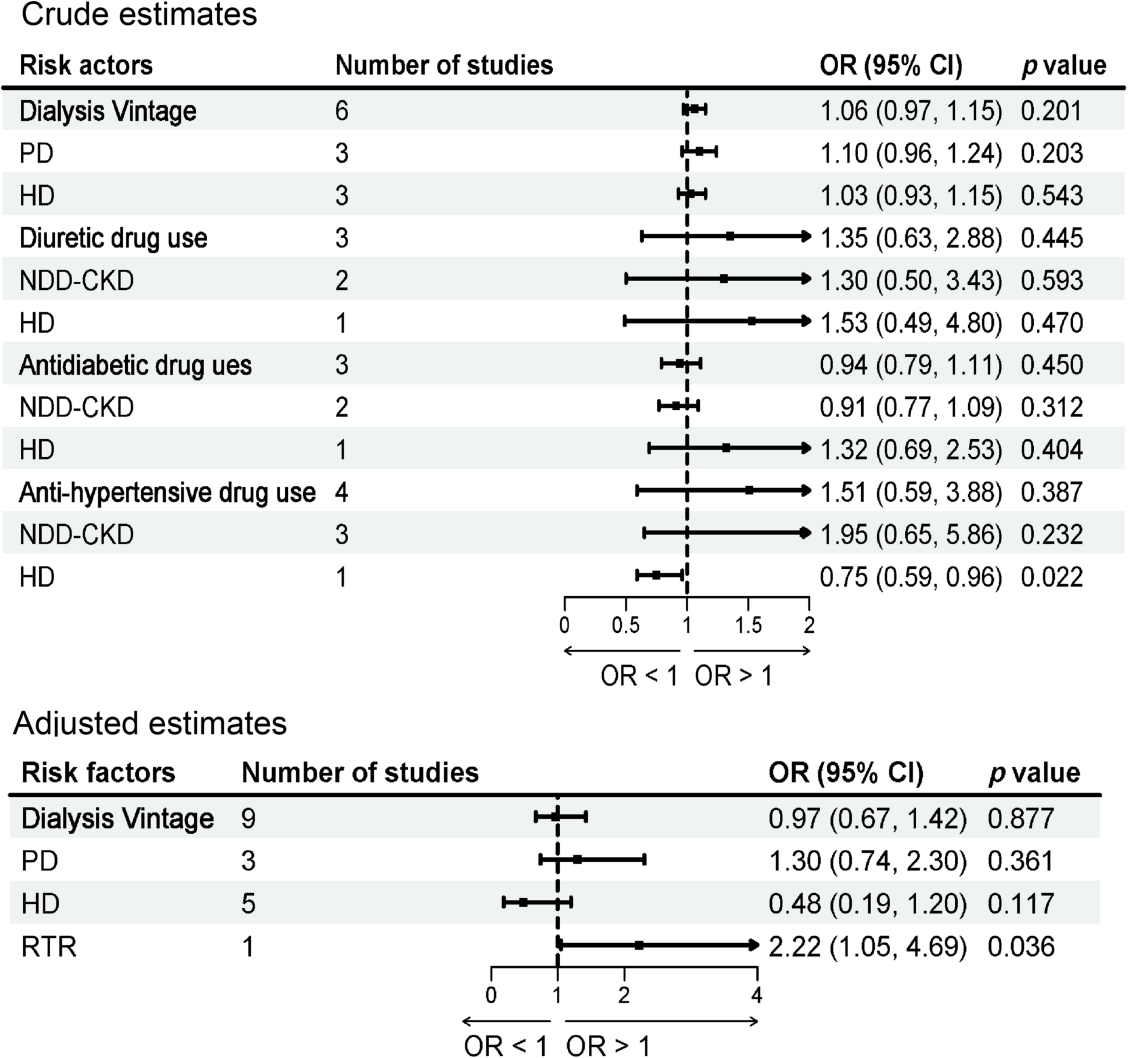
Meta‐analysis of the associations between treatment‐related factors and sarcopenia in patients with CKD.

Nine studies [5, 37, 38, 42, 50, 58, 62, 65, 67] reported the association between dialysis vintage and sarcopenia in patients with CKD. Subgroup analyses according to treatment modality showed that per 1 month increase in dialysis vintage was significantly associated with an increased risk of sarcopenia in RTR patients (adjusted OR = 2.22; 95% CI: 1.05–4.69; *p* = 0.036; Figure 6). No significant associations were observed in PD or HD patients.

##### 3.2.5.2 Anti‐Hypertensive Drug use

Four studies [40, 55, 60, 69] reported the association between anti‐hypertensive drug use and sarcopenia in patients with CKD. Subgroup analyses according to treatment modality showed that anti‐hypertensive drug use was significantly associated with a lower risk of sarcopenia in patients with HD (crude OR = 0.71; 95% CI: 0.54–0.93; *p* = 0.014; Figure 6).

Additionally, results of our meta‐analysis indicated that there was no significant association between the use of diuretics or drugs to treat diabetes and the risk of sarcopenia in patients with CKD. Subgroup analyses based on dialysis modality were conducted for each risk factor, and no significant associations were observed in any subgroup. Detailed results were presented in Figure 6.

### 3.3 Publication Bias

In this study, funnel plots and Egger's tests were performed for each individual factor with at least three included studies to assess publication bias. All funnel plots evaluating the risk of publication bias exhibited symmetry, and the results of Egger's tests were non‐significant (*p* > 0.05). For analyses assessing TC, the Egger's test indicated potential publication bias (*p* = 0.034 < 0.5). Therefore, the trim‐and‐fill method was applied, which imputed two potentially missing studies to achieve funnel plot symmetry. After adjustment, the pooled effect estimate did not change substantially, suggesting that the results for total cholesterol were robust to potential publication bias. For analyses in which funnel plot asymmetry was observed, the trim‐and‐fill method was applied, and the adjusted estimates did not differ substantially from the original results. These findings indicate that there was no evidence of publication bias among the included studies. Detailed results are presented in Data S3.

### 3.4 Heterogeneity and Sensitivity Analysis

For all risk factors exhibiting substantial heterogeneity, we conducted subgroup analyses (based on crowd source, sarcopenia definition, study design, muscle mass assessment method, and geographic region), sensitivity analyses by sequentially excluding individual studies, and meta‐regression analyses to explore potential sources of heterogeneity.

For the factors of serum albumin, PhA, diuretic use, and anti‐hypertensive drug use, the leave‐one‐out sensitivity analysis showed that, for each factor, the exclusion of a specific individual study (not the same study across all factors) substantially reduced heterogeneity. To ensure transparency and comprehensiveness, we retained all studies in the meta‐analysis and discussed the potential influence of these individual studies on the robustness of the findings. For the factors of CKD stages 3, body water, subgroup analysis indicated that the definition of Sarcopenia is the source of heterogeneity. However, for age, BMI, body protein content, dialysis vintage, HDL, and serum phosphorus, the corresponding analyses did not identify any specific sources of heterogeneity. Detailed results are presented in Data S3.

## 4 Discussion

Fifty‐eight original studies were included in this research. Potential risk factors for sarcopenia in patients with CKD encompass older age, lower BMI, diabetes and so on. Risk factors are different for different populations such as NDD‐CKD, HD, PD, and RTR. The most significant aspects of human aging include alterations in physiology and body composition, particularly the modifications or redistribution of muscle and adipose tissue, despite the overall body weight being constant. As age progresses, the disrupted equilibrium between protein synthesis and proteolysis in skeletal muscle leads to a gradual reduction in muscle mass, strength, and functionality [6, 86]. Given that the majority of CKD patients are elderly, it is crucial to evaluate and monitor sarcopenia in all patients.

Our findings indicated that for each 1 kg/m^2^ decrease in BMI, the risk of sarcopenia increased among patients with CKD, with this association being particularly pronounced in both NDD‐CKD and HD patients. In patients with CKD, a reduced BMI often indicates decreased nutritional intake and poorer energy reserves, potentially aggravating muscle catabolism in this population [87]. In addition, patients undergoing dialysis often experience chronic inflammation and metabolic acidosis, which further accelerates the degradation of muscle proteins and also affects muscle protein synthesis [88]. Nutritional guidelines [89] also suggest that for adult patients with CKD on maintenance dialysis, it is recommended that overweight/obesity status (based on BMI) can be used as a prospective indicator of lower mortality, while underweight status and morbid obesity (based on BMI) can be used as prospective indicators of higher mortality. This further emphasizes the importance of maintaining adequate nutritional status in patients with CKD to mitigate muscle loss and its associated adverse outcomes in this population.

Our findings indicate that engaging in regular exercise is associated with a reduced incidence of sarcopenia among patients undergoing haemodialysis. Exercise rehabilitation can enhance metabolic rate, promote muscle protein synthesis, and reduce muscle catabolism in these patients. It also improves physical function and nutritional status, helps with weight management, enhances cardiovascular health, increases bone mineral density, and alleviates pain [90]. In our study, the result showed there is a significant association between smoking and sarcopenia among patients with NDD‐CKD. While numerous studies [20, 40, 55, 59, 67] indicated that alcohol consumption diminishes muscle mass and strength, hence fostering sarcopenia, this correlation has not been notably observed in patients with CKD in our study. The KDIGO 2024 Clinical Practice Guideline points out that smoking indirectly induces sarcopenia by accelerating the deterioration of renal function and metabolic disorders (such as vitamin D deficiency and insulin resistance), and quitting smoking is a core measure for the comprehensive management of CKD [91]. The impact of alcohol consumption on patients with CKD may not be fully elucidated in certain studies, owing to the predominance of more significant risk factors for sarcopenia, such as inflammation and metabolic disorders, which obscure the effects of these behaviours, as well as limitations in sample sizes and follow‐up durations. The adverse effects of smoking and alcohol consumption on the general health of CKD patients remain. Effective interventions targeting these modifiable risk factors are essential to reduce the incidence of sarcopenia among patients with CKD.

Patients with COPD experience chronic hypoxia and oxidative stress, resulting in mitochondrial dysfunction and subsequent impairment of muscle cell energy consumption [92]. Oxidative stress is a significant risk factor for patients with NDD‐CKD, as the deterioration of renal function results in the accumulation of oxidative byproducts in the body, which are crucial for preserving muscle function [93]. The interplay of COPD and CKD markedly diminishes patients' muscle synthesis capacity, resulting in elevating the prevalence of sarcopenia. The results in our study showed that diabetes is a risk factor for sarcopenia in patients with CKD. CKD patients usually have metabolic disorders, including insulin resistance, which can interfere with the amount and timing of glucose uptake by skeletal muscle and reduce the availability of energy required for muscle maintenance and repair [94]. This leads to increased muscle protein degradation and reduced muscle synthesis, which in turn leads to the development of sarcopenia. Consequently, we must consider certain comorbidities in CKD patients that may elevate the incidence of sarcopenia. Anaemia is a common symptom in patients with CKD, caused by insufficient erythropoietin (EPO) production, abnormal iron metabolism, blood loss, inflammation, nutrient deficiencies and oxidative stress [95]. Although some studies [40, 59, 69] have suggested that the prevalence of sarcopenia may increase with advancing CKD stages, the current evidence on this association remains inconsistent. Our meta‐analysis also did not find a significant positive correlation between CKD stage and sarcopenia risk. Nevertheless, proactive management at various stages of CKD to prevent or mitigate sarcopenia remains critically important.

Our research indicated that elevated body water and diminished body protein levels are risk factors for sarcopenia in NDD‐CKD patients, but the converse applies to PD patients. In dialysis patients, extra water must be eliminated during dialysis, and in patients with decreased intracellular water (ICW) and muscle mass, total body water measures are likely to be low [49]. In patients with dialysis sarcopenia, muscle breakdown is a result of inflammation and metabolic changes, while elevated levels of non‐muscle‐derived proteins (e.g., inflammatory proteins) contribute to elevated BPC measurements that obscure the true sarcopenic state. Thus, for dialysis patients, low body water and high BPC values are often indicative of reduced muscle mass and poor nutritional status, and are strongly associated with an elevated risk of sarcopenia. In contrast, increased body water in patients with NDD‐CKD is usually indicative of volume overload due to decreased renal function.

Our results suggest that low eGFR increases sarcopenia in patients with NDD‐CKD. Our subgroup analysis based on eGFRcre and eGFRcye revealed a difference in results. Creatinine‐based estimated eGFR may overestimate kidney function in patients with sarcopenia, whereas cystatin C‐based eGFR is less influenced by muscle mass [96]. Reduced levels of albumin and prealbumin are common in the population with CKD in our study. The 2020 Kidney Disease Outcomes Quality Initiative (KDOQI) guidelines for CKD nutrition recognize that albumin and/or serum prealbumin may be considered supplemental tools for assessing nutritional status [89]. Providing effective measures to improve the nutritional status of patients with CKD is strongly recommended.

This study confirmed the significant association between elevated inflammatory markers (including TNF‐α and CRP), and sarcopenia among patients with CKD. In CKD patients, chronic inflammation affects the quality of the uptake mechanism through various mechanisms. The increase in inflammatory factors such as TNF‐α, can accelerate the loss of muscle protein [97, 98]. Current research [97] indicated that diminished serum albumin levels correlate with decreased muscle mass in relatively healthy, well‐nourished elderly individuals, and that serum albumin concentrations are typically linked to chronic inflammation. Chronic inflammation can also increase the permeability of capillaries, causing serum albumin to leak from blood vessels into tissue transparency, thereby reducing serum albumin concentration [98]. Consequently, it is crucial to monitor the inflammatory condition of CKD patients and implement prompt interventions.

Studies have also shown that factors such as dialysis vintage, low basal metabolic rate [34, 37], low bone mass [57, 66], high Charlson comorbidity index (CCI) [44, 49], lowdaily steps [62, 69], low functional capacity [35, 39], and high β2‐microglobulin (β2‐MG) [20, 55] are risk factors for the development of sarcopenia in patients with CKD. Nevertheless, owing to the paucity of pertinent research in the literature, we were unable to perform a more comprehensive study of these characteristics. Additional research is required in the future for further verification and elucidation.

Clinically, bioelectrical impedance analysis (BIA) is commonly used to assess muscle mass in patients. However, in dialysis patients, BIA and body composition measurements are greatly affected by hydration status and electrolyte fluctuations, which may lead to an overestimation of body protein content while underestimating functional muscle mass [99, 100]. In different stages of CKD, there is a complex interplay between hydration status, nutritional status, inflammation, and measurement artefacts, necessitating cautious interpretation of body composition parameters [100]. It is essential to combine body composition assessments with functional measurements, such as handgrip strength and gait speed, to guide accurate diagnosis and personalized intervention strategies. In the case of CKD patients with low BMI and co‐morbidities, effective interventions are needed to reduce the level of inflammation in the body and increase the protein content, which in turn improves nutritional levels and increases muscle mass.

This study has numerous advantages. This was a thorough meta‐analysis examining the related risk variables in individuals with CKD, offering essential baseline data and a reference for further research in this domain. Secondly, the study performed a thorough examination of various databases to guarantee the inclusivity and representativeness of the selected studies, employing a meticulous search strategy to encompass a broad spectrum of pertinent literature, thereby augmenting the study's comprehensiveness and scientific rigor. In addition, our analysis conducted detailed subgroup evaluations across different CKD populations, including patients with NDD‐CKD, PD, HD and RTR, enabling a more precise understanding of risk patterns within these specific subgroups. The quality of the included studies was meticulously evaluated, resulting in a high overall research quality that enhanced the reliability of the study's conclusions.

However, this study has limitations. This systematic review excluded articles written in languages other than English and Chinese, potentially excluding pertinent studies and impacting the generalizability of the findings. Secondly, while the primary sources of heterogeneity in most studies were discernible via meta‐analysis, significant heterogeneity persisted post‐data amalgamation, and the sources of heterogeneity in certain studies remained ambiguous, despite the application of meta‐regression and subgroup analysis. Moreover, the included studies originated from various countries and timeframes, thus introducing the effects of geographical and temporal variables, with disparate diagnostic criteria and measurement techniques for sarcopenia. This has partially impacted the consistency and comparability of the outcomes. The insufficient sample size may have compromised the accuracy of the statistical analysis results and hindered a comprehensive representation of the real influence of these risk factors across various groups. Consequently, while this study offers initial insights, additional high‐quality research with adequate sample numbers is necessary to further validate these specific risk variables, thereby enhancing the universality and dependability of the findings.

## 5 Conclusions

This meta‐analysis comprehensively demonstrated that sarcopenia in CKD is influenced by a multifactorial interplay of demographic characteristics, comorbidities, nutritional and body composition indicators, biochemical and metabolic markers, inflammation, and treatment‐related factors. This study comprehensively illustrated that the development of sarcopenia in patients with CKD is influenced by a variety of risk factors across various domains. The identification of patients at high risk of sarcopenia who could benefit from enhanced prophylaxis and treatment can be facilitated by the knowledge of risk factors that have a strong association with sarcopenia in patients with CKD. It is imperative to prioritize the identification of modifiable risk factors in order to enhance the effectiveness of prevention and treatment.

## Funding

This work was supported by the Zhejiang Provincial Natural Science Foundation (No. LZ25H270001), National College Students Innovation and Entrepreneurship Training Program (No. 202410344020) and Zhejiang Provincial Xinmiao Talents Program (New Young Talent Program) for college students (No. 2024R410).

## Conflicts of Interest

The authors declare no conflicts of interest.

## Supporting information


**Data S1:** Supplementary Information.

**Data S2:** Supplementary Information.

**Data S3:** Supplementary Information.

**Data S4:** Supplementary Information.

## Data Availability

The original contributions presented in the study are included in the article/Supplementary Material. Further inquiries can be directed to the corresponding authors.
