# Supplementary material for: Risk Factors Associated With Sarcopenia in Patients With Chronic Kidney Disease: A Systematic Review and Meta‐Analysis
Source: J Cachexia Sarcopenia Muscle. 2025 Dec 28;17(1):e70166. doi: 10.1002/jcsm.70166 (PMC12745342; doi:10.1002/jcsm.70166)
Supplement: Supplementary file 1 — Data S1: Supplementary Information. [file JCSM-17-e70166-s004.docx]

**Supporting material 1**

*Risk Factors Associated with Sarcopenia in Patients With chronic kidney disease: A Systematic Review and Meta-analysis*

*Table S1 Search strategy*

*Table S1.1 Search strategy by Pubmed*

|  | *Pubmed* | *Results* |
| --- | --- | --- |
| *#1* | *"renal insufficiency, chronic"[MeSH Terms] OR "renal insufficiency, chronic"[MeSH Terms] OR ("renal"[All Fields] AND "insufficiency"[All Fields] AND "chronic"[All Fields]) OR "chronic renal insufficiency"[All Fields] OR ("chronic"[All Fields] AND "renal"[All Fields] AND "insufficiencies"[All Fields]) OR "chronic renal insufficiencies"[All Fields] OR ("renal insufficiency, chronic"[MeSH Terms] OR ("renal"[All Fields] AND "insufficiency"[All Fields] AND "chronic"[All Fields]) OR "chronic renal insufficiency"[All Fields] OR ("renal"[All Fields] AND "insufficiencies"[All Fields] AND "chronic"[All Fields]) OR "renal insufficiencies chronic"[All Fields]) OR ("renal insufficiency, chronic"[MeSH Terms] OR ("renal"[All Fields] AND "insufficiency"[All Fields] AND "chronic"[All Fields]) OR "chronic renal insufficiency"[All Fields] OR ("chronic"[All Fields] AND "renal"[All Fields] AND "insufficiency"[All Fields])) OR ("renal insufficiency, chronic"[MeSH Terms] OR ("renal"[All Fields] AND "insufficiency"[All Fields] AND "chronic"[All Fields]) OR "chronic renal insufficiency"[All Fields] OR ("kidney"[All Fields] AND "insufficiency"[All Fields] AND "chronic"[All Fields]) OR "kidney insufficiency chronic"[All Fields]) OR ("renal insufficiency, chronic"[MeSH Terms] OR ("renal"[All Fields] AND "insufficiency"[All Fields] AND "chronic"[All Fields]) OR "chronic renal insufficiency"[All Fields] OR ("chronic"[All Fields] AND "kidney"[All Fields] AND "insufficiency"[All Fields]) OR "chronic kidney insufficiency"[All Fields]) OR ("renal insufficiency, chronic"[MeSH Terms] OR ("renal"[All Fields] AND "insufficiency"[All Fields] AND "chronic"[All Fields]) OR "chronic renal insufficiency"[All Fields] OR ("chronic"[All Fields] AND "kidney"[All Fields] AND "insufficiencies"[All Fields])) OR ("renal insufficiency, chronic"[MeSH Terms] OR ("renal"[All Fields] AND "insufficiency"[All Fields] AND "chronic"[All Fields]) OR "chronic renal insufficiency"[All Fields] OR ("kidney"[All Fields] AND "insufficiencies"[All Fields] AND "chronic"[All Fields])) OR ("renal insufficiency, chronic"[MeSH Terms] OR ("renal"[All Fields] AND "insufficiency"[All Fields] AND "chronic"[All Fields]) OR "chronic renal insufficiency"[All Fields] OR ("chronic"[All Fields] AND "kidney"[All Fields] AND "diseases"[All Fields]) OR "chronic kidney diseases"[All Fields]) OR ("renal insufficiency, chronic"[MeSH Terms] OR ("renal"[All Fields] AND "insufficiency"[All Fields] AND "chronic"[All Fields]) OR "chronic renal insufficiency"[All Fields] OR ("chronic"[All Fields] AND "kidney"[All Fields] AND "disease"[All Fields]) OR "chronic kidney disease"[All Fields]) OR ("renal insufficiency, chronic"[MeSH Terms] OR ("renal"[All Fields] AND "insufficiency"[All Fields] AND "chronic"[All Fields]) OR "chronic renal insufficiency"[All Fields] OR ("disease"[All Fields] AND "chronic"[All Fields] AND "kidney"[All Fields]) OR "disease chronic kidney"[All Fields]) OR ("renal insufficiency, chronic"[MeSH Terms] OR ("renal"[All Fields] AND "insufficiency"[All Fields] AND "chronic"[All Fields]) OR "chronic renal insufficiency"[All Fields] OR ("diseases"[All Fields] AND "chronic"[All Fields] AND "kidney"[All Fields]) OR "diseases chronic kidney"[All Fields]) OR ("renal insufficiency, chronic"[MeSH Terms] OR ("renal"[All Fields] AND "insufficiency"[All Fields] AND "chronic"[All Fields]) OR "chronic renal insufficiency"[All Fields] OR ("kidney"[All Fields] AND "disease"[All Fields] AND "chronic"[All Fields]) OR "kidney disease chronic"[All Fields]) OR ("renal insufficiency, chronic"[MeSH Terms] OR ("renal"[All Fields] AND "insufficiency"[All Fields] AND "chronic"[All Fields]) OR "chronic renal insufficiency"[All Fields] OR ("kidney"[All Fields] AND "diseases"[All Fields] AND "chronic"[All Fields]) OR "kidney diseases chronic"[All Fields]) OR ("renal insufficiency, chronic"[MeSH Terms] OR ("renal"[All Fields] AND "insufficiency"[All Fields] AND "chronic"[All Fields]) OR "chronic renal insufficiency"[All Fields] OR ("chronic"[All Fields] AND "renal"[All Fields] AND "diseases"[All Fields]) OR "chronic renal diseases"[All Fields]) OR ("renal insufficiency, chronic"[MeSH Terms] OR ("renal"[All Fields] AND "insufficiency"[All Fields] AND "chronic"[All Fields]) OR "chronic renal insufficiency"[All Fields] OR ("chronic"[All Fields] AND "renal"[All Fields] AND "disease"[All Fields]) OR "chronic renal disease"[All Fields] OR "kidney failure, chronic"[MeSH Terms] OR ("kidney"[All Fields] AND "failure"[All Fields] AND "chronic"[All Fields]) OR "chronic kidney failure"[All Fields]) OR ("renal insufficiency, chronic"[MeSH Terms] OR ("renal"[All Fields] AND "insufficiency"[All Fields] AND "chronic"[All Fields]) OR "chronic renal insufficiency"[All Fields] OR ("disease"[All Fields] AND "chronic"[All Fields] AND "renal"[All Fields]) OR "disease chronic renal"[All Fields]) OR ("renal insufficiency, chronic"[MeSH Terms] OR ("renal"[All Fields] AND "insufficiency"[All Fields] AND "chronic"[All Fields]) OR "chronic renal insufficiency"[All Fields] OR ("diseases"[All Fields] AND "chronic"[All Fields] AND "renal"[All Fields]) OR "diseases chronic renal"[All Fields]) OR ("renal insufficiency, chronic"[MeSH Terms] OR ("renal"[All Fields] AND "insufficiency"[All Fields] AND "chronic"[All Fields]) OR "chronic renal insufficiency"[All Fields] OR ("renal"[All Fields] AND "disease"[All Fields] AND "chronic"[All Fields]) OR "renal disease chronic"[All Fields]) OR ("renal insufficiency, chronic"[MeSH Terms] OR ("renal"[All Fields] AND "insufficiency"[All Fields] AND "chronic"[All Fields]) OR "chronic renal insufficiency"[All Fields] OR ("renal"[All Fields] AND "diseases"[All Fields] AND "chronic"[All Fields]) OR "renal diseases chronic"[All Fields])* | *378421* |
| *#2* | *"sarcopenia"[MeSH Terms] OR "sarcopenia"[MeSH Terms] OR "sarcopenia"[All Fields] OR "sarcopenias"[All Fields] OR (("muscle s"[All Fields] OR "muscles"[MeSH Terms] OR "muscles"[All Fields] OR "muscle"[All Fields]) AND* | *238689* |
| *#3* | *"risk factors"[MeSH Terms] OR "risk factors"[MeSH Terms] OR ("risk"[All Fields] AND "factors"[All Fields]) OR "risk factors"[All Fields] OR ("factor"[All Fields] AND "risk"[All Fields]) OR "factor risk"[All Fields] OR* | *3284168* |
| *#4* | *#1 AND #2 AND #3* | *675* |

*Table S1.2 Search strategy by Embase*

|  | *Embase* | *Results* |
| --- | --- | --- |
| *#1* | *('Chronic Renal Insufficiencies' or 'Renal Insufficiencies, Chronic' or 'Chronic Renal Insufficiency' or 'Kidney Insufficiency, Chronic' or 'Chronic Kidney Insufficiency' or 'Chronic Kidney Insufficiencies' or 'Kidney Insufficiencies, Chronic' or 'Chronic Kidney Diseases' or 'Chronic Kidney Disease' or 'Disease, Chronic Kidney' or 'Diseases, Chronic Kidney' or 'Kidney Disease, Chronic' or 'Kidney Diseases, Chronic' or 'Chronic Renal Diseases' or 'Chronic Renal Disease' or 'Disease, Chronic Renal' or 'Diseases, Chronic Renal' or 'Renal Disease, Chronic' or 'Renal Diseases, Chronic').mp. [mp=title, abstract, heading word, drug trade name, original title, device manufacturer, drug manufacturer, device trade name, keyword heading word, floating subheading word, candidate term word]* | *157211* |
| *#2* | *('Sarcopenia' or 'Muscular Atrophy' or 'Sarcopenias' or 'muscle mass' or 'muscle strength' or 'Atrophies, Muscular' or 'Atrophy, Muscular' or 'Muscular Atrophies' or 'Atrophy, Muscle' or 'Atrophies, Muscle' or 'Muscle Atrophies' or 'Muscle Atrophy' or 'Neurogenic Muscular Atrophy' or 'Atrophies, Neurogenic Muscular' or 'Atrophy, Neurogenic Muscular' or 'Muscular Atrophies, Neurogenic' or 'Muscular Atrophy, Neurogenic' or 'Neurogenic Muscular Atrophies' or 'Neurotrophic Muscular Atrophy' or 'Atrophies, Neurotrophic Muscular' or 'Atrophy, Neurotrophic Muscular' or 'Muscular Atrophies, Neurotrophic' or 'Muscular Atrophy, Neurotrophic' or 'Neurotrophic Muscular Atrophies').mp. [mp=title, abstract, heading word, drug trade name, original title, device manufacturer, drug manufacturer, device trade name, keyword heading word, floating subheading word, candidate term word]* | *204013* |
| *#3* | *('Factor, Risk' or 'Risk Factor' or 'Social Risk Factors' or 'Factor, Social Risk' or 'Factors, Social Risk' or 'Risk Factor, Social' or 'Risk Factors, Social' or 'Social Risk Factor' or 'Health Correlates' or 'Correlates, Health' or 'Population at Risk' or 'Populations at Risk' or 'Risk Scores' or 'Risk Score' or 'Score, Risk' or 'Risk Factor Scores' or 'Risk Factor Score' or 'Score, Risk Factor' or 'risk factors').mp. [mp=title, abstract, heading word, drug trade name, original title, device manufacturer, drug manufacturer, device trade name, keyword heading word, floating subheading word, candidate term word]* | *1915312* |
| *#4* | *#1 AND #2 AND #3* | *380* |

*Table S1.3 Search strategy by Web of science*

|  | *Web of science* | *Results* |
| --- | --- | --- |
| *#1* | *TS=(Chronic Renal insufficiencies OR Chronic Renal insufficiency OR Chronic KidneyInsufficiency OR Chronic Kidney Diseases OR Chronic Kidney Disease OR Chronic Renal Diseases OR Chronic Renal Disease) and Preprint Citation Index (Exclude-Database]* | *296307* |
| *#2* | *TS=(Muscular Atrophies, Neurogenic OR Muscular Atrophy, Neurogenic OR NeurogenicMuscular Atrophies OR Neurotrophic Muscular Atrophy OR Atrophies,Neurotrophic Muscular OR Atrophy, Neurotrophic Muscular OR MuscularAtrophies, Neurotrophic OR Muscular Atrophy, Neurotrophic OR NeurotrophicMuscular Atrophies) and Preprint Citation Index (Exclude - Database)* | *295153* |
| *#3* | *TS=(Factors, Social Risk OR Risk Factor, Social OR Risk Factors, Social OR Social RiskFactor OR Health Correlates OR Correlates, Health OR Population at Risk ORPopulations at Risk OR Risk Scores OR Risk Score OR Score, Risk OR Risk FactorScores OR Risk Factor Score OR Score, Risk Factor) and Preprint Citation index(Exclude- Database)* | *3330859* |
| *#4* | *#1 AND #2 AND #3* | *1041* |

*Table S1.4 Search strategy by Wanfang*

|  | *Wanfang* | *Results* |
| --- | --- | --- |
| *#1* | *慢性肾脏病**（主题） + 慢性肾脏疾**病（主题） + 慢性肾功能不全（主题）+ 慢性肾病（主题）* | *49057* |
| *#2* | *肌少症（主题） + 肌肉减少症（主题）+ 肌减少症（主题）* | *124261* |
| *#3* | *因素（主题） + 影响因素（主题） + 健康相关（主题） + 风险因素（主题） + 社会风险因素（主题） + 风险得分（主题）* | *6463041* |
| *#4* | *#1 AND #2 AND #3* | *109* |

*Table S1.5 Search strategy by CNKI*

|  | *CNKI* | *Results* |
| --- | --- | --- |
| *#1* | *慢性肾病（篇关摘） + 慢性肾脏疾病（篇关摘） + 慢性肾功能不全（篇关摘）+ 肾功能不全、慢性（篇关摘）* | *17401* |
| *#2* | *肌少症（篇关摘） + 肌肉减少症（篇关摘）+ 肌减少症（篇关摘）* | *3398* |
| *#3* | *因素（篇关摘） + 风险（篇关摘） + 健康相关（篇关摘） + 风险因素（篇关摘） + 社会风险因素（篇关摘） + 风险得分（篇关摘）* | *7250729* |
| *#4* | *#1 AND #2 AND #3* | *624* |

*Table S1.6 Search strategy by VIP*

|  | *VIP* | *Results* |
| --- | --- | --- |
| *#1* | *[肾功能不全, 慢性](https://jksmed.juhe.com.cn/#/subject/detail/D051436)（题名/关键词）* | *3057* |
| *#2* | *肌肉减少症（题名/关键词）* | *2016* |
| *#3* | *影响因素分析（题名/关键词）* | *164250* |
| *#4* | *#1 AND #2 AND #3* | *409* |

*Table S1.7 Search strategy by Sinomed*

|  | *Sinomed* | *Results* |
| --- | --- | --- |
| *#1* | *慢性肾病**（主题） + 慢性肾脏疾病（主题） + 慢性肾功能不全（主题）+ 肾功能不全、慢性（主题）* | *27763* |
| *#2* | *肌少症**（主题） + 肌肉减少症（主题）+ 肌减少症（主题）* | *2216* |
| *#3* | *因素（主题） + 影响因素（主题） + 健康相关（主题） + 风险因素（主题） + 社会风险因素（主题） + 风险得分（主题）* | *1290467* |
| *#4* | *#1 AND #2 AND #3* | *7* |

*Table S1.8 Search strategy by CMAJD*

|  | *Chinese Medical Association Journal Database* | *Results* |
| --- | --- | --- |
| *#1* | *透析（全部字段）* | *7711* |
| *#2* | *肌少症（全部字段）* | *305* |
| *#3* | *因素（全部字段）* | *110161* |
| *#4* | *#1 AND #2 AND #3* | *16* |

*Table S1.9 Search strategies for supplementary literature retrieval (July 2024–May 2025)*

| *Search strategies for supplementary literature retrieval (July 2024–May 2025)* | | |
| --- | --- | --- |
| *Database* | **Query** | *Results* |
| *Pubmed* | Search: ****((Chronic Renal Insufficiencies OR Renal Insufficiencies, Chronic OR Chronic Renal Insufficiency OR Kidney Insufficiency, Chronic OR Chronic Kidney Insufficiency OR Chronic Kidney Insufficiencies OR Kidney Insufficiencies, Chronic OR Chronic Kidney Diseases OR Chronic Kidney Disease OR Disease, Chronic Kidney OR Diseases, Chronic Kidney OR Kidney Disease, Chronic OR Kidney Diseases, Chronic OR Chronic Renal Diseases OR Chronic Renal Disease OR Disease, Chronic Renal OR Diseases, Chronic Renal OR Renal Disease, Chronic OR Renal Diseases, Chronic) AND (Sarcopenias OR muscle mass OR muscle strength OR Atrophies, Muscular OR Atrophy, Muscular OR Muscular Atrophies OR Atrophy, Muscle OR Atrophies, Muscle OR Muscle Atrophies OR Muscle Atrophy OR Neurogenic Muscular Atrophy OR Atrophies, Neurogenic Muscular OR Atrophy, Neurogenic Muscular OR Muscular Atrophies, Neurogenic OR Muscular Atrophy, Neurogenic OR Neurogenic Muscular Atrophies OR Neurotrophic Muscular Atrophy OR Atrophies, Neurotrophic Muscular OR Atrophy, Neurotrophic Muscular OR Muscular Atrophies, Neurotrophic OR Muscular Atrophy, Neurotrophic OR Neurotrophic Muscular Atrophies)) AND (Factor, Risk OR Risk Factor OR Social Risk Factors OR Factor, Social Risk OR Factors, Social Risk OR Risk Factor, Social OR Risk Factors, Social OR Social Risk Factor OR Health Correlates OR Correlates, Health OR Population at Risk OR Populations at Risk OR Risk Scores OR Risk Score OR Score, Risk OR Risk Factor Scores OR Risk Factor Score OR Score, Risk Factor)**** Filters: ****in the last 1 year**** | 123 |
| *Embase* | *1 ('Chronic Renal Insufficiencies' or 'Renal Insufficiencies, Chronic' or 'Chronic Renal Insufficiency' or 'Kidney Insufficiency, Chronic' or 'Chronic Kidney Insufficiency' or 'Chronic Kidney Insufficiencies' or 'Kidney Insufficiencies, Chronic' or 'Chronic Kidney Diseases' or 'Chronic Kidney Disease' or 'Disease, Chronic Kidney' or 'Diseases, Chronic Kidney' or 'Kidney Disease, Chronic' or 'Kidney Diseases, Chronic' or 'Chronic Renal Diseases' or 'Chronic Renal Disease' or 'Disease, Chronic Renal' or 'Diseases, Chronic Renal' or 'Renal Disease, Chronic' or 'Renal Diseases, Chronic').mp.*  *2 ('Sarcopenia' or 'Muscular Atrophy' or 'Sarcopenias' or 'muscle mass' or 'muscle strength' or 'Atrophies, Muscular' or 'Atrophy, Muscular' or 'Muscular Atrophies' or 'Atrophy, Muscle' or 'Atrophies, Muscle' or 'Muscle Atrophies' or 'Muscle Atrophy' or 'Neurogenic Muscular Atrophy' or 'Atrophies, Neurogenic Muscular' or 'Atrophy, Neurogenic Muscular' or 'Muscular Atrophies, Neurogenic' or 'Muscular Atrophy, Neurogenic' or 'Neurogenic Muscular Atrophies' or 'Neurotrophic Muscular Atrophy' or 'Atrophies, Neurotrophic Muscular' or 'Atrophy, Neurotrophic Muscular' or 'Muscular Atrophies, Neurotrophic' or 'Muscular Atrophy, Neurotrophic' or 'Neurotrophic Muscular Atrophies').mp.*  *3 ('Factor, Risk' or 'Risk Factor' or 'Social Risk Factors' or 'Factor, Social Risk' or 'Factors, Social Risk' or 'Risk Factor, Social' or 'Risk Factors, Social' or 'Social Risk Factor' or 'Health Correlates' or 'Correlates, Health' or 'Population at Risk' or 'Populations at Risk' or 'Risk Scores' or 'Risk Score' or 'Score, Risk' or 'Risk Factor Scores' or 'Risk Factor Score' or 'Score, Risk Factor' or 'risk factors').mp.* | *99* |
| *Web of science* | *[(((PY=(2024.7-2025.7)) AND TS=(Chronic Renal Insufficiencies OR Renal Insufficiencies, Chronic OR Chronic Renal Insufficiency OR Kidney Insufficiency, Chronic OR Chronic Kidney Insufficiency OR Chronic Kidney Insufficiencies OR Kidney Insufficiencies, Chronic OR Chronic Kidney Diseases OR Chronic Kidney Disease OR Disease, Chronic Kidney OR Diseases, Chronic Kidney OR Kidney Disease, Chronic OR Kidney Diseases, Chronic OR Chronic Renal Diseases OR Chronic Renal Disease OR Disease, Chronic Renal OR Diseases, Chronic Renal OR Renal Disease, Chronic OR Renal Diseases, Chronic)) AND TS=(sarcopeniaa OR muscle mass OR muscle strength OR Atrophies, Muscular OR Atrophy, Muscular OR Muscular Atrophies OR Atrophy, Muscle OR Atrophies, Muscle OR Muscle Atrophies OR Muscle Atrophy OR Neurogenic Muscular Atrophy OR Atrophies, Neurogenic Muscular OR Atrophy, Neurogenic Muscular OR Muscular Atrophies, Neurogenic OR Muscular Atrophy, Neurogenic OR Neurogenic Muscular Atrophies OR Neurotrophic Muscular Atrophy OR Atrophies, Neurotrophic Muscular OR Atrophy, Neurotrophic Muscular OR Muscular Atrophies, Neurotrophic OR Muscular Atrophy, Neurotrophic OR Neurotrophic Muscular Atrophies)) AND TS=(Factor, Risk OR Risk Factor OR Social Risk Factors OR Factor, Social Risk OR Factors, Social Risk OR Risk Factor, Social OR Risk Factors, Social OR Social Risk Factor OR Health Correlates OR Correlates, Health OR Population at Risk OR Populations at Risk OR Risk Scores OR Risk Score OR Score, Risk OR Risk Factor Scores OR Risk Factor Score OR Score, Risk Factor) and Preprint Citation Index (Exclude – Database)](https://res1.zcmu.edu.cn/https/vpn/3/P7TXE55GPNSXT3LPMNTT6Z5MMF3GT7UBPSTT6Z5P/wos/alldb/summary/4e0d441a-8ba1-491e-a8bd-5d2cd5945b61-016e040c40/relevance/1)* | *181* |
| *Wanfang* | *(主题:(慢性肾病 OR 慢性肾功能不全 OR 慢性肾脏疾病 OR 肾功能不全、慢性 ) and 主题:(肌肉减少症 OR 肌少症 OR 肌减少症) and 主题:(因素 OR 风险 OR 风险因素 OR 社会风险因素 OR 健康相关 OR 风险得分)) and 出版时间:[* TO 2025-05-21}* | *202* |
| *CNKI* | *（主题：慢性肾病＋慢性肾功能不全＋慢性肾脏疾病＋肾功能不全、慢性）A渡*  *ND（主题：肌肉減少症＋肌少症＋肌减少症）AND（主题：因素＋风险＋风险因素＋社会风险因素＋健康相关＋风险得分）AND（主题：慢性肾病＋慢性肾功能不全＋慢性肾脏疾病＋肾功能不全、慢性）AND（主题：肌肉減少症＋肌少症＋肌减少症）AND（主题：因素＋风险＋风险因素＋社会风险因素＋健康相关＋风险得分）* | *2* |
| *VIP* | *主题=肾功能不全, 慢性 AND 主题=肌肉减少症 AND 主题=影响因素分析* | *0* |
| *Sinomed* | *因素 OR 风险 OR 风险因素 OR 社会风险因素 OR 健康相关 OR 风险得分 OR 影响因素* | *5* |
| *Chinese Medical Association Journal Database* | *透析（全部字段） AND 肌少症（全部字段） AND 因素（全部字段）* | *0* |

*The search strategy used for this supplementary retrieval was identical to that employed in the initial search.*
