# Supplementary material for: Risk Factors Associated With Sarcopenia in Patients With Chronic Kidney Disease: A Systematic Review and Meta‐Analysis
Source: J Cachexia Sarcopenia Muscle. 2025 Dec 28;17(1):e70166. doi: 10.1002/jcsm.70166 (PMC12745342; doi:10.1002/jcsm.70166)
Supplement: Supplementary file 2 — Data S2: Supplementary Information. [file JCSM-17-e70166-s003.docx]

**Supporting material 2**

*Table S2 Quality assessment*

*Table S2.1 Risk of bias of the included studies was assessed using the Joanna Briggs Institute Critical Appraisal tool for cross-sectional studies.*

| **First author(year)** | **1.Were the criteria for inclusion in the sample clearly defined?** | **2.Were the study subjects and the setting described in detail?** | **3.Was the exposure measured in a valid and reliable way?** | **4.Were objective, standard criteria used for measurement of the condition?** | **5.Were confounding factors identified?** | **6.Were strategies to deal with confounding factors stated?** | **7.Were the outcomes measured in a valid and reliable way?** | **8.Was appropriate statistical analysis used?** | **Overall appraisal** |
| --- | --- | --- | --- | --- | --- | --- | --- | --- | --- |
| **Der-Sheng Han(2011)** | **yes** | **yes** | **yes** | **yes** | **yes** | **yes** | **yes** | **yes** | **A** |
| **Viviane O. Leal M.S.(2011)** | **yes** | **yes** | **yes** | **yes** | **unclear** | **unclear** | **unclear** | **yes** | **B** |
| **Nihal Ozkayar(2014)** | **yes** | **yes** | **yes** | **yes** | **yes** | **unclear** | **unclear** | **yes** | **B** |
| **Viviane Angelina de Souza(2017)** | **yes** | **yes** | **yes** | **yes** | **yes** | **unclear** | **yes** | **yes** | **B** |
| **M Yanishi(2017)** | **yes** | **yes** | **yes** | **yes** | **yes** | **unclear** | **yes** | **yes** | **B** |
| **Tufan A(2017)** | **yes** | **yes** | **yes** | **yes** | **yes** | **unclear** | **yes** | **yes** | **B** |
| **Atefeh As’habi(2018)** | **yes** | **yes** | **yes** | **yes** | **yes** | **yes** | **yes** | **yes** | **A** |
| **Claudia D’Alessandro(2018)** | **yes** | **yes** | **yes** | **yes** | **yes** | **yes** | **yes** | **yes** | **A** |
| **Seiko Ishikawa(2018)** | **yes** | **yes** | **yes** | **yes** | **yes** | **yes** | **yes** | **yes** | **A** |
| **S Yoowannakul(2018)** | **yes** | **yes** | **yes** | **yes** | **yes** | **yes** | **yes** | **yes** | **A** |
| **Bruna Guida(2019)** | **yes** | **yes** | **yes** | **yes** | **yes** | **yes** | **yes** | **yes** | **A** |
| **Carlos Quiñónez-Olivas(2019)** | **yes** | **yes** | **yes** | **yes** | **yes** | **yes** | **yes** | **yes** | **A** |
| **Shen Yiwei(2019)** | **yes** | **yes** | **yes** | **yes** | **yes** | **unclear** | **yes** | **yes** | **B** |
| **Elane Viana Furtado Hortegal(2020)** | **yes** | **yes** | **yes** | **yes** | **yes** | **unclear** | **yes** | **yes** | **B** |
| **Qian Zhang(2020)** | **yes** | **yes** | **yes** | **yes** | **yes** | **yes** | **yes** | **yes** | **A** |
| **Akira Saito(2020)** | **yes** | **yes** | **yes** | **yes** | **unclear** | **unclear** | **yes** | **yes** | **B** |
| **Song Liangchen(2020)** | **yes** | **yes** | **yes** | **yes** | **yes** | **yes** | **yes** | **yes** | **A** |
| **Seok Hui Kang(2020)** | **yes** | **yes** | **yes** | **yes** | **yes** | **yes** | **yes** | **yes** | **A** |
| **Zhu Beixia(2020)** | **yes** | **yes** | **yes** | **yes** | **unclear** | **unclear** | **yes** | **yes** | **B** |
| **Ruben Abdala(2021)** | **yes** | **yes** | **yes** | **yes** | **unclear** | **unclear** | **yes** | **yes** | **B** |
| **Maria Mattera(2021)** | **yes** | **yes** | **yes** | **yes** | **yes** | **unclear** | **yes** | **yes** | **B** |
| **Marille Umakanthan(2021)** | **yes** | **yes** | **yes** | **yes** | **yes** | **yes** | **yes** | **yes** | **A** |
| **Bang-Gee Hsu(2021)** | **yes** | **yes** | **yes** | **yes** | **yes** | **yes** | **yes** | **yes** | **A** |
| **Du Wen(2021)** | **yes** | **yes** | **yes** | **yes** | **unclear** | **unclear** | **yes** | **yes** | **B** |
| **de Amorim Geraldo José(2022)** | **yes** | **yes** | **yes** | **yes** | **yes** | **unclear** | **yes** | **yes** | **B** |
| **Jéssica Santos Xavier(2022)** | **yes** | **yes** | **yes** | **yes** | **yes** | **unclear** | **unclear** | **yes** | **B** |
| **Rafael Moreno-González(2023)** | **yes** | **yes** | **yes** | **yes** | **yes** | **yes** | **yes** | **yes** | **A** |
| **Jianming Zhang(2023)** | **yes** | **yes** | **yes** | **yes** | **yes** | **yes** | **yes** | **yes** | **A** |
| **Li Hong(2023)** | **yes** | **yes** | **yes** | **yes** | **unclear** | **unclear** | **yes** | **yes** | **B** |
| **Seyda Gul Ozcan(2024)** | **yes** | **yes** | **yes** | **yes** | **unclear** | **unclear** | **yes** | **yes** | **B** |
| **Gloria Kojo(2014)** | **yes** | **yes** | **yes** | **yes** | **yes** | **yes** | **yes** | **yes** | **A** |
| **Álvarez(2024)** | **yes** | **yes** | **yes** | **yes** | **yes** | **yes** | **yes** | **yes** | **A** |
| **[Hsu(2024)](https://pubmed.ncbi.nlm.nih.gov/?term=)** | **yes** | **yes** | **yes** | **yes** | **yes** | **yes** | **yes** | **yes** | **A** |
| **Hu(2024)** | **yes** | **yes** | **unclear** | **yes** | **yes** | **yes** | **yes** | **yes** | **B** |
| **Huang(2024)** | **yes** | **yes** | **yes** | **yes** | **yes** | **yes** | **yes** | **yes** | **A** |
| **Qaisar(2024)** | **yes** | **yes** | **yes** | **yes** | **yes** | **yes** | **yes** | **yes** | **A** |
| **Wu(2024)** | **yes** | **yes** | **yes** | **yes** | **yes** | **yes** | **yes** | **yes** | **A** |
| **Zeng(2024)** | **yes** | **yes** | **unclear** | **yes** | **yes** | **unclear** | **yes** | **yes** | **B** |
| **Zhao(2024)** | **yes** | **yes** | **yes** | **yes** | **yes** | **yes** | **yes** | **yes** | **A** |
| **M(2025)** | **yes** | **yes** | **yes** | **yes** | **yes** | **yes** | **yes** | **yes** | **A** |
| **Mansouri(2025)** | **yes** | **yes** | **yes** | **yes** | **yes** | **yes** | **yes** | **yes** | **A** |

*Each of item can be assessed as “yes”, “no”, “unclear”, and “not applicable” . The quality level of the research that is satisfactory for all items is A, and the quality level of the research that is satisfactory for some items is B (items 1-3 are "not")*

*Table S2.2 Quality assessment of the case-control studies was performed using the Newcastle-Ottawa Scale (NOS) Quality Assessment Tool.*

| **First author(year)** | **Study population selection** | | | | **Comparability between groups** | **Measurement of exposure factors** | | **Overall appraisal** |
| --- | --- | --- | --- | --- | --- | --- | --- | --- |
|  |  |  |  |  |  |  |  |  |
|  | **Is the Case Definition Adequate?** | **Representativeness of the Cases(1 point)** | **Selection of Controls(1 point)** | **Definition of Controls(1 point）** | **Comparability of Cases and Controls on the Basis of the Design or Analysis(2 points)** | **Ascertainment of Exposure(1 point)** | **Non-Response Rate(1 point)** |  |
| **Wang Qian(2023)** | **1** | **1** | **1** | **0** | **2** | **1** | **1** | **7** |
| **Yiqi Song(2022)** | **1** | **1** | **1** | **1** | **2** | **1** | **0** | **7** |
| **Saliha Yildirim(2022)** | **1** | **1** | **1** | **1** | **2** | **1** | **0** | **7** |
| **Mayuko Hori(2024)** | **1** | **1** | **1** | **1** | **2** | **1** | **0** | **7** |

*A perfect score is 9 points; 0 to 4 indicates a low-quality study, and 5 to 9 indicates a high-quality study.*

*Table S2.3 Quality assessment of the cohort studies was performed using the Newcastle-Ottawa Scale (NOS) Quality Assessment Tool.*

| **First author(year)** | **Study population selection** | | | | **Comparability between groups** | **Measurement of exposure factors** | | | **Overall appraisal** |
| --- | --- | --- | --- | --- | --- | --- | --- | --- | --- |
|  |  |  |  |  |  |  |  |  |  |
|  | **Representativeness of the Exposed Cohort(1 point)** | **Selection of the Non-Exposed Cohort(1 point)** | **Ascertainment of Exposure(1 point)** | **Demonstration That Outcome of Interest Was Not Present at Start of Study(1 point)** | **Comparability of Cohorts on the Basis of the Design or Analysis(2 points)** | **Assessment of Outcome(1 point)** | **Was Follow-Up Long Enough for Outcomes to Occur(1 point)** | **Adequacy of Follow Up of Cohorts(1 point)** |  |
| **Jung Nam An(2022)** | **1** | **1** | **1** | **1** | **2** | **1** | **0** | **0** | **7** |
| **Wesley J. Visser(2020)** | **1** | **1** | **1** | **1** | **2** | **1** | **1** | **1** | **9** |
| **Ming-Dian Yu(2021)** | **1** | **1** | **1** | **1** | **2** | **1** | **0** | **0** | **7** |
| **Jung Nam An(2021)** | **1** | **1** | **1** | **1** | **2** | **1** | **0** | **0** | **7** |
| **Zhang Qi(2020)** | **1** | **1** | **1** | **1** | **2** | **1** | **0** | **0** | **7** |
| **Chen Xuan(2024)** | **1** | **1** | **1** | **1** | **2** | **1** | **0** | **0** | **7** |
| **Alirezaei(2024)** | **1** | **1** | **1** | **1** | **2** | **1** | **1** | **0** | **8** |
| **Ben-Noach(2024)** | **1** | **1** | **1** | **1** | **2** | **0** | **1** | **0** | **7** |
| **Miyasato(2024)** | **1** | **1** | **1** | **1** | **1** | **1** | **1** | **1** | **8** |
| **Nishihira(2024)** | **1** | **1** | **1** | **0** | **2** | **1** | **0** | **1** | **7** |
| **Chang(2025)** | **1** | **1** | **0** | **1** | **2** | **1** | **1** | **1** | **8** |

*A perfect score is 9 points; 0 to 4 indicates a low-quality study, and 5 to 9 indicates a high-quality study.*

*Table S2.4 The quality of the randomized controlled trials was evaluated using the Cochrane Handbook for Systematic Reviews of Interventions.*

| **First author(year)** | **Selection bias** | | **Performane bias** | **Attrition bias** | **Reporting bias** | **Other bias** | **Overall appraisal** |
| --- | --- | --- | --- | --- | --- | --- | --- |
|  | **Bias arising from the randomization process** | **Bias due to deviations from intended interventions** |  |  |  |  |  |
| **Nie Lei(2023)** | **Low risk of bias** | **Unclear** | **Unclear** | **Low risk of bias** | **Low risk of bias** | **Low risk of bias** | **Probably low risk of bias** |
| **Avinash Kumar Dubey(2021)** | **Low risk of bias** | **Unclear** | **Unclear** | **Low risk of bias** | **Low risk of bias** | **Low risk of bias** | **Probably low risk of bias** |
