# Supplementary material for: Risk Factors Associated With Sarcopenia in Patients With Chronic Kidney Disease: A Systematic Review and Meta‐Analysis
Source: J Cachexia Sarcopenia Muscle. 2025 Dec 28;17(1):e70166. doi: 10.1002/jcsm.70166 (PMC12745342; doi:10.1002/jcsm.70166)
Supplement: Supplementary file 3 — Data S3: Supplementary Information. [file JCSM-17-e70166-s002.docx]

**Supporting material 3**

*Age*

Figure S 1 Sensitivity Analysis of the Association Between Age and Sarcopenia in Patients with CKD

*
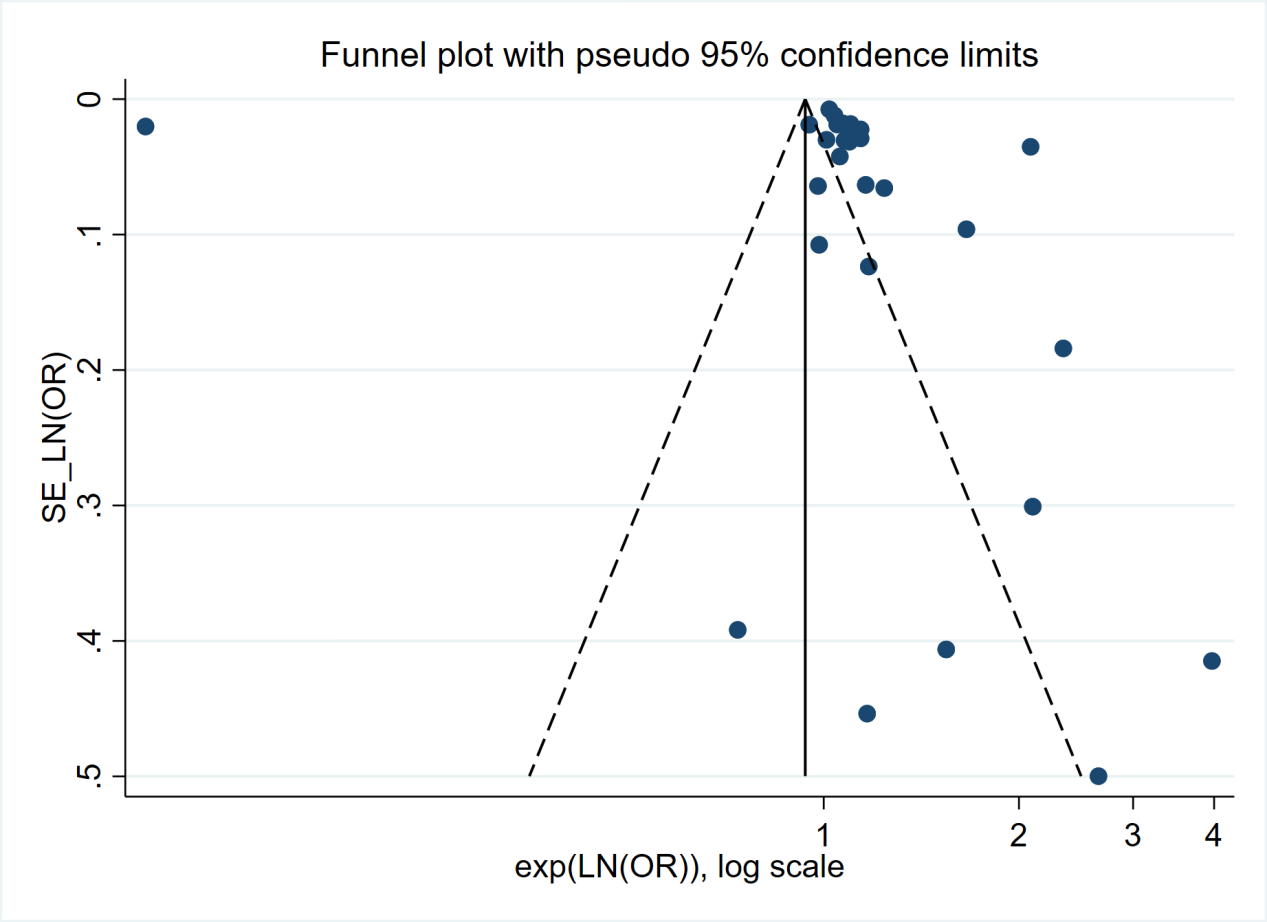
*

Figure S 2Funnel Plot of the Association Between Age and Sarcopenia in Patients with Chronic Kidney Disease

*
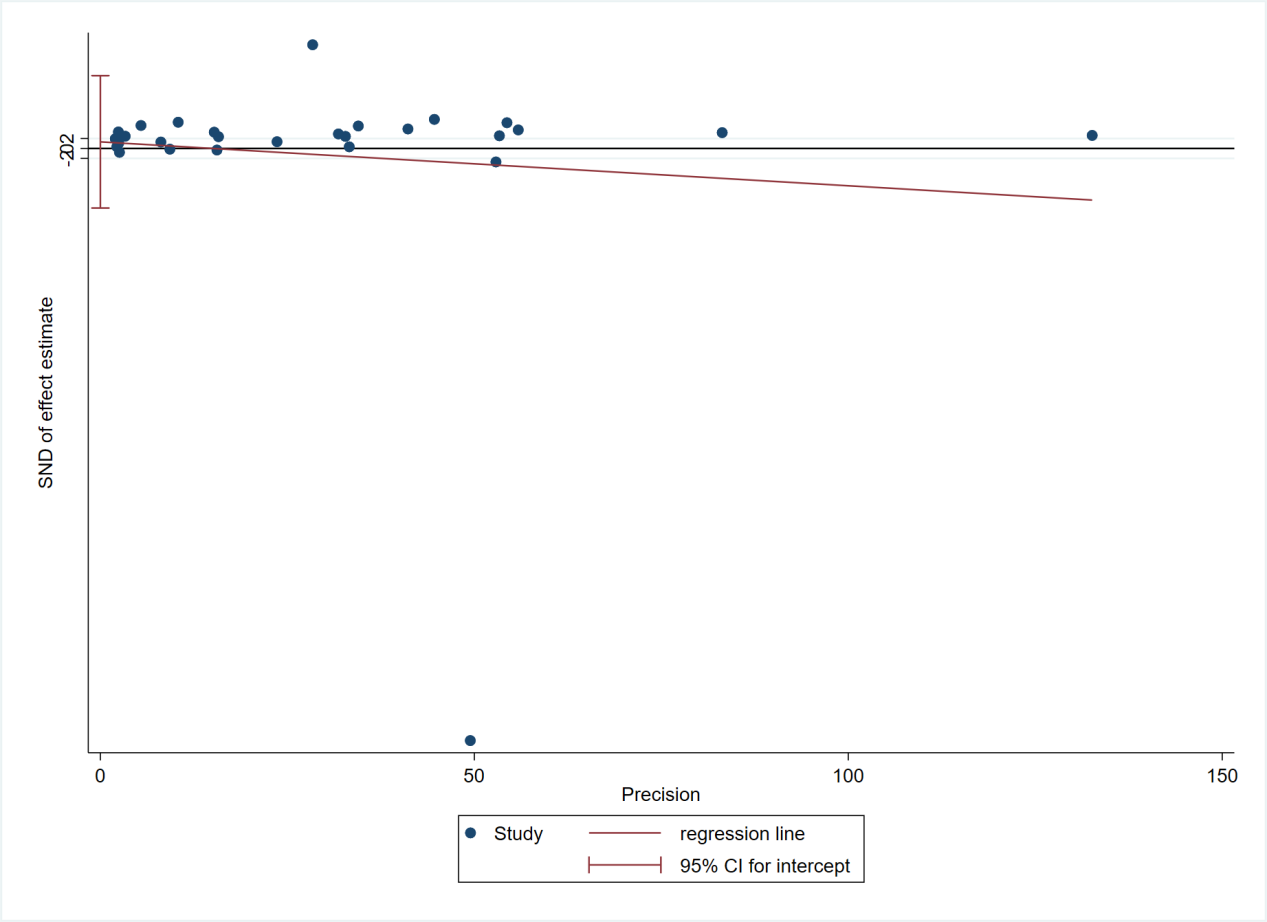
*

Figure S 3Egger's Test of the Association Between Age and Sarcopenia in Patients with CKD

Figure S 4Meta-Regression Analysis of the Association Between Age and Sarcopenia in Patients with CKD

*
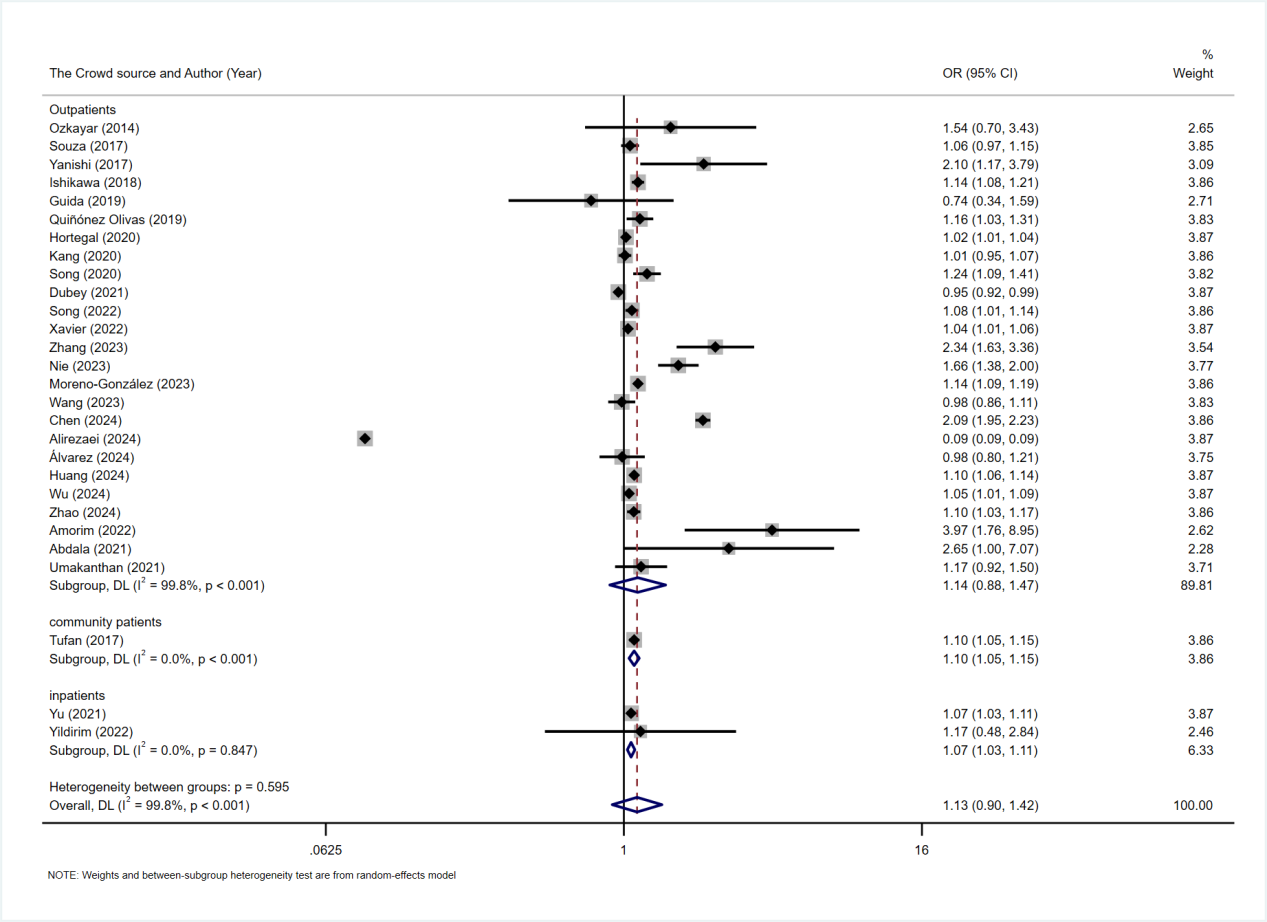
*

Figure S 5Forest Plot of Subgroup Analysis by the Crowds Source for the Association between Age and Sarcopenia in Patients with CKD

*
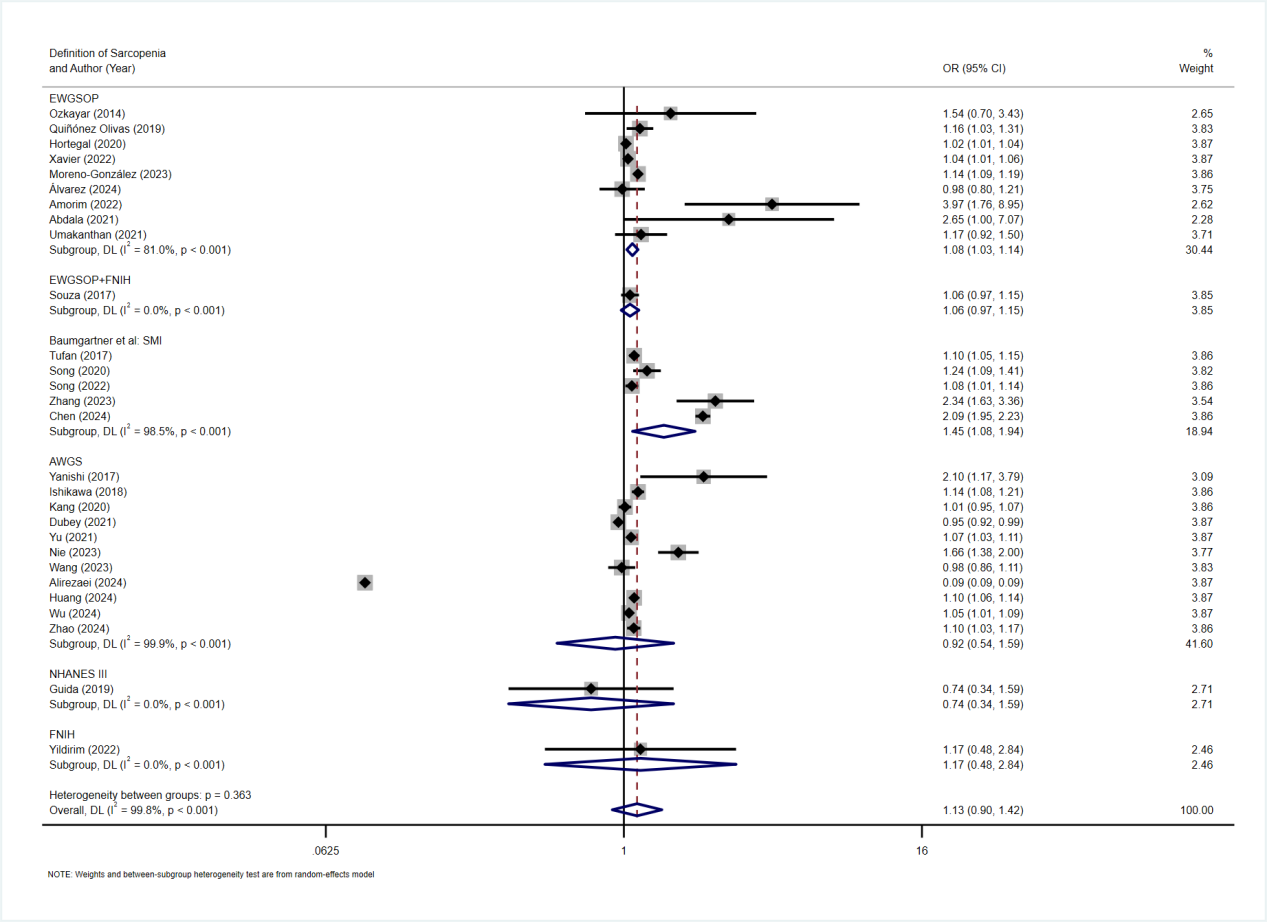
*

Figure S 6Forest Plot of Subgroup Analysis by Muscle Mass Assessment Method for the Association between Age and Sarcopenia in Patients with CKD

*
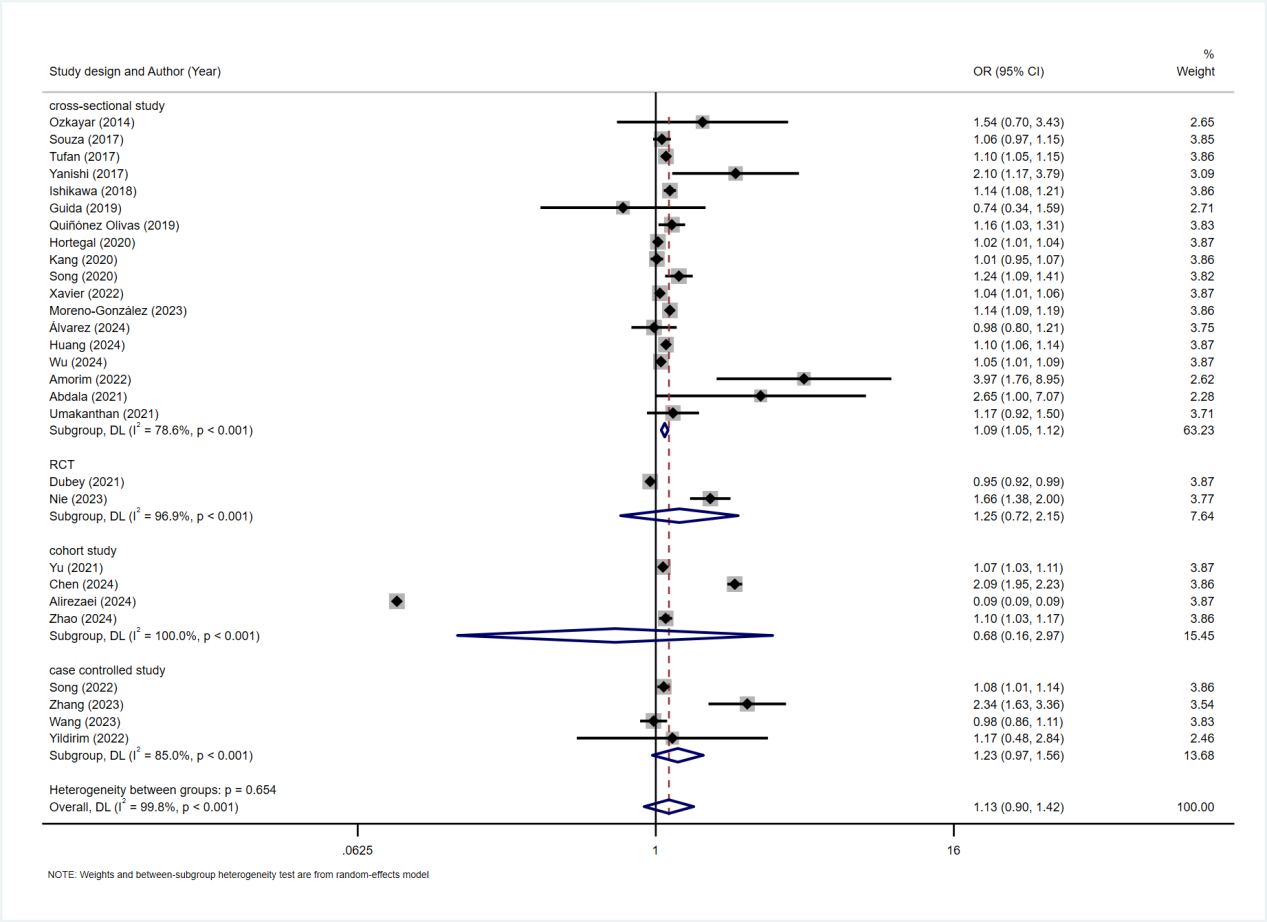
*

Figure S 7Forest Plot of Subgroup Analysis by Study Design for the Association between Age and Sarcopenia in Patients with CKD

*Figure 1 Forest Plot of Subgroup Analysis by Study Design for the Association between Age and Sarcopenia in Patients with CKD*

*
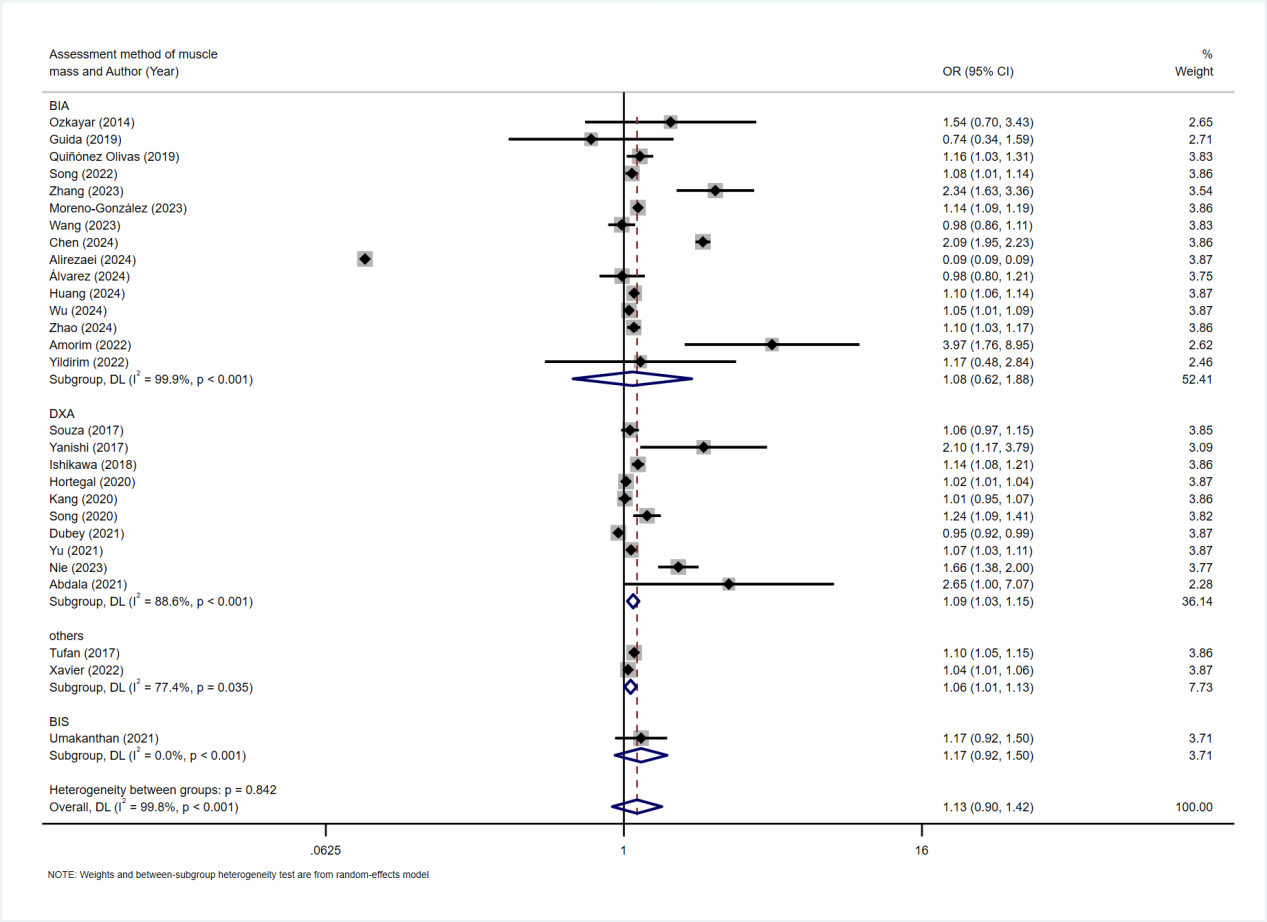
*

Figure S 8Forest Plot of Subgroup Analysis by Muscle Mass Assessment Method for the Association between Age and Sarcopenia in Patients with CKD

*
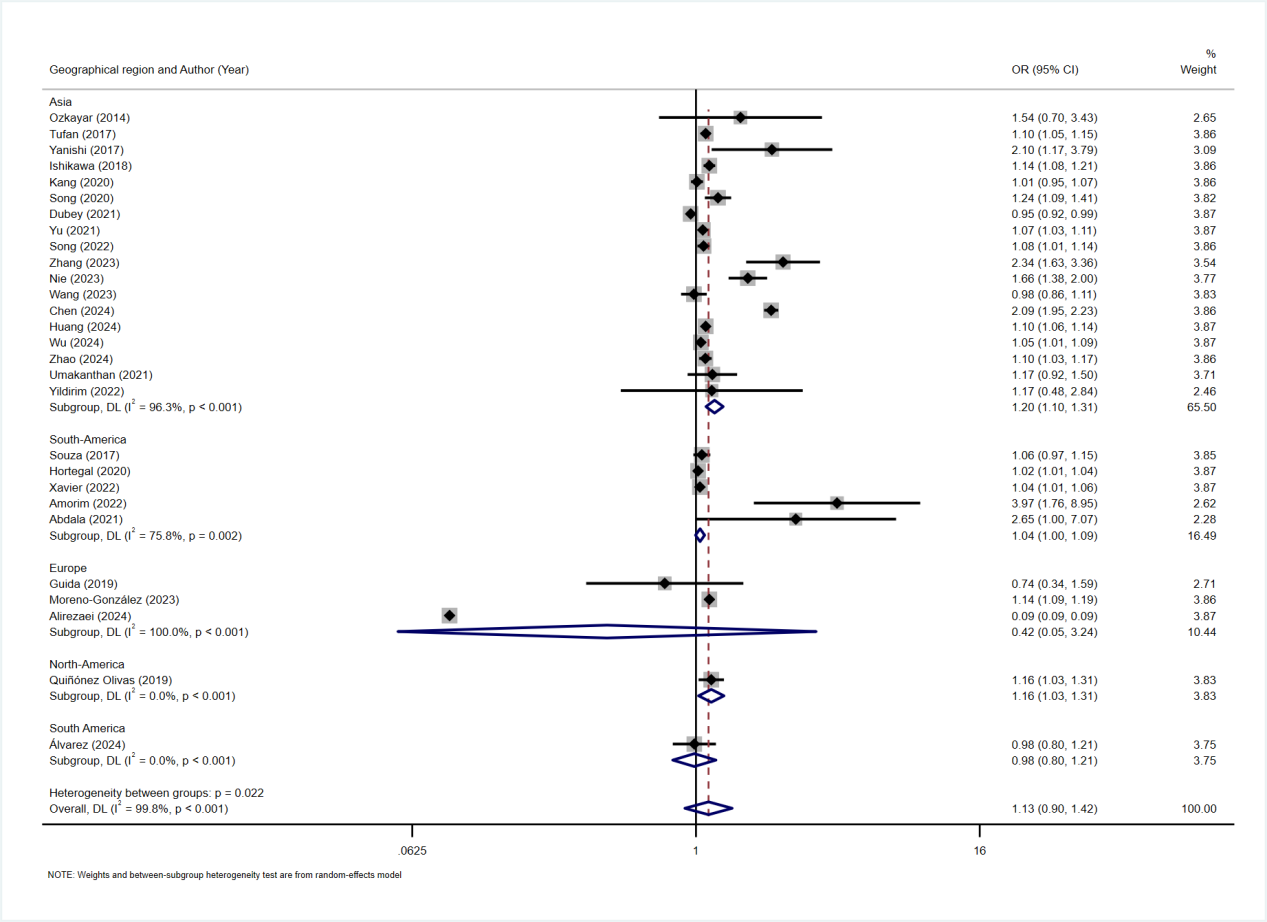
*

Figure S 9Forest Plot of Subgroup Analysis by Geographic Region for the Association between Age and Sarcopenia in Patients with CKD

*Alcohol consumption history*

**

Figure S 10Sensitivity Analysis of the Association between Alcohol Consumption History and Sarcopenia in Patients with CKD

*
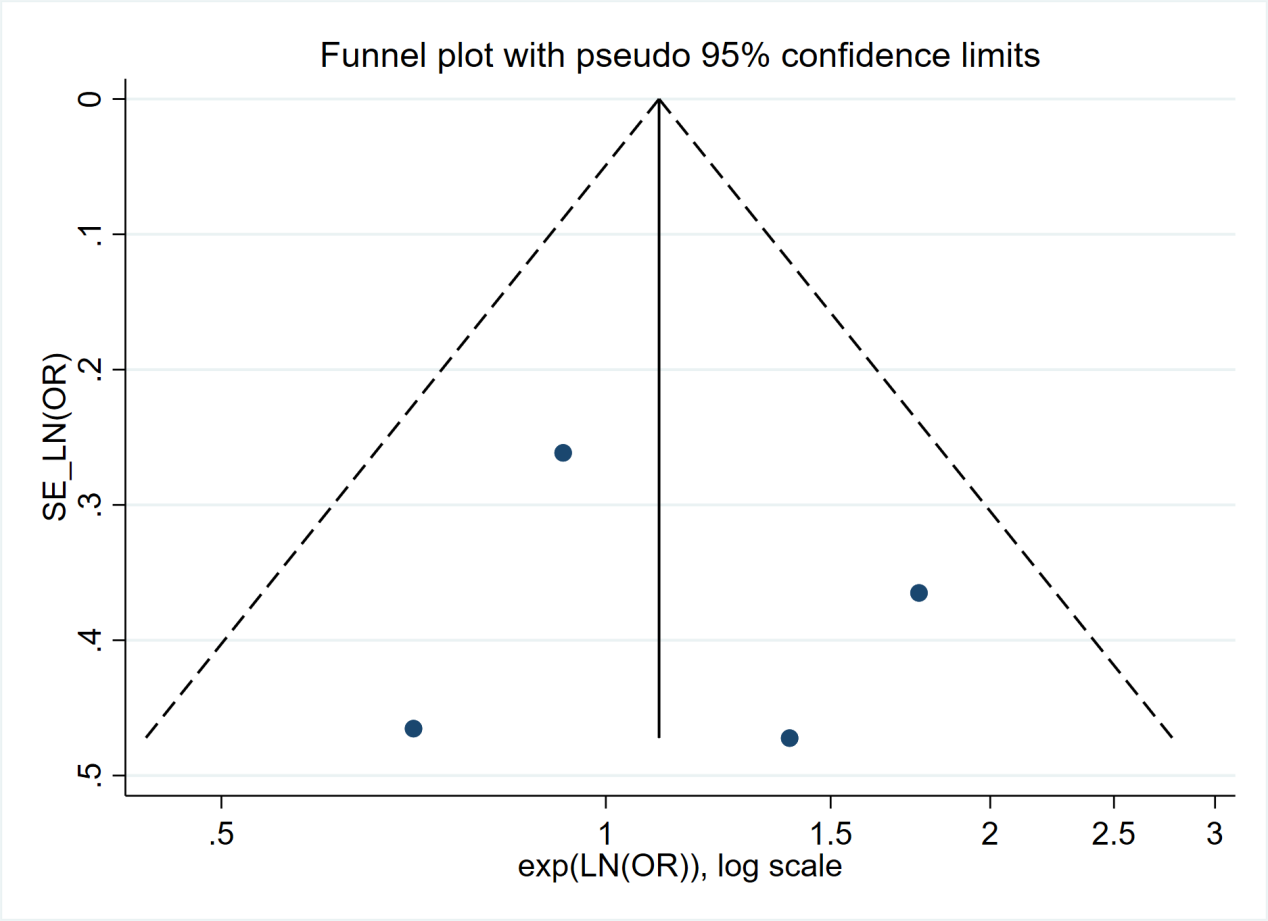
*

Figure S 11Funnel Plot for Publication Bias of the Association between Alcohol Consumption History and Sarcopenia in Patients with CKD

*
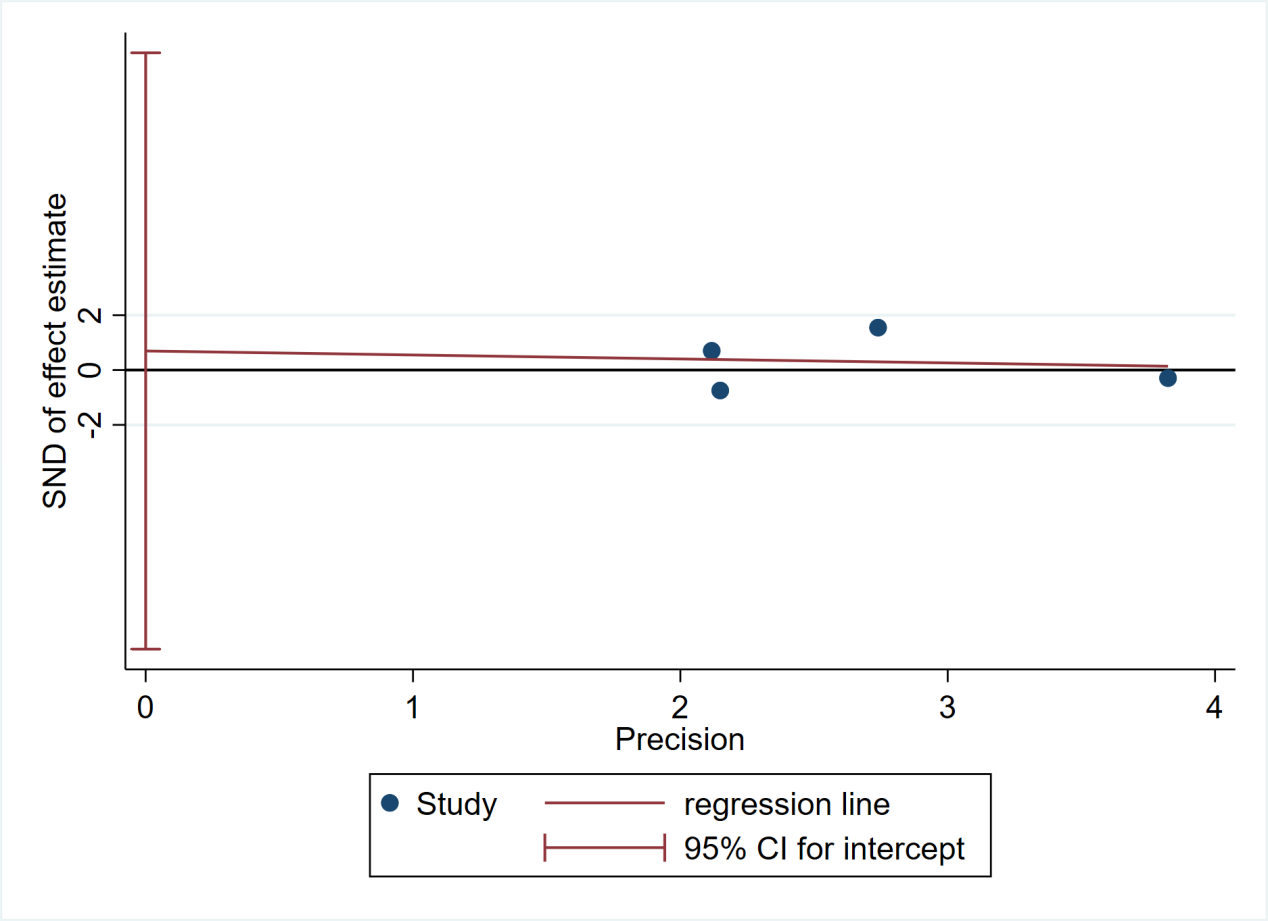
*

**

Figure S 12Egger's Test for Publication Bias of the Association between Alcohol Consumption History and Sarcopenia in Patients with CKD

*Antidiabetic drugs*

**

Figure S 13 Sensitivity Analysis of the Association between Antidiabetic Drugs and Sarcopenia in Patients with CKD

*
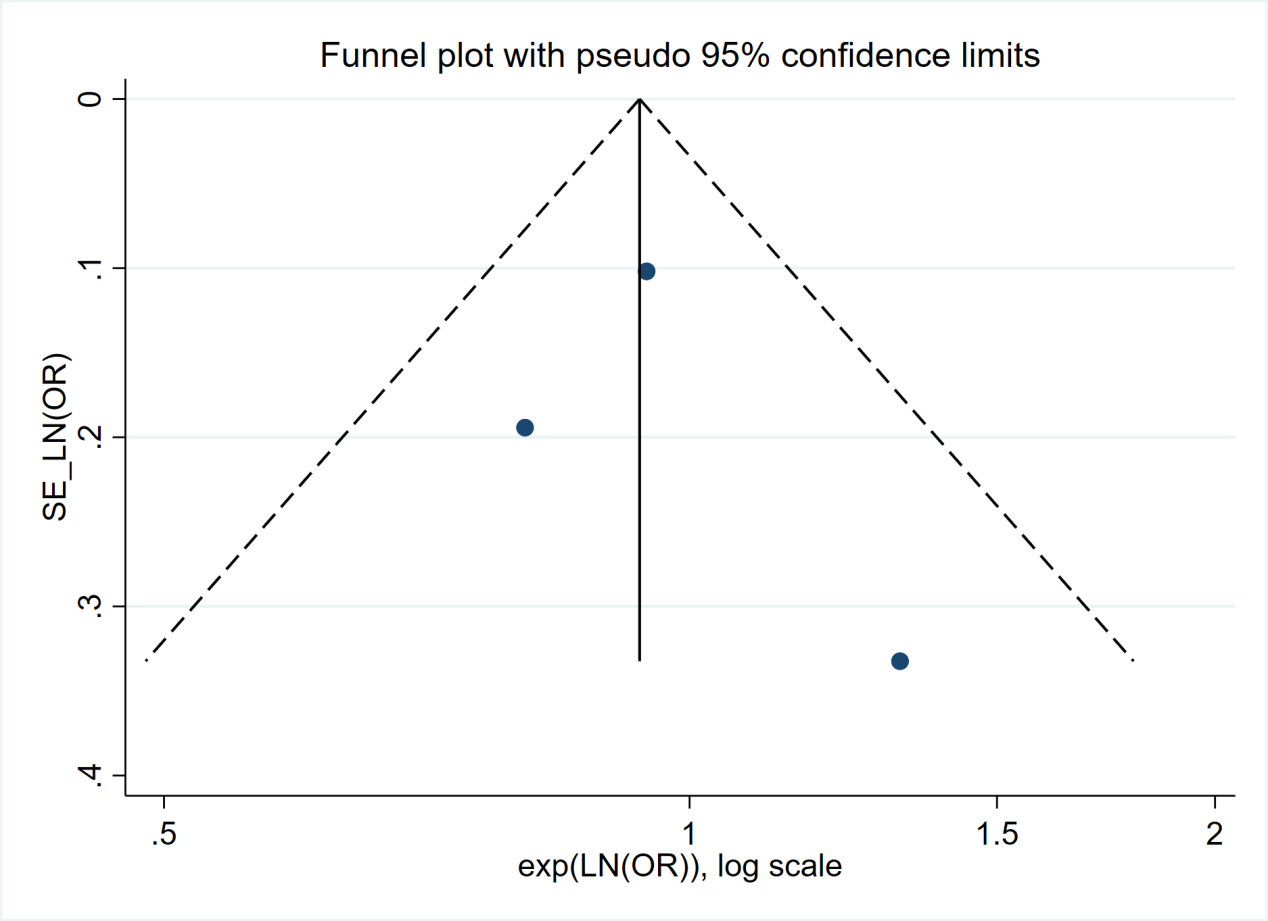
*

Figure S 14Funnel Plot for Publication Bias of the Association between Antidiabetic Drugs and Sarcopenia in Patients with CKD

*
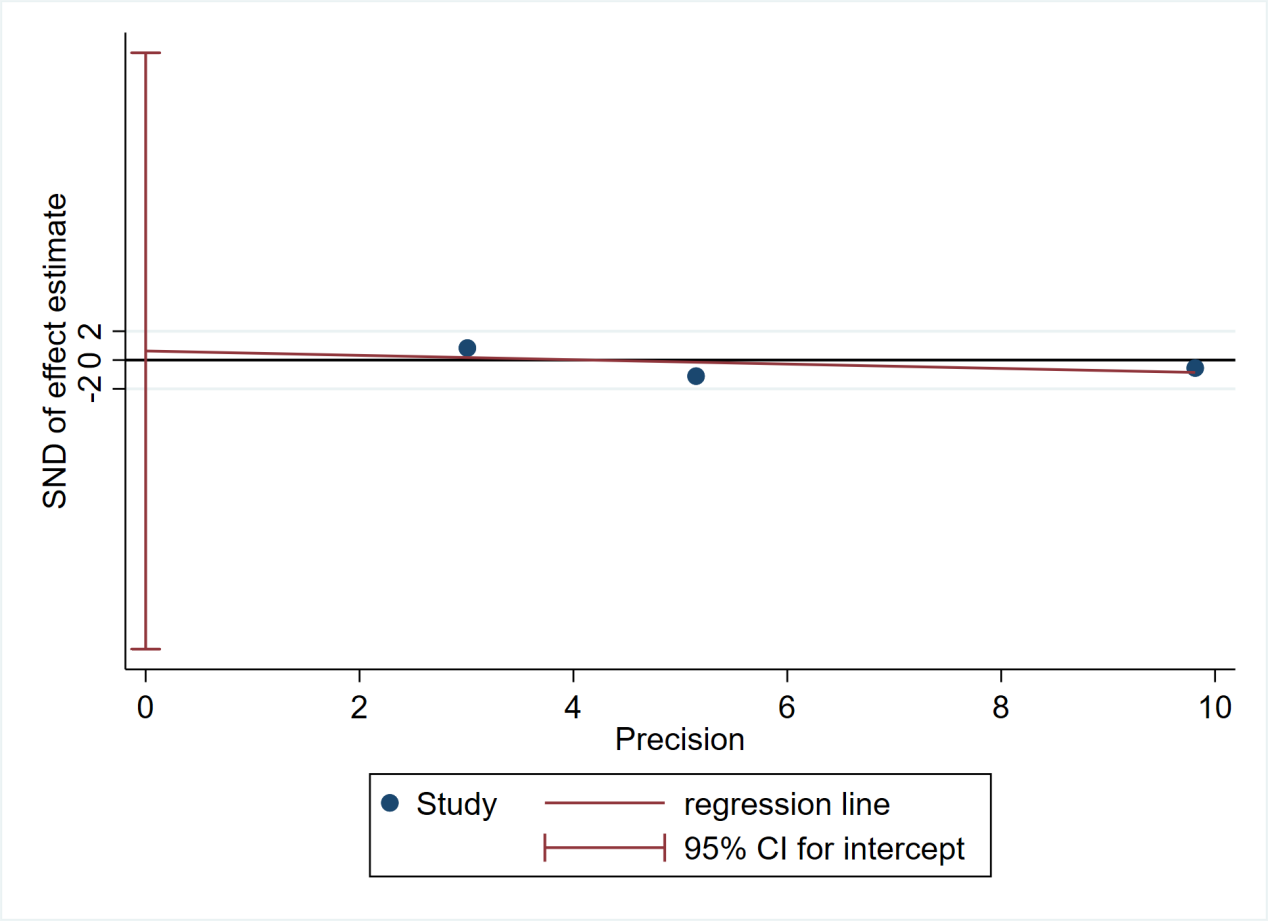
*

**

Figure S 15Egger's Test for Publication Bias of the Association between Antidiabetic Drugs and Sarcopenia in Patients with CKD

*Anti-hypertensive drug*

**

Figure S 16Sensitivity Analysis of the Association between Anti-hypertensive Drugs and Sarcopenia in Patients with CKD

*
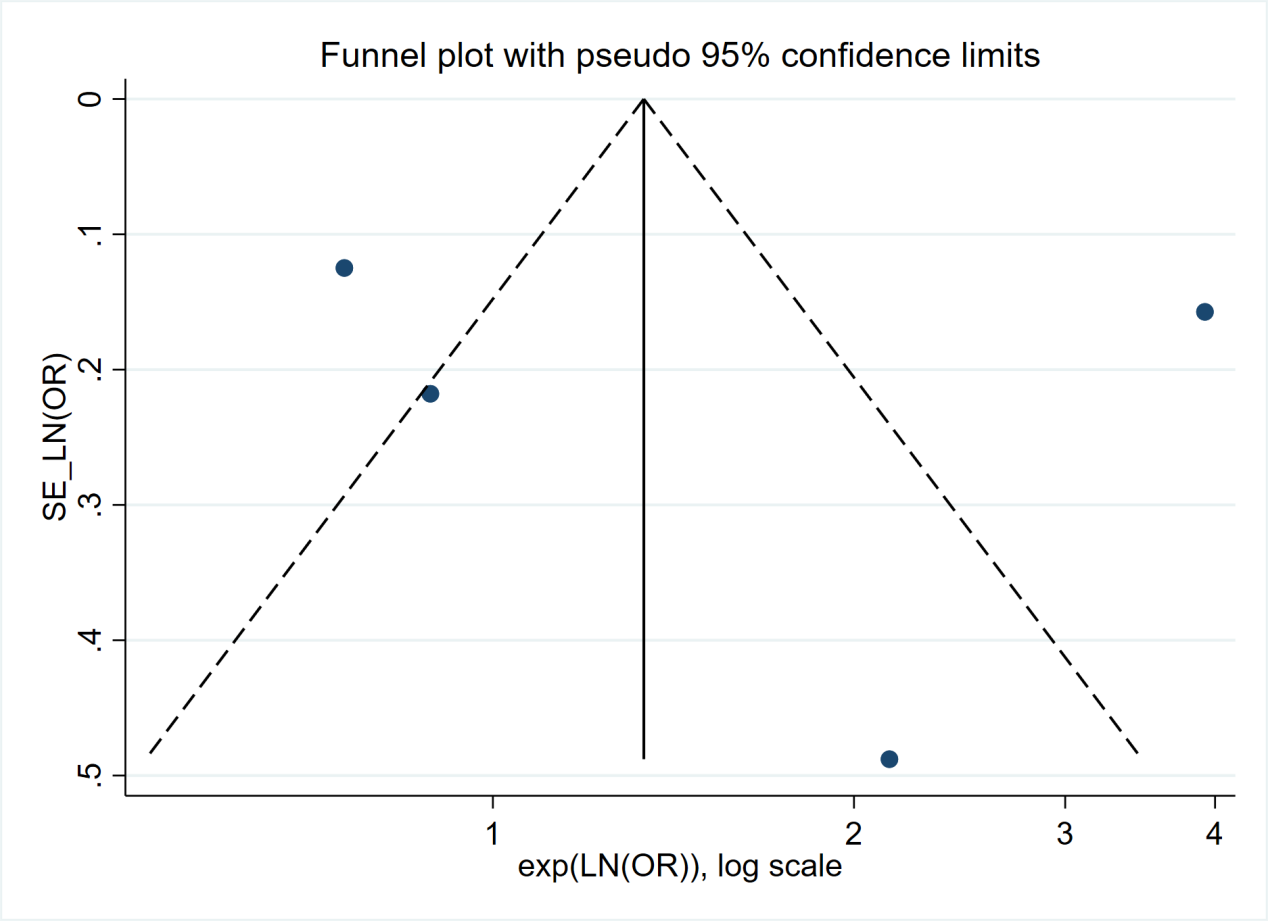
*

Figure S 17Funnel Plot for Publication Bias of the Association between Anti-hypertensive Drugs and Sarcopenia in Patients with CKD

*
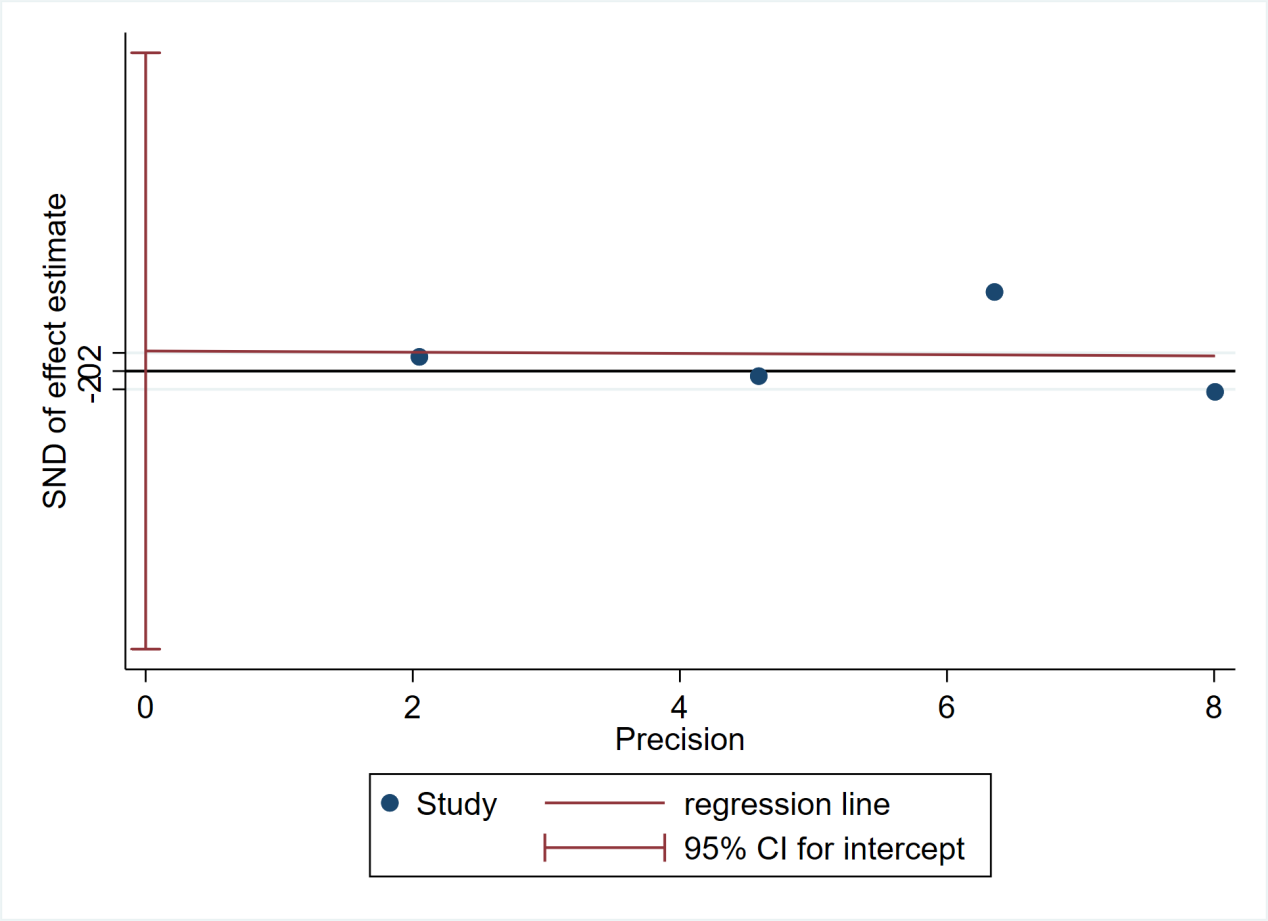
*

**

Figure S 18Egger's Test for Publication Bias of the Association between Anti-hypertensive Drugs and Sarcopenia in Patients with CKD

*Results After Excluding Studies with High Heterogeneity in Sensitivity Analysis*

*In this Meta-analysis, the initial analysis showed high overall heterogeneity (I² = 95.9% > 50%, P = 0.000 < 0.1). Through sensitivity analysis, we identified that the study by Ishikawa (2018) significantly contributed to this heterogeneity. The unique feature of this study is its focus on the impact of loop diuretics on sarcopenia, which differs from other types of diuretics studied. This variation in drug type may have caused the heterogeneity. After excluding this study, the heterogeneity significantly decreased (I² = 55.4% < 50%, P = 0.582 > 0.1), and the pooled effect size became more robust. This suggests that the difference in drug types might be the primary source of heterogeneity. Ultimately, we chose to retain this study and fully discussed the impact of this decision on the conclusions in the report.*

*
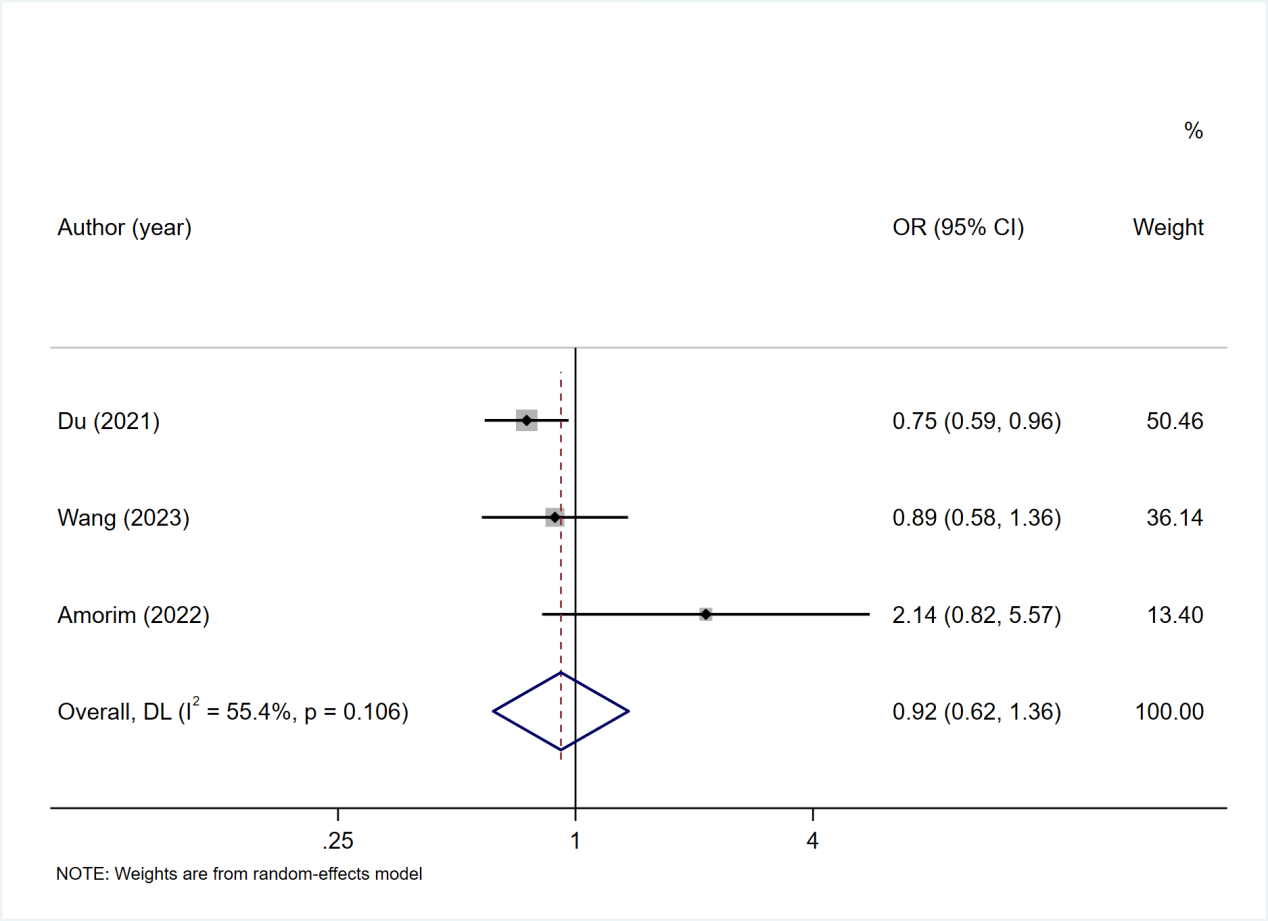
*

Figure S 19Forest Plot for the Association between Anti-hypertensive Drugs and Sarcopenia in Patients with CKD (Excluding Ishikawa 2018)

**

Figure S 20Sensitivity Analysis for the Association between Anti-hypertensive Drugs and Sarcopenia in Patients with CKD (Excluding Ishikawa 2018)

*
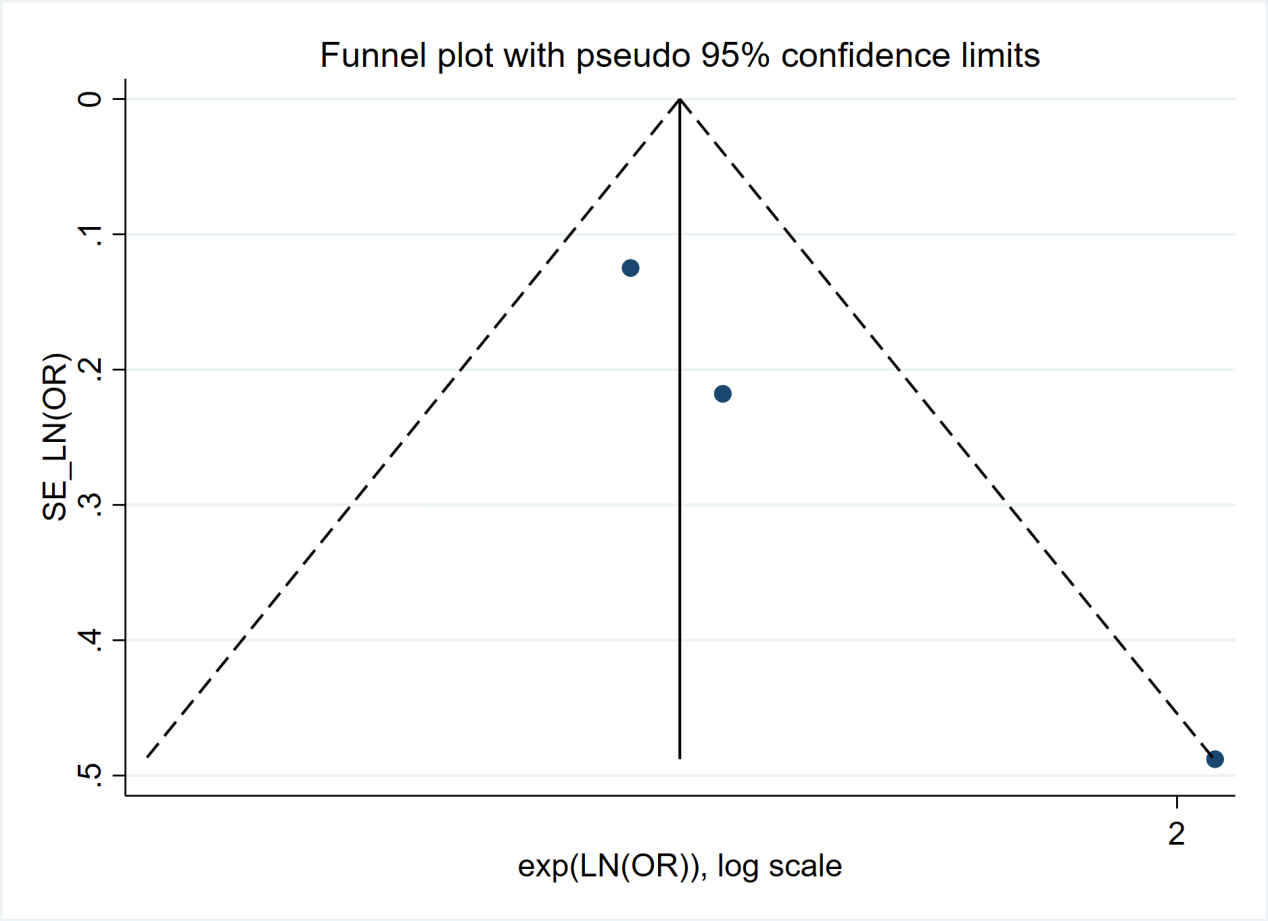
*

Figure S 21Funnel Plot for Publication Bias of the Association between Anti-hypertensive Drugs and Sarcopenia in Patients with CKD (Excluding Ishikawa 2018)

*
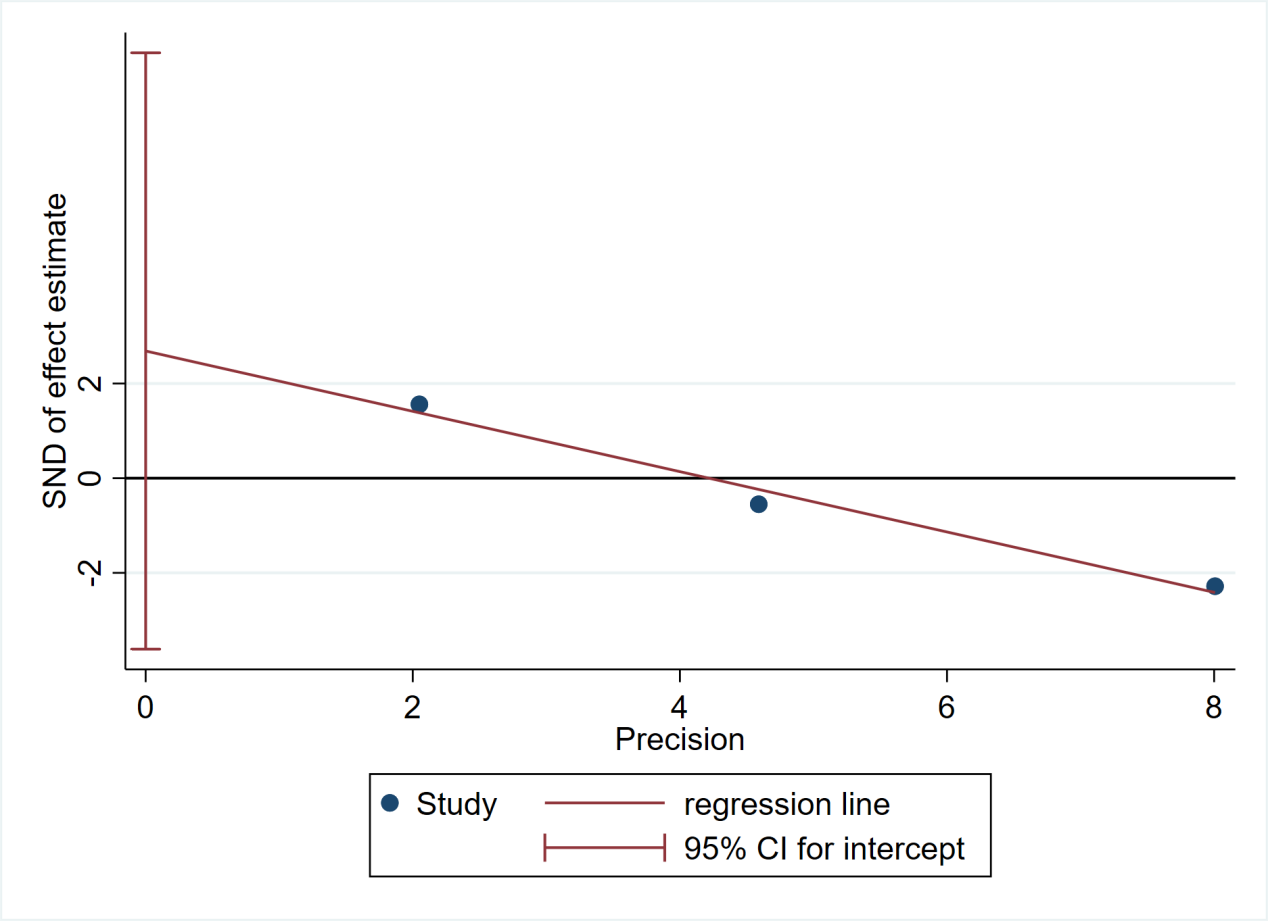
*

**

Figure S 22Egger's Test for Publication Bias of the Association between Anti-hypertensive Drugs and Sarcopenia in Patients with CKD (Excluding Ishikawa 2018)

*BMI(Body Mass Index)*

Figure S 23Sensitivity Analysis of the Association Between BMI and Sarcopenia in Patients with CKD

*
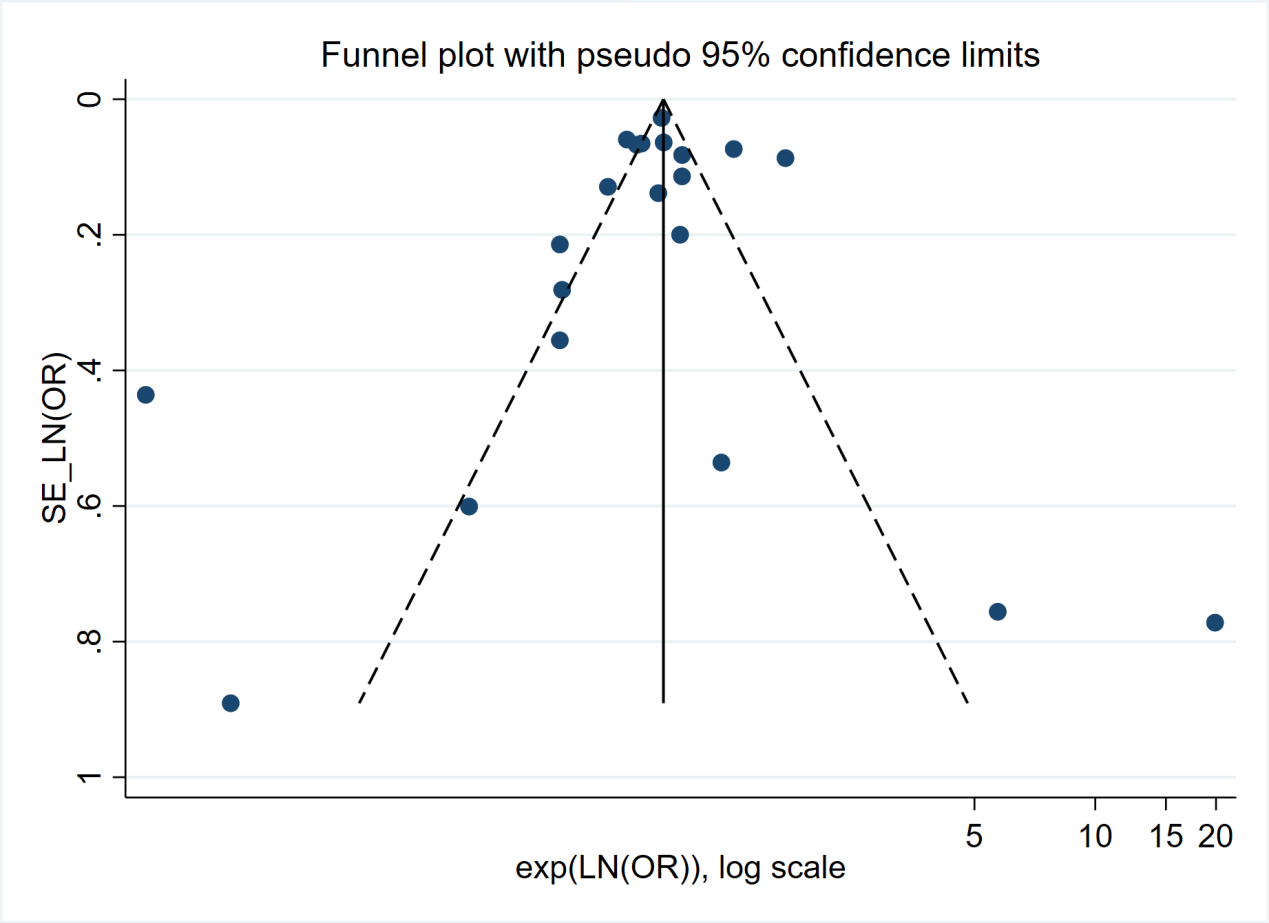
*

Figure S 24Funnel Plot of the Association Between BMI and Sarcopenia in Patients with Chronic Kidney Disease

*
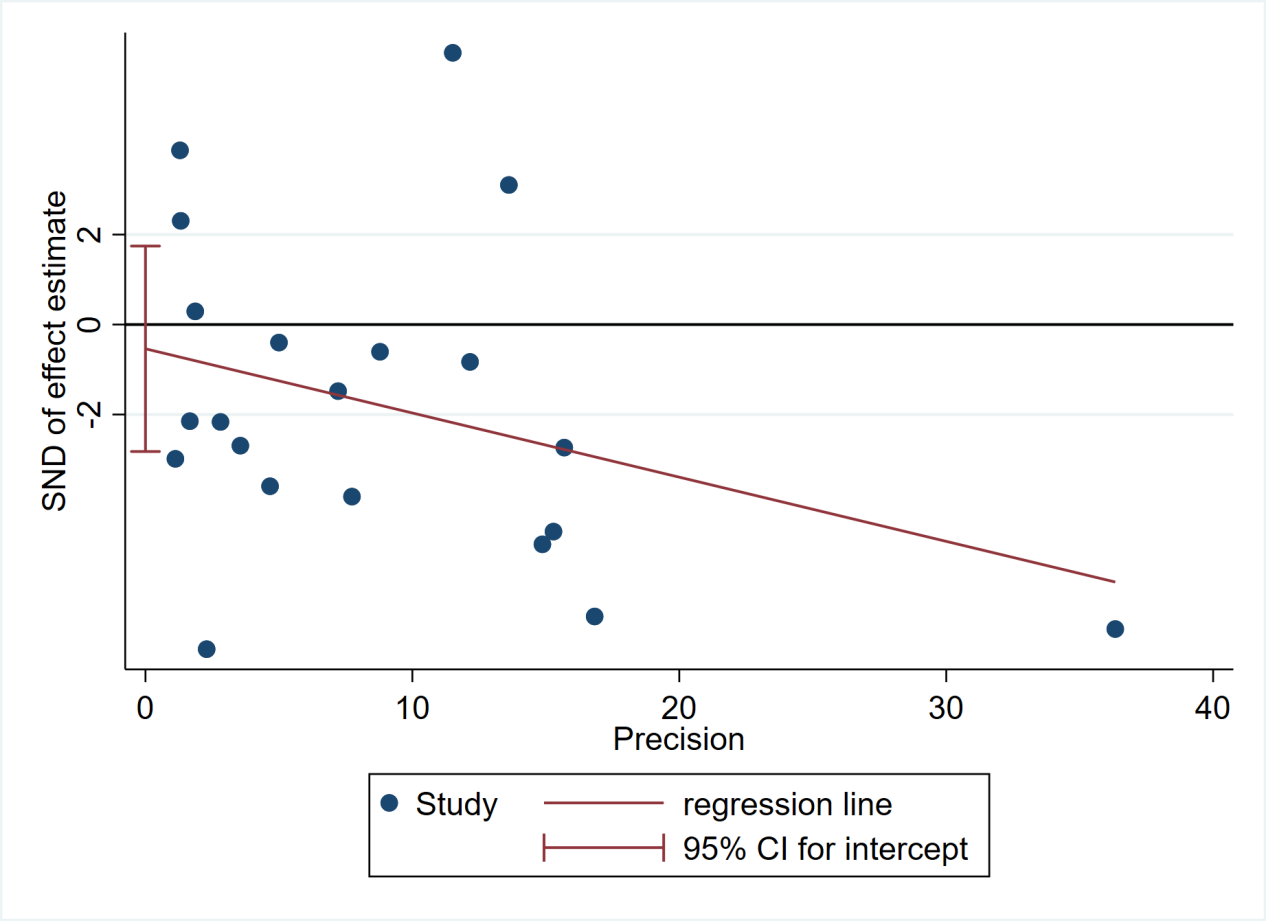
*

Figure S 25Egger's Test of the Association Between BMI and Sarcopenia in Patients with CKD

*
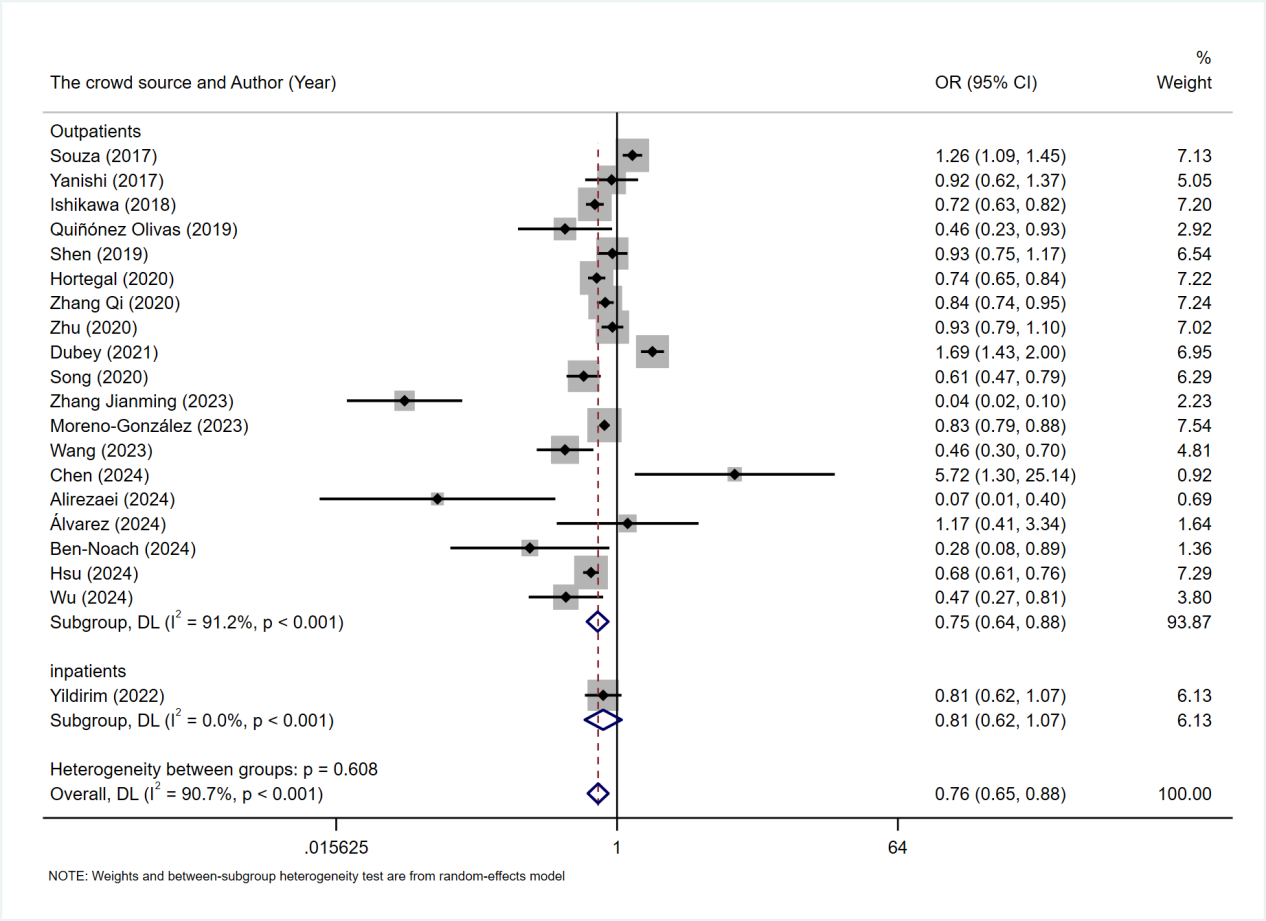
*

Figure S 26Forest Plot of the Subgroup Analysis of the Association Between BMI and Sarcopenia in Patients with CKD Based on the Crowds Source

*
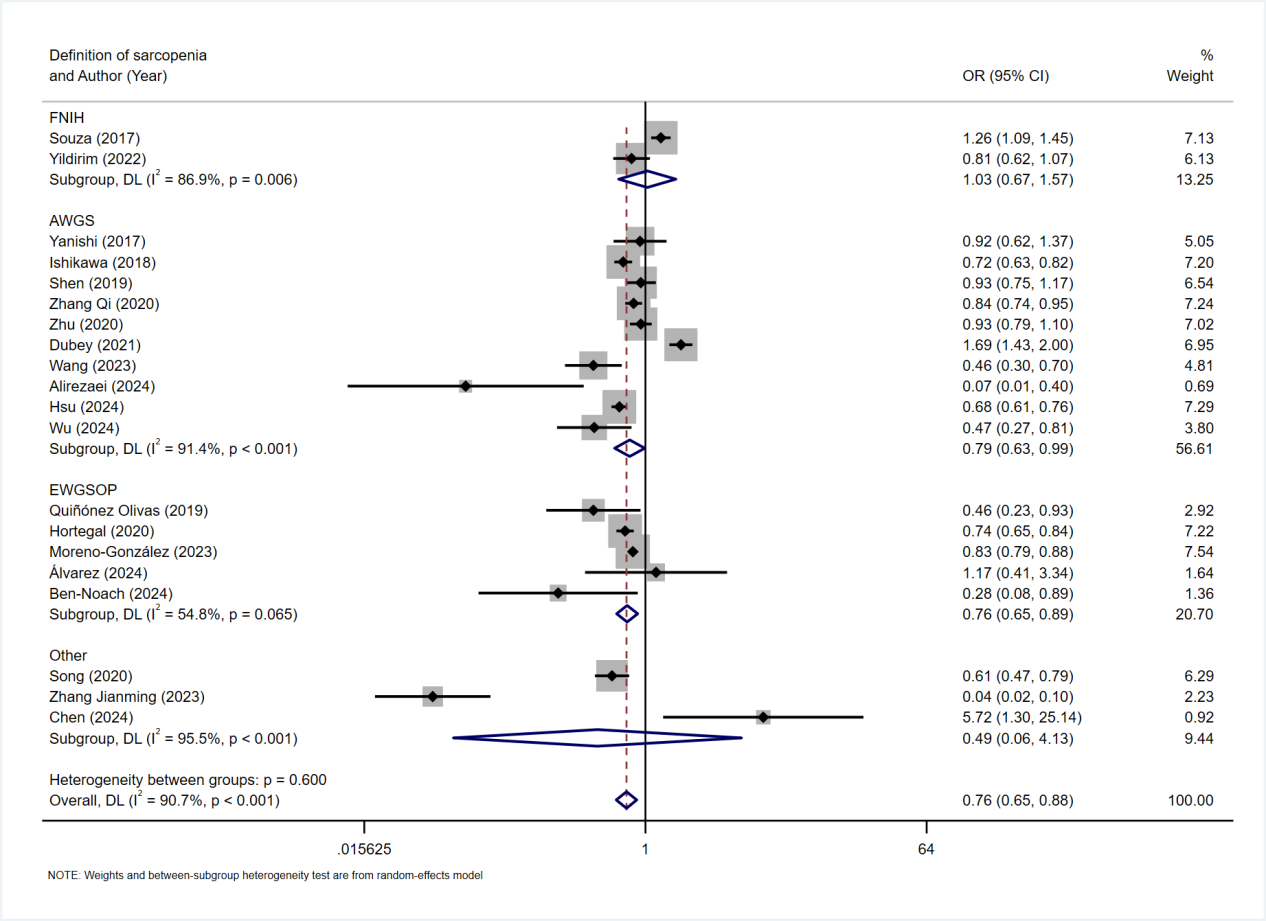
*

Figure S 27Forest Plot of the Subgroup Analysis of the Association Between BMI and Sarcopenia in Patients with CKD Based on the Definition of Sarcopenia

*
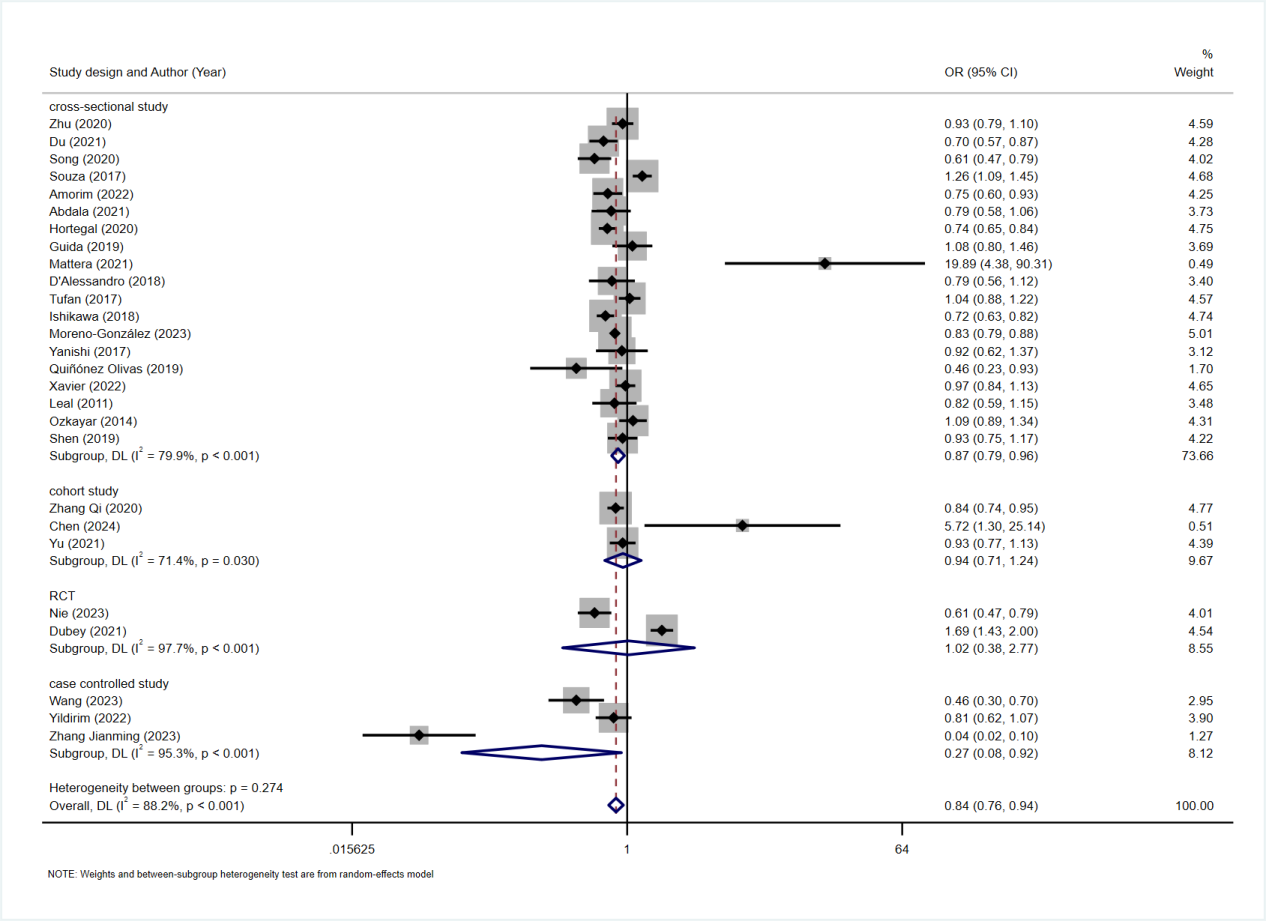
*

Figure S 28Forest Plot of the Subgroup Analysis of the Association Between BMI and Sarcopenia in Patients with CKD Based on the Study Design

*
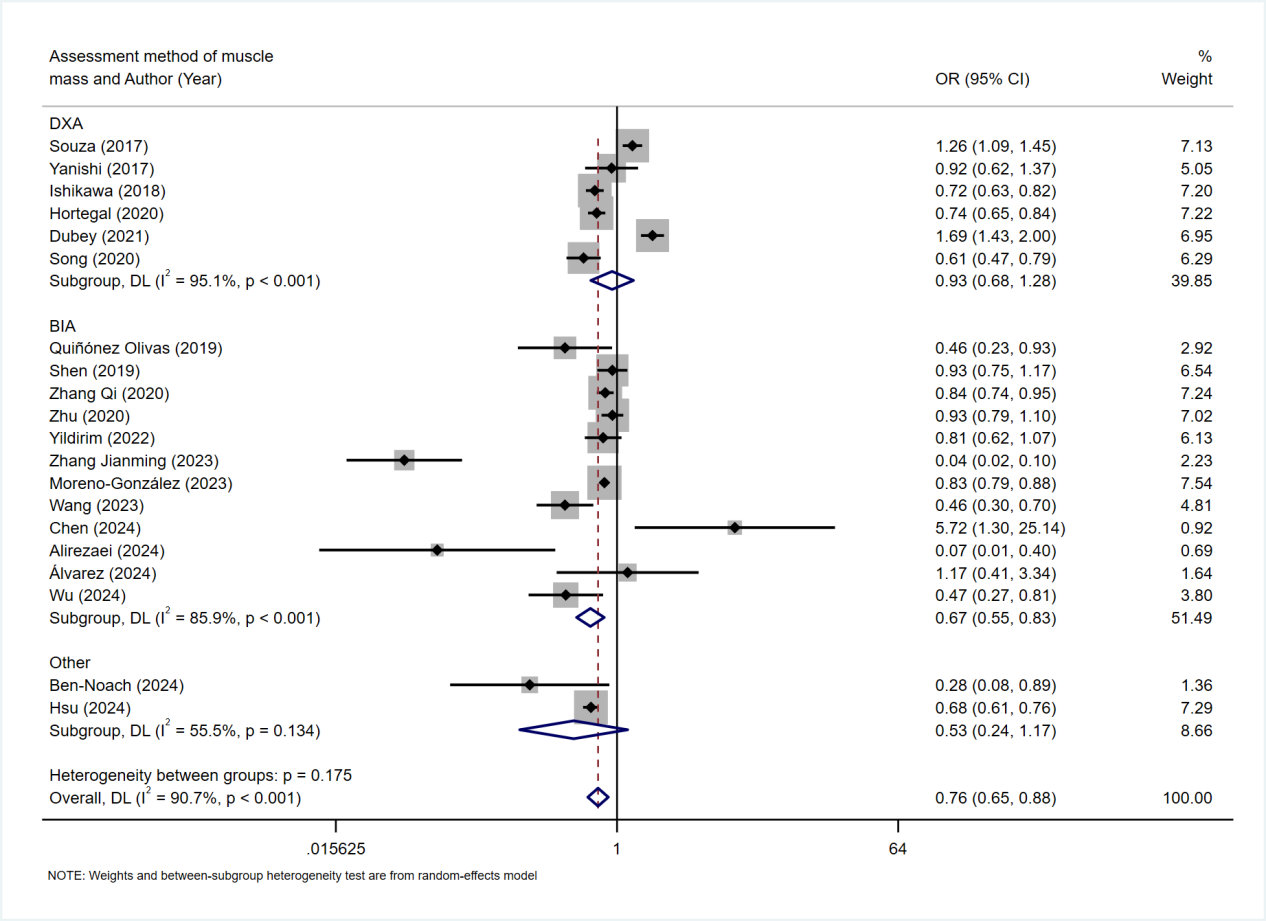
*

Figure S 29Forest Plot of the Subgroup Analysis of the Association Between BMI and Sarcopenia in Patients with CKD Based on the Assessment Method of Muscle Mass

*
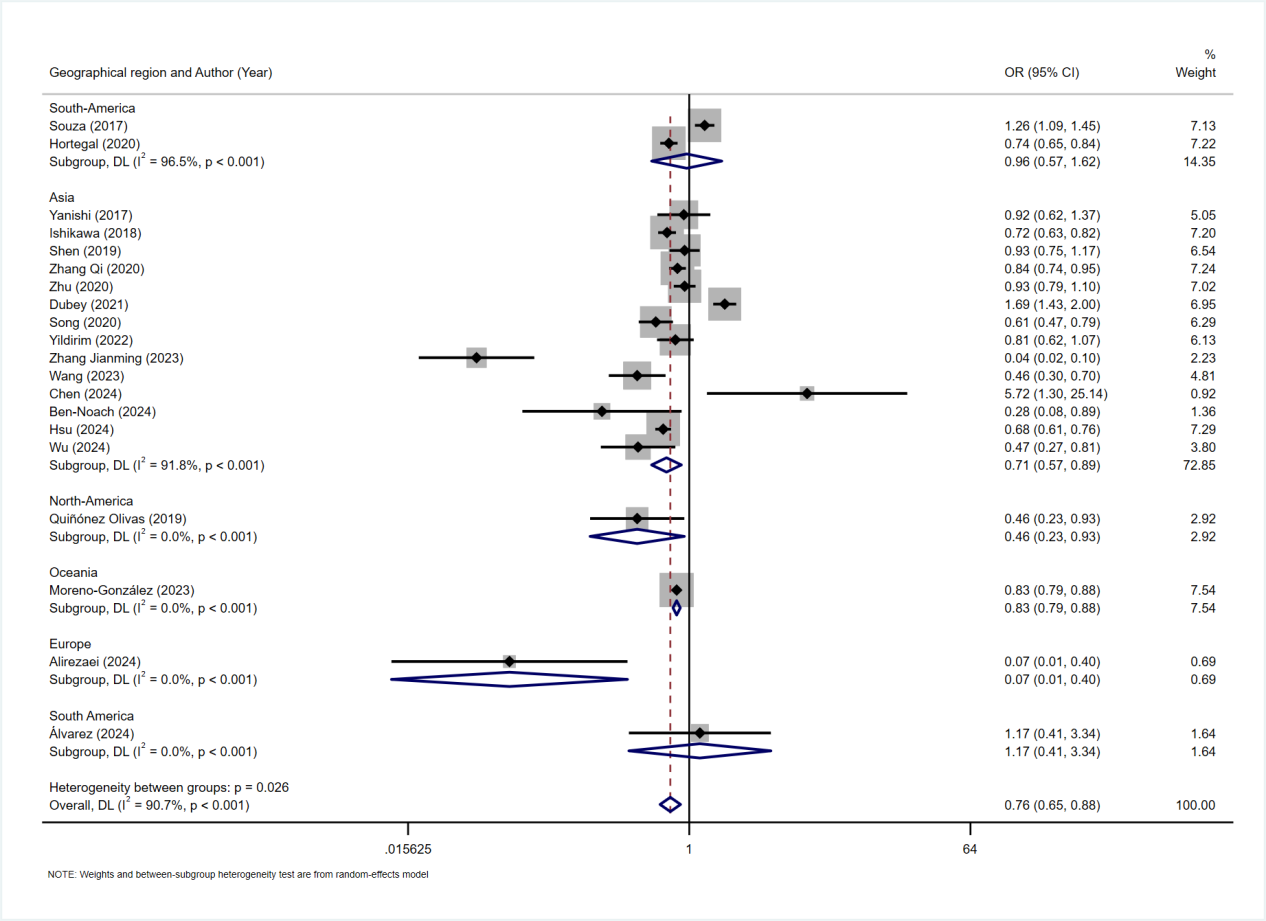
*

Figure S 30Forest Plot of the Subgroup Analysis of the Association Between BMI and Sarcopenia in Patients with CKD Based on the Geographical Region

*
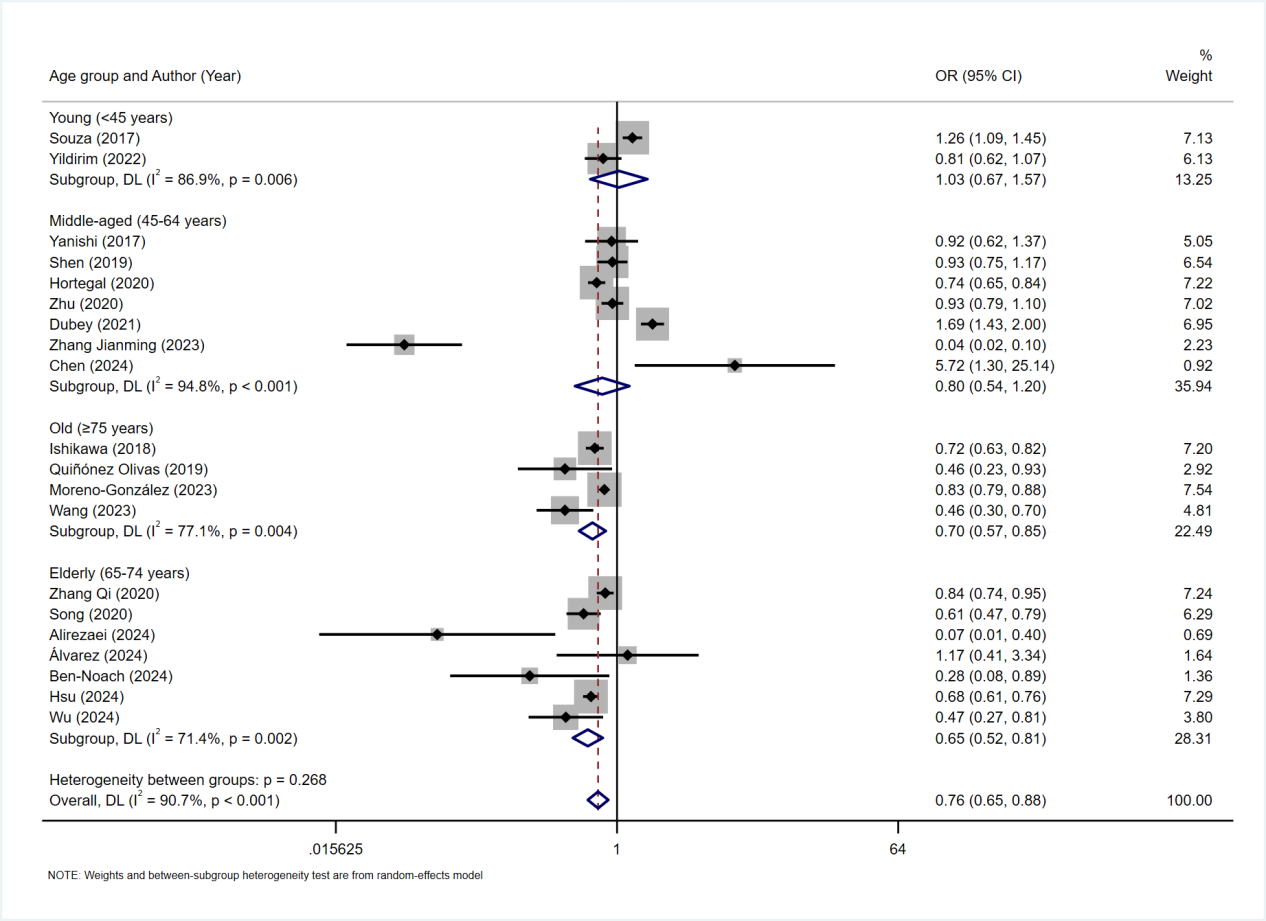
*

Figure S 31Forest Plot of the Subgroup Analysis of the Association Between BMI and Sarcopenia in Patients with CKD Based on the Age Group

Figure S 32 Meta-Regression Analysis of the Association Between BMI and Sarcopenia in Patients with CKD

*Bicarbonate*

**

Figure S 33Sensitivity Analysis of the Association Between Bicarbonate and Sarcopenia in Patients with CKD

*
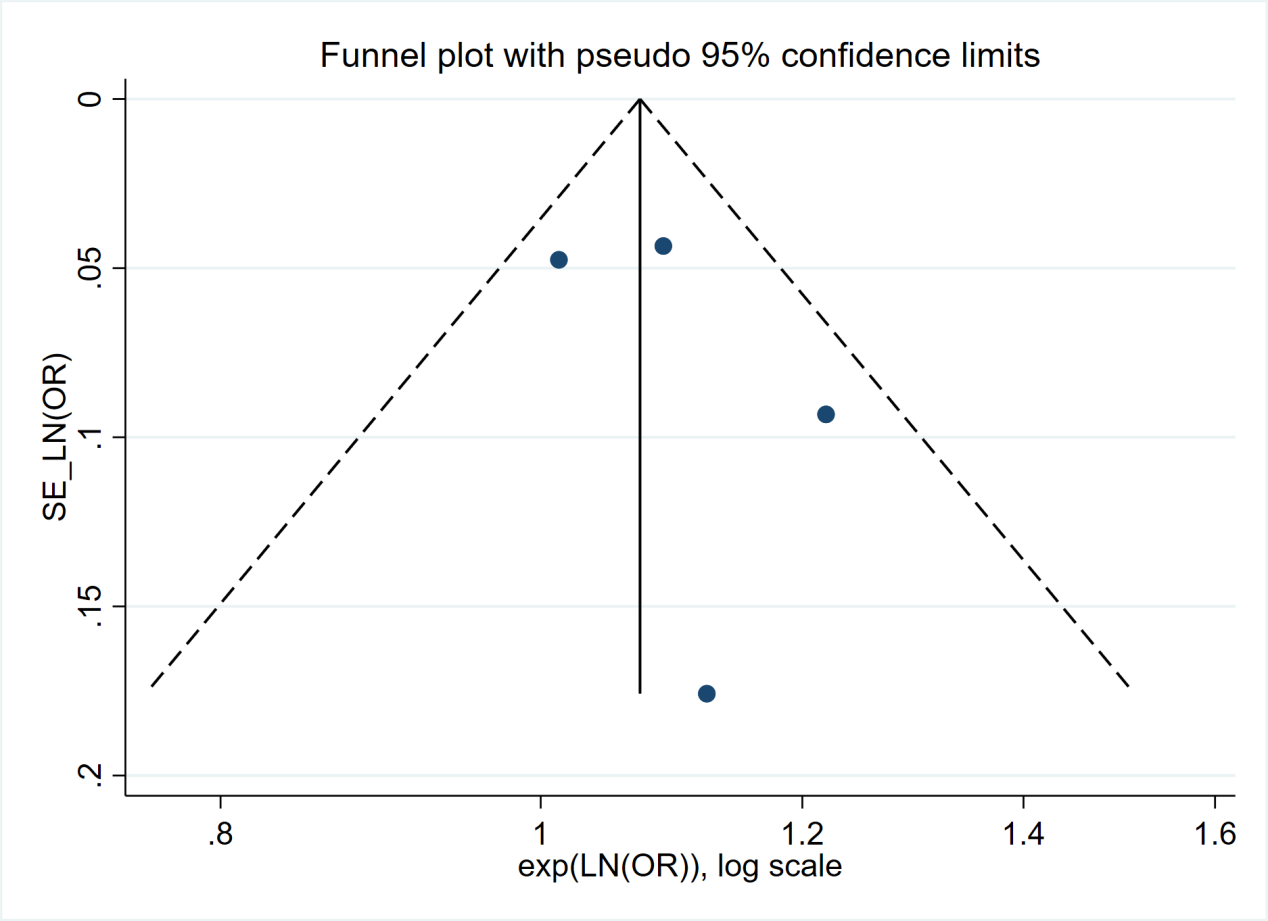
*

Figure S 34Funnel Plot of the Association Between Bicarbonate and Sarcopenia in Patients with Chronic Kidney Disease

*
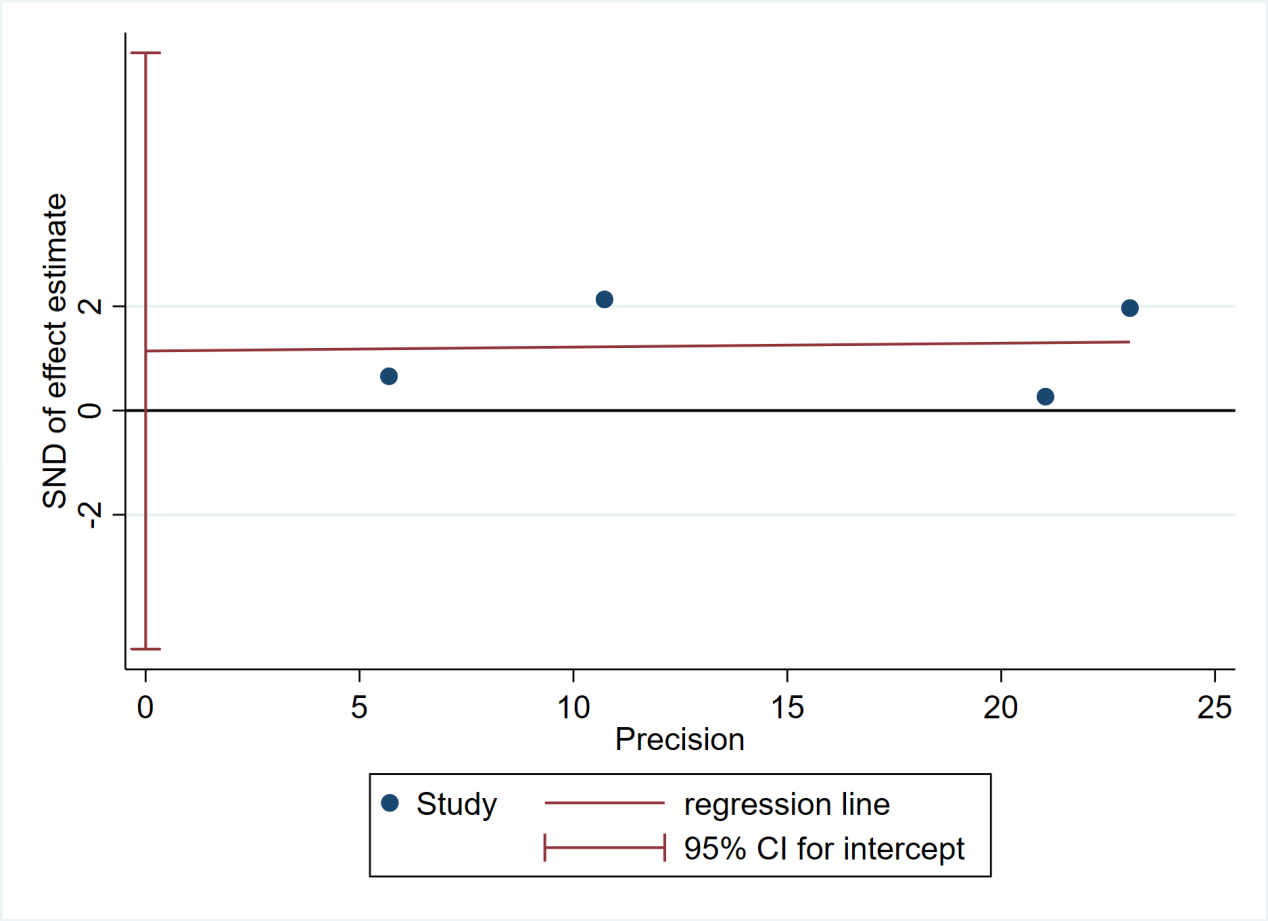
*

**

Figure S 35Egger's Test of the Association Between Bicarbonate and Sarcopenia in Patients with CKD

*Body protein content*

**

Figure S 36Sensitivity Analysis of the Association Between Body Protein Content and Sarcopenia in Patients with CKD

*
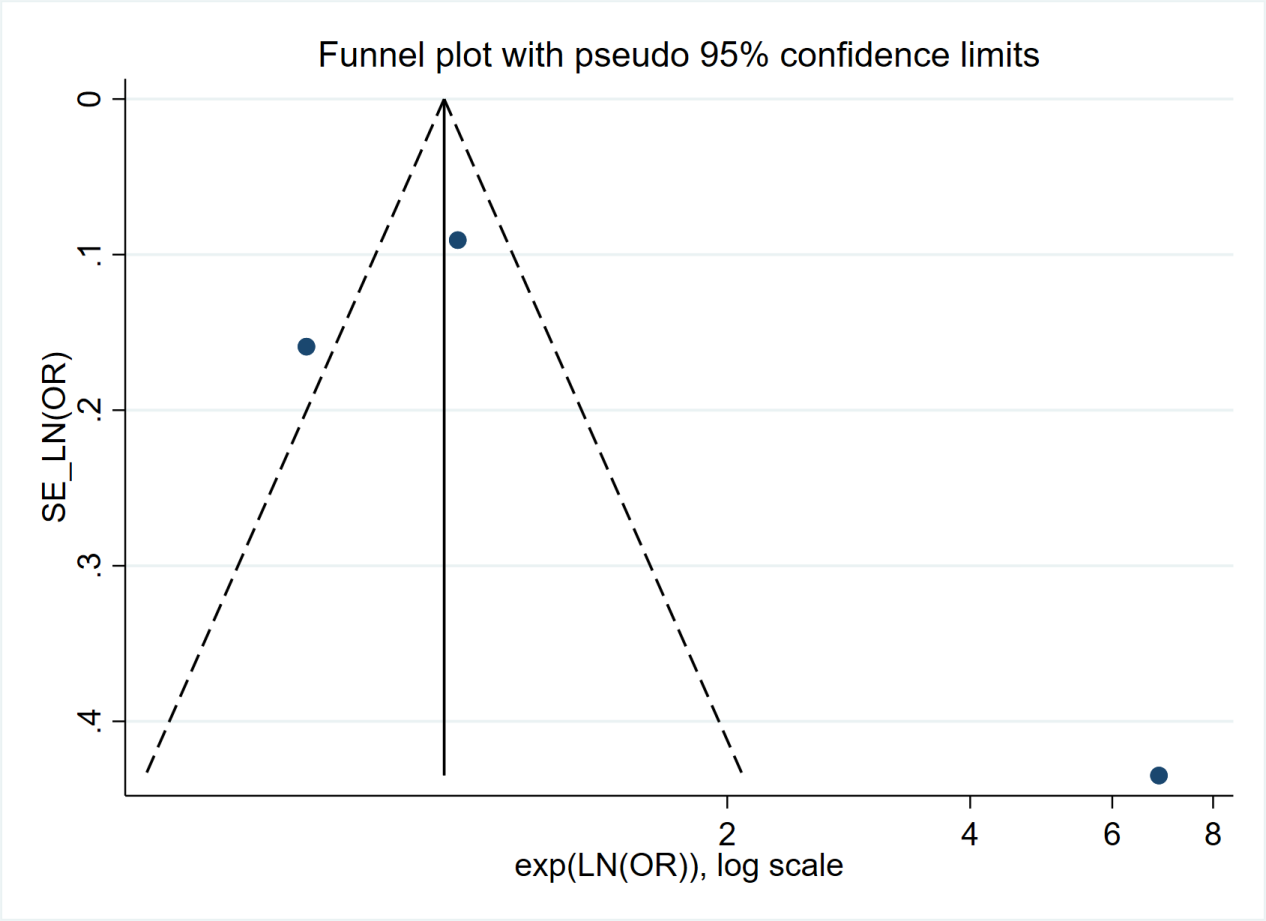
*

Figure S 37Funnel Plot of the Association Between Body Protein Content and Sarcopenia in Patients with Chronic Kidney Disease

*Figure 7 Funnel Plot of the Association Between Body Protein Content and Sarcopenia in Patients with Chronic Kidney Disease*

*
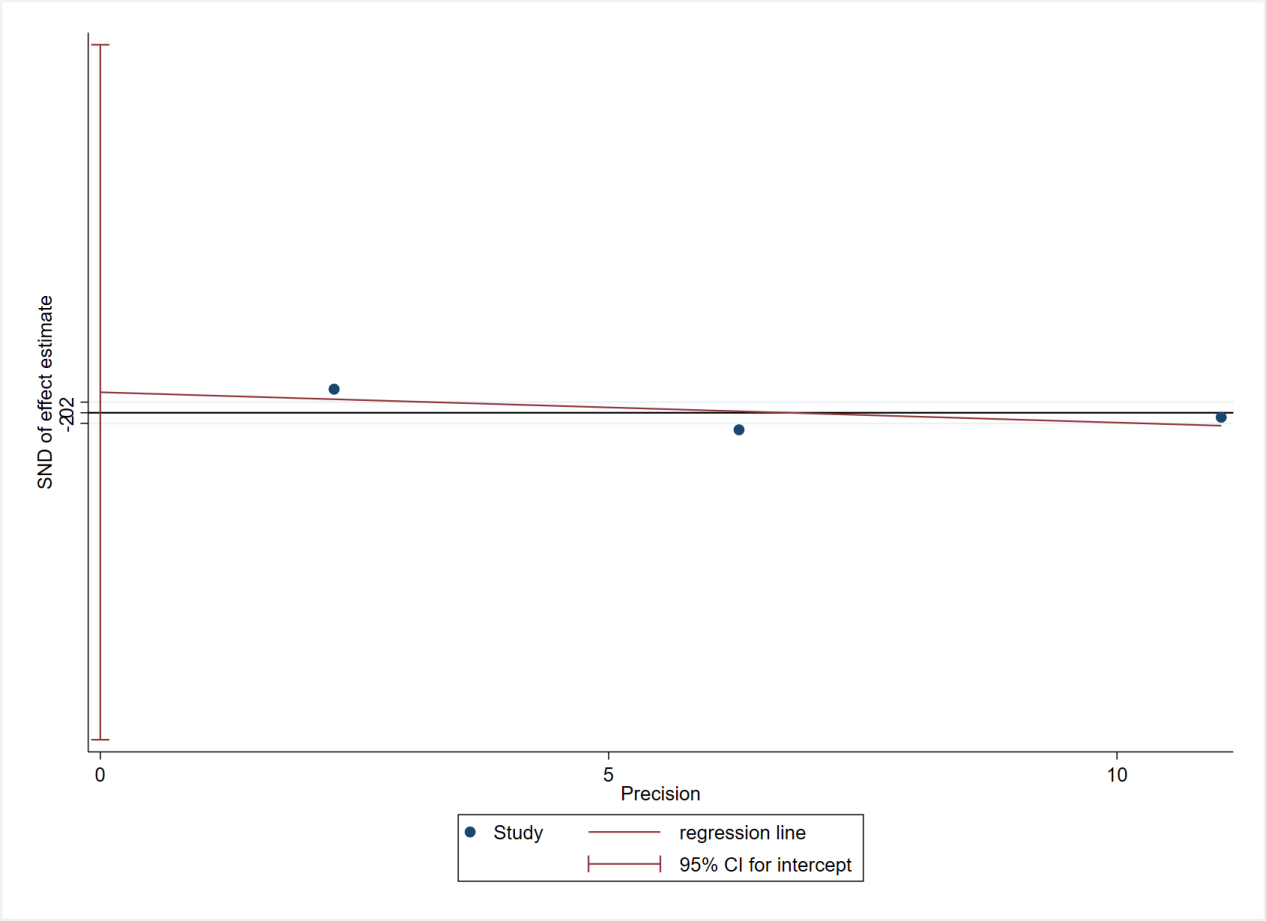
*

**

Figure S 38Egger's Test of the Association Between Body Protein Content and Sarcopenia in Patients with CKD

*Results After Excluding Studies with High Heterogeneity in Sensitivity Analysis*

*The results showed significant heterogeneity. Sensitivity analysis was conducted using the leave-one-out method, and removing any single study did not affect the overall results, indicating the stability of the meta-analysis. Due to the limited number of studies, subgroup analysis and meta-regression analysis were not performed.*

*Carbon dioxide binding capacity*

Figure S 39Sensitivity Analysis of the Association Between Carbon Dioxide Binding Capacity and Sarcopenia in Patients with CKD

*
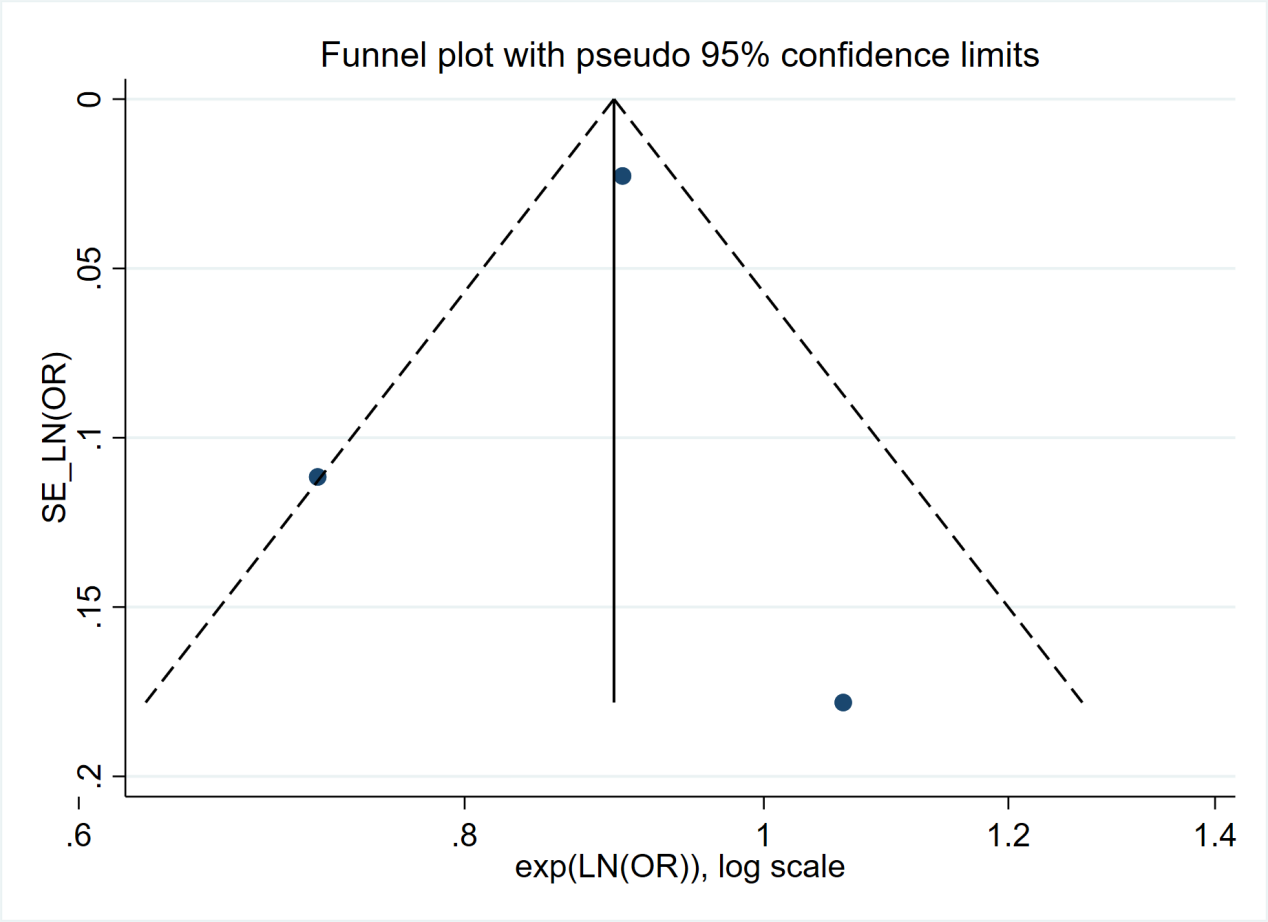
*

Figure S 40Funnel Plot of the Association Between Carbon Dioxide Binding Capacity and Sarcopenia in Patients with Chronic Kidney Disease

*
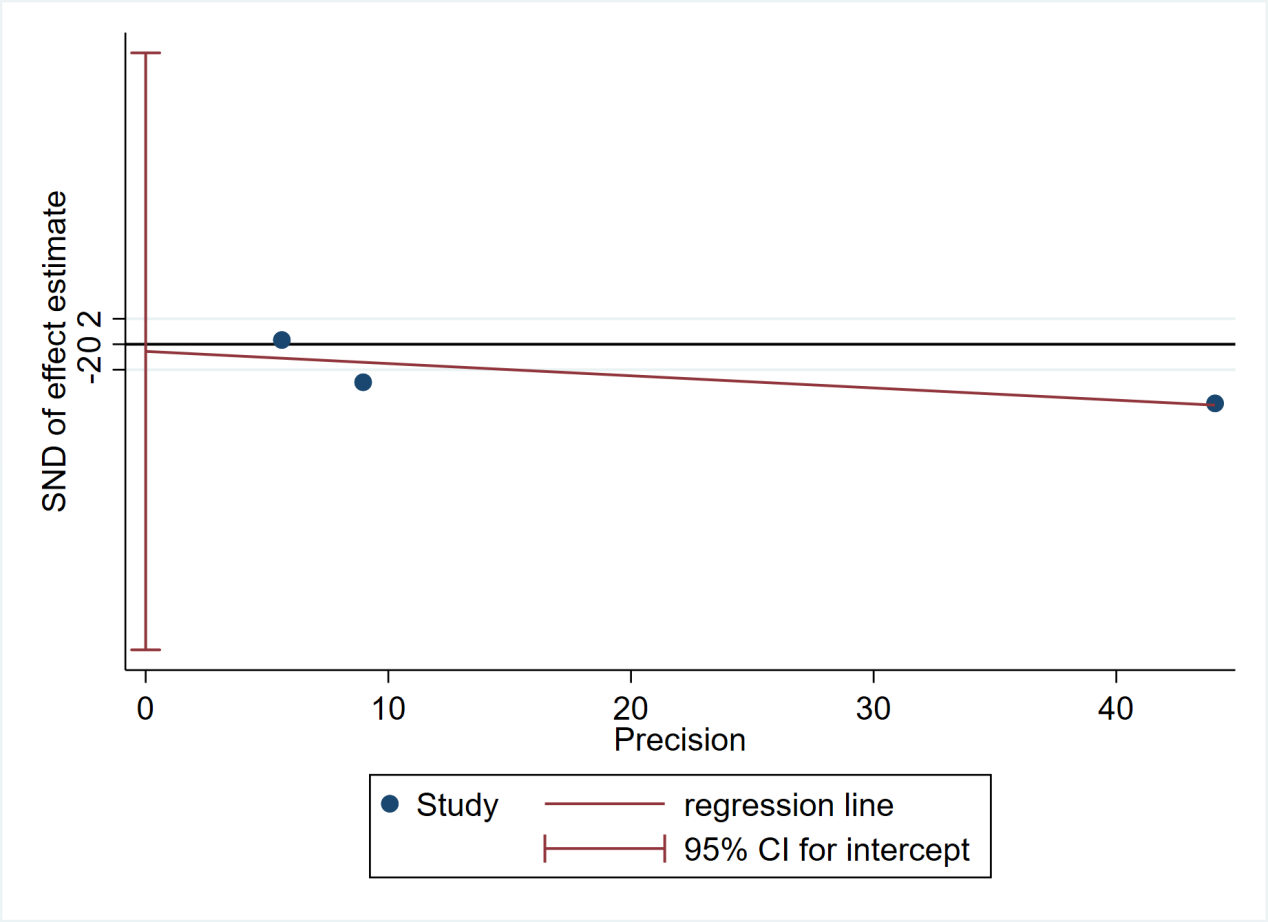
*

*
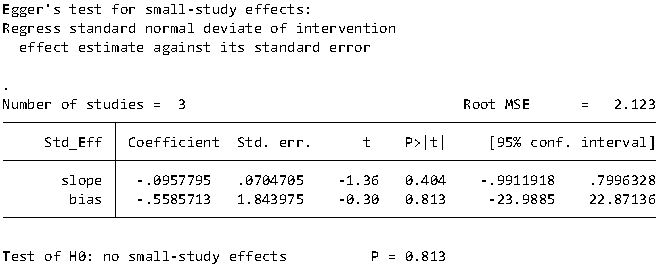
*

Figure S 41Egger's Test of the Association Between Carbon Dioxide Binding Capacity and Sarcopenia in Patients with CKD

*COPD(Chronic Obstructive Pulmonary Disease)*

**

Figure S 42 Sensitivity Analysis of the Association Between COPD and Sarcopenia in Patients with CKD

*
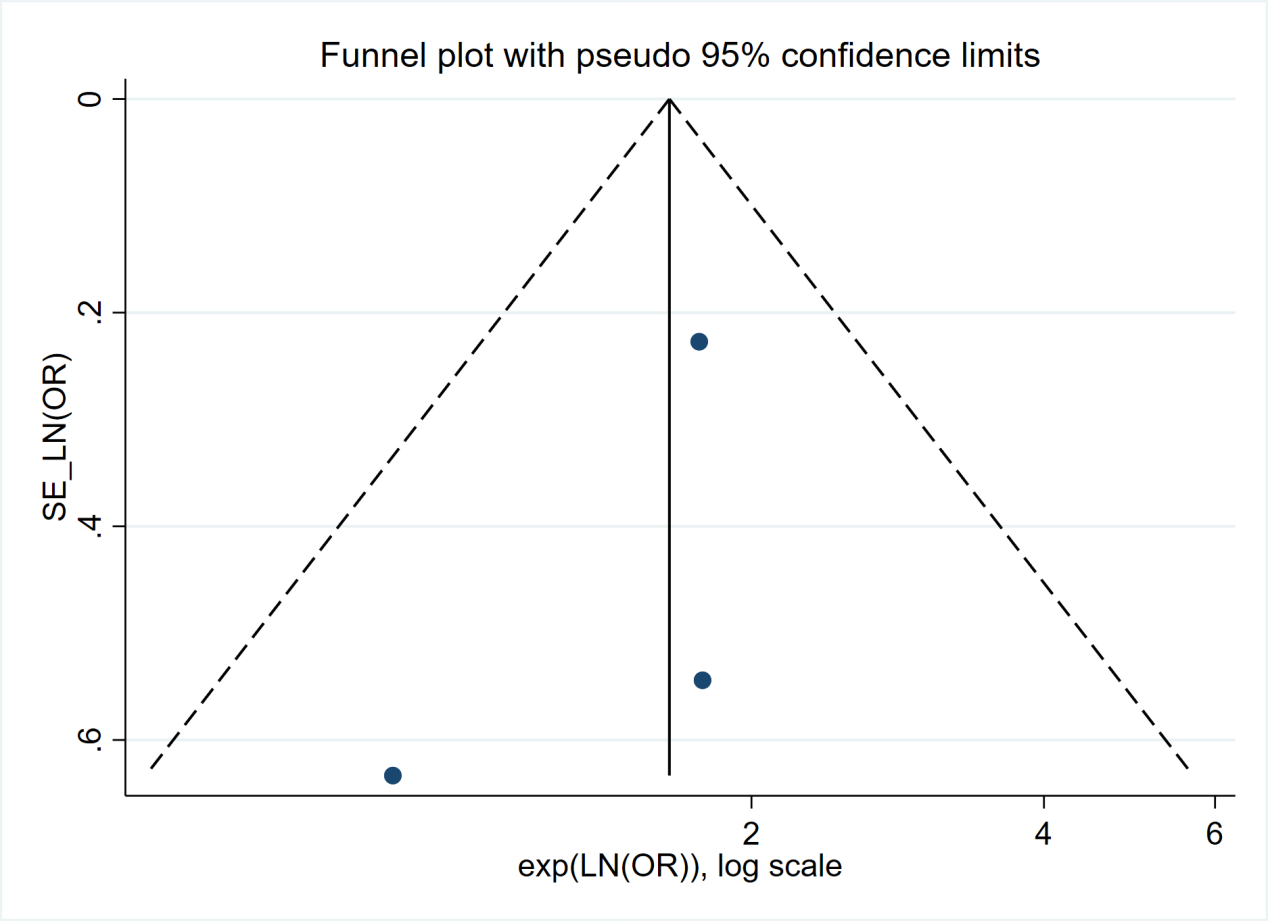
*

Figure S 43Funnel Plot of the Association Between COPD and Sarcopenia in Patients with Chronic Kidney Disease

*
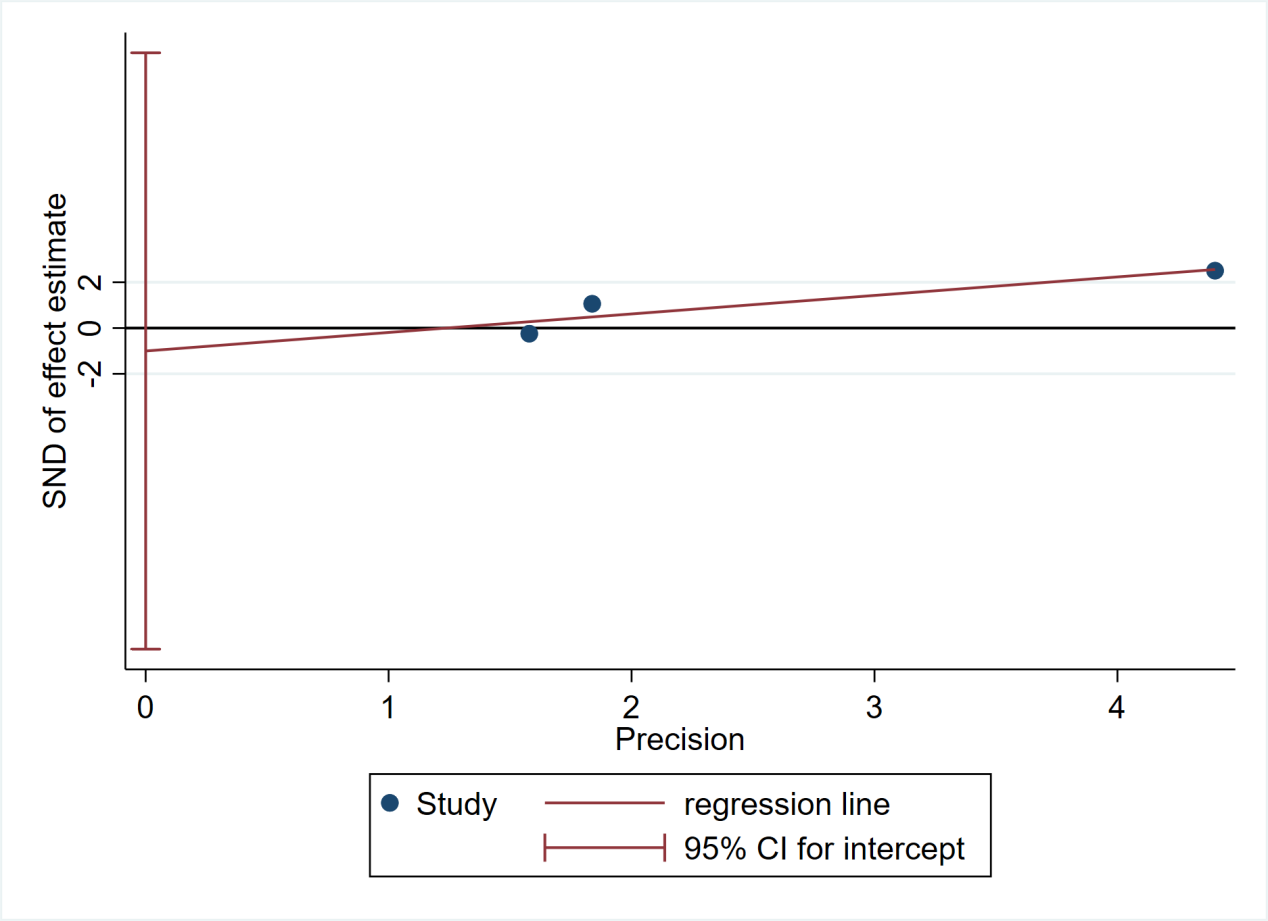
*

**

Figure S 44Egger's Test of the Association Between COPD and Sarcopenia in Patients with CKD

*CRP(C-reactive Protein)*

Figure S 45Sensitivity Analysis of the Association Between CRP and Sarcopenia in Patients with CKD

*
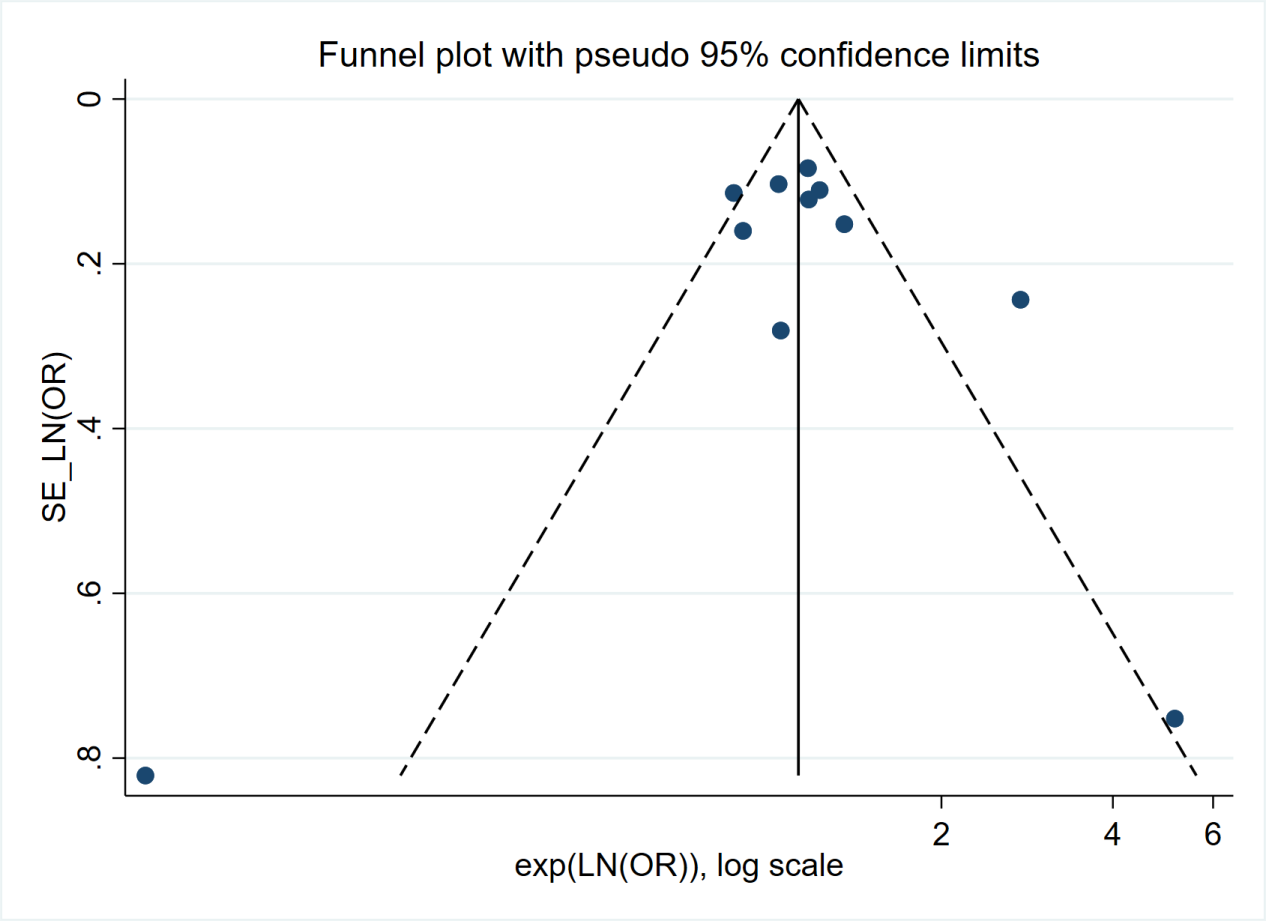
*

Figure S 46Funnel Plot of the Association Between CRP and Sarcopenia in Patients with Chronic Kidney Disease

*
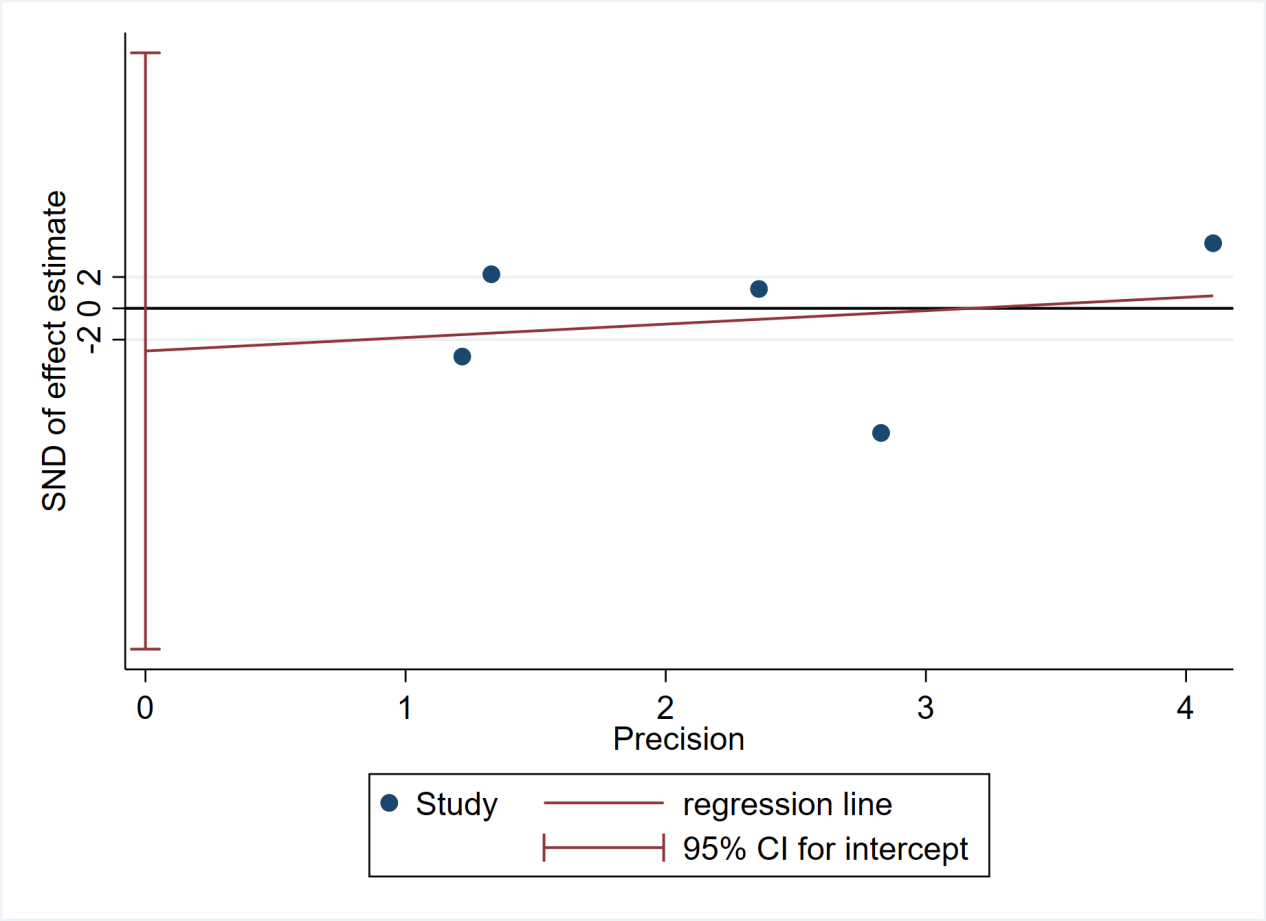
*

Figure S 47Egger's Test of the Association Between CRP and Sarcopenia in Patients with CKD

*
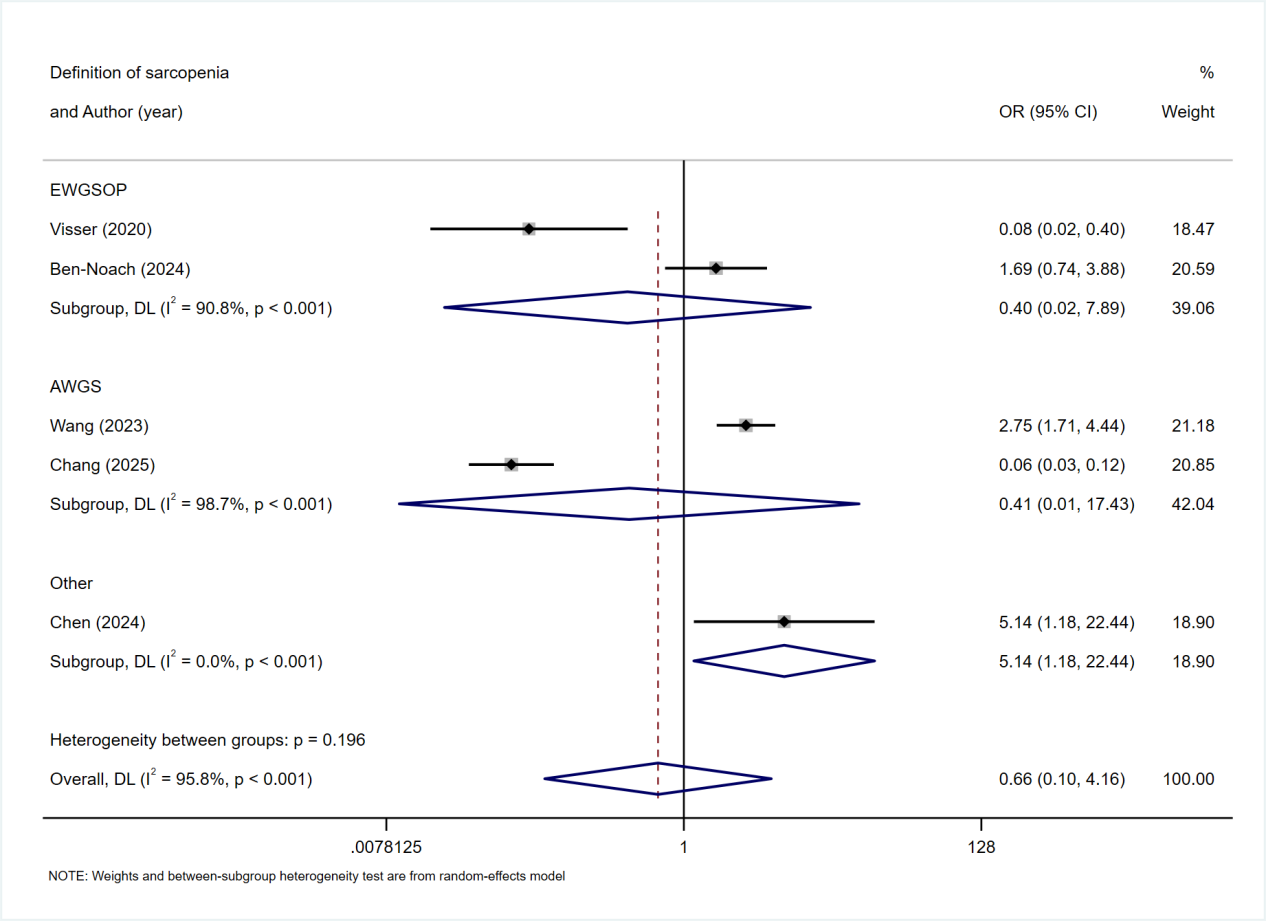
*

Figure S 48Forest Plot of the Subgroup Analysis of the Association Between CRP and Sarcopenia in Patients with CKD Based on the Definition of Sarcopenia

*
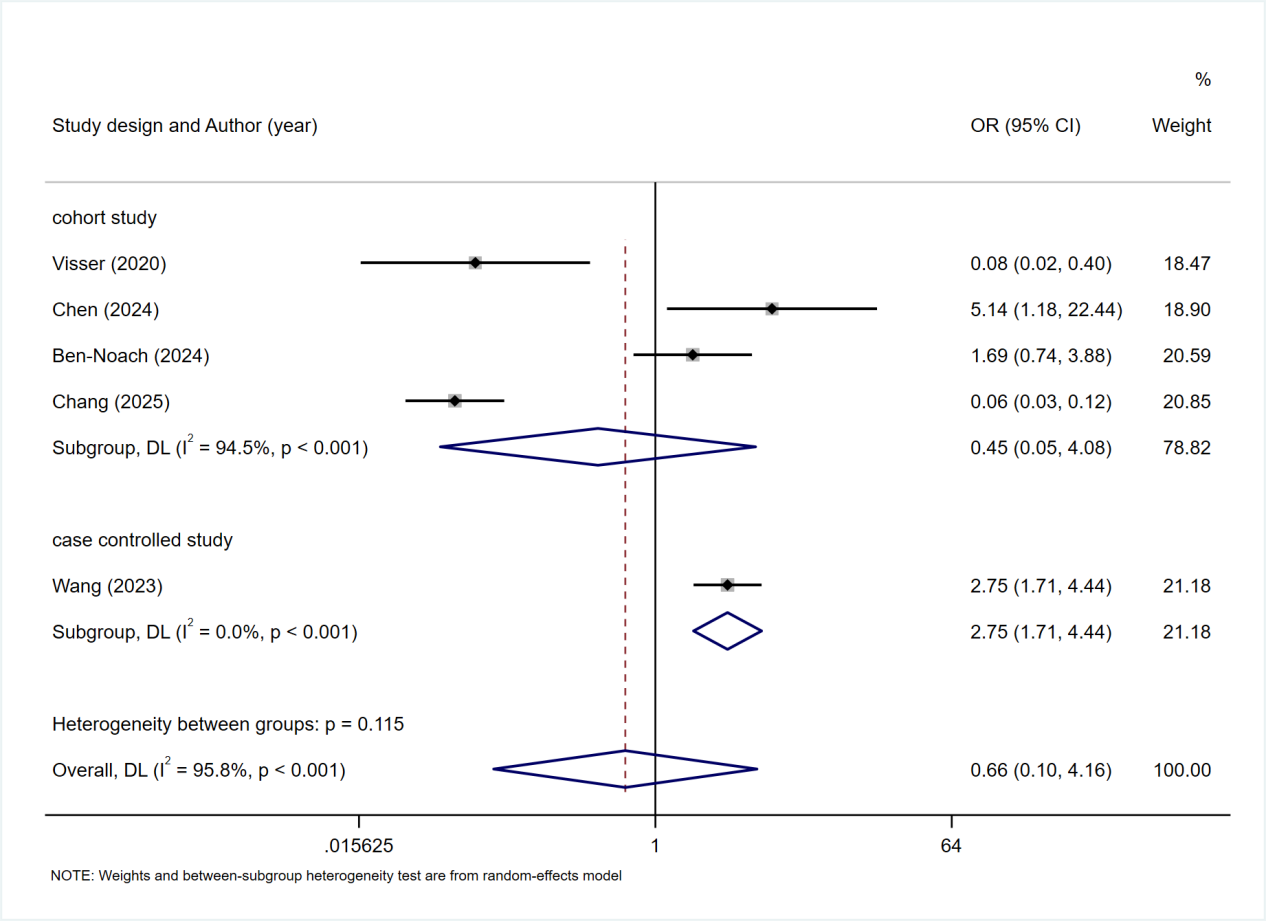
*

Figure S 49Forest Plot of the Subgroup Analysis of the Association Between CRP and Sarcopenia in Patients with CKD Based on the Study Design

*
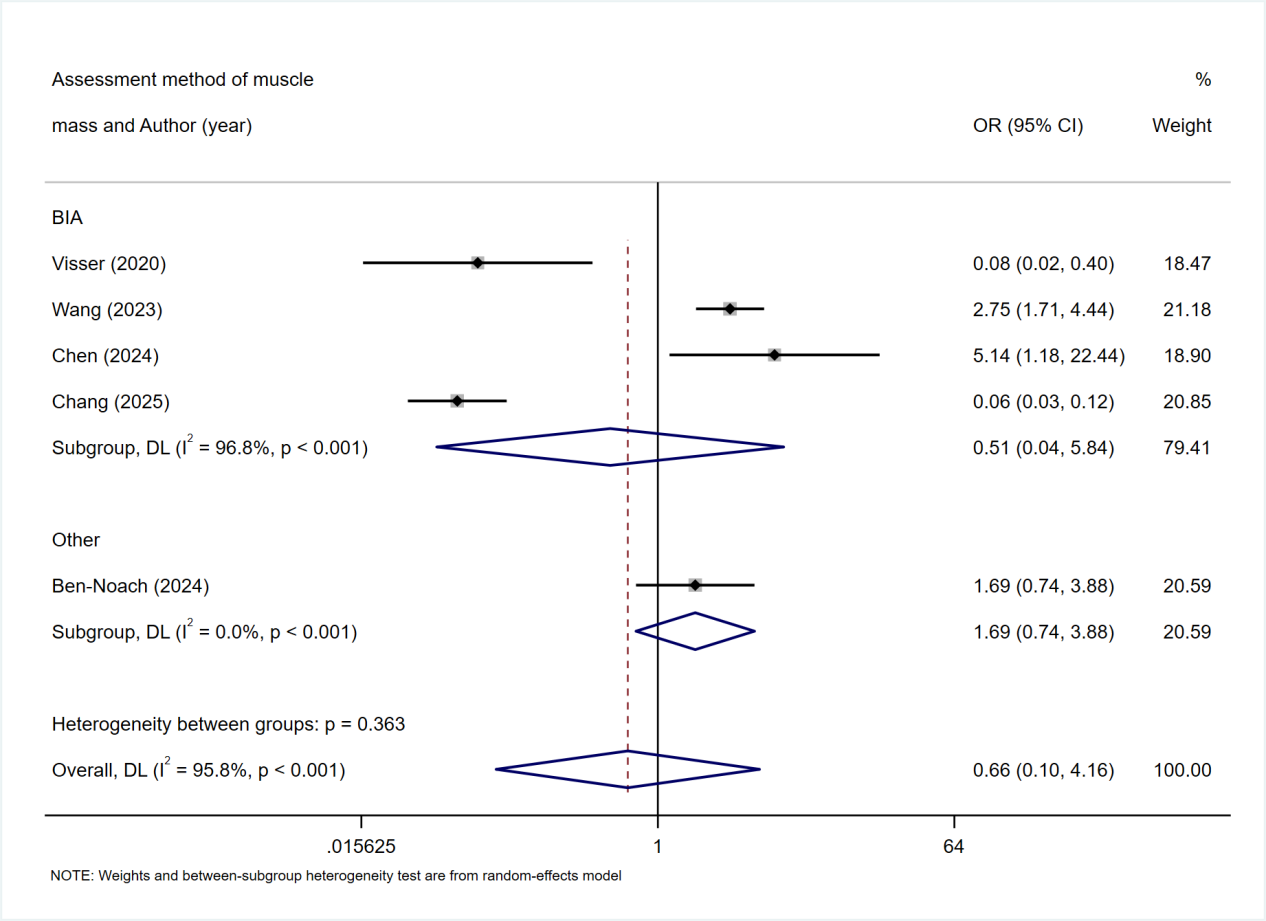
*

Figure S 50Forest Plot of the Subgroup Analysis of the Association Between CRP and Sarcopenia in Patients with CKD Based on the Assessment Method of Muscle Mass

*
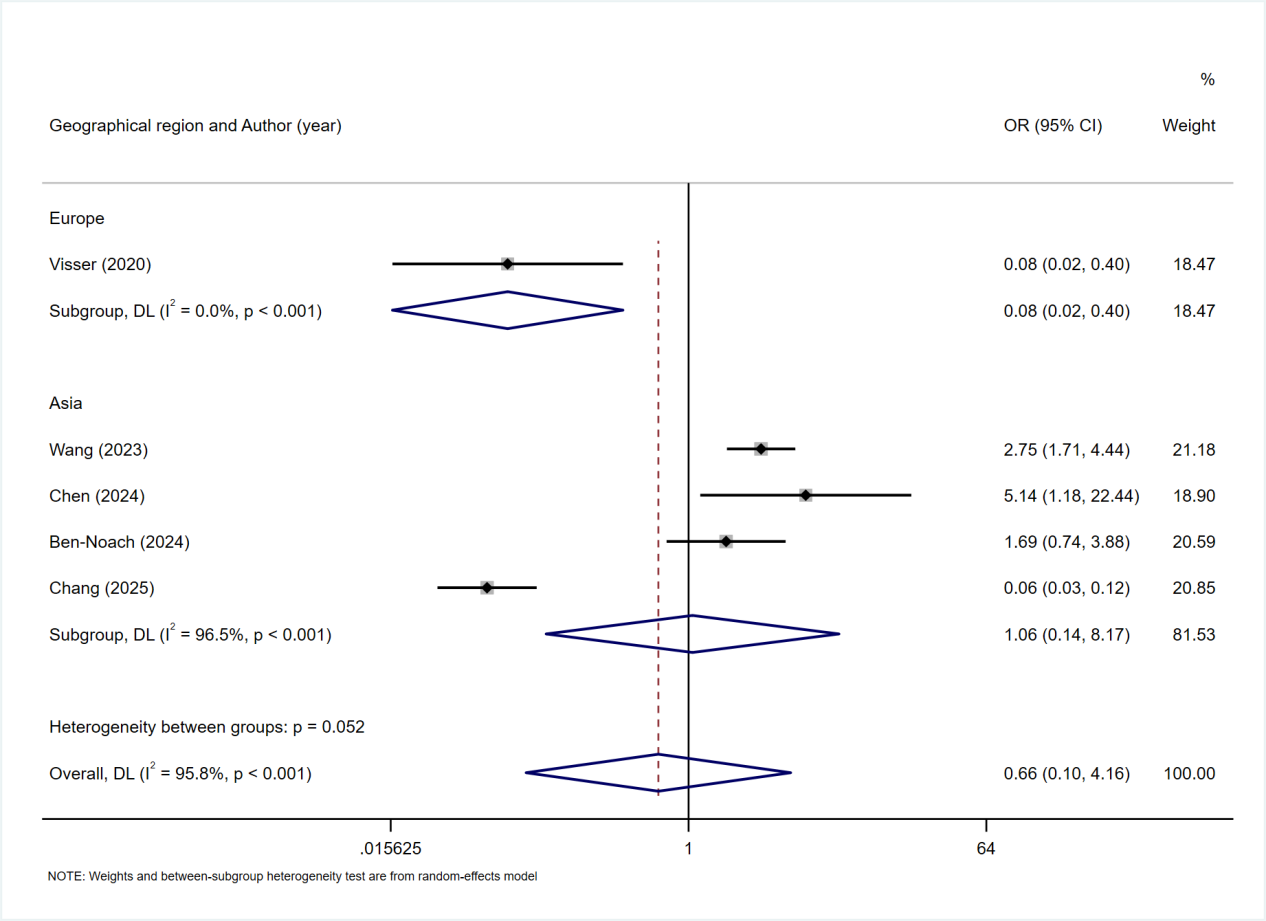
*

Figure S 51Forest Plot of the Subgroup Analysis of the Association Between CRP and Sarcopenia in Patients with CKD Based on the Geographical Region

*
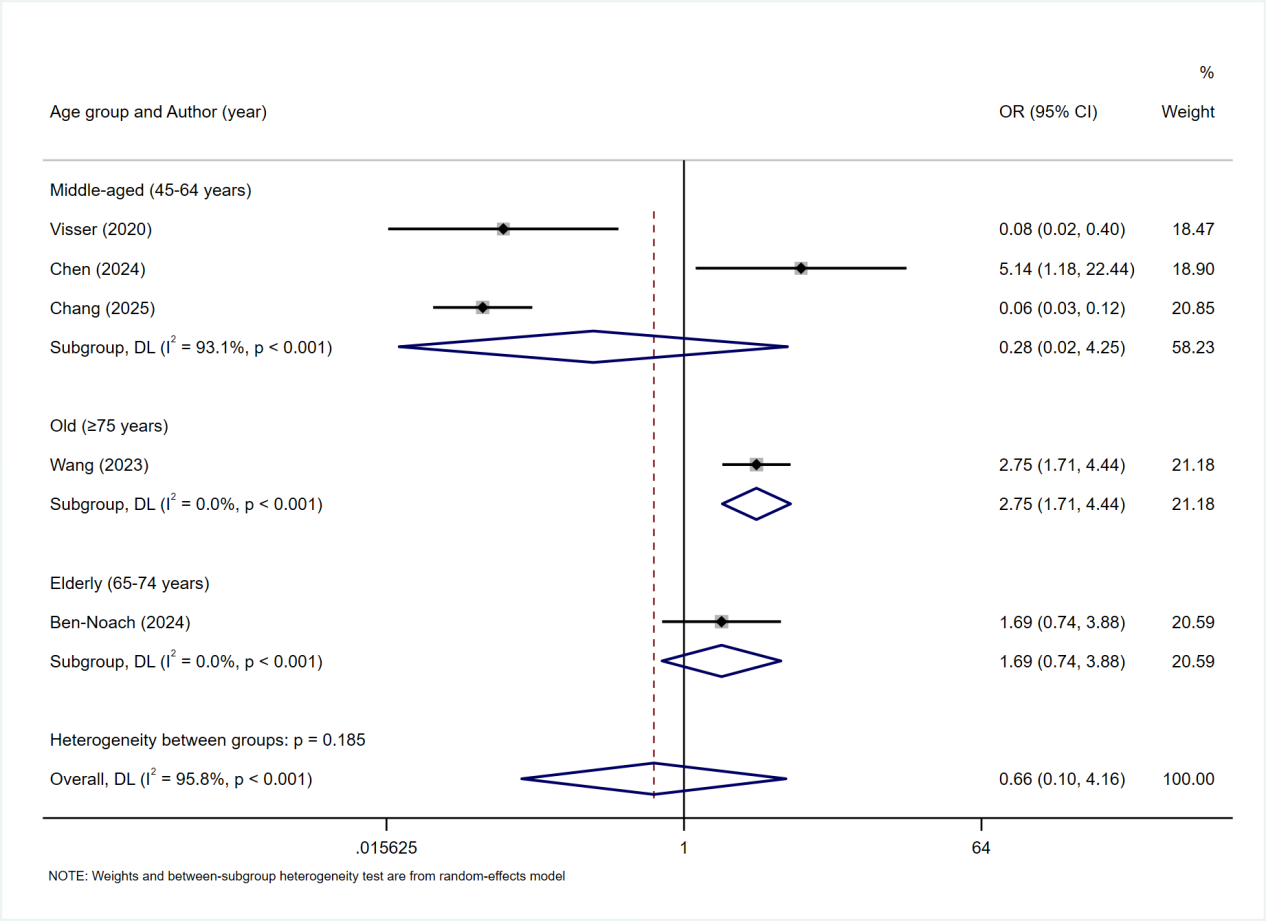
*

Figure S 52Forest Plot of the Subgroup Analysis of the Association Between CRP and Sarcopenia in Patients with CKD Based on the Age Group

*Despite conducting comprehensive analyses, including sensitivity analysis, subgroup analysis, and meta-regression, no specific source of heterogeneity was identified. The observed heterogeneity may be attributed to unmeasured or unreported factors, variations in study design, or differences in population characteristics that were not fully captured in the included studies. As a result, the source of heterogeneity remains unclear.*

*Hs-CRP(C-reactive Protein)*

Figure S 53Sensitivity Analysis of the Association Between hs-CRP and Sarcopenia in Patients with CKD

*
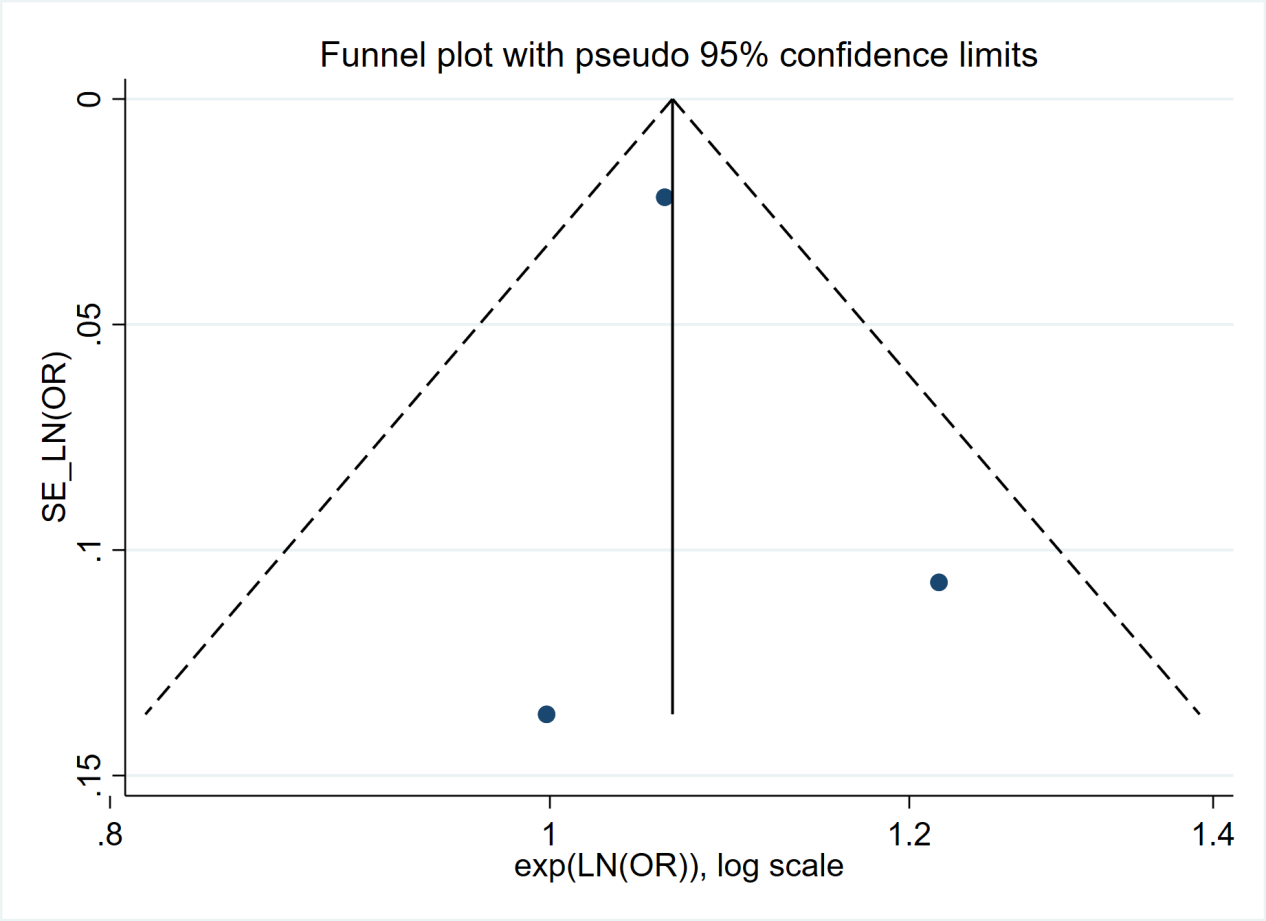
*

Figure S 54Funnel Plot of the Association Between hs-CRP and Sarcopenia in Patients with Chronic Kidney Disease

*Figure 24 Funnel Plot of the Association Between hs-CRP and Sarcopenia in Patients with Chronic Kidney Disease*

*
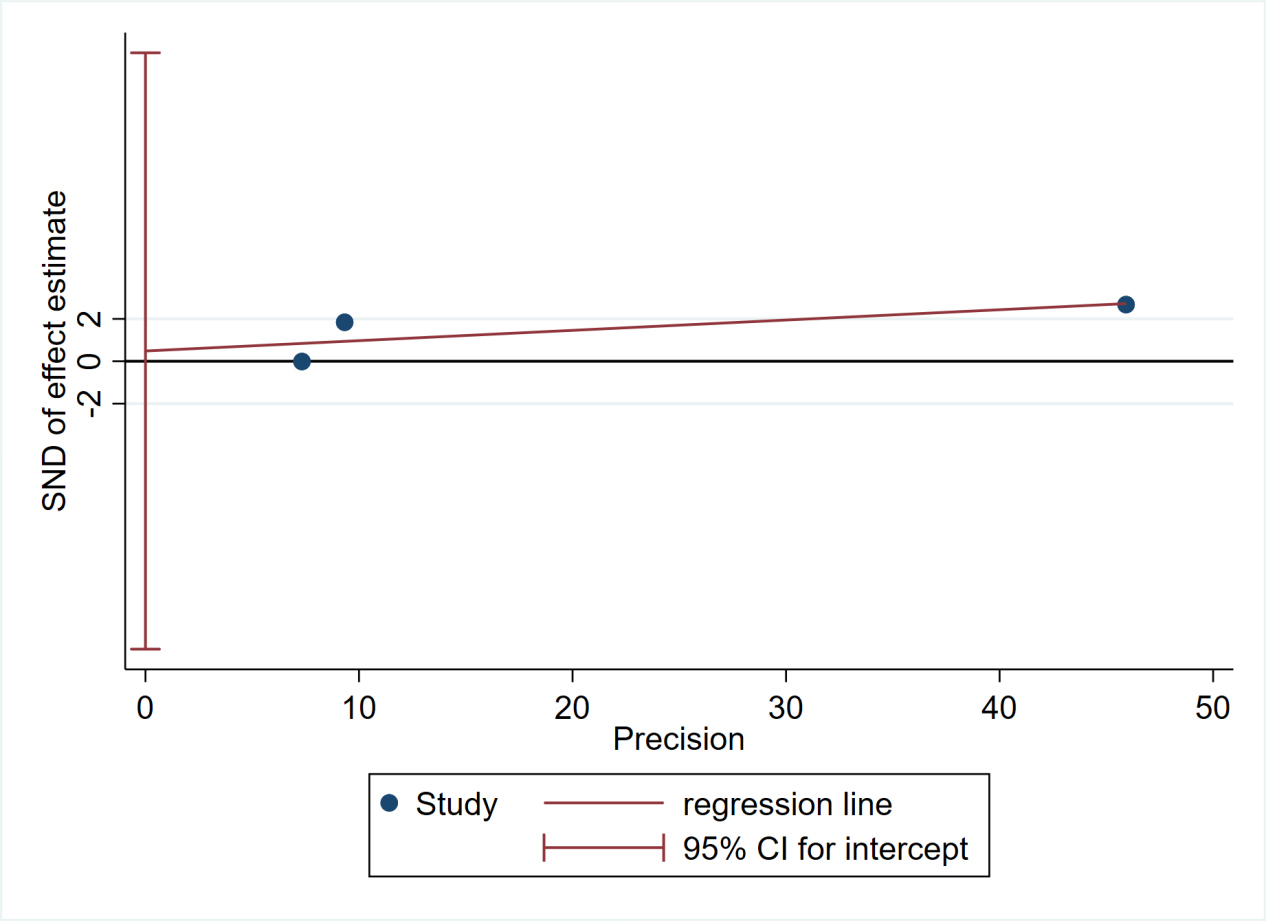
*


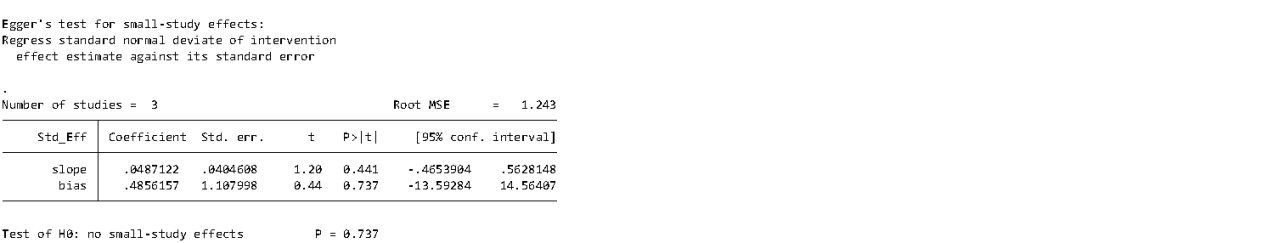


Figure S 55Egger's Test of the Association Between hs-CRP and Sarcopenia in Patients with CKD

Due to the limited number of available studies, further analysis could not be conducted.

*Diabetes*

Figure S 56Sensitivity Analysis of the Association Between Diabetes and Sarcopenia in Patients with CKD

*
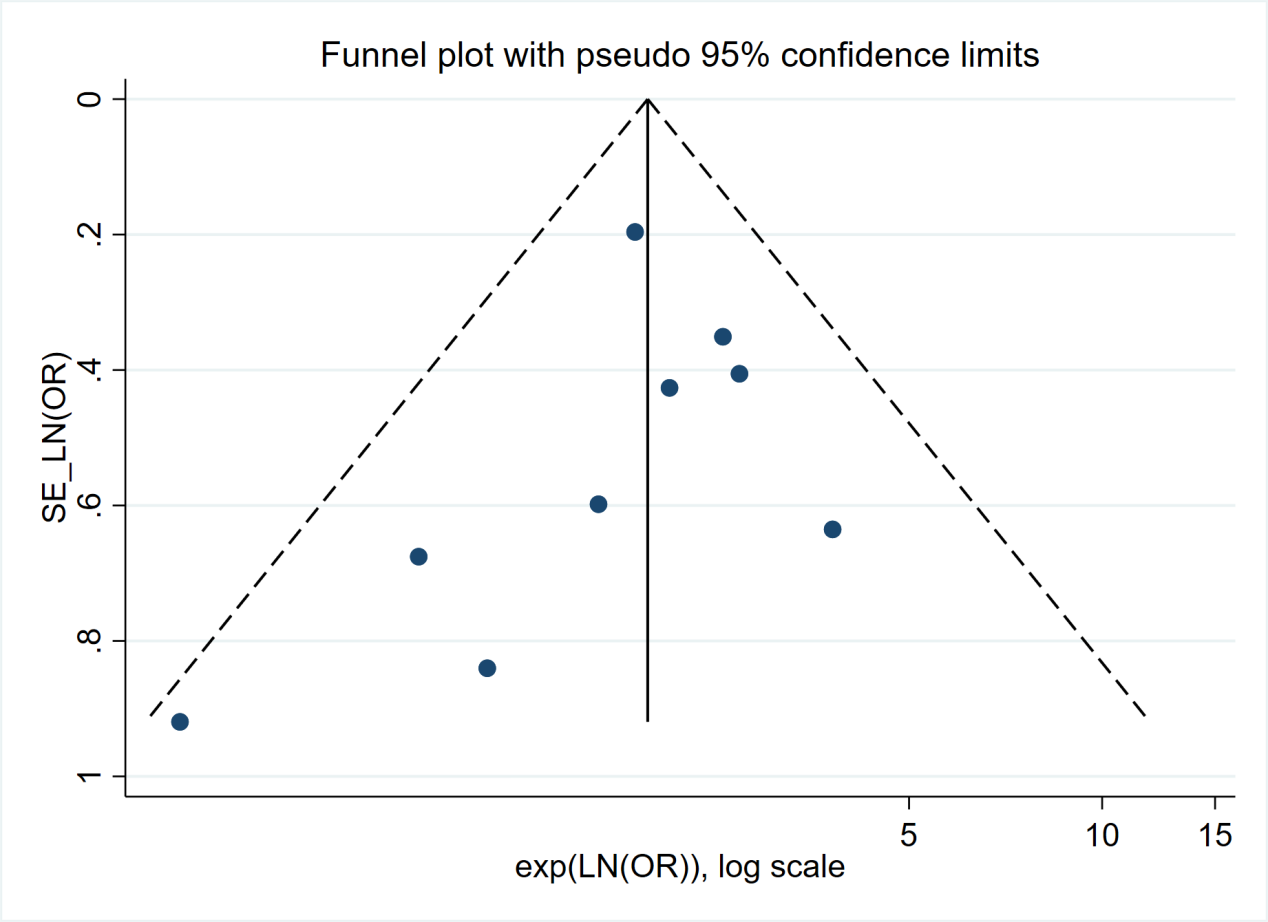
*

Figure S 57Funnel Plot of the Association Between Diabetes and Sarcopenia in Patients with Chronic Kidney Disease

*
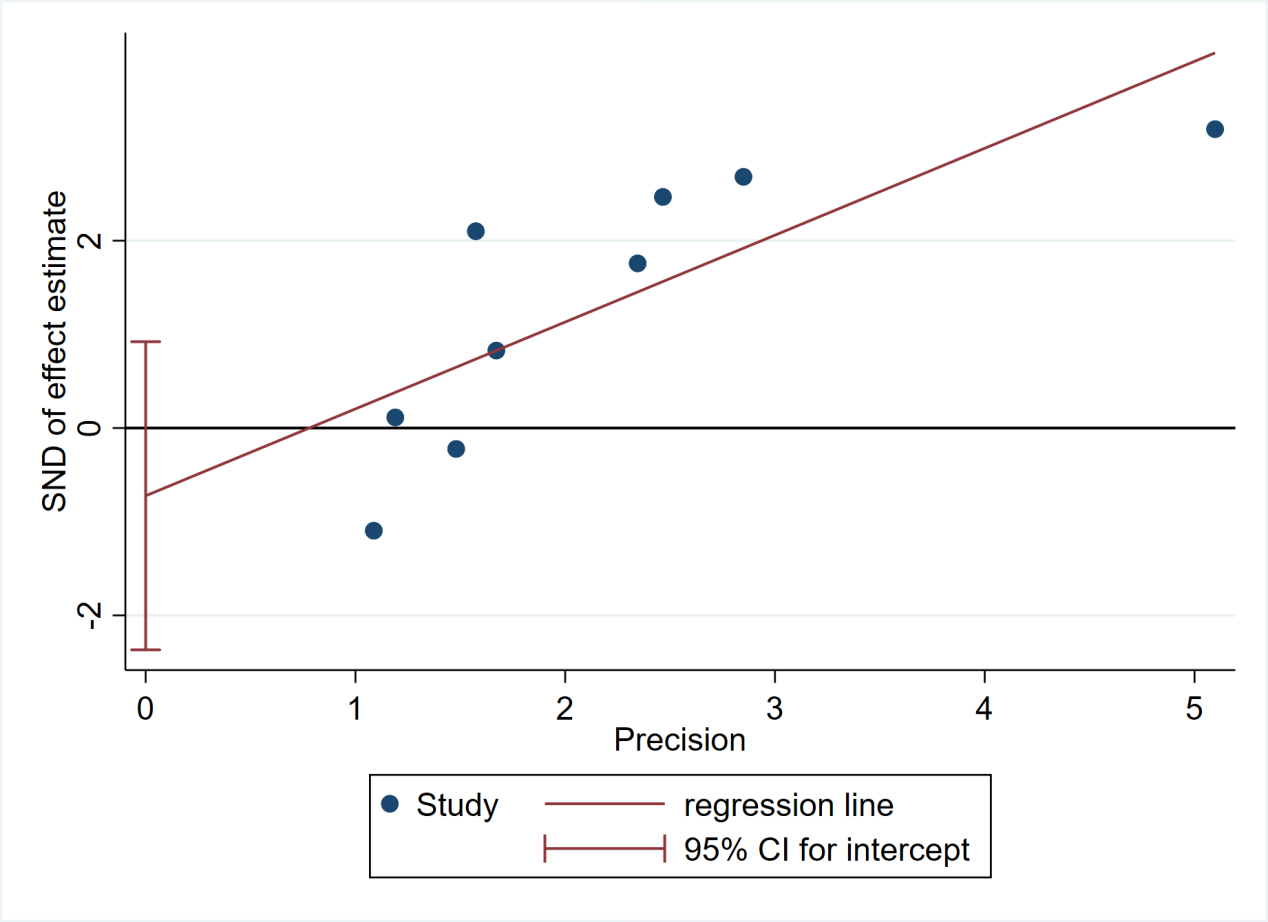
*

*
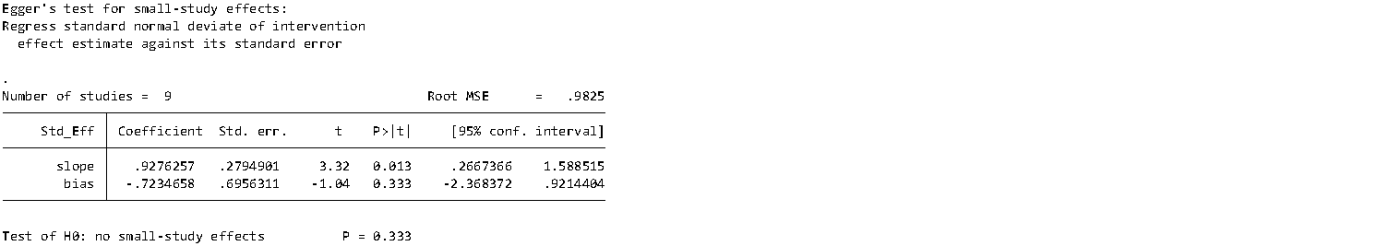
*

Figure S 58Egger's Test of the Association Between Diabetes and Sarcopenia in Patients with CKD

*Dialysis Vintage*

Figure S 59Sensitivity Analysis of the Association Between Dialysis Vintage and Sarcopenia in Patients with CKD

Figure S 60Funnel Plot of the Association Between Dialysis Vintage and Sarcopenia in Patients with CKD

*Figure 30 Funnel Plot of the Association Between Dialysis Vintage and Sarcopenia in Patients with CKD*

Figure S 61Egger's Test of the Association Between Dialysis Vintage and Sarcopenia in Patients with CKD

Diuretic use

**

Figure S 62Sensitivity Analysis of the Association Between Diuretic Use and Sarcopenia in Patients with CKD

*
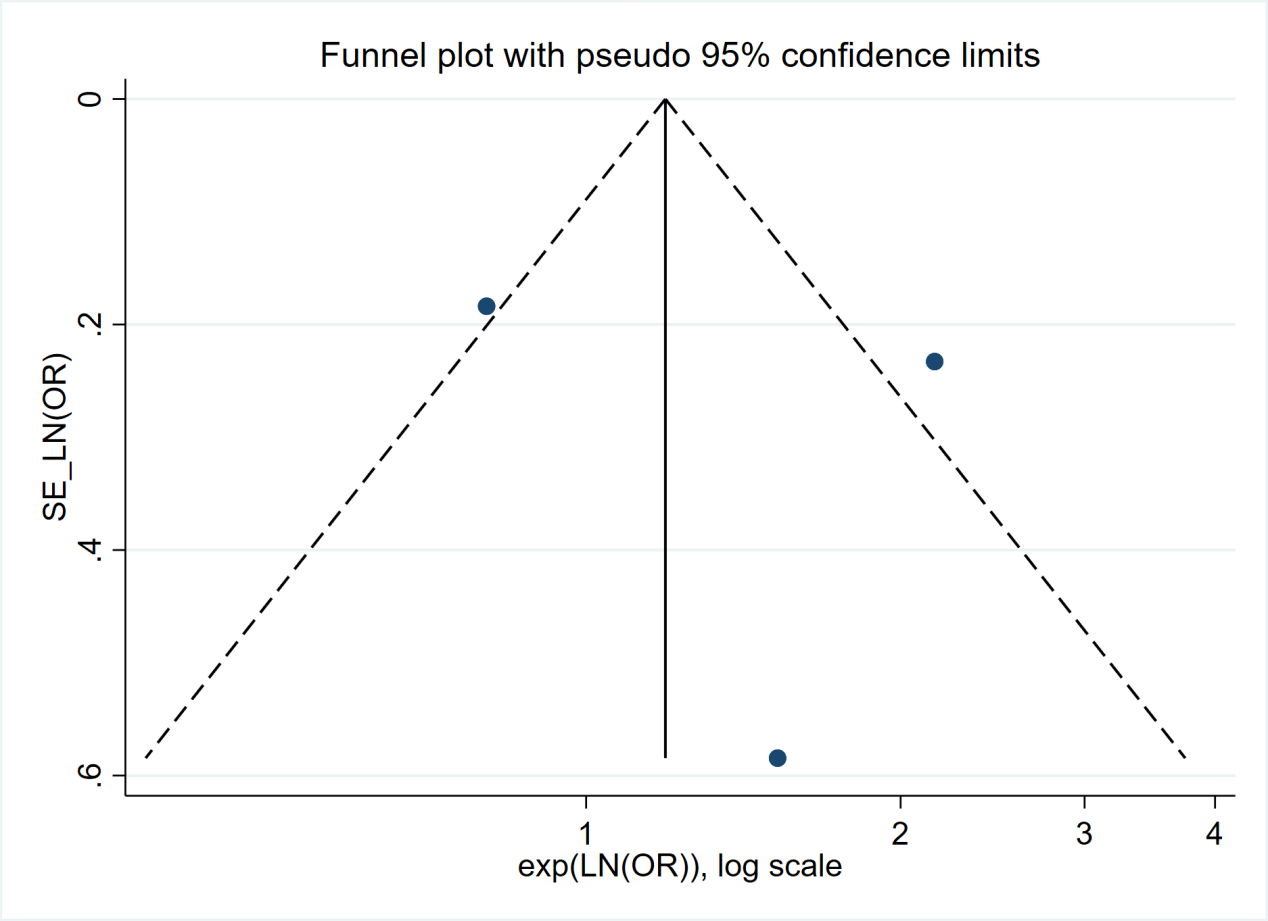
*

Figure S 63Funnel Plot of the Association Between Diuretic Use and Sarcopenia in Patients with CKD

*
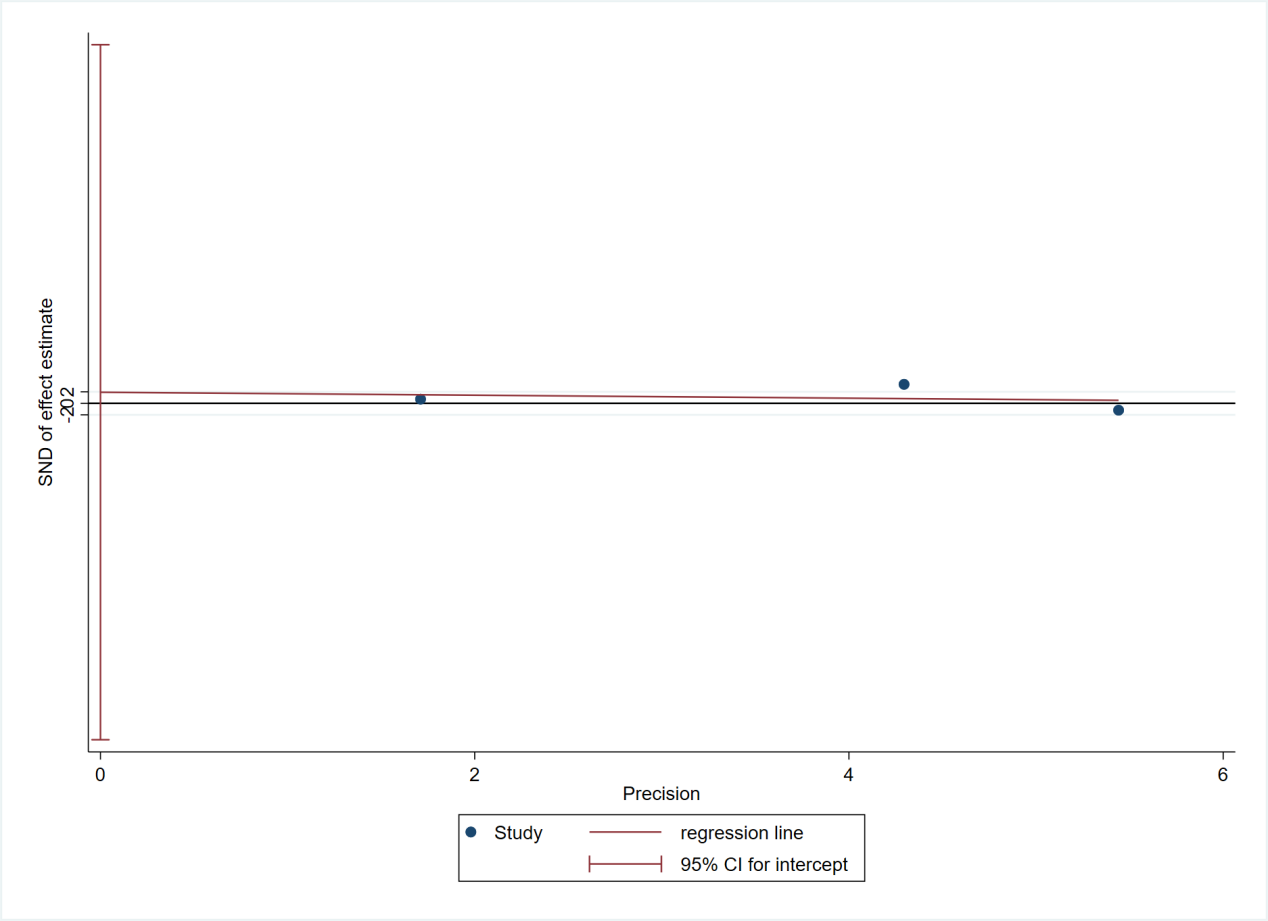
*

**

Figure S 64 Egger's Test of the Association Between Diuretic Use and Sarcopenia in Patients with CKD

*Results After Excluding Studies with High Heterogeneity in Sensitivity Analysis*

*In this meta-analysis, the initial analysis showed high overall heterogeneity (I² = 82.3% > 50%, P = 0.004 < 0.1). Sensitivity analysis revealed that one study Amorim (2022), contributed significantly to the heterogeneity. The uniqueness of this study lies in the fact that its geographical region is South America, which differs from the predominantly Asia-based populations in other studies, potentially affecting the estimation of its effect size. After excluding this study, heterogeneity was significantly reduced (I² = 0.0% < 50%, P = 0.582 > 0.1), and the pooled effect size became more robust. This suggests that geographical region may be the primary source of heterogeneity. Ultimately, we decided to retain this study and thoroughly discussed the impact of this decision on the conclusions in the report.*

*
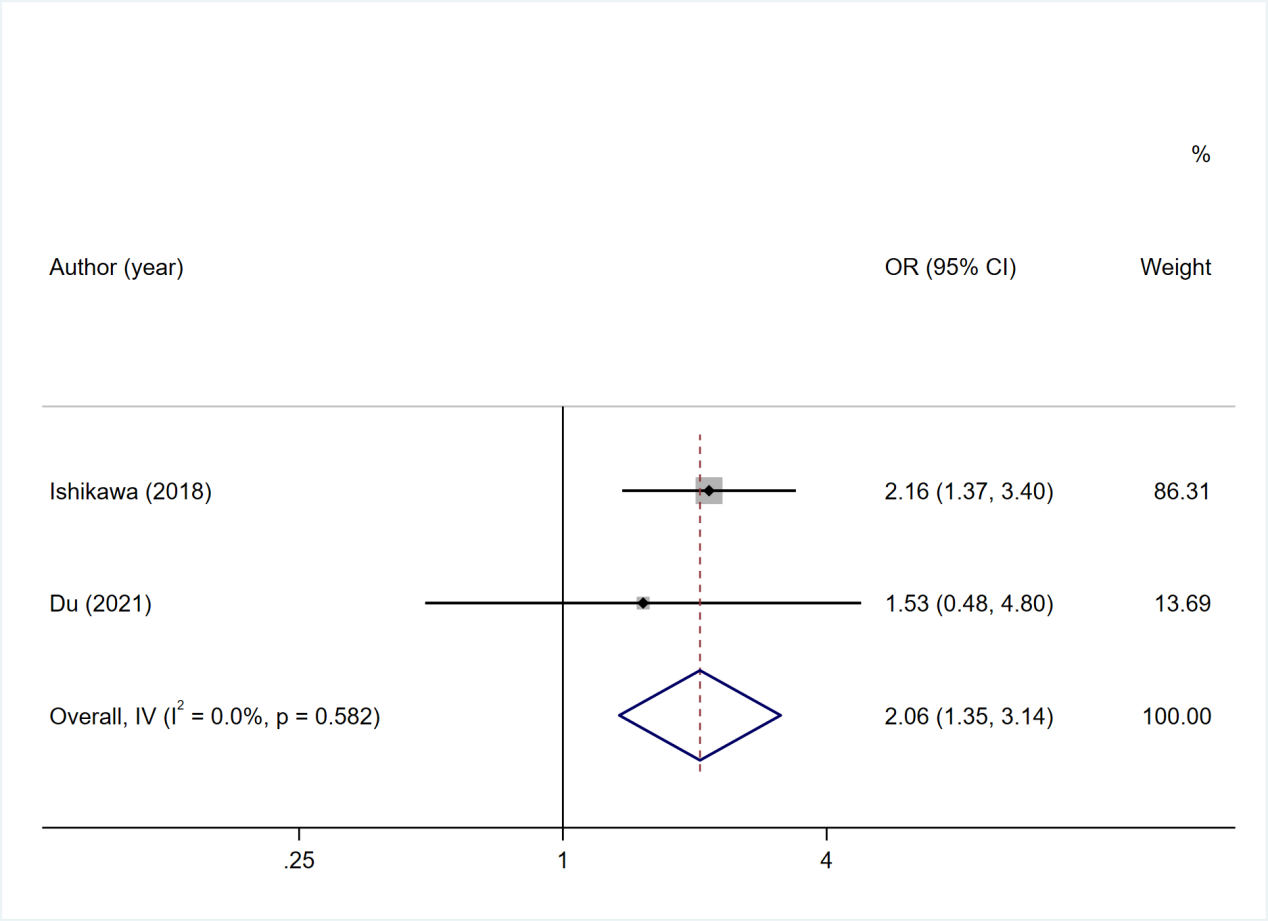
*

Figure S 65Forest Plot of the Association Between Diuretic Use and Sarcopenia in Patients with CKD (Excluding Amorim 2022)

**

Figure S 66Sensitivity Analysis of the Association Between Diuretic Use and Sarcopenia in Patients with CKD (Excluding Amorim 2022)

*
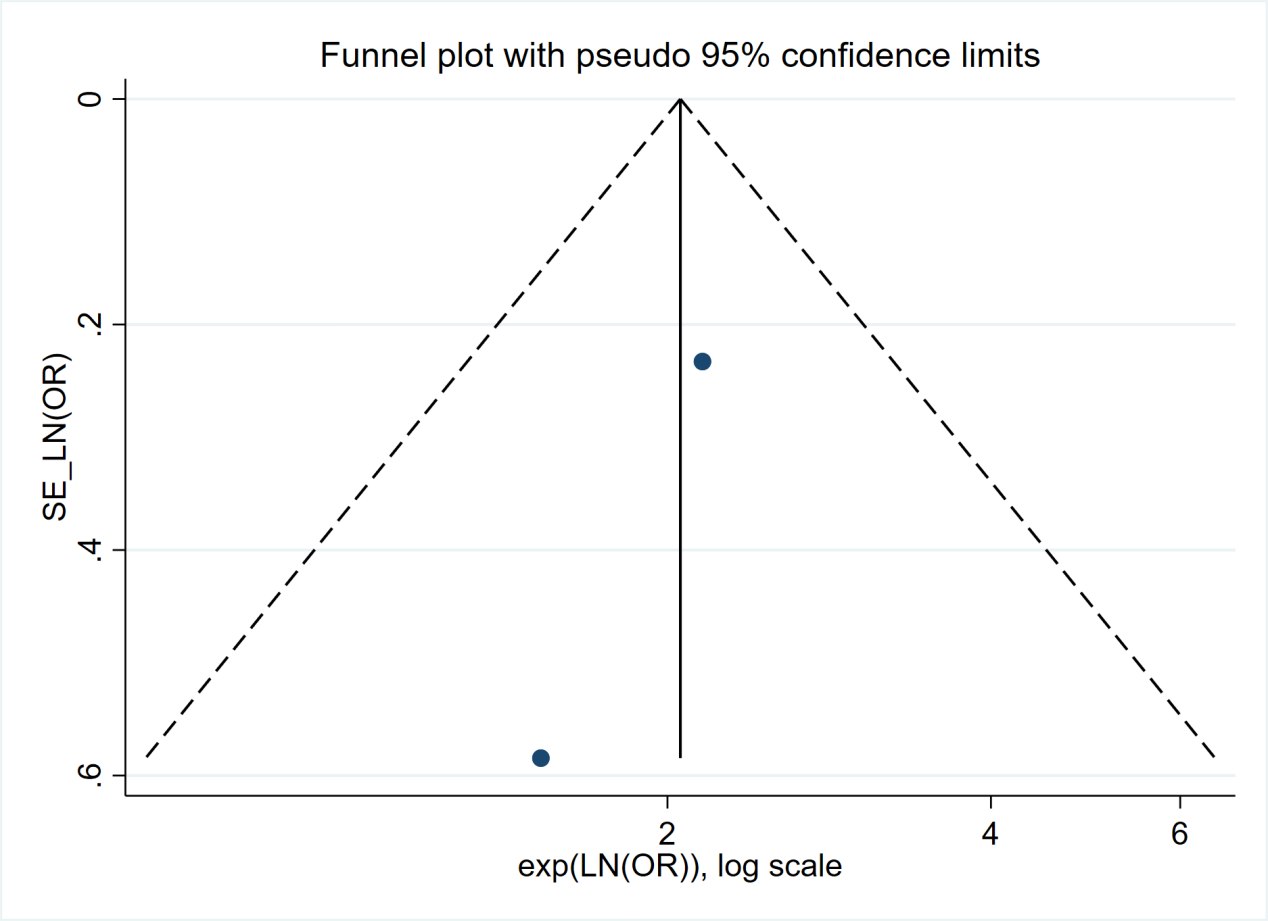
*

Figure S 67Funnel Plot of the Association Between Diuretic Use and Sarcopenia in Patients with CKD (Excluding Amorim 2022)

*Figure 37 Funnel Plot of the Association Between Diuretic Use and Sarcopenia in Patients with CKD (Excluding Amorim 2022)*

*
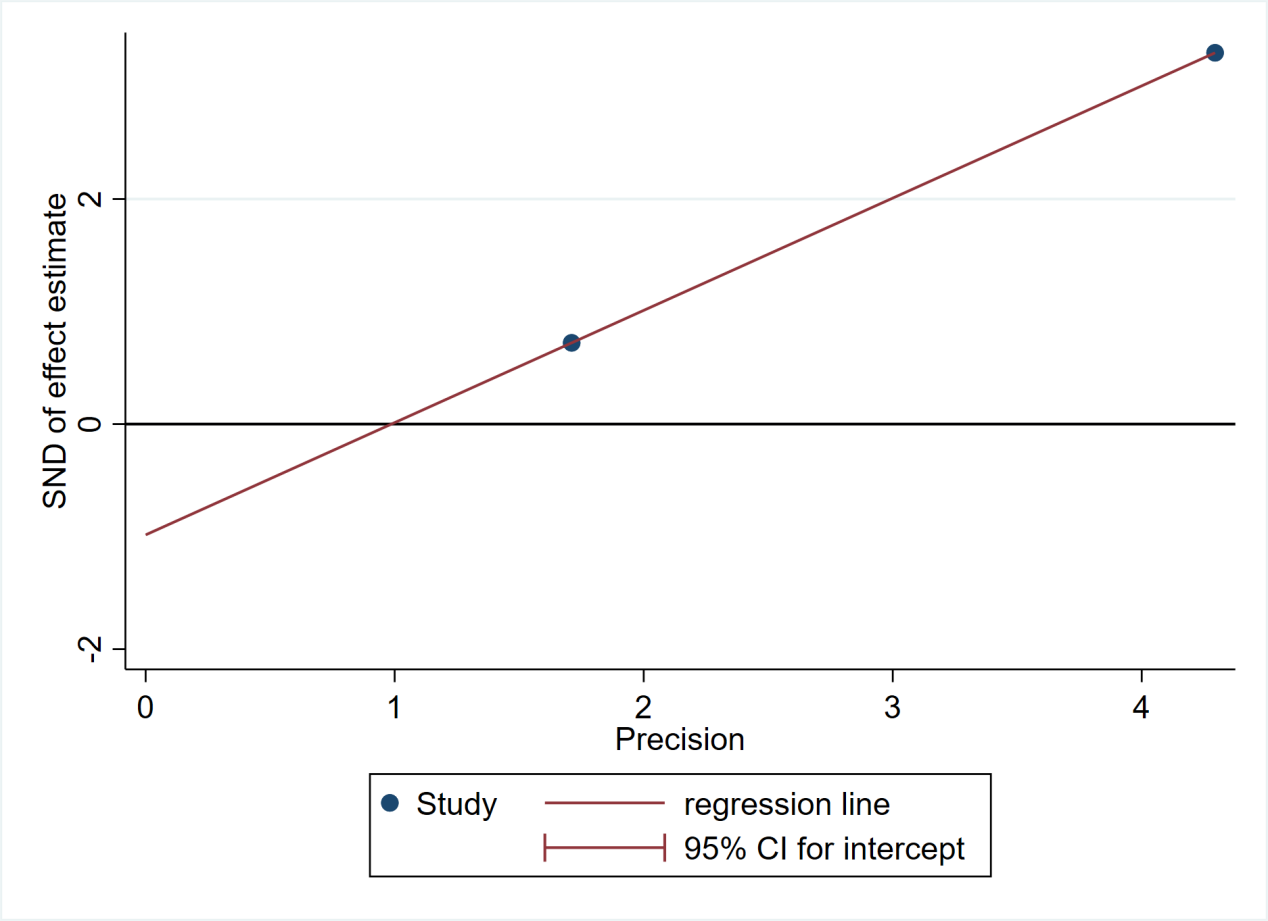
*

**

Figure S 68Egger's Test of the Association Between Diuretic Use and Sarcopenia in Patients with CKD (Excluding Amorim 2022)

*eGFR(estimated Glomerular Filtration Rate)*

Figure S 69 Sensitivity Analysis of the Association Between eGFR and Sarcopenia in Patients with CKD

*
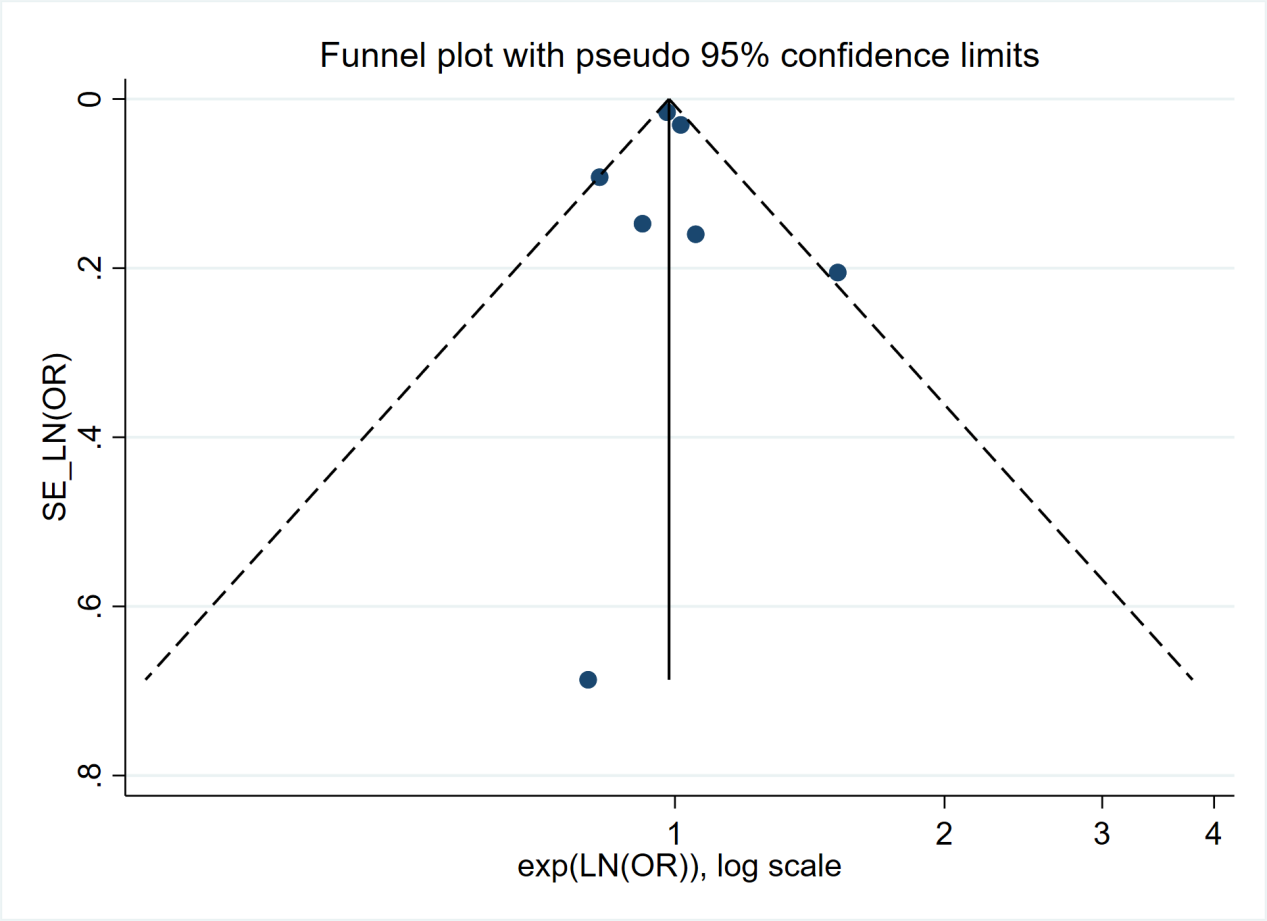
*

Figure S 70Funnel Plot of the Association Between eGFR and Sarcopenia in Patients with CKD

*
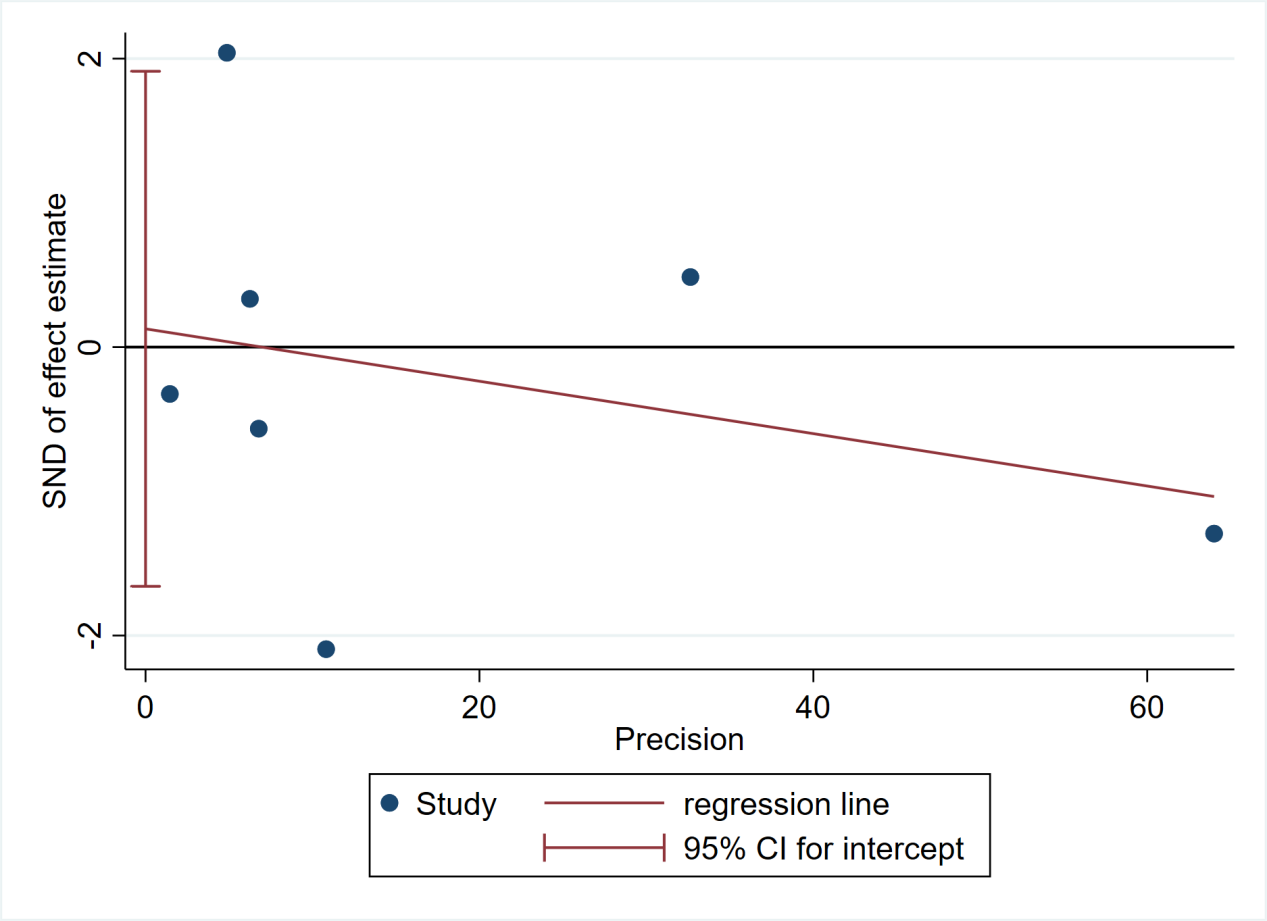
*

*
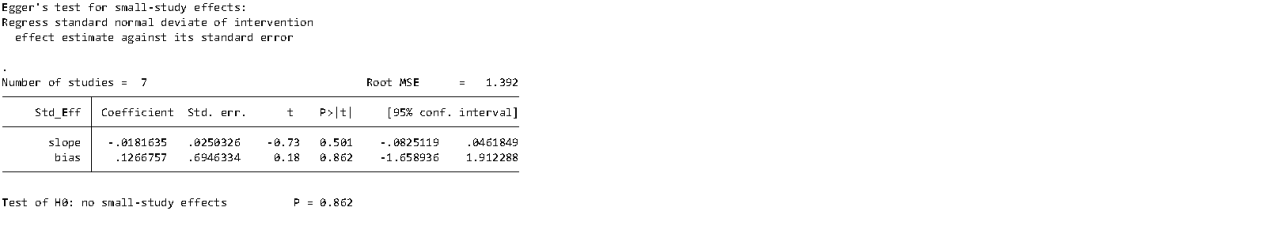
*

Figure S 71Egger's Test of the Association Between eGFR and Sarcopenia in Patients with CKD


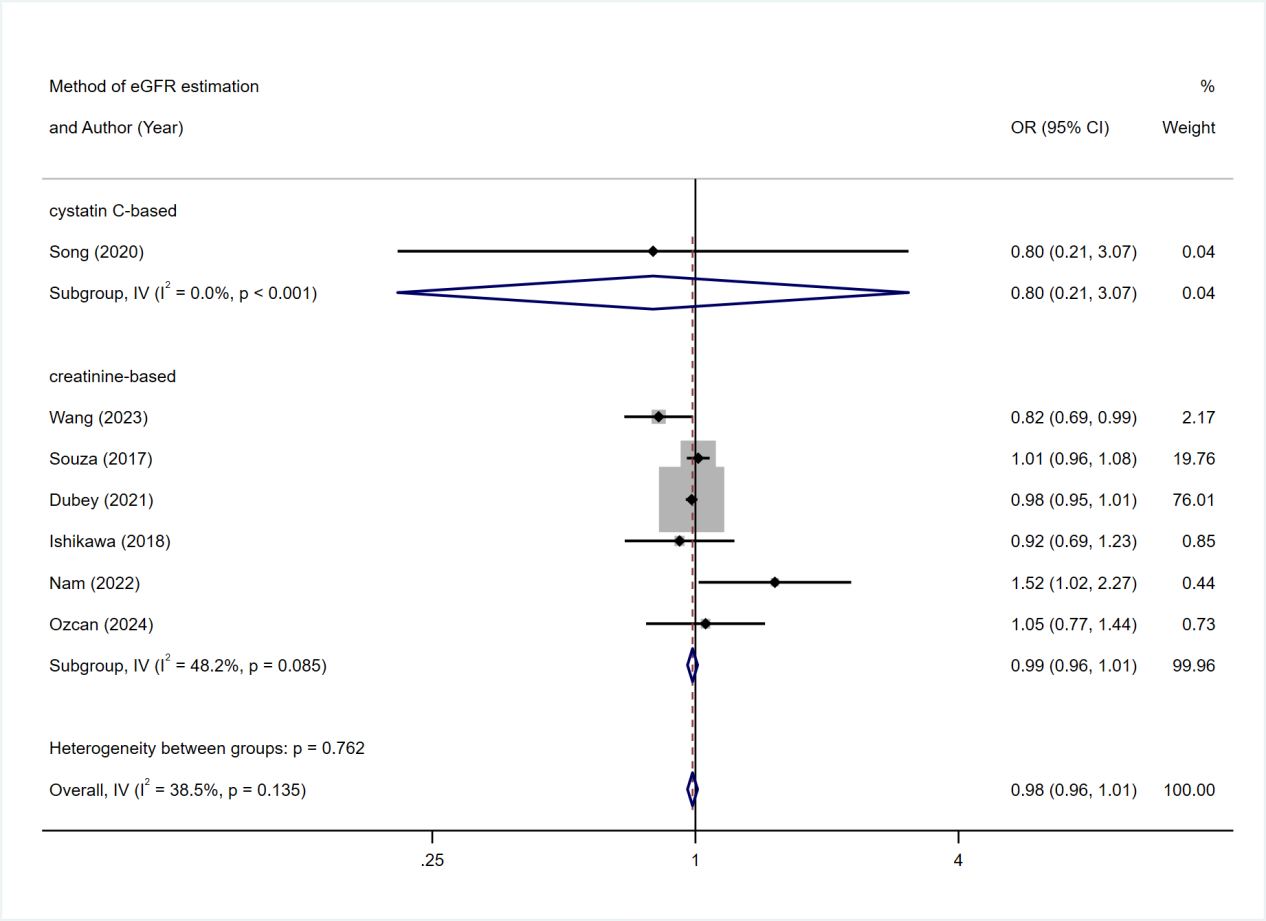


Figure S 72Forest Plot of the Subgroup Analysis of the Association Between eGFR and Sarcopenia in Patients with CKD Based on the eGFR estimation

*Female vs male*

Figure S 73Sensitivity Analysis of the Association Between Female and Sarcopenia in Patients with CKD

*
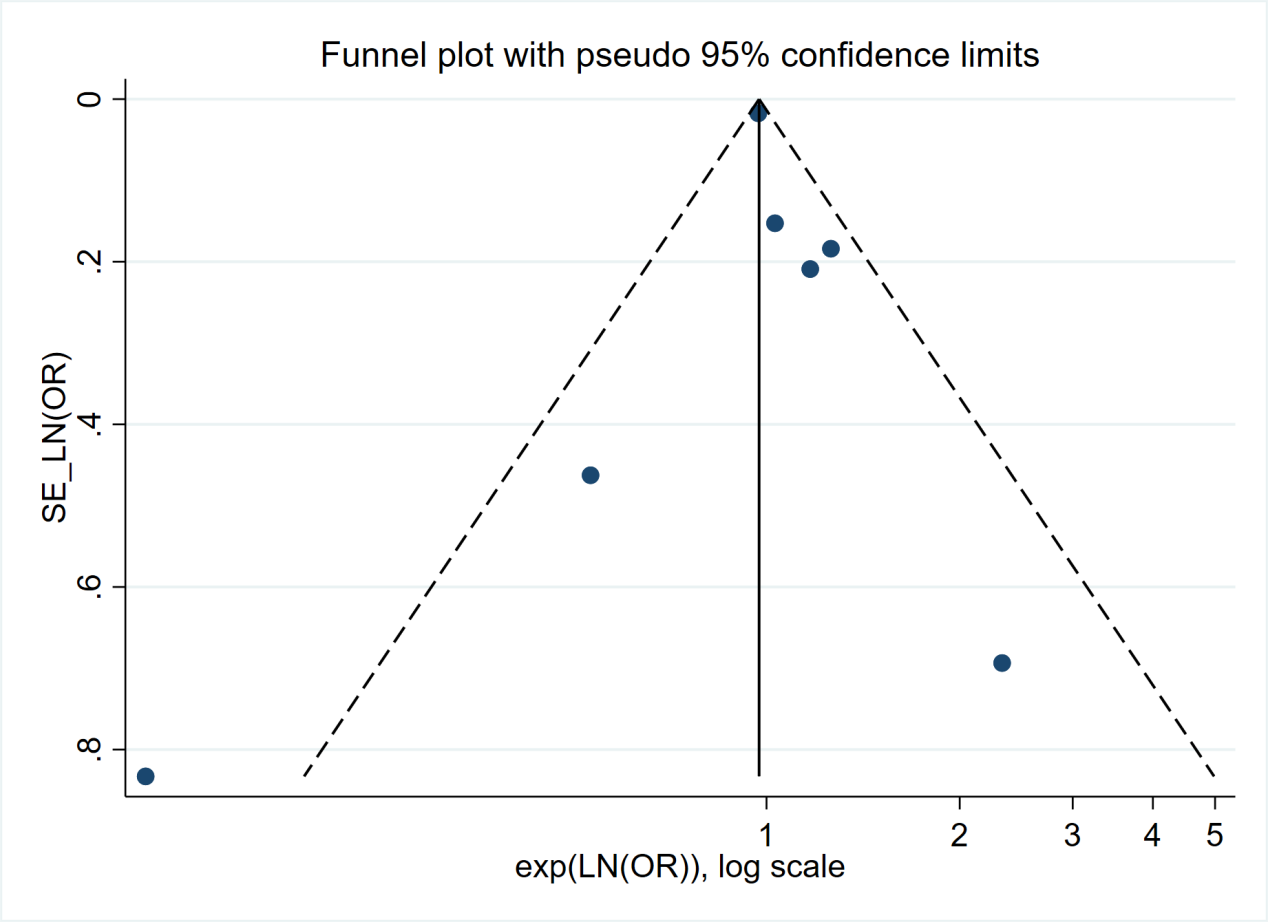
*

Figure S 74Funnel Plot of the Association Between Female and Sarcopenia in Patients with CKD

*
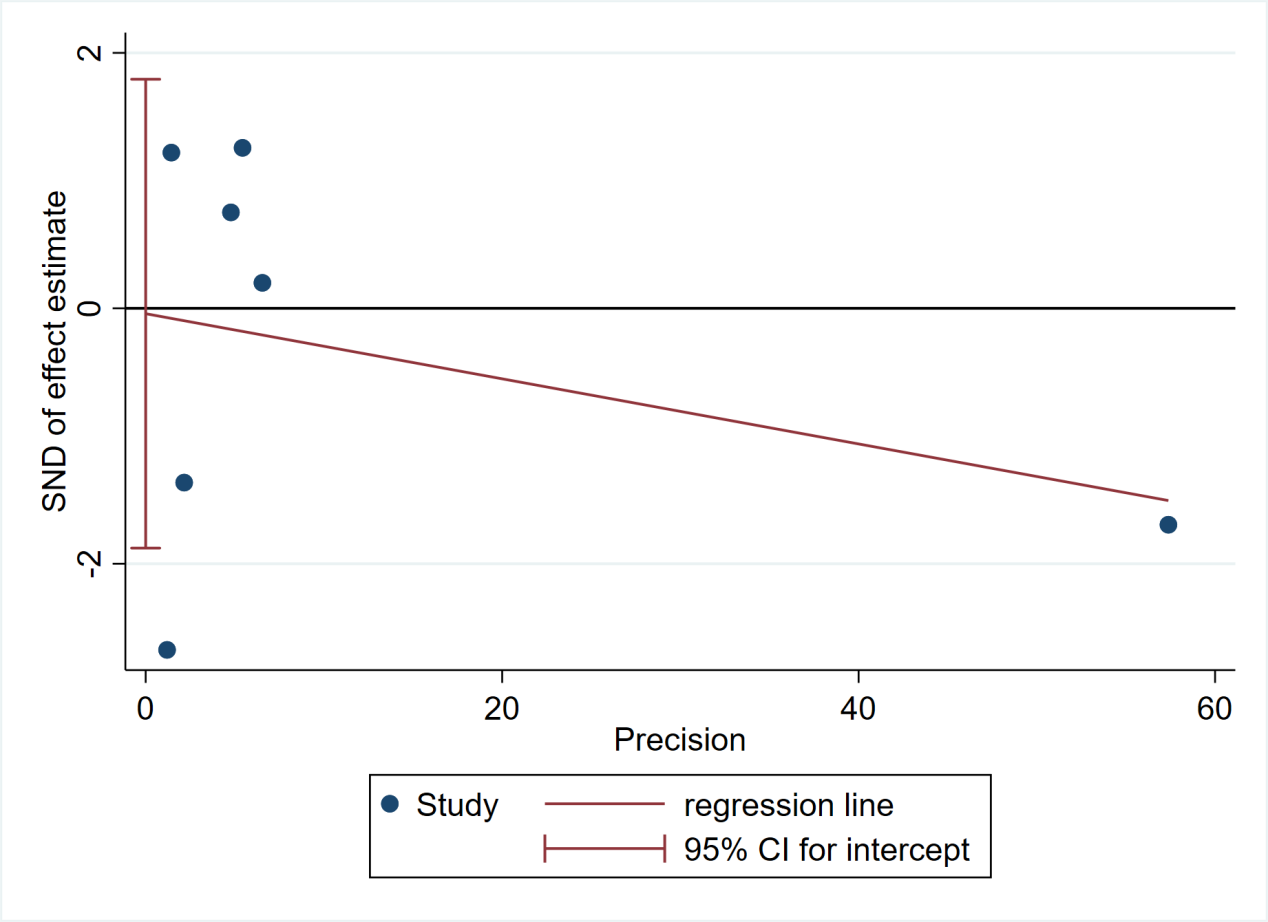
*

*
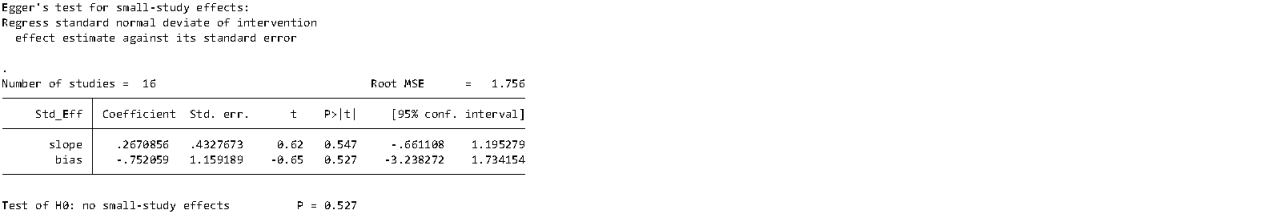
*

Figure S 75Egger's Test of the Association Between Female and Sarcopenia in Patients with CKD

*HDL(High-Density Lipoprotein)*

Figure S 76 Sensitivity Analysis of the Association Between HDL and Sarcopenia in Patients with CKD

*
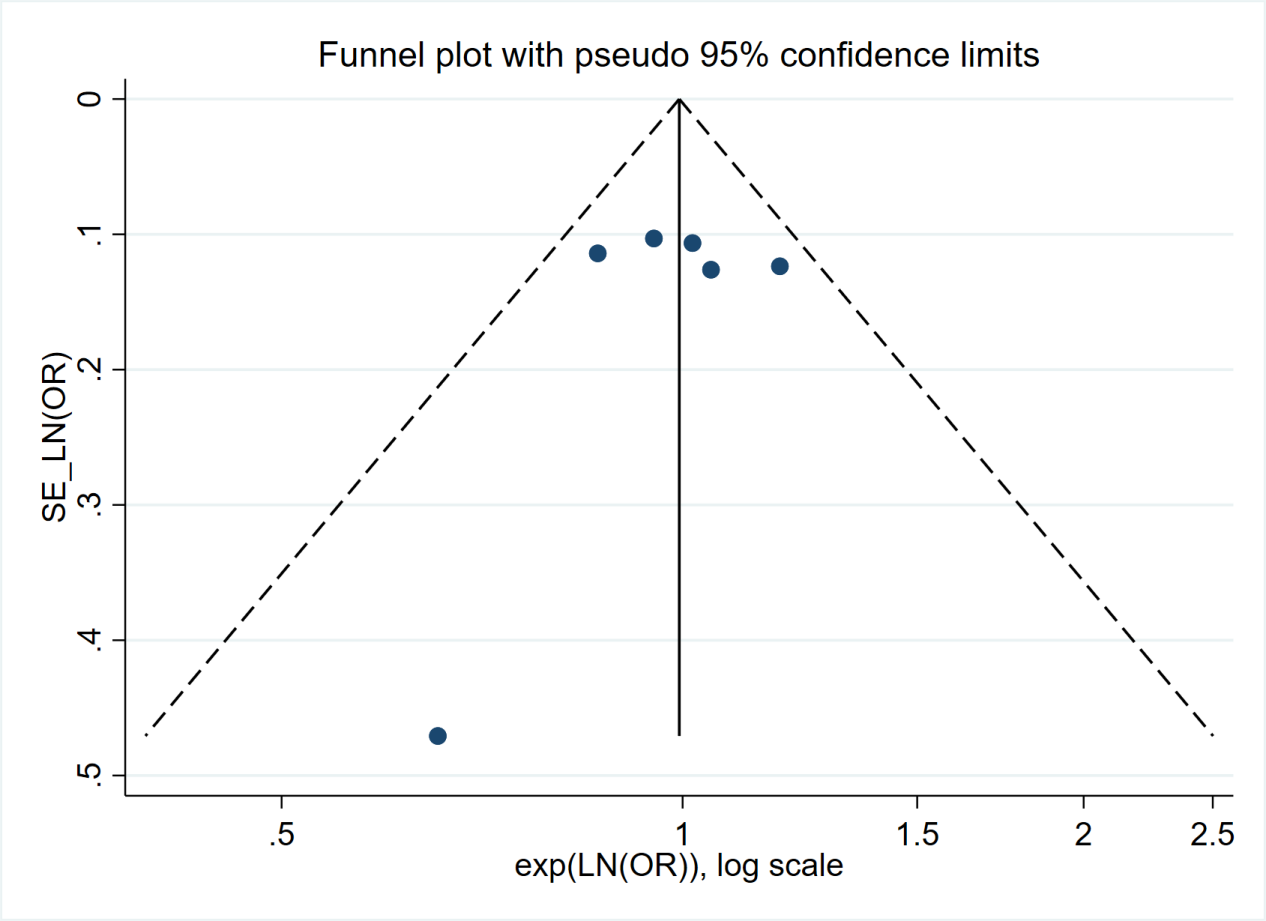
*

Figure S 77Funnel Plot of the Association Between HDL and Sarcopenia in Patients with CKD

Figure S 78Egger's Test of the Association Between HDL and Sarcopenia in Patients with CKD

*Figure 47 Egger's Test of the Association Between HDL and Sarcopenia in Patients with CKD*

*Hemoglobin*

Figure S 79Sensitivity Analysis of the Association Between Hemoglobin and Sarcopenia in Patients with CKD

Figure S 80Funnel Plot of the Association Between Hemoglobin and Sarcopenia in Patients with CKD

Figure S 81Egger's Test of the Association Between Hemoglobin and Sarcopenia in Patients with CKD

*History of cardiovascular disease*

**

Figure S 82Sensitivity Analysis of the Association Between History of Cardiovascular Disease and Sarcopenia in Patients with CKD

*
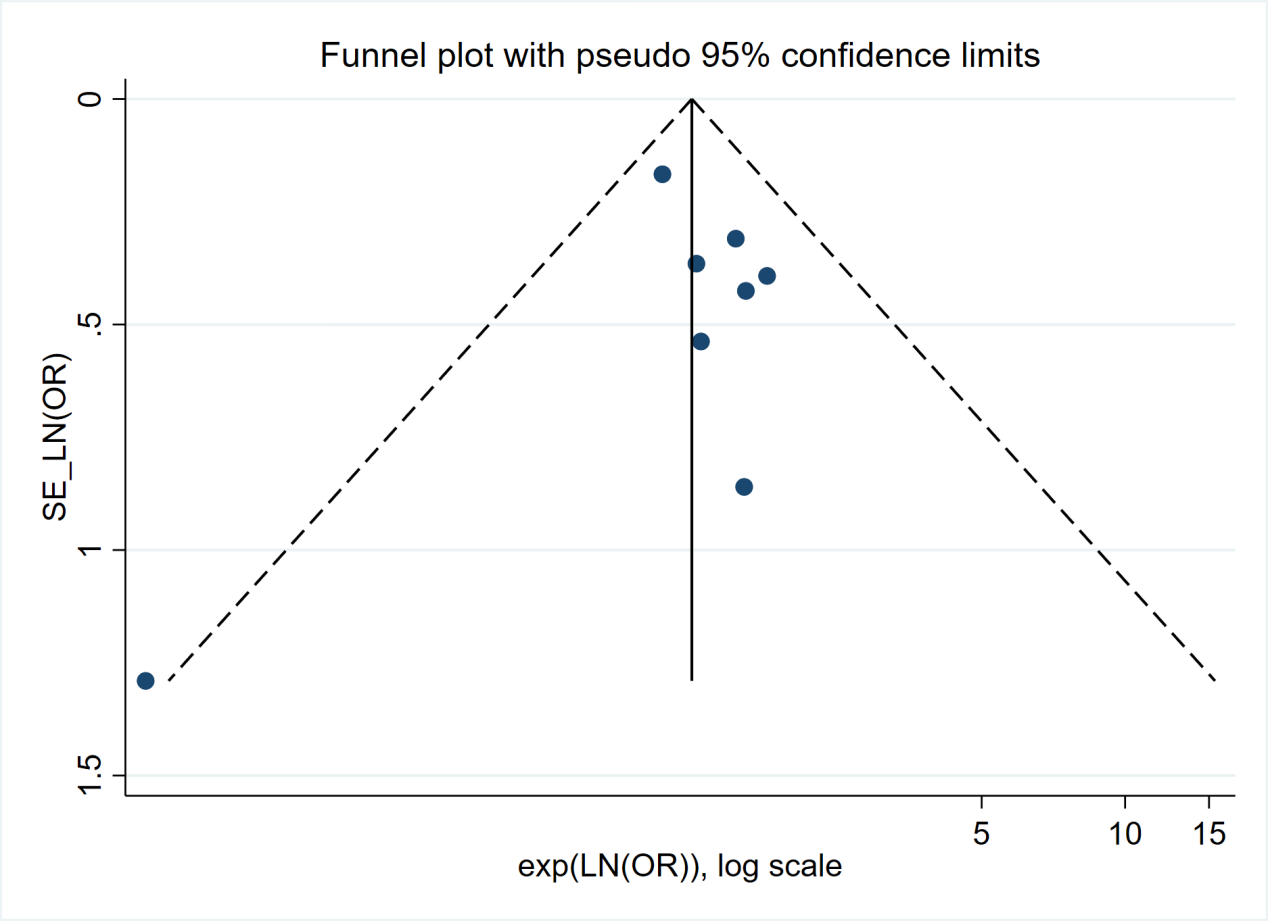
*

Figure S 83Funnel Plot of the Association Between History of Cardiovascular Disease and Sarcopenia in Patients with CKD

*Figure 52 Funnel Plot of the Association Between History of Cardiovascular Disease and Sarcopenia in Patients with CKD*

*
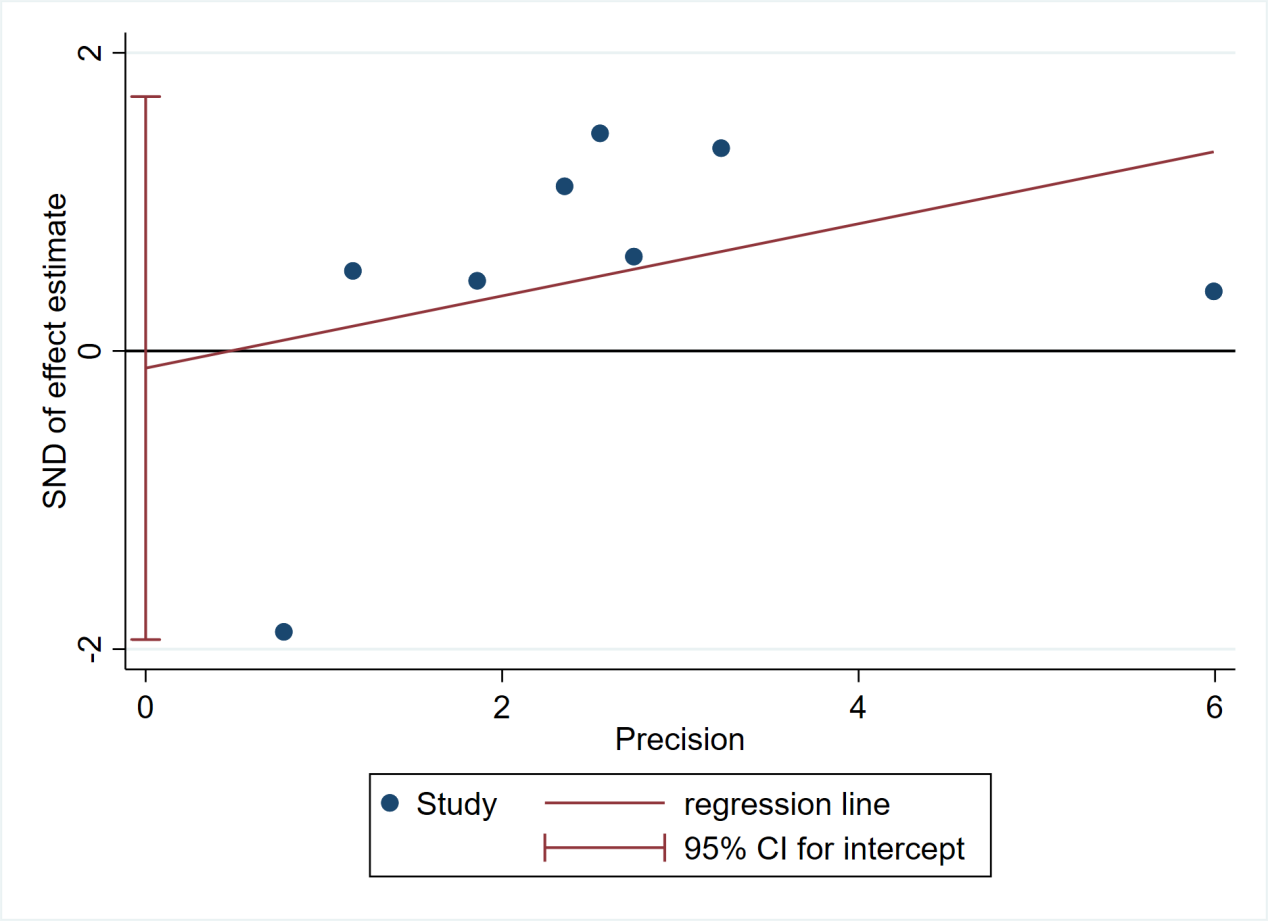
*

**

Figure S 84Egger's Test of the Association Between History of Cardiovascular Disease and Sarcopenia in Patients with CKD

*Hypertension*

Figure S 85Sensitivity Analysis of the Association Between Hypertension and Sarcopenia in Patients with CKD

*
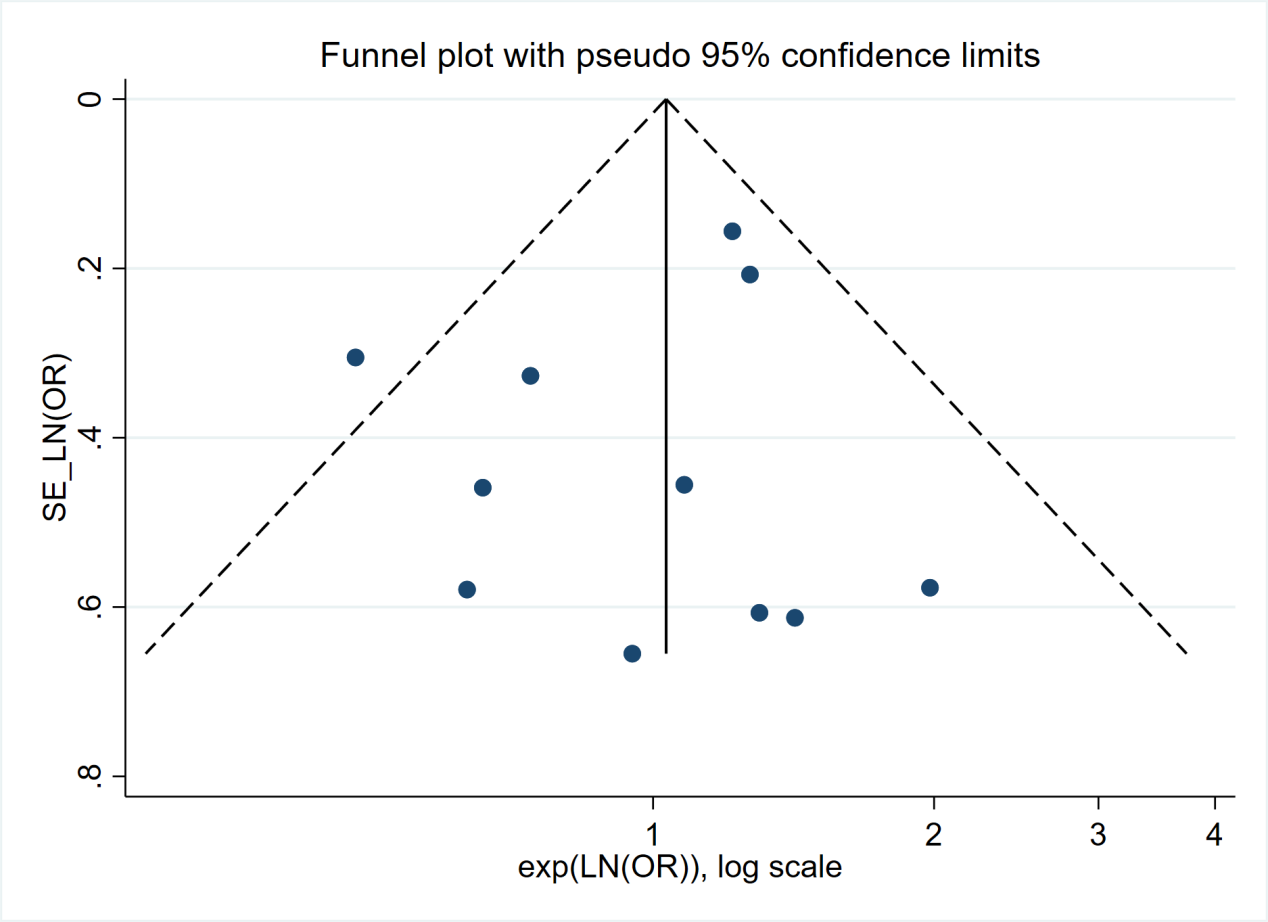
*

Figure S 86Funnel Plot of the Association Between Hypertension and Sarcopenia in Patients with CKD

*Figure 55 Funnel Plot of the Association Between Hypertension and Sarcopenia in Patients with CKD*

*
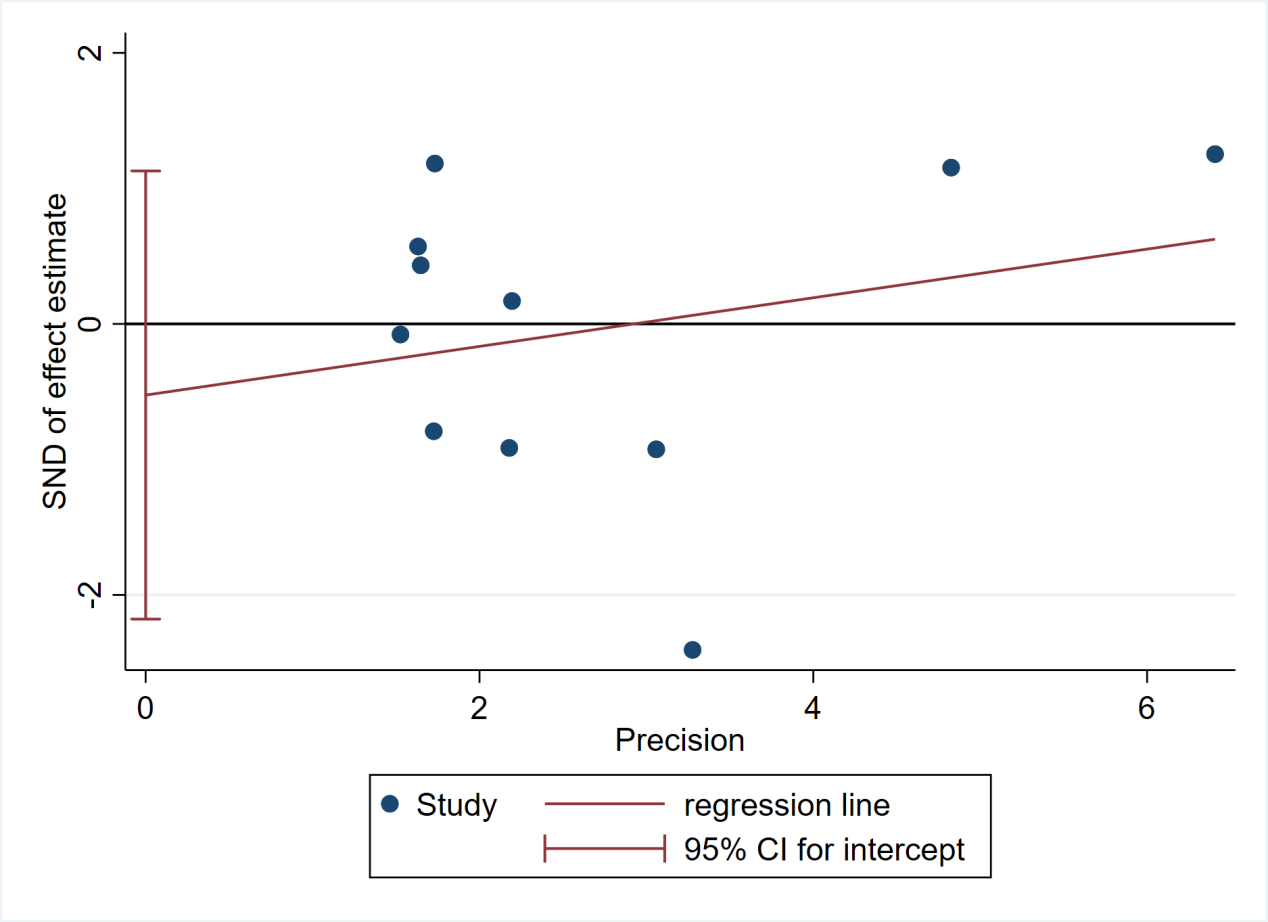
*

Figure S 87Egger's Test of the Association Between Hypertension and Sarcopenia in Patients with CKD

*PAB(Prealbumin)*

**

Figure S 88Sensitivity Analysis of the Association Between PAB and Sarcopenia in Patients with CKD

*Figure 57 Sensitivity Analysis of the Association Between PAB and Sarcopenia in Patients with CKD*

*
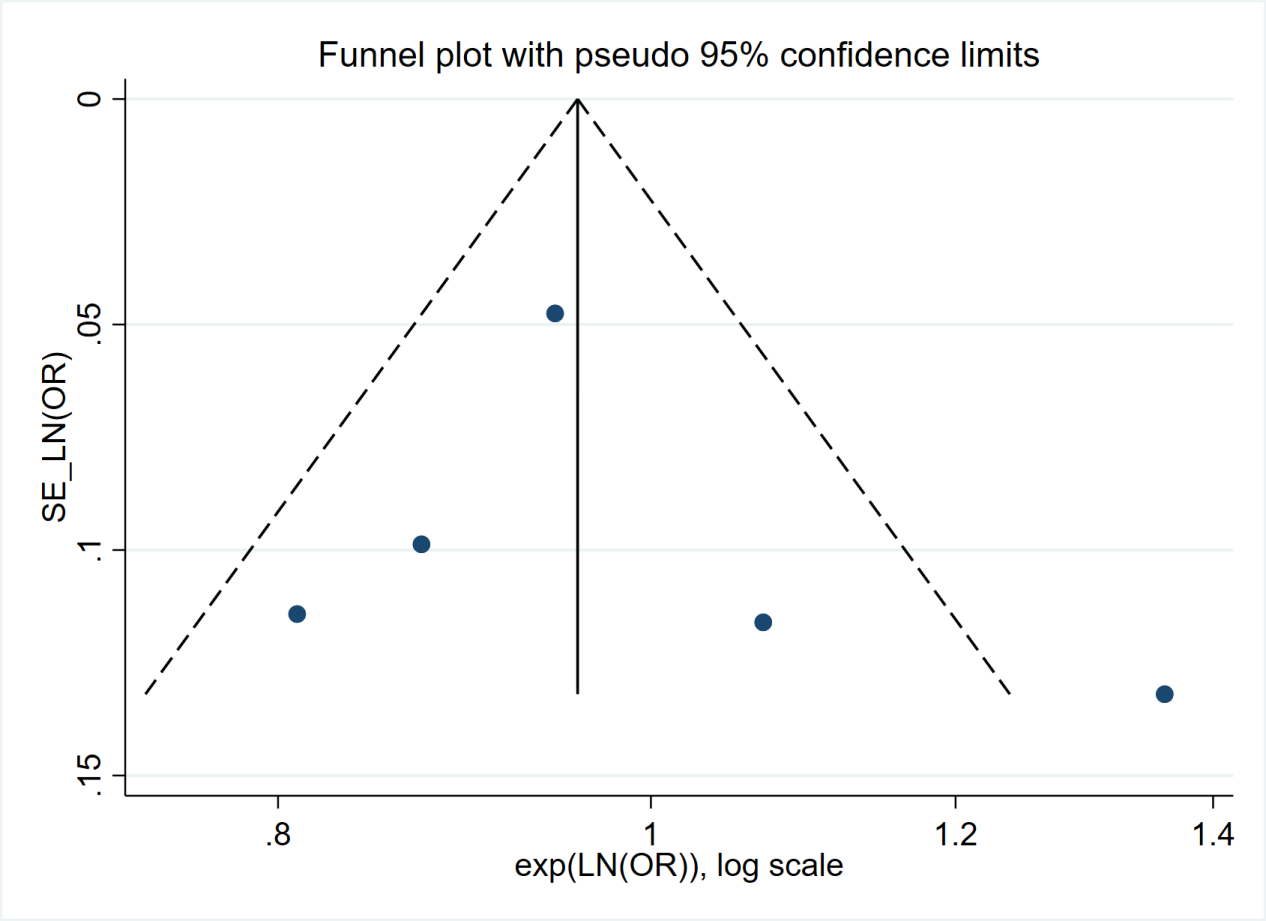
*

Figure S 89Funnel Plot of the Association Between PAB and Sarcopenia in Patients with CKD

*
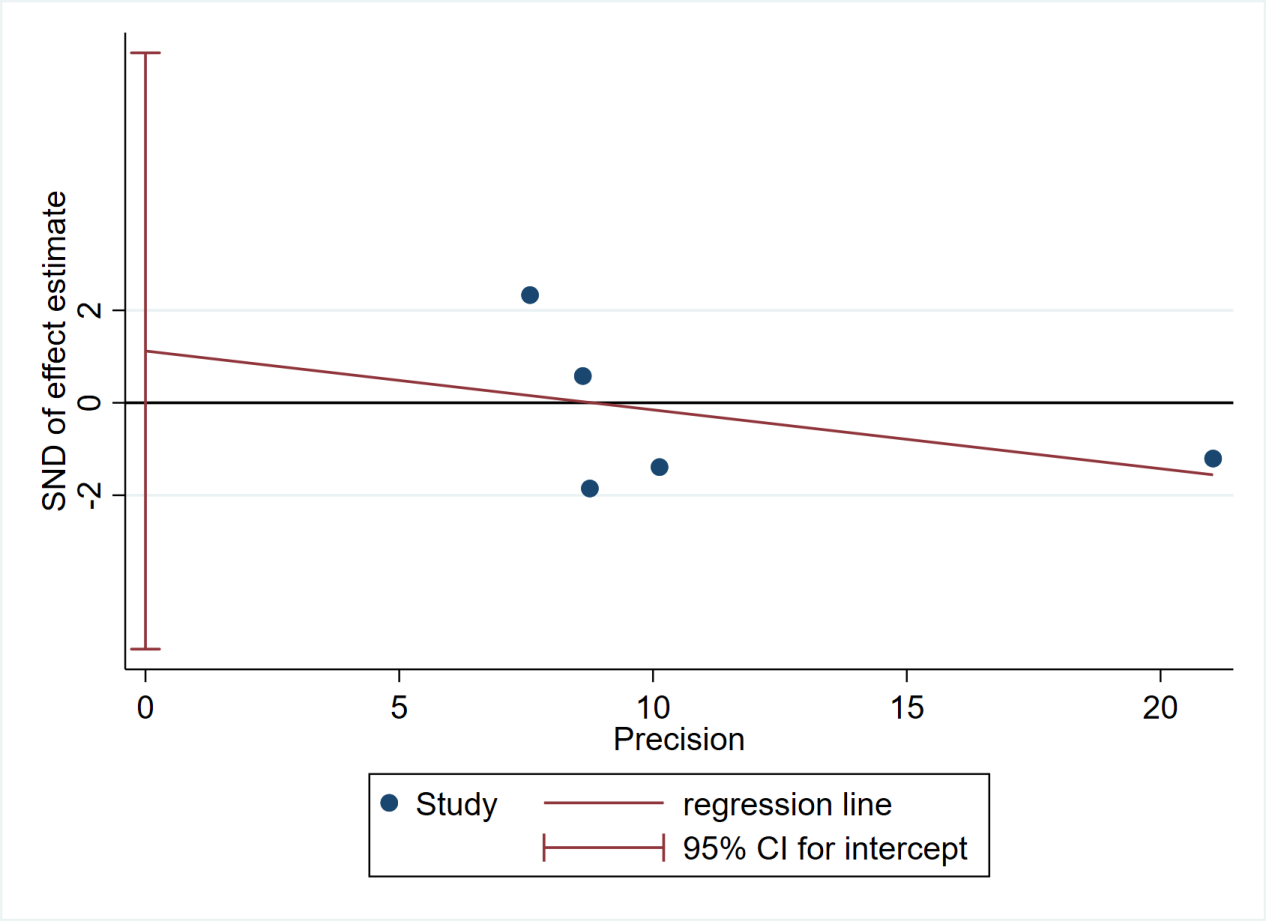
*

**

Figure S 90Egger's Test of the Association Between PAB and Sarcopenia in Patients with CKD

*Figure 59 Egger's Test of the Association Between PAB and Sarcopenia in Patients with CKD*

*Results After Excluding Studies with High Heterogeneity in Sensitivity Analysis*

*In this meta-analysis, the initial analysis revealed substantial heterogeneity (I² = 64.1% > 50%, P = 0.025 < 0.1). Sensitivity analysis identified that the study by Chen (2024) contributed significantly to this heterogeneity. The distinguishing factor of this study is that the OR values were calculated using a different algorithm, with a data format that could not be uniformly merged. The OR values were likely derived from a specific statistical model incorporating multiple covariates or adjustment factors, which led to discrepancies compared to the effect sizes calculated through standard SMD conversion. This difference in methodology from other studies likely influenced the effect size estimation. After excluding this study, the heterogeneity dropped significantly (I² = 13.5% < 50%, P = 0.325 > 0.1), and the pooled effect size became more robust. Therefore, it is hypothesized that the model used to calculate the OR values may be the primary source of heterogeneity. In the end, we decided to retain this study and fully discuss its impact on the conclusions.*

*
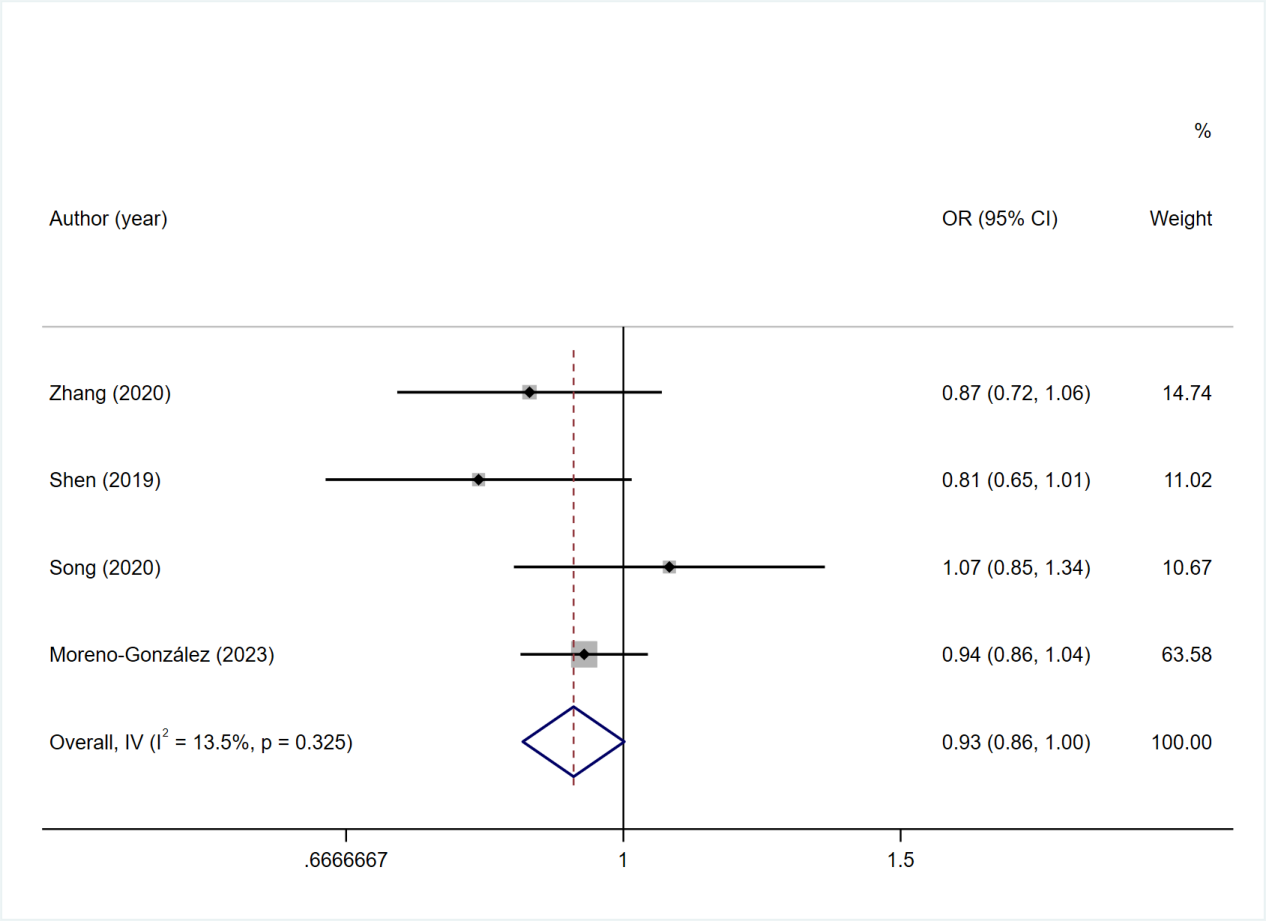
*

Figure S 91Forest Plot of the Association Between PAB and Sarcopenia in Patients with CKD (Excluding Chen 2024)

**

Figure S 92Sensitivity Analysis of the Association Between PAB and Sarcopenia in Patients with CKD (Excluding Chen 2024)

*
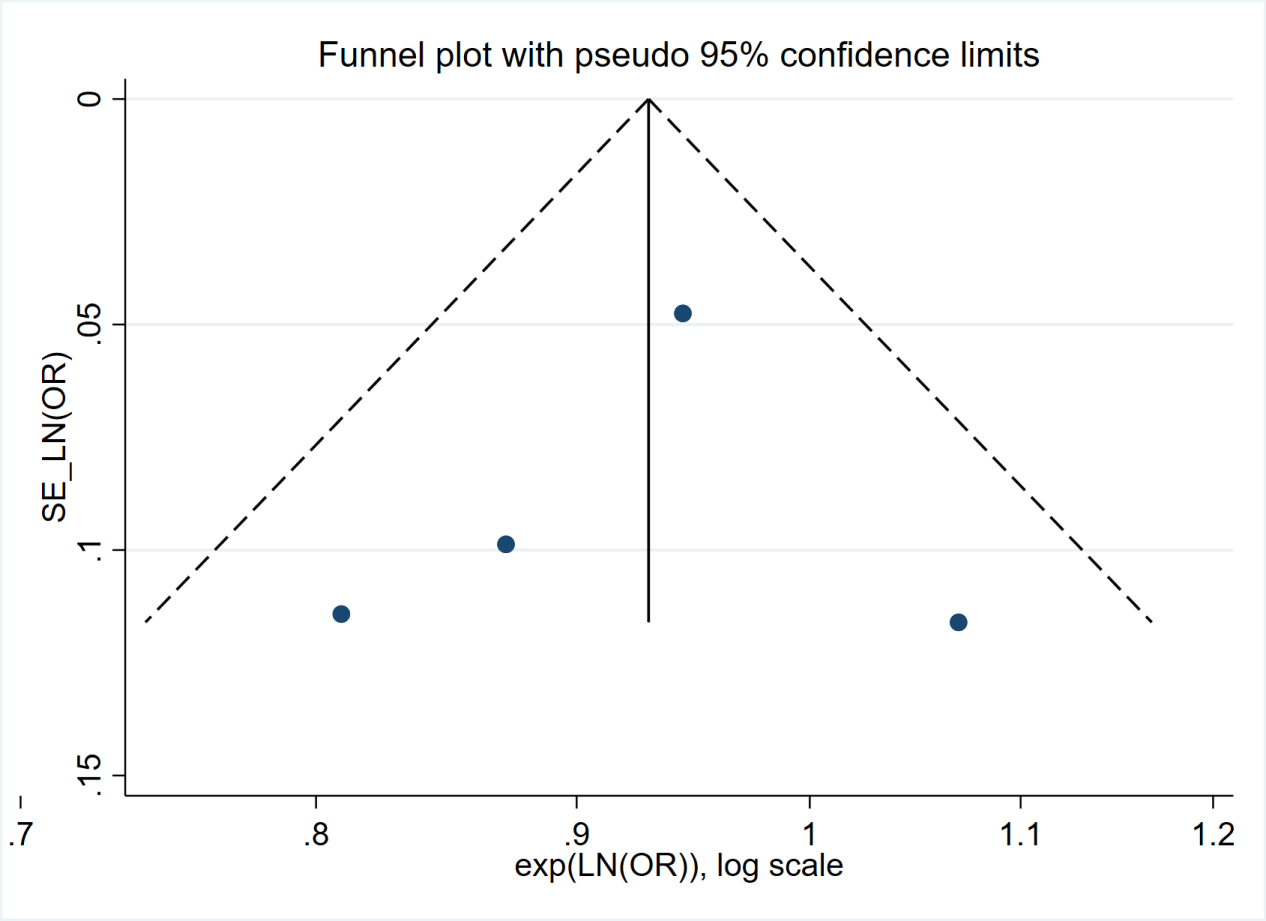
*

Figure S 93Funnel Plot of the Association Between PAB and Sarcopenia in Patients with CKD (Excluding Chen 2024)

*
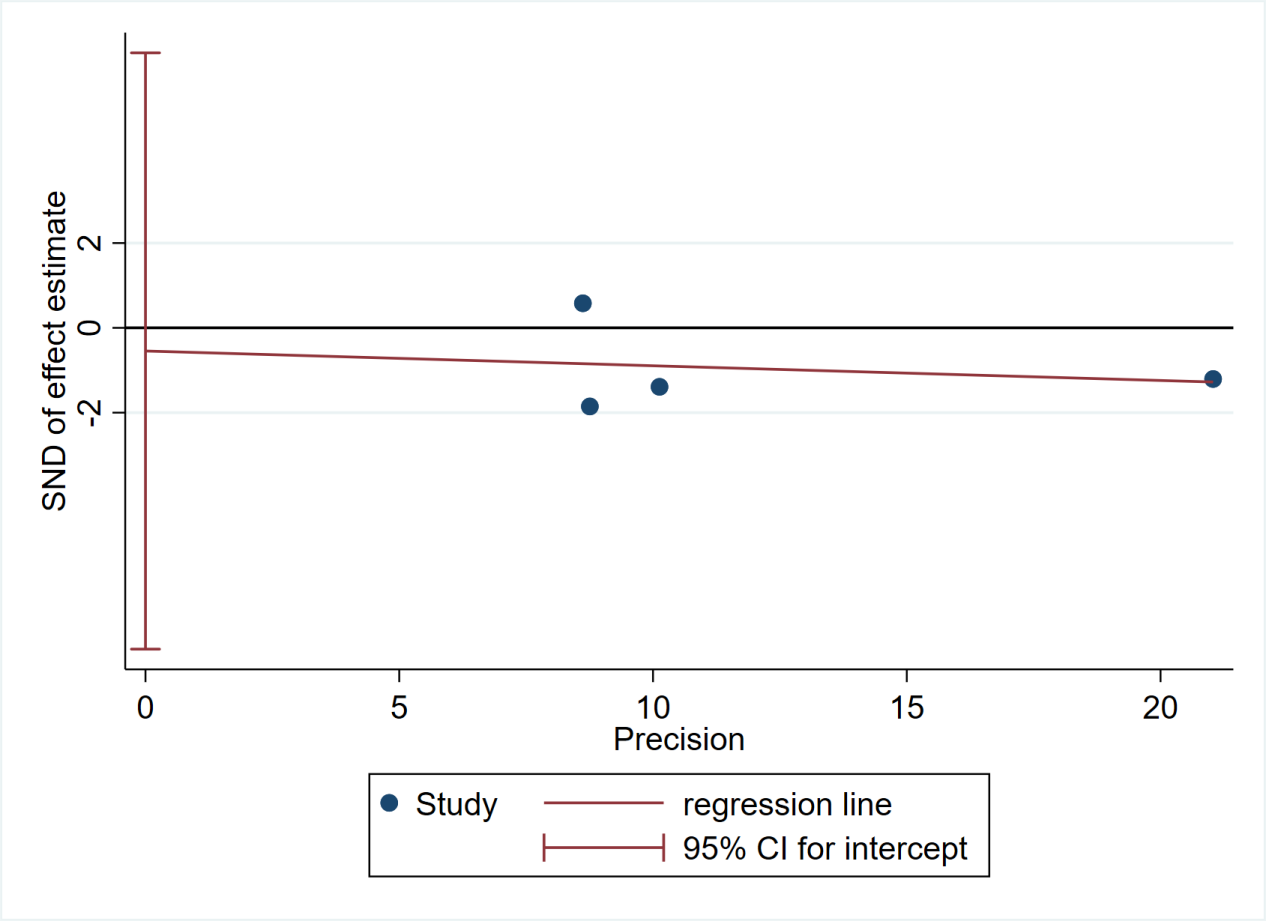
*

**

Figure S 94Egger's Test of the Association Between PAB and Sarcopenia in Patients with CKD (Excluding Chen 2024)

*PhA(Phase Angle)*

**

Figure S 95Sensitivity Analysis of the Association Between PhA and Sarcopenia in Patients with CKD

*
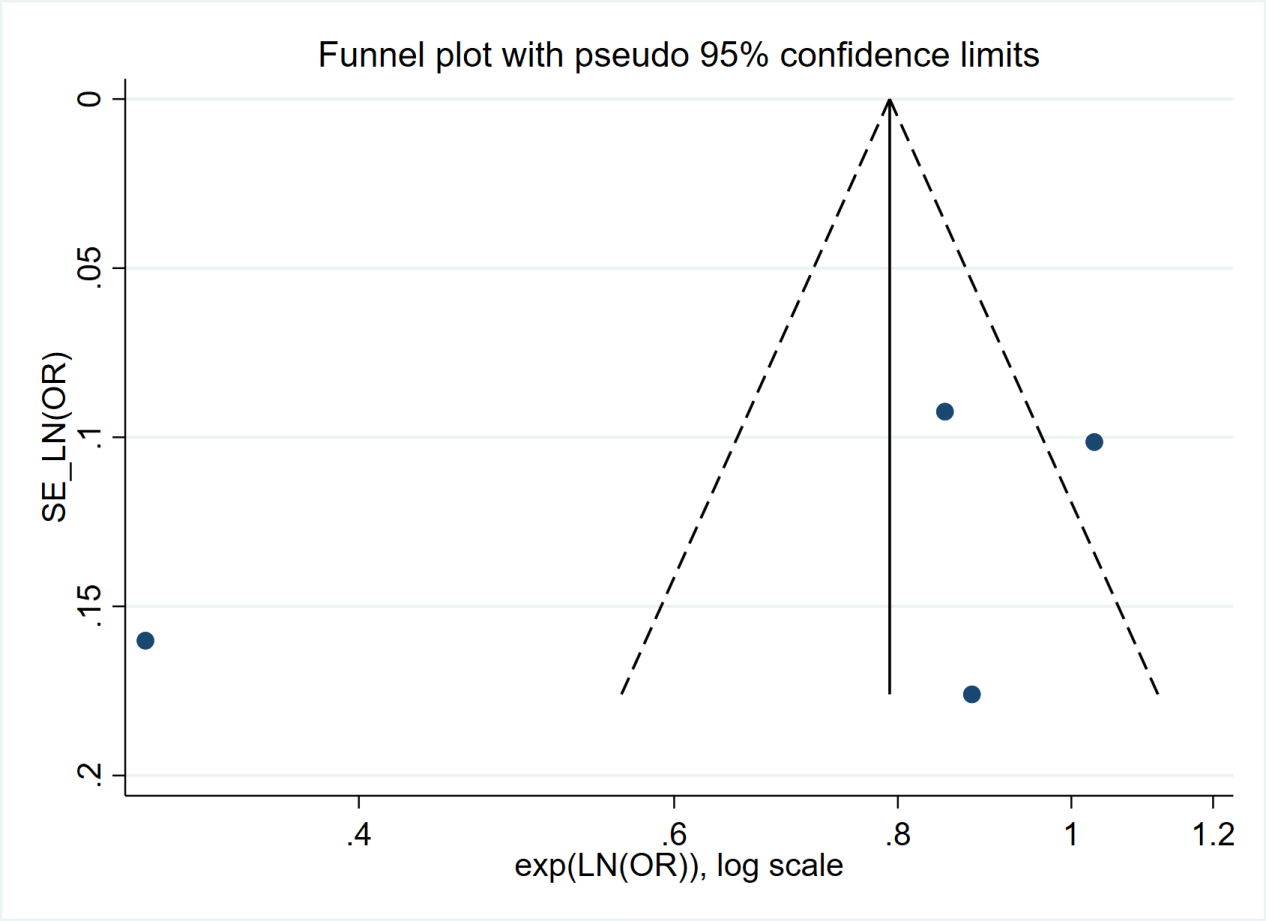
*

Figure S 96Funnel Plot of the Association Between PhA and Sarcopenia in Patients with CKD

*
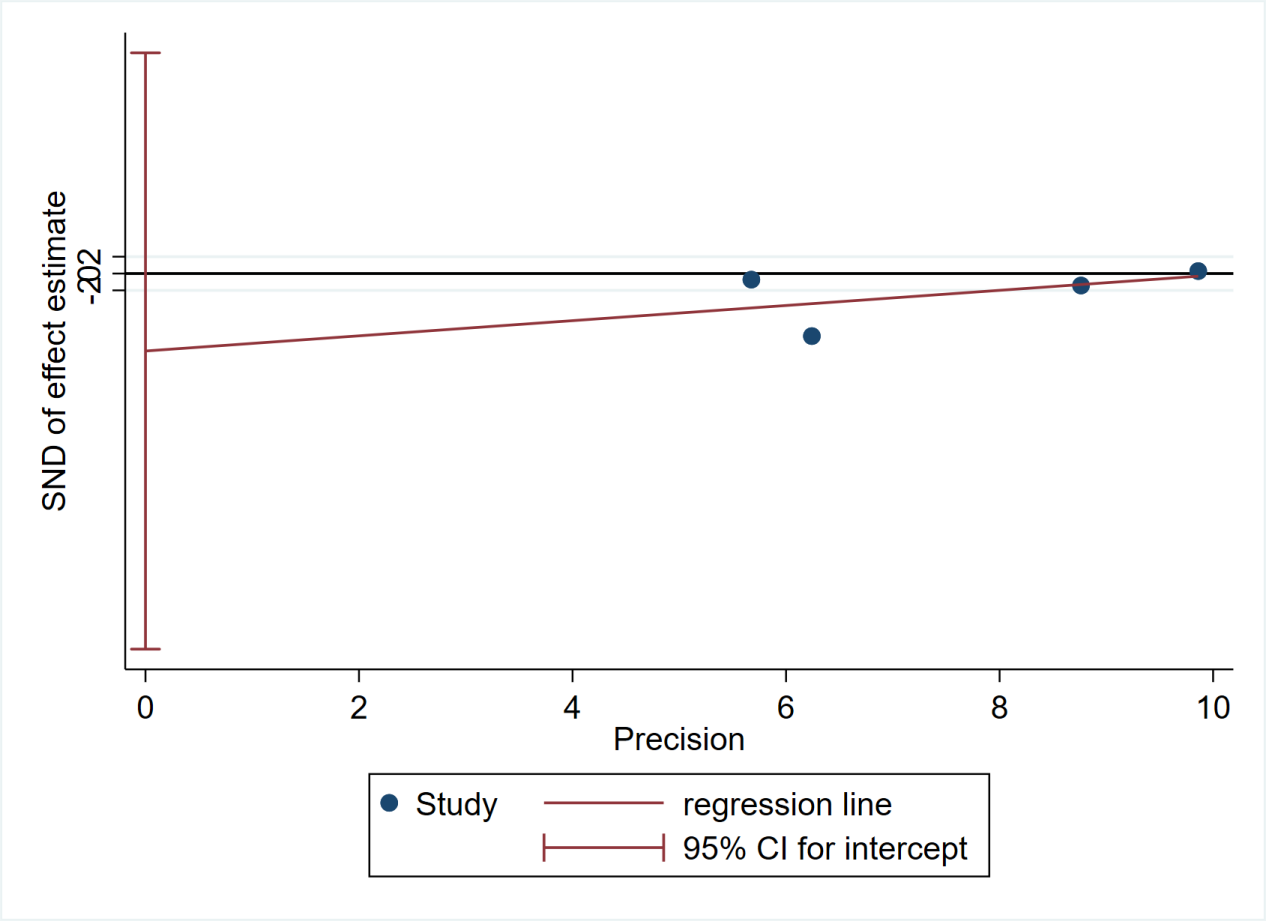
*

**

Figure S 97Egger's Test of the Association Between PhA and Sarcopenia in Patients with CKD

*Results After Excluding Studies with High Heterogeneity in Sensitivity Analysis*

*In this meta-analysis, the initial heterogeneity test revealed significant heterogeneity (I² = 93.1%). To identify the source of this heterogeneity, we conducted a sensitivity analysis by systematically excluding each study. The results showed that heterogeneity dropped significantly to I² = 1.8% after excluding the study by Amorim (2022). Further analysis indicated that Amorim (2022) was the only study focused on non-dialysis chronic kidney disease (ND-CKD) patients, whereas the other studies involved dialysis patients or other types of kidney disease. Therefore, it can be inferred that the specific population in Amorim (2022) is the primary source of the observed heterogeneity.*

*
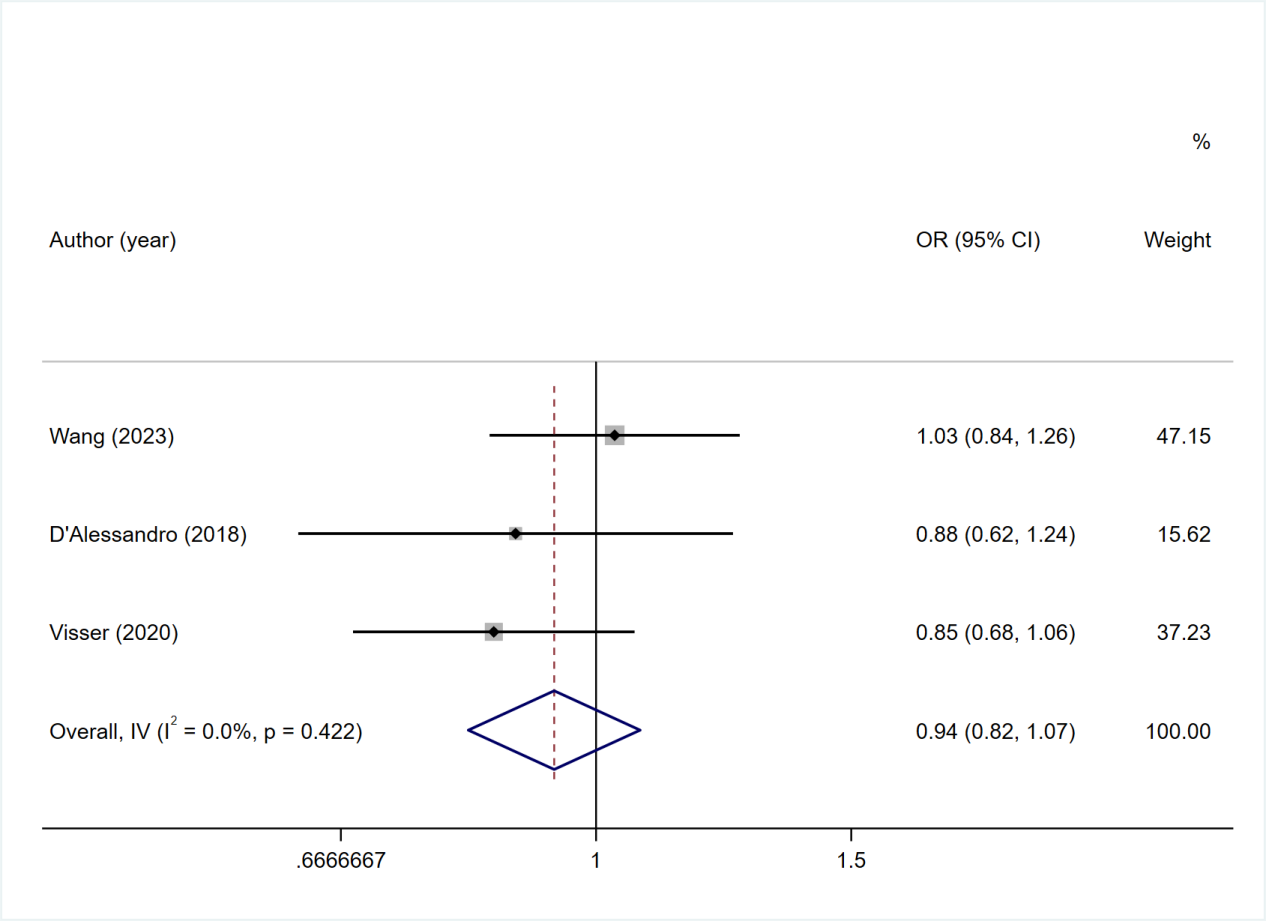
*

Figure S 98Forest Plot of the Association Between PhA and Sarcopenia in Patients with CKD (Excluding Amorim 2022)

**

Figure S 99Sensitivity Analysis of the Association Between PhA and Sarcopenia in Patients with CKD (Excluding Amorim 2022)

*
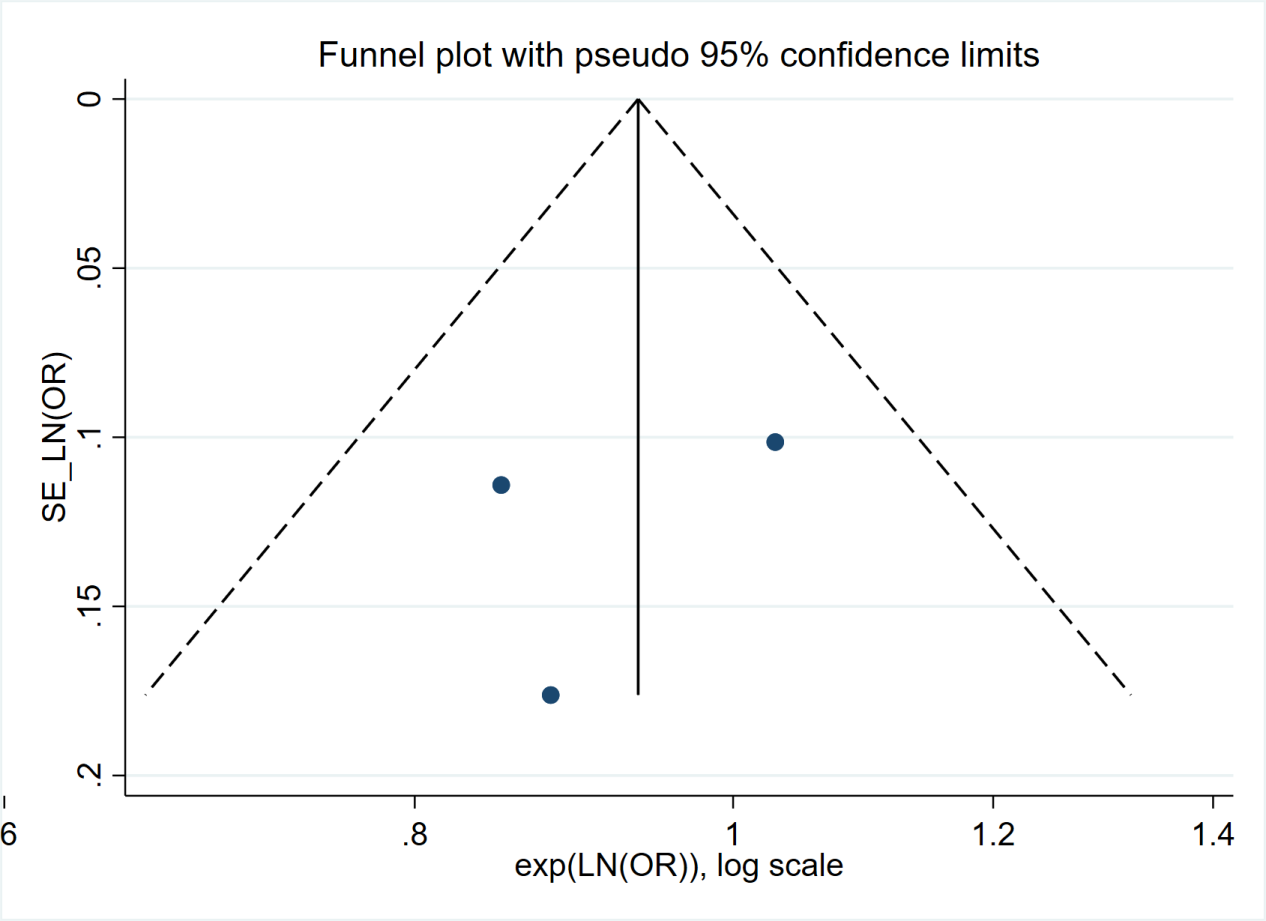
*

Figure S 100Funnel Plot of the Association Between PhA and Sarcopenia in Patients with CKD (Excluding Amorim 2022)

*
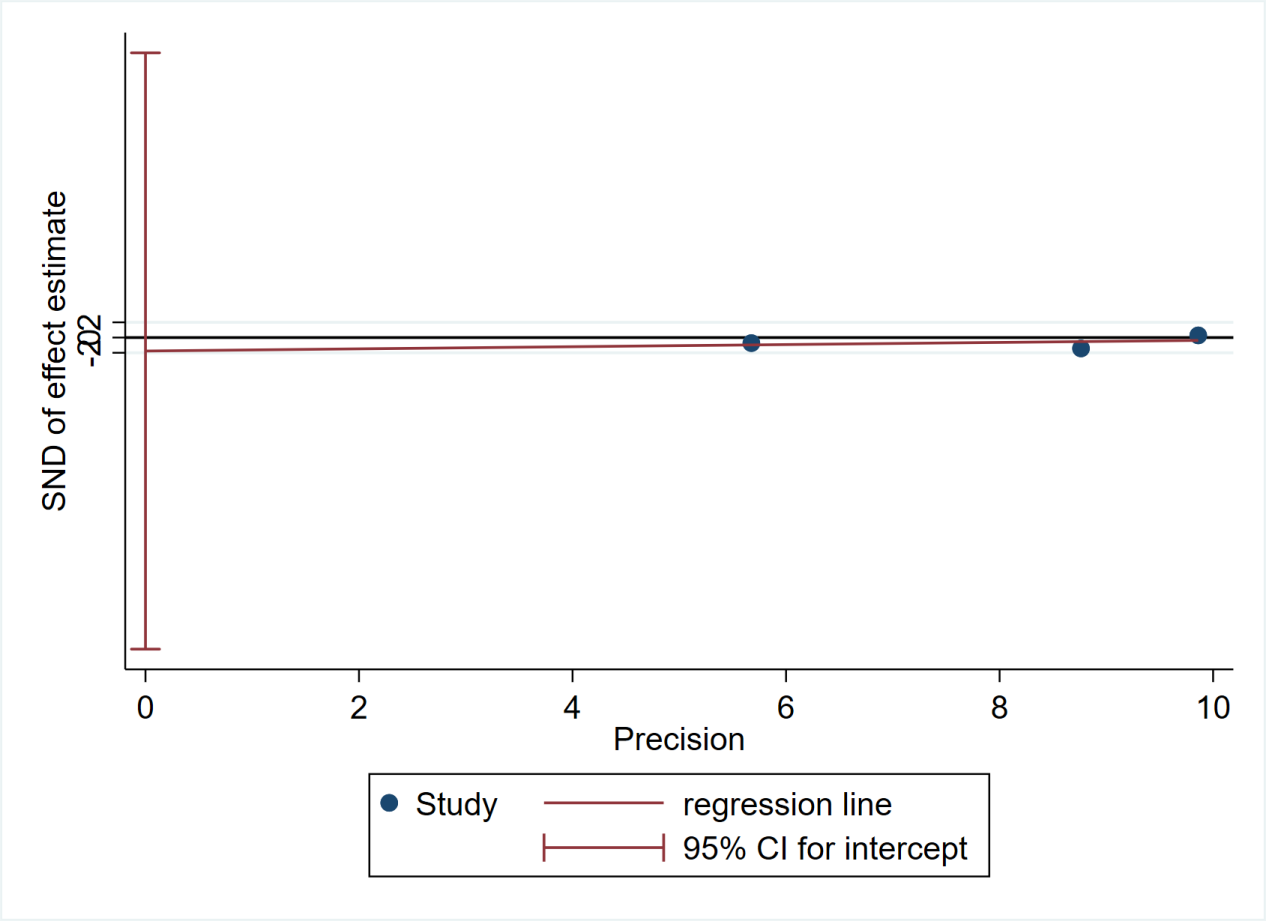
*

**

Figure S 101Egger's Test of the Association Between PhA and Sarcopenia in Patients with CKD (Excluding Amorim 2022)

*Regular exercise*

**

Figure S 102Sensitivity Analysis of the Association Between Regular Exercise and Sarcopenia in Patients with CKD

*
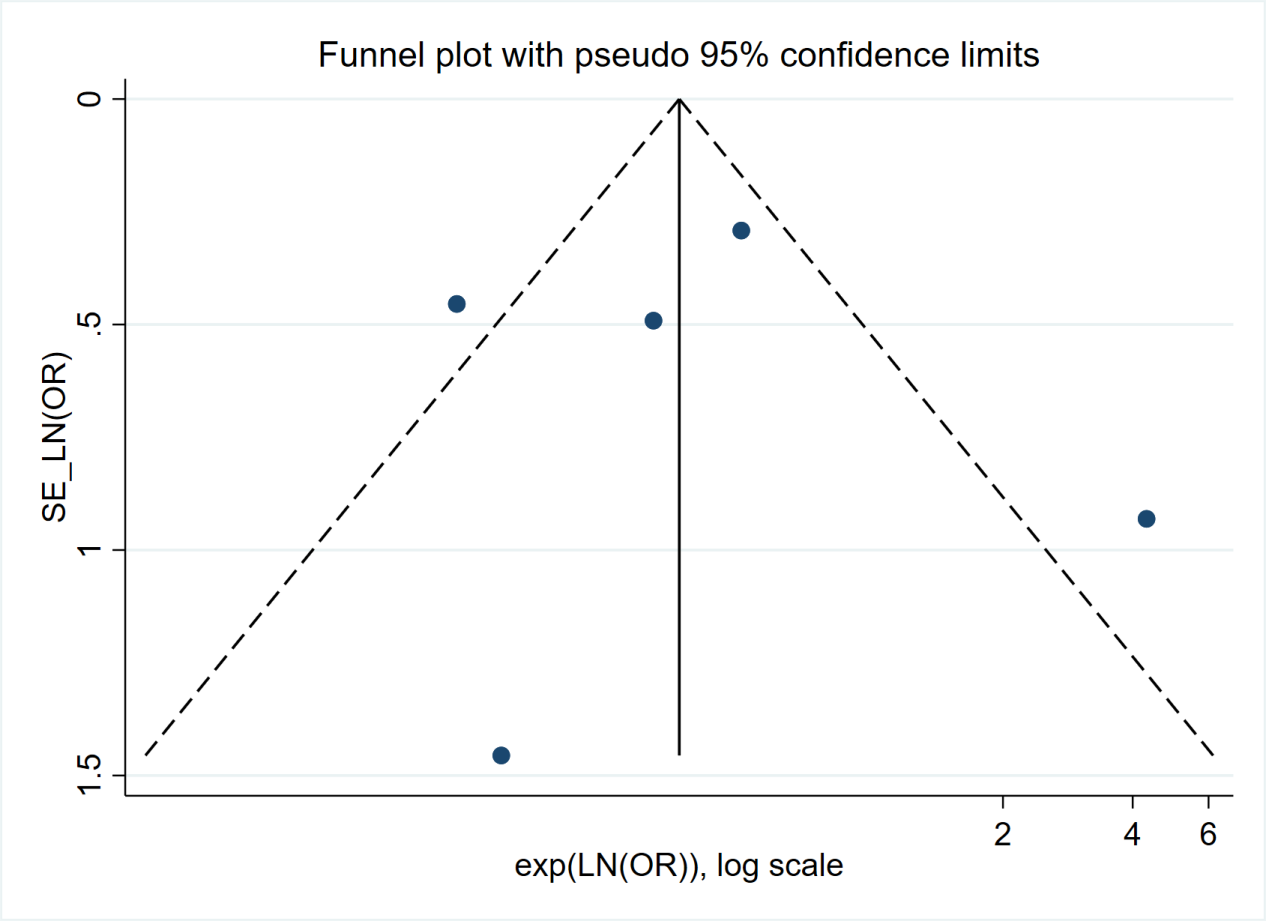
*

Figure S 103Funnel Plot of the Association Between Regular Exercise and Sarcopenia in Patients with CKD

*Figure 72 Funnel Plot of the Association Between Regular Exercise and Sarcopenia in Patients with CKD*

*
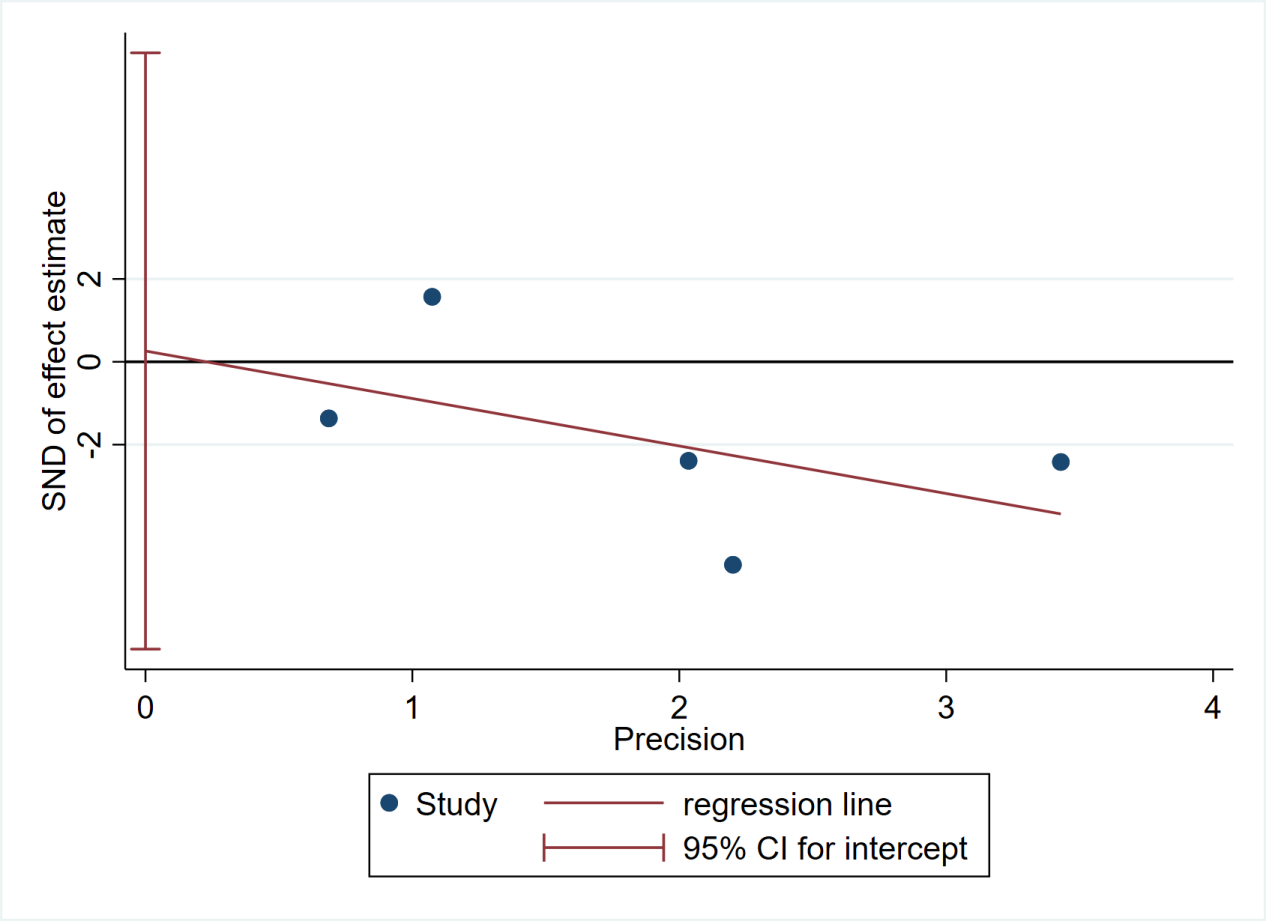
*

**

Figure S 104Egger's Test of the Association Between Regular Exercise and Sarcopenia in Patients with CKD

*Results After Excluding Studies with High Heterogeneity in Sensitivity Analysis*

*By observing the changes in the I² statistic after excluding each study, it was noted that when the study by Wang (2023) was excluded, the I² value decreased from 74.8% to 63.7%, which is not a substantial reduction. However, the exclusion of this study led to a significant change in the overall effect estimate, suggesting that this study may be a primary source of heterogeneity. Upon thorough review of the literature, it was found that this study had a different design compared to the others, as it was a case-control study, whereas the other studies were cross-sectional. Therefore, the difference in study design may be the source of the heterogeneity.*

*
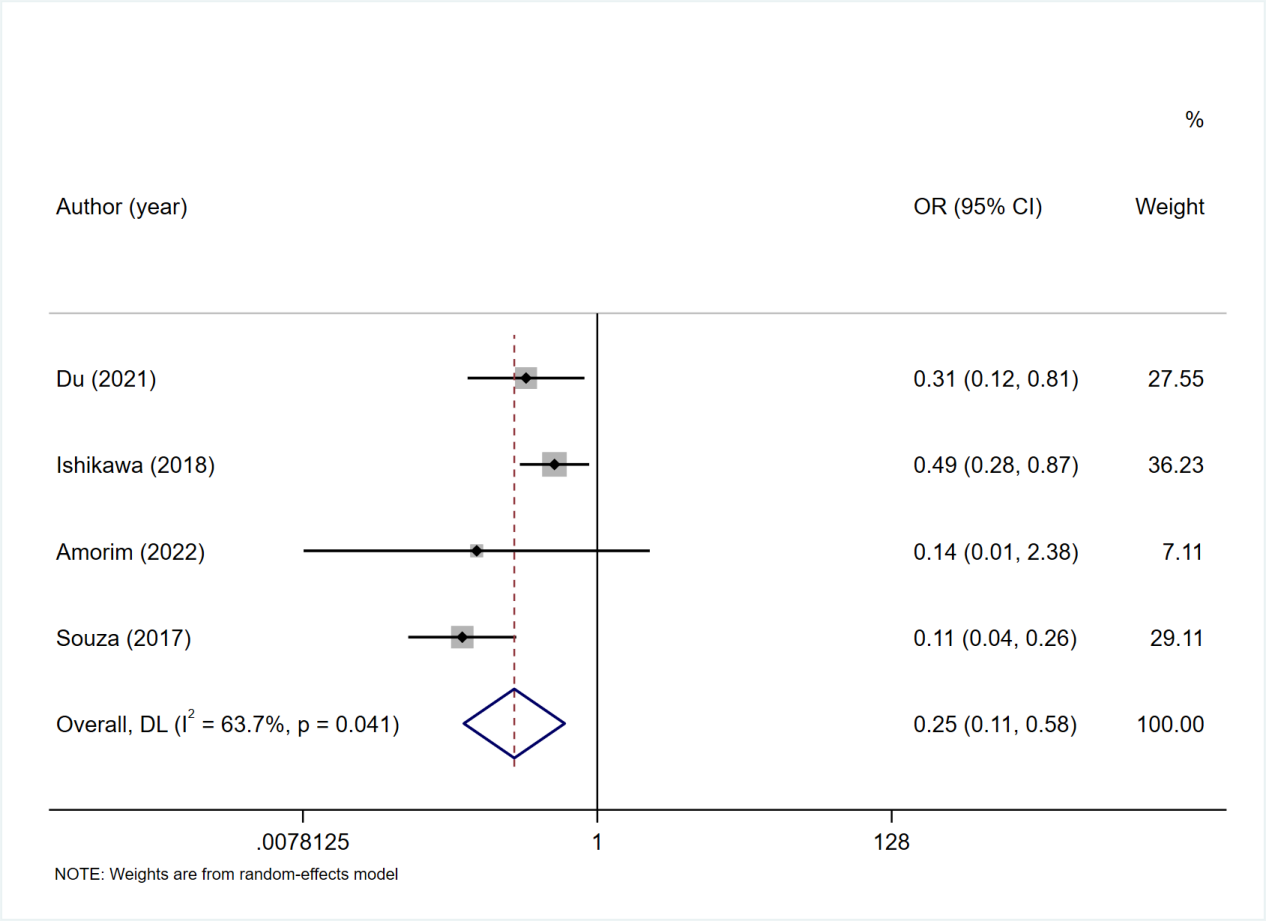
*

Figure S 105Forest Plot of the Association Between Regular Exercise and Sarcopenia in Patients with CKD (Excluding Wang 2023)

**

Figure S 106Sensitivity Analysis of the Association Between Regular Exercise and Sarcopenia in Patients with CKD (Excluding Wang 2023)

*Figure 75 Sensitivity Analysis of the Association Between Regular Exercise and Sarcopenia in Patients with CKD (Excluding Wang 2023)*

*
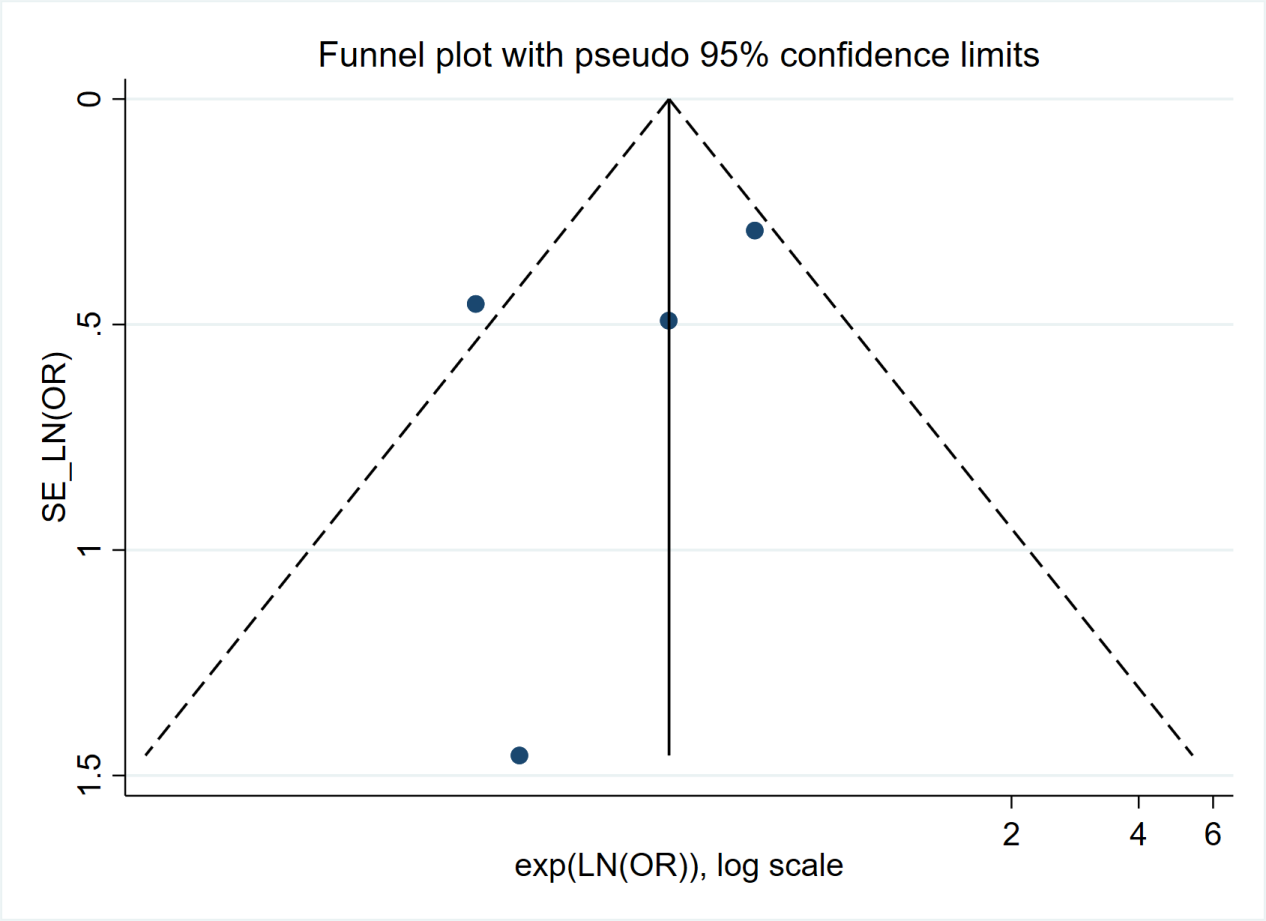
*

Figure S 107Funnel Plot of the Association Between Regular Exercise and Sarcopenia in Patients with CKD (Excluding Wang 2023)

*
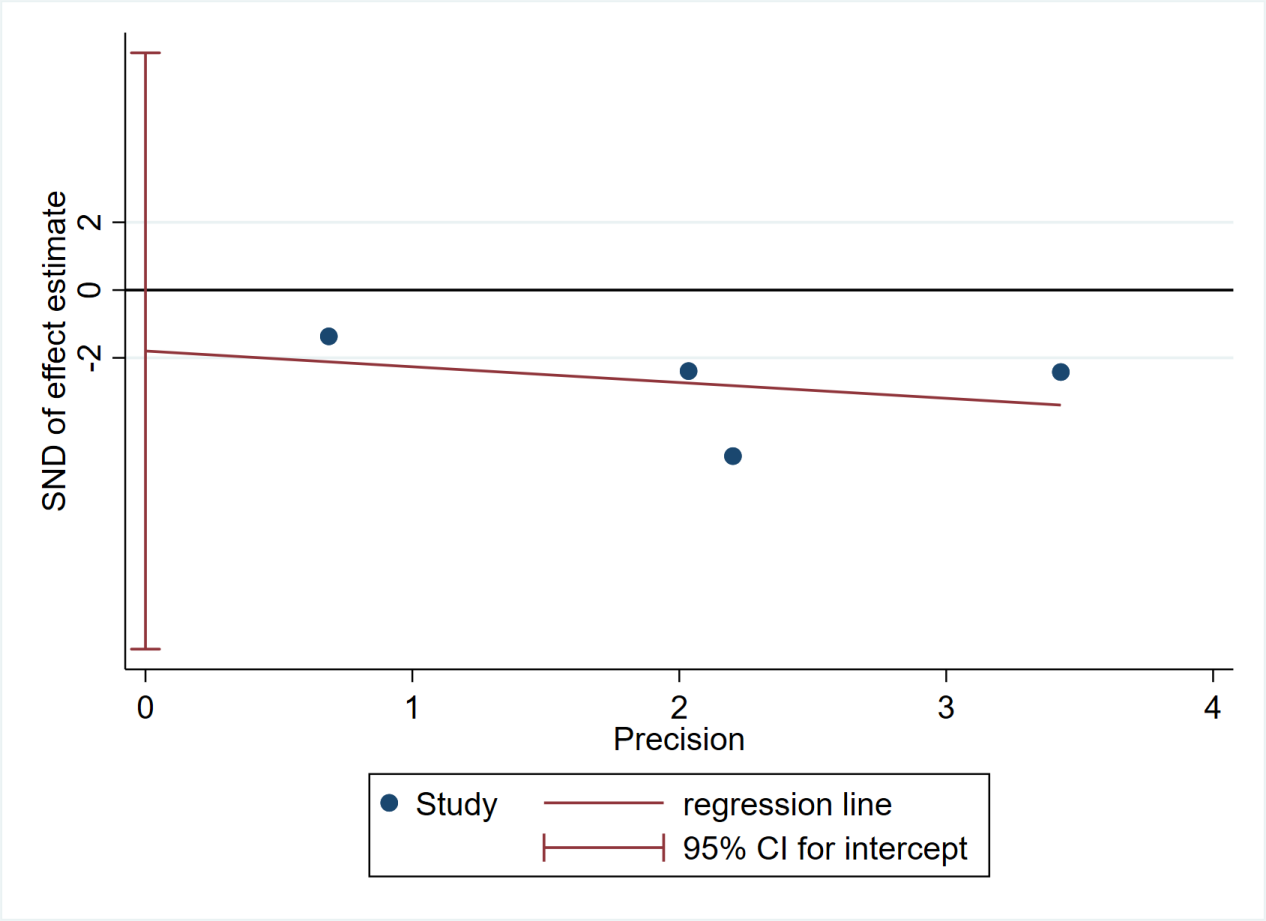
*

**

Figure S 108Egger's Test of the Association Between Regular Exercise and Sarcopenia in Patients with CKD (Excluding Wang 2023)

*serum albumin*

Figure S 109Sensitivity Analysis of the Association Between Serum Albumin and Sarcopenia in Patients with CKD

Figure S 110Funnel Plot of the Association Between Serum Albumin and Sarcopenia in Patients with CKD

Figure S 111Egger's Test of the Association Between Serum Albumin and Sarcopenia in Patients with CKD

*Serum calcium*

Figure S 112Sensitivity Analysis of the Association Between Serum Calcium and Sarcopenia in Patients with CKD

*
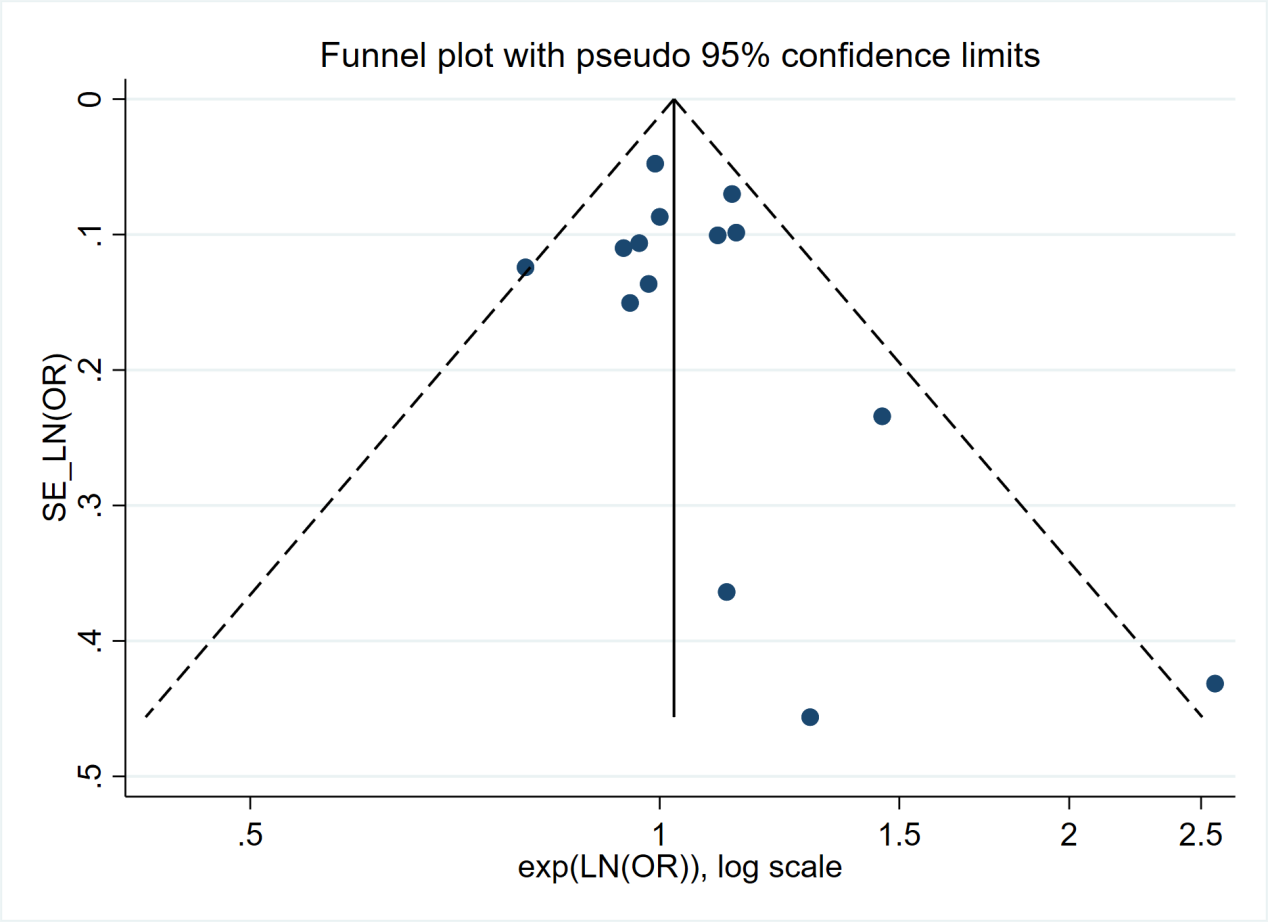
*

Figure S 113Funnel Plot of the Association Between Serum Calcium and Sarcopenia in Patients with CKD

*
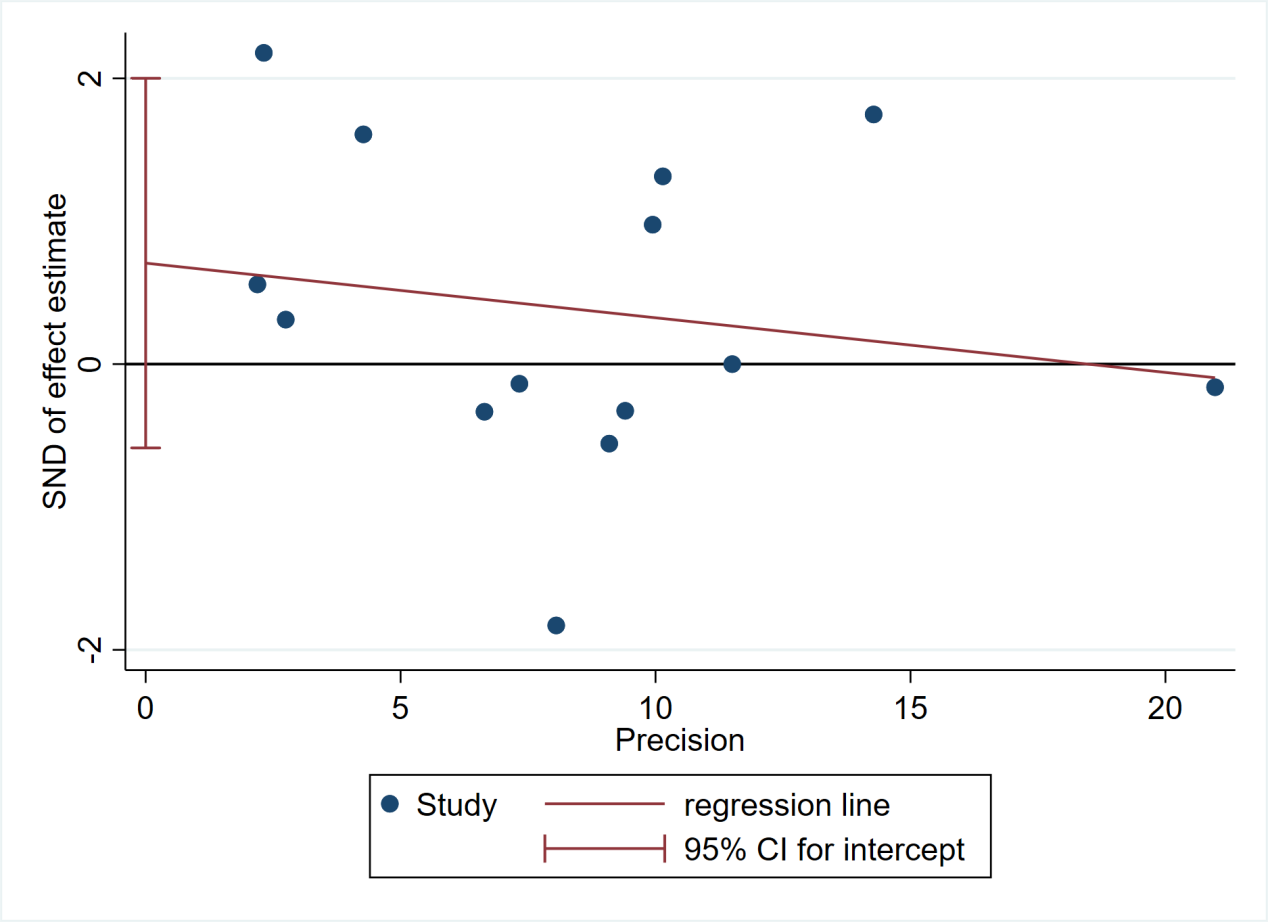
*

Figure S 114Egger's Test of the Association Between Serum Calcium and Sarcopenia in Patients with CKD

*Serum phosphorus*

**

Figure S 115Sensitivity Analysis of the Association Between Serum phosphorus and Sarcopenia in Patients with CKD

*
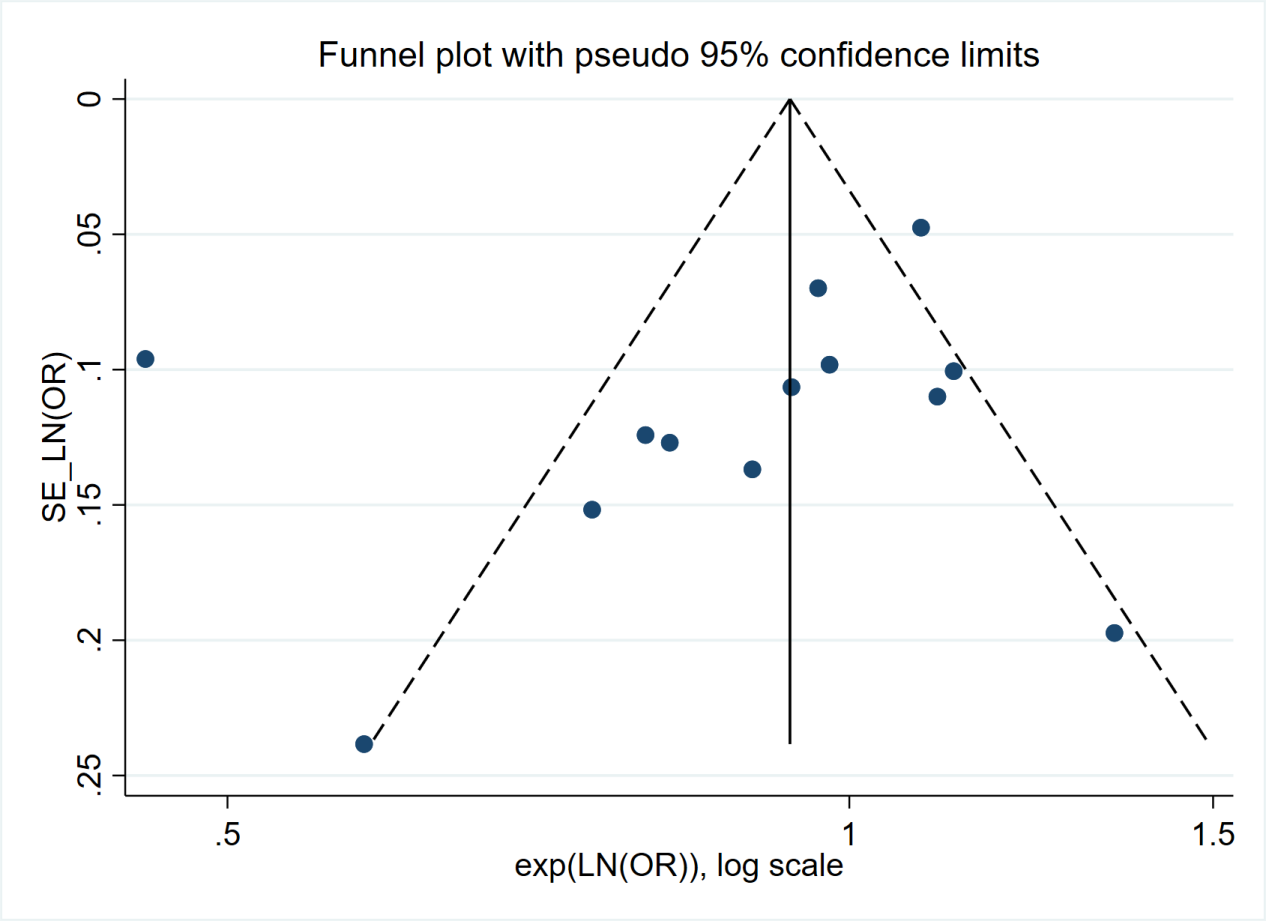
*

Figure S 116Funnel Plot of the Association Between Serum phosphorus and Sarcopenia in Patients with CKD

*
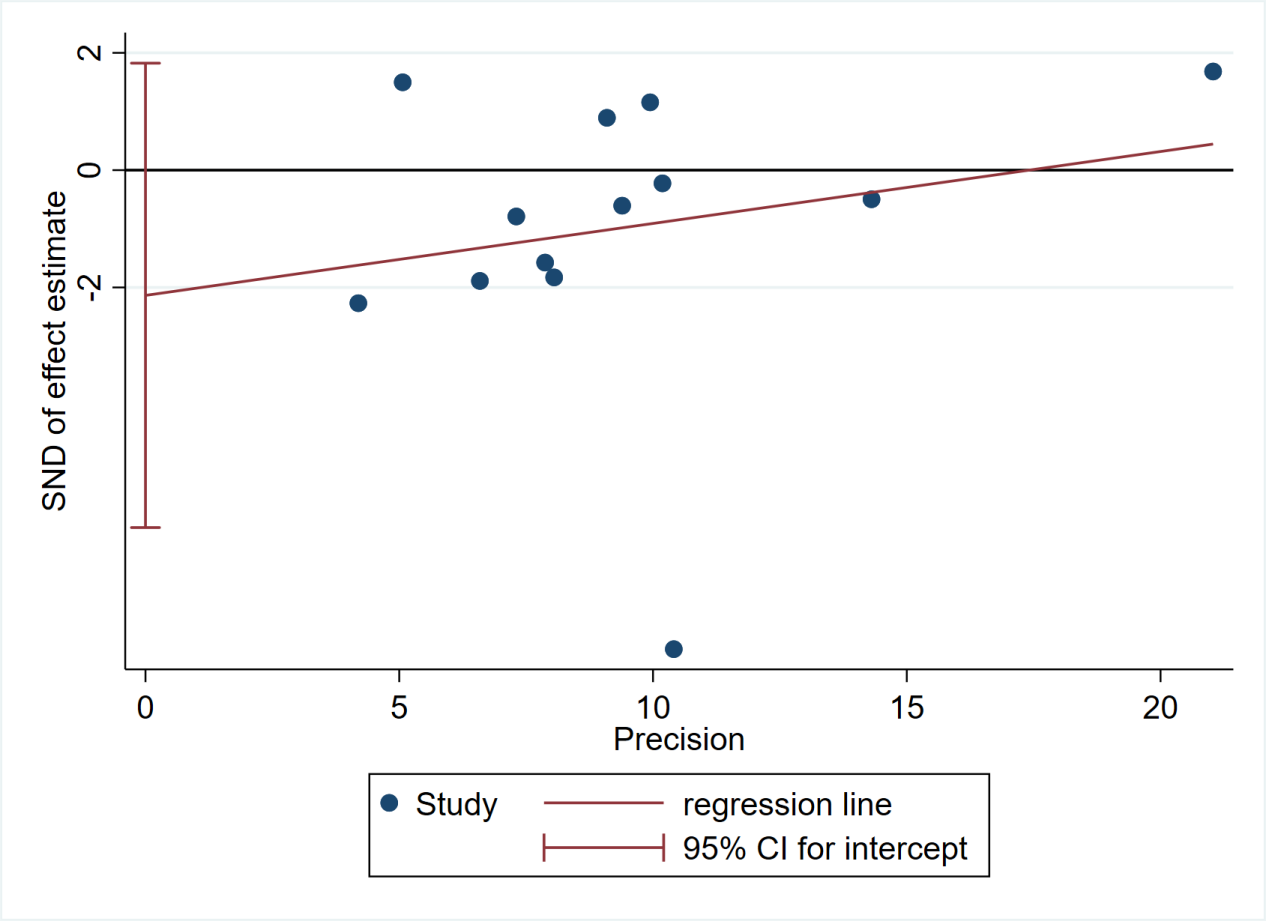
*

**

Figure S 117Egger's Test of the Association Between Serum phosphorus and Sarcopenia in Patients with CKD

**

Figure S 118Meta-Regression Analysis of the Association Between Serum phosphorus and Sarcopenia in Patients with CKD

*
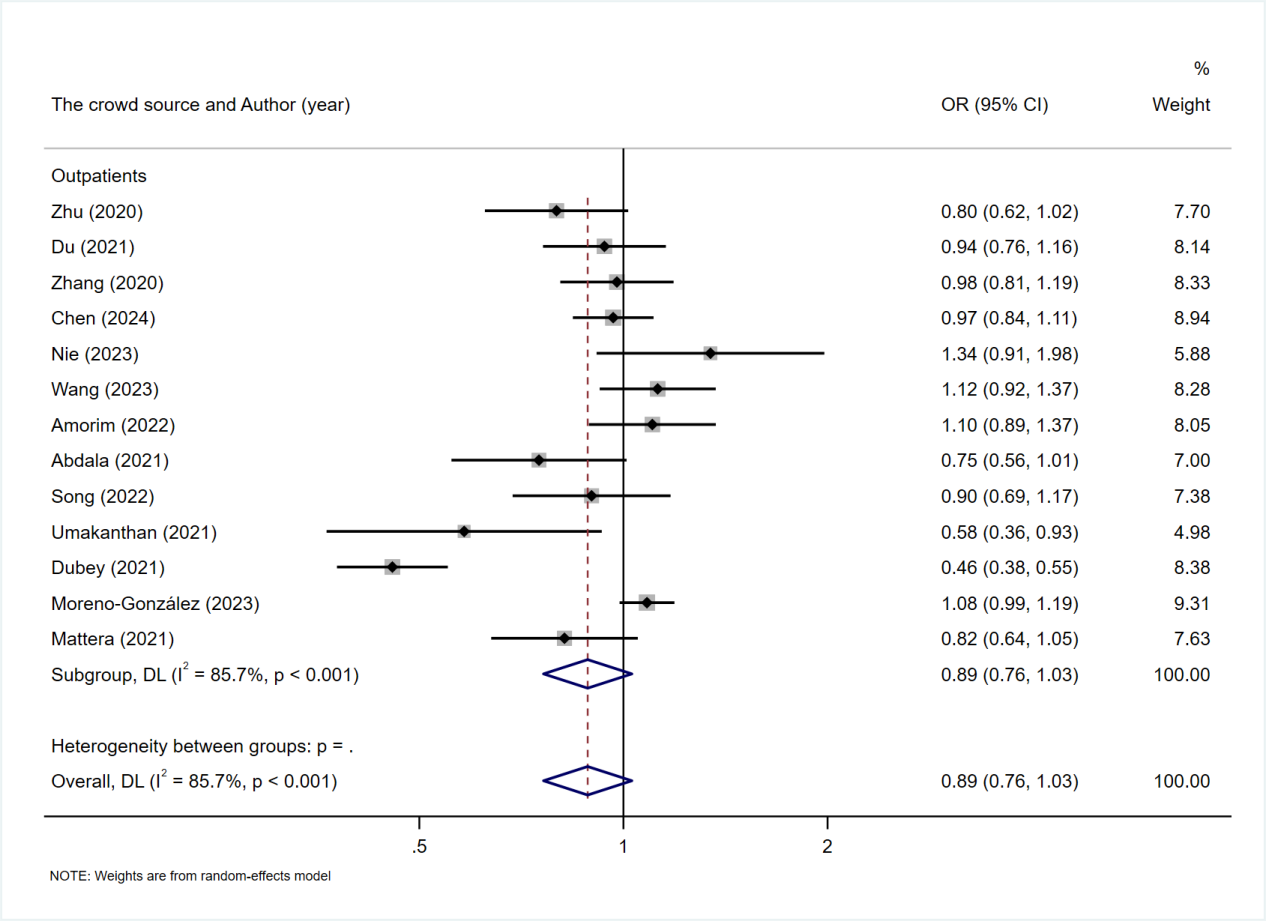
*

Figure S 119Forest Plot of the Subgroup Analysis of the Association Between Serum phosphorus and Sarcopenia in Patients with CKD Based on the Crowds Source

*
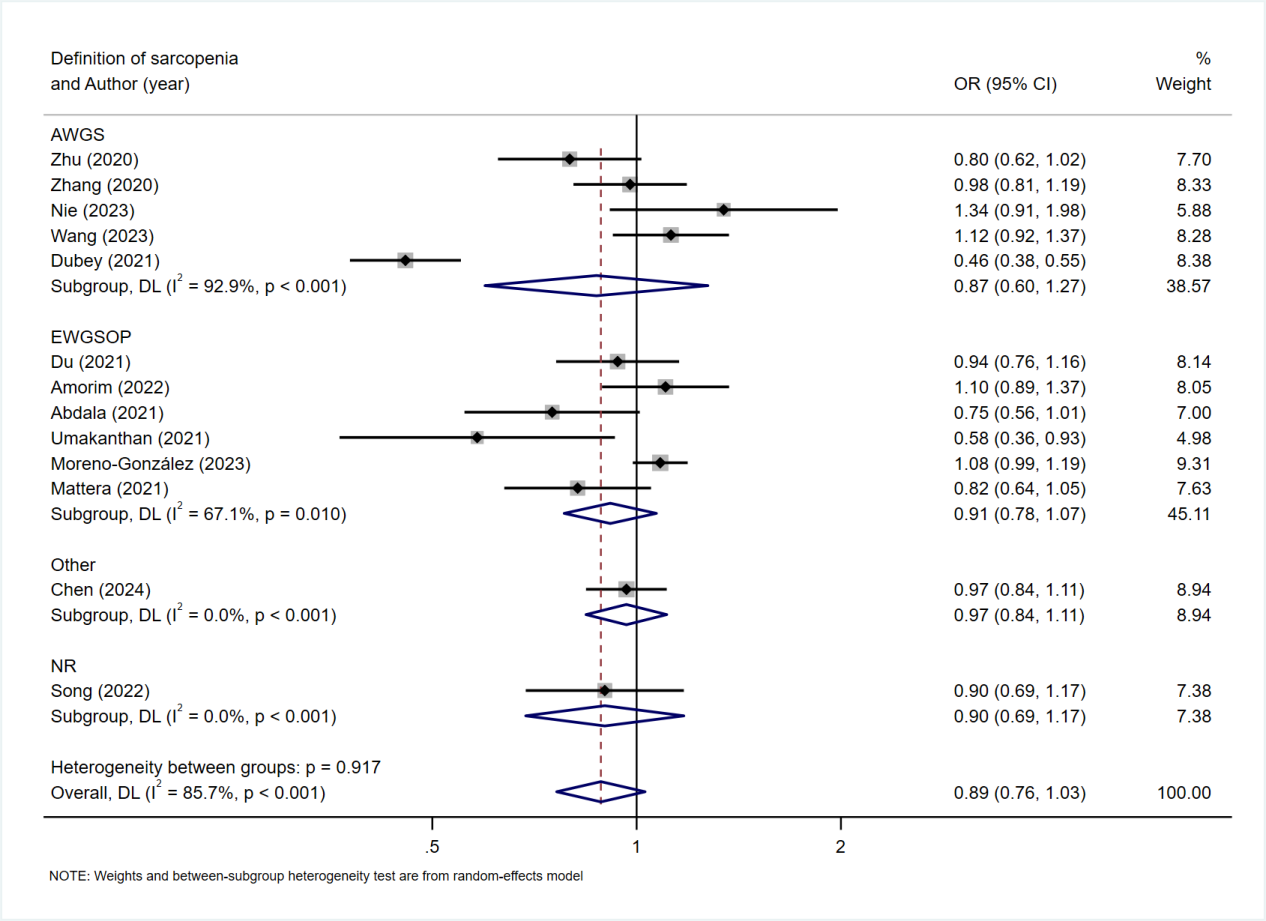
*

Figure S 120Forest Plot of the Subgroup Analysis of the Association Between Serum phosphorus and Sarcopenia in Patients with CKD Based on the Definition of Sarcopenia

*
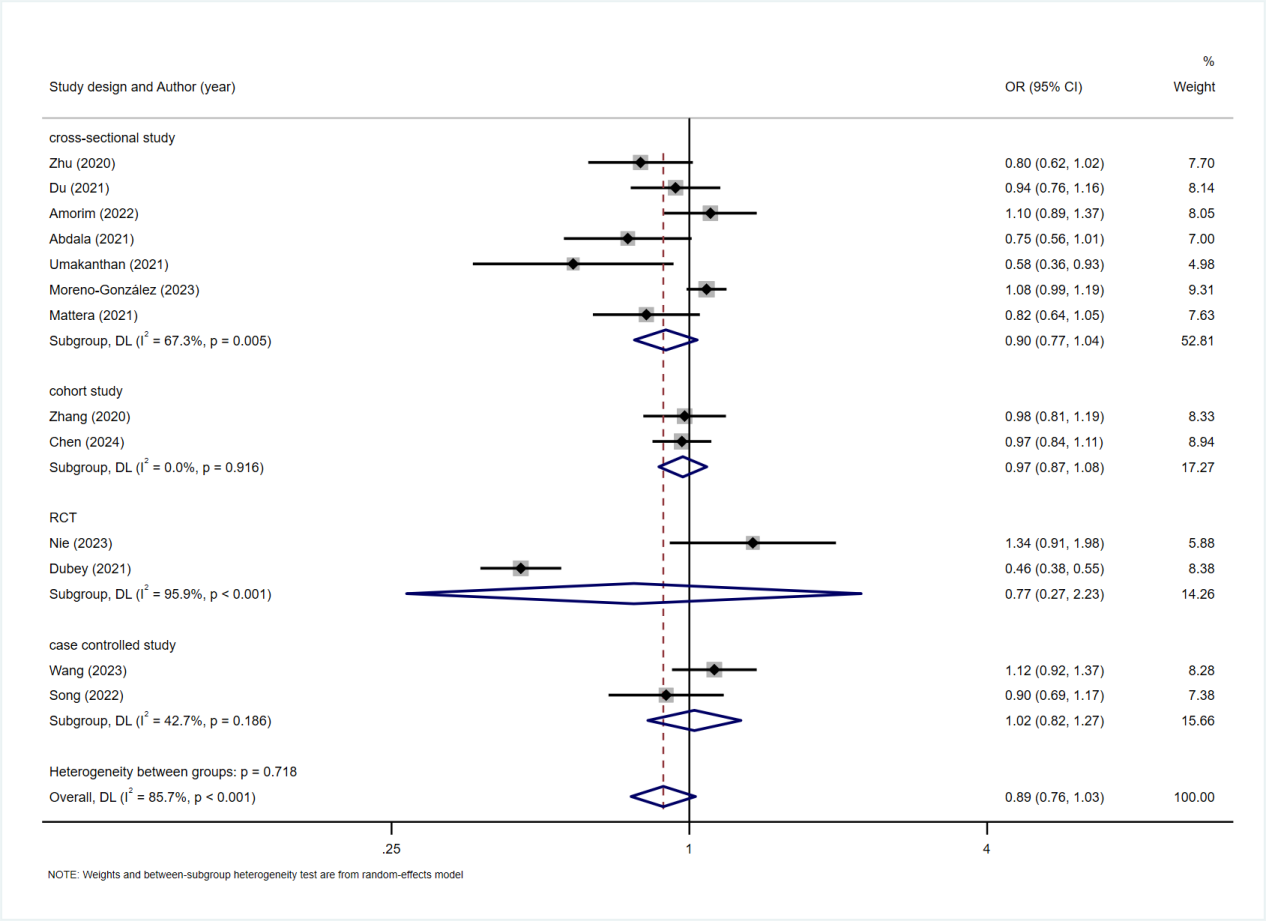
*

Figure S 121Forest Plot of the Subgroup Analysis of the Association Between Serum phosphorus and Sarcopenia in Patients with CKD Based on the Study Design

*
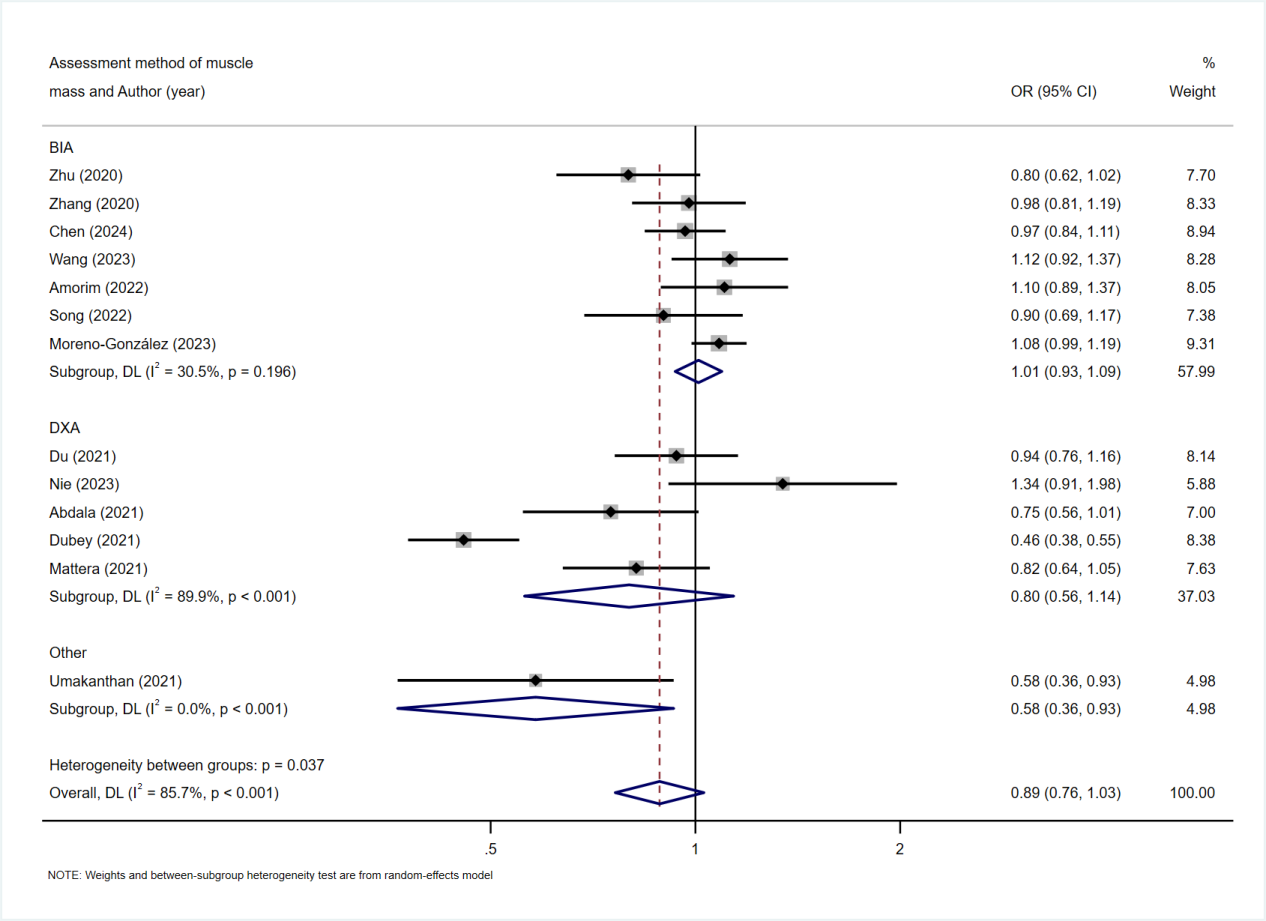
*

Figure S 122Forest Plot of the Subgroup Analysis of the Association Between Serum phosphorus and Sarcopenia in Patients with CKD Based on the Assessment Method of Muscle Mass

*
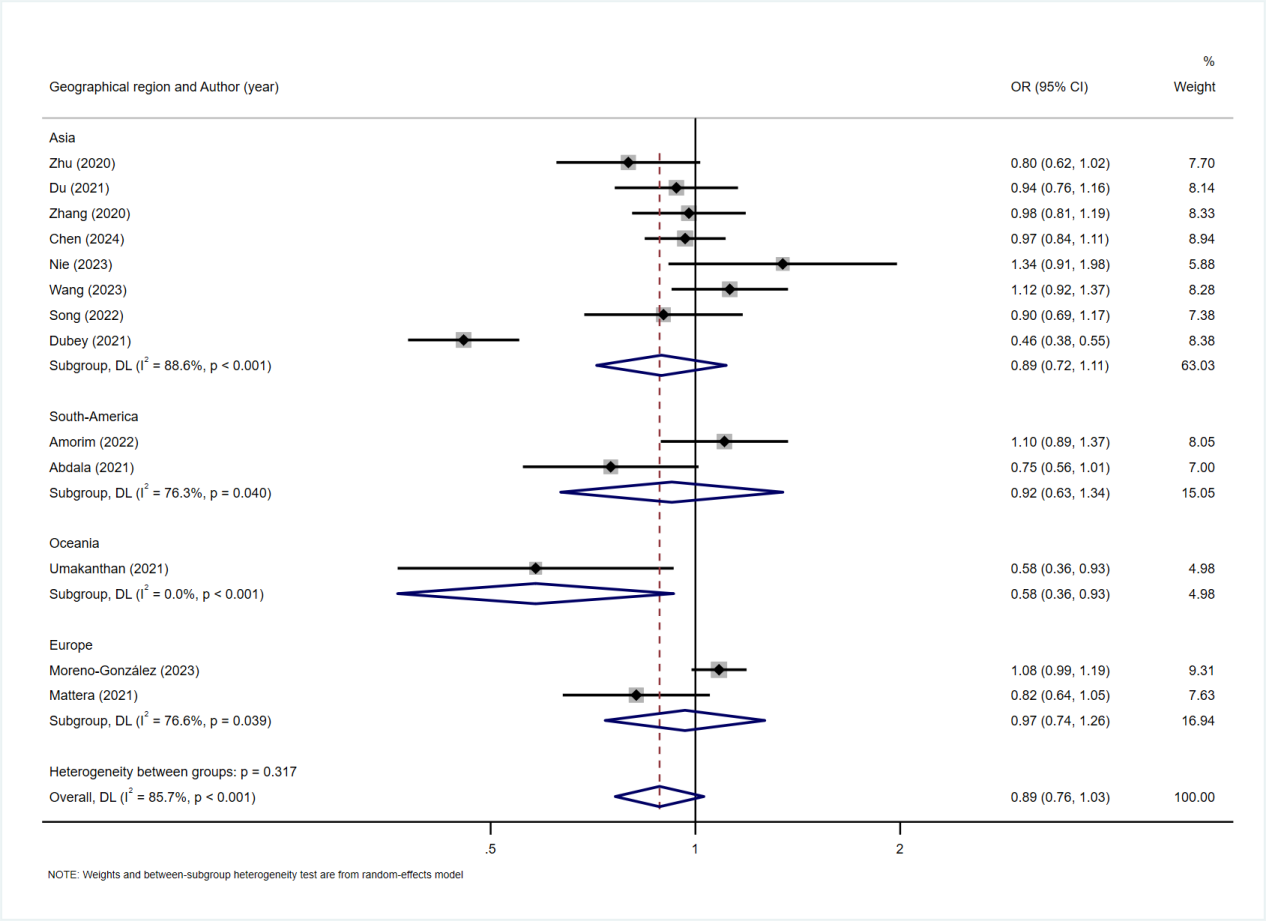
*

Figure S 123Forest Plot of the Subgroup Analysis of the Association Between Serum phosphorus and Sarcopenia in Patients with CKD Based on the Geographical Region

*
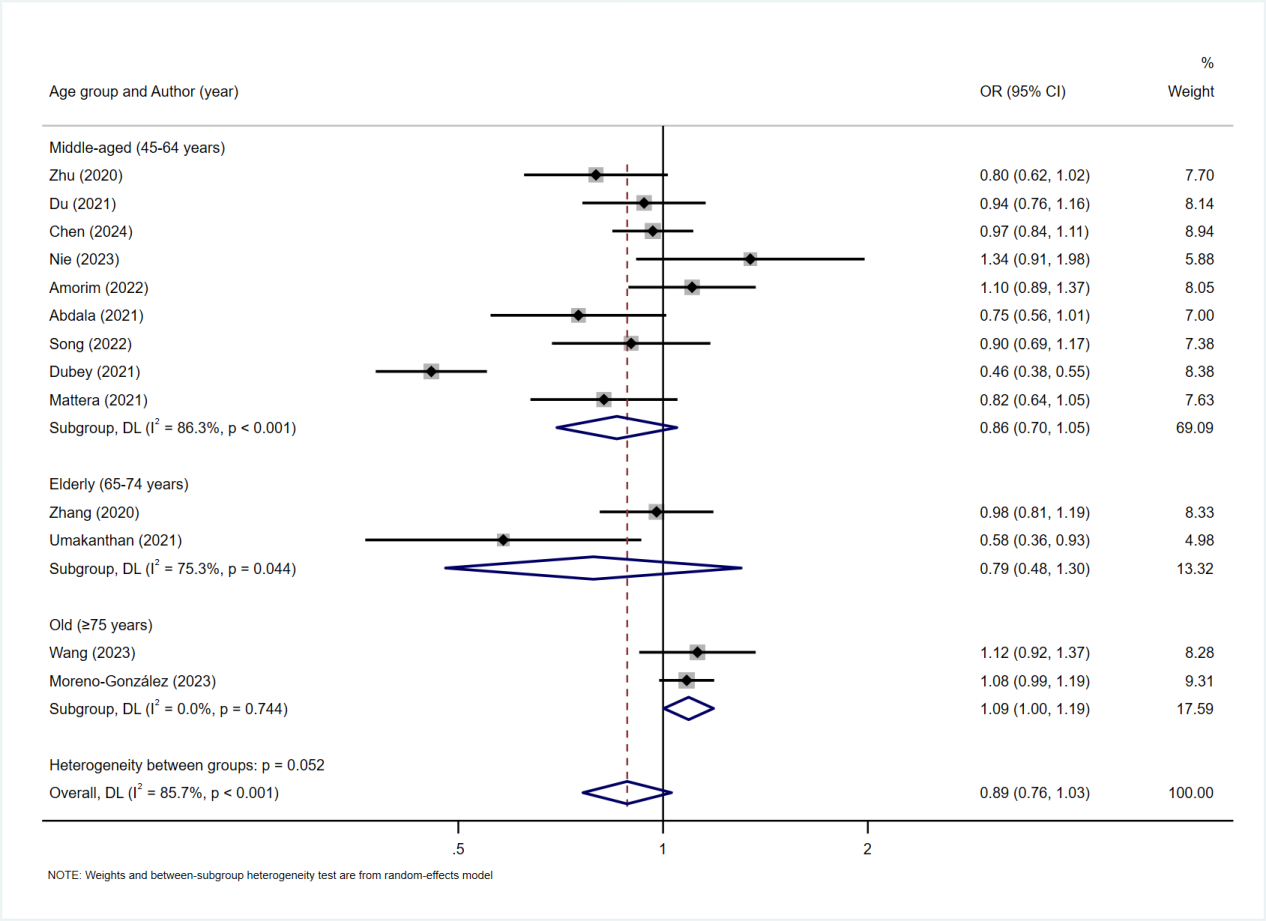
*

Figure S 124Forest Plot of the Subgroup Analysis of the Association Between Serum phosphorus and Sarcopenia in Patients with CKD Based on the Age Group

*Figure 93 Forest Plot of the Subgroup Analysis of the Association Between Serum phosphorus and Sarcopenia in Patients with CKD Based on the Age Group*

*Despite conducting comprehensive analyses, including sensitivity analysis, subgroup analysis, and meta-regression, no specific source of heterogeneity was identified. The observed heterogeneity may be attributed to unmeasured or unreported factors, variations in study design, or differences in population characteristics that were not fully captured in the included studies. As a result, the source of heterogeneity remains unclear.*

*Serum PTH(Serum Parathyroid Hormone)*

**

Figure S 125Sensitivity Analysis of the Association Between Serum PTH and Sarcopenia in Patients with CKD

*
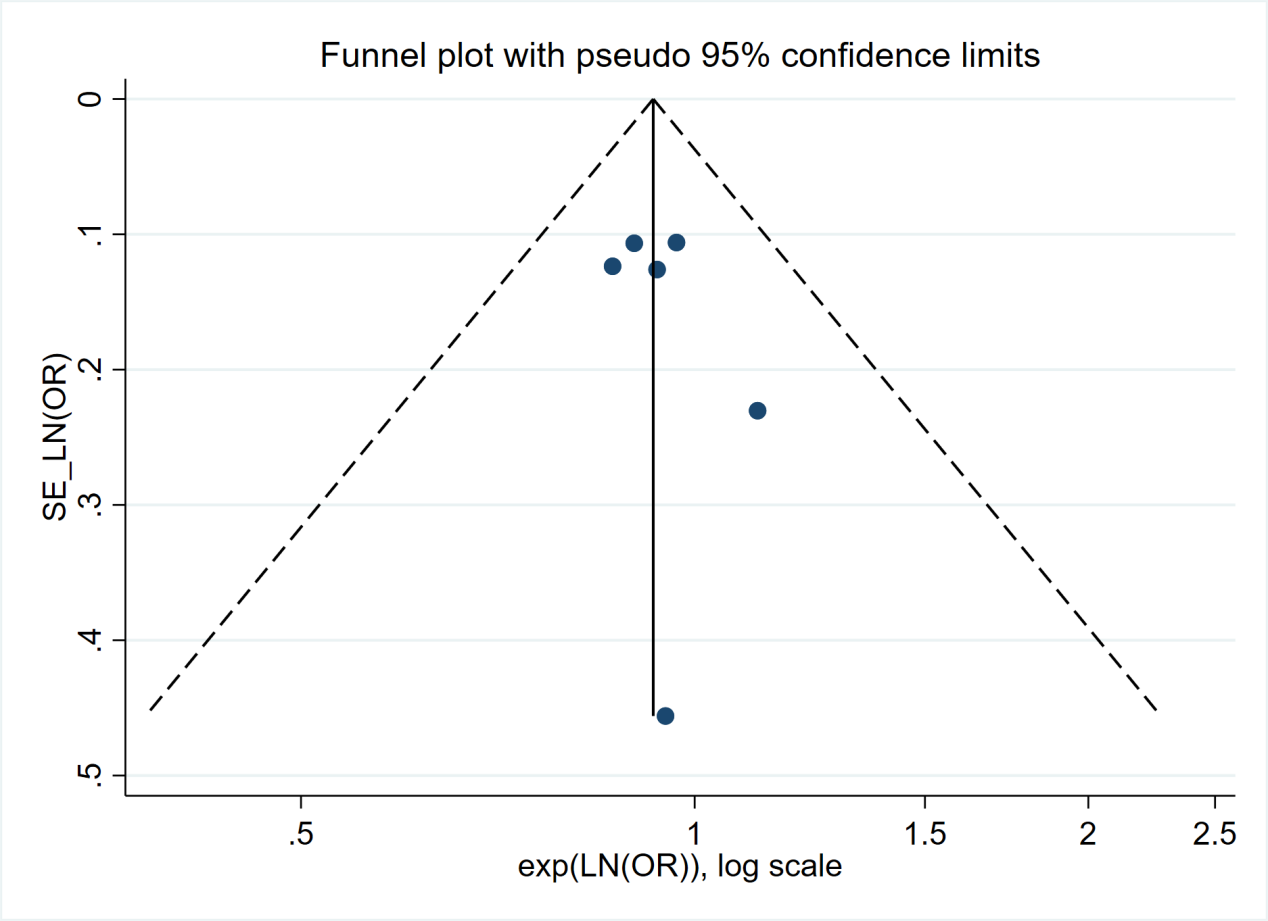
*

Figure S 126Funnel Plot of the Association Between Serum PTH and Sarcopenia in Patients with CKD

*
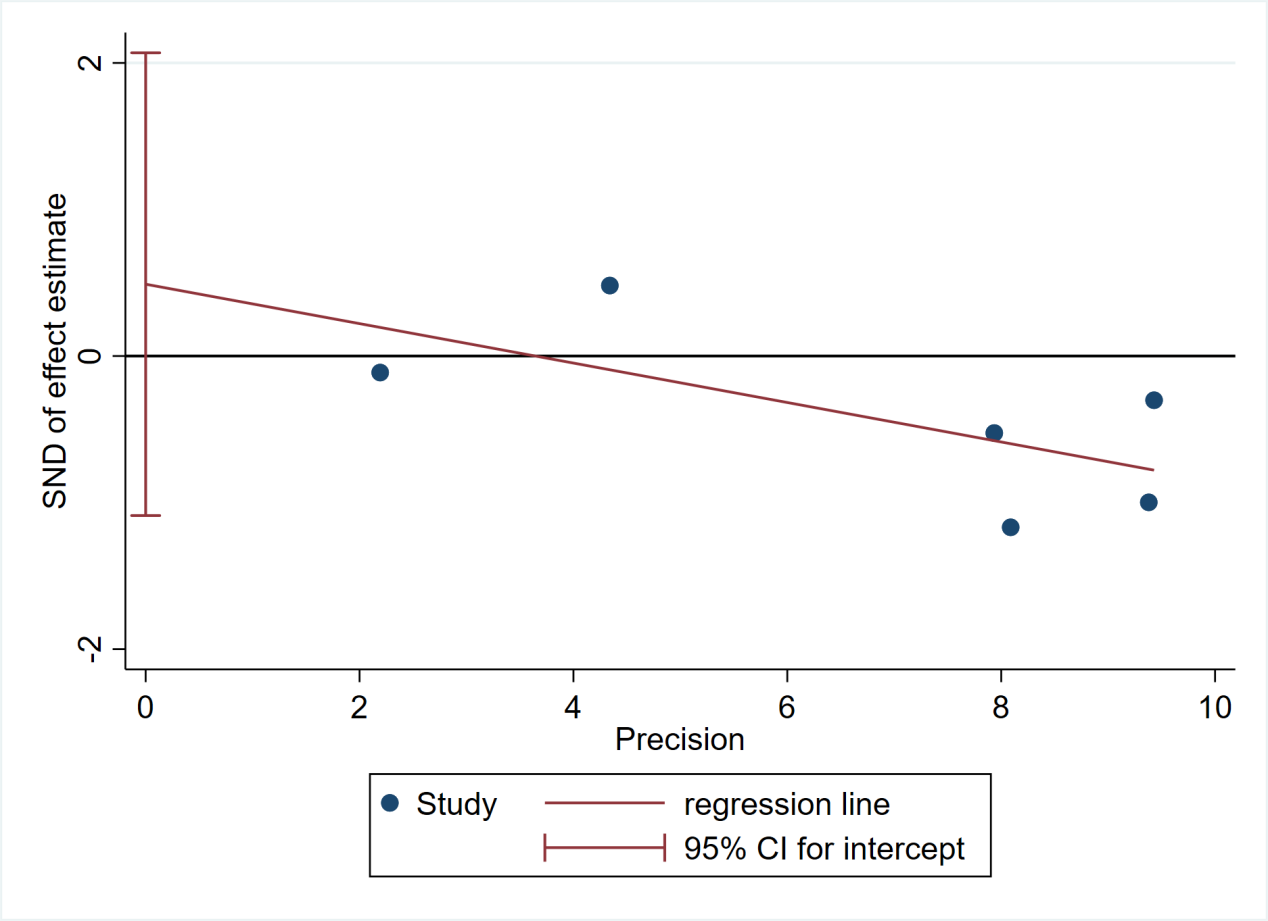
*

**

Figure S 127Egger's Test of the Association Between Serum PTH and Sarcopenia in Patients with CKD

*Smoking history*

**

Figure S 128Sensitivity Analysis of the Association Between Smoking History and Sarcopenia in Patients with CKD

*
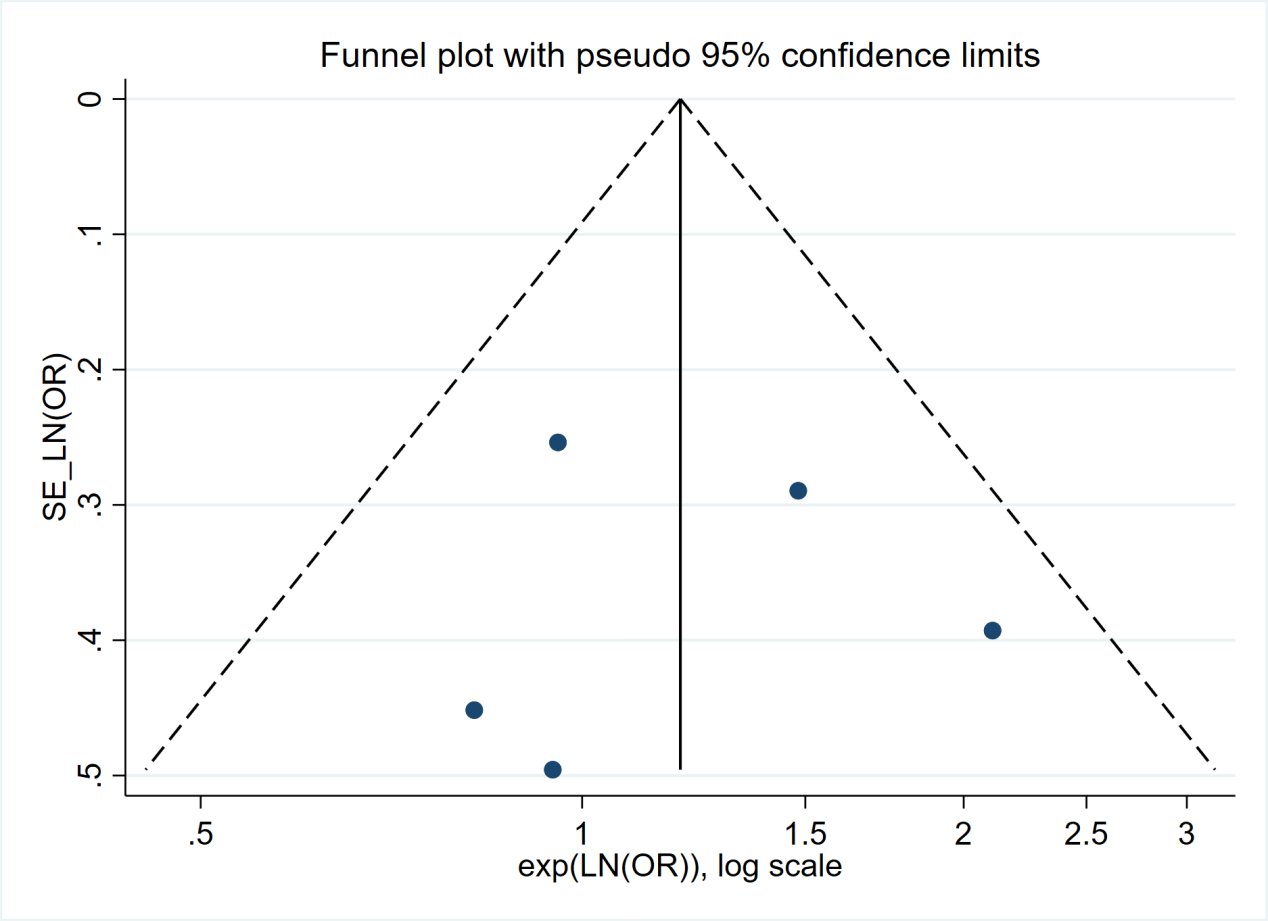
*

Figure S 129Funnel Plot of the Association Between Smoking History and Sarcopenia in Patients with CKD

*
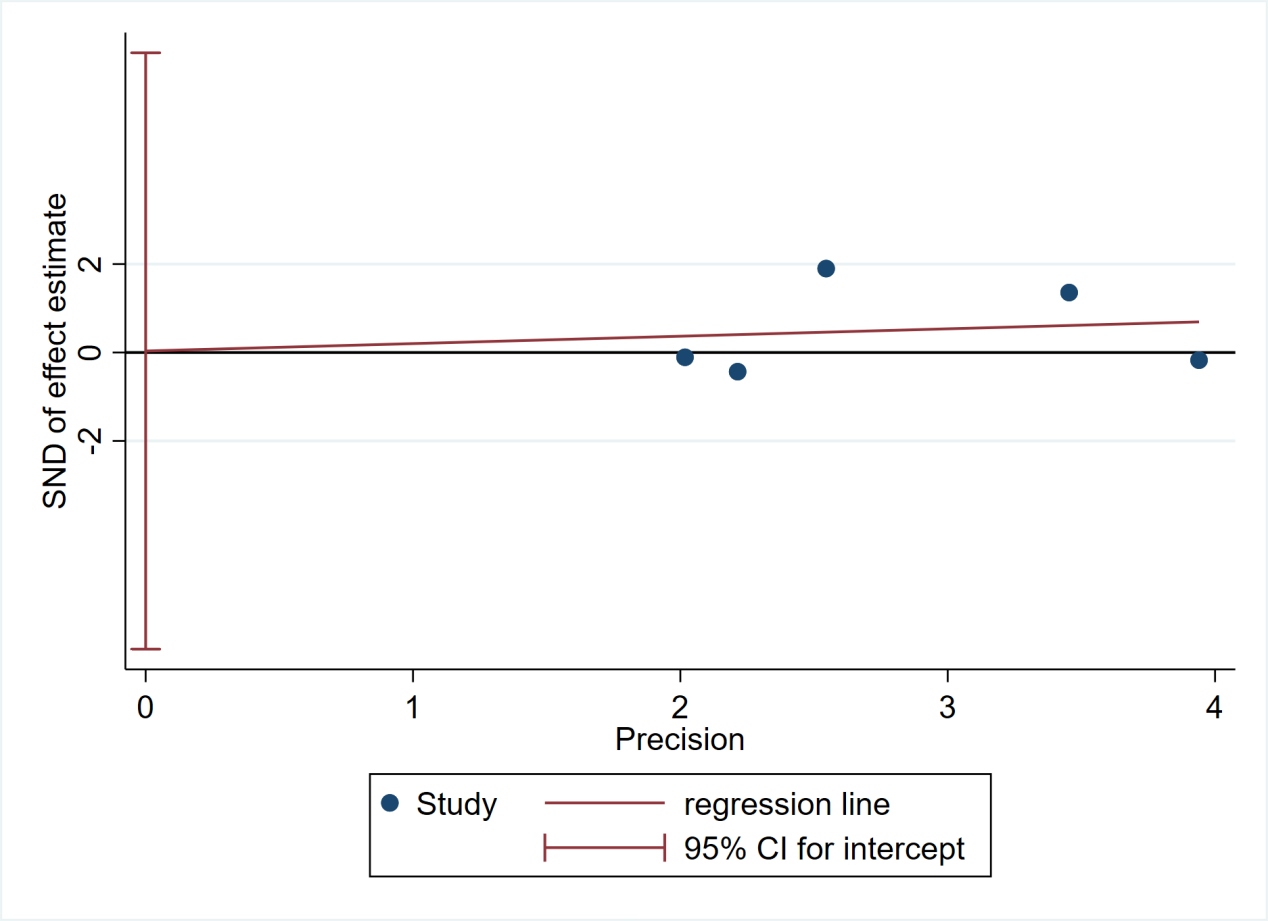
*

**

Figure S 130Egger's Test of the Association Between Smoking History and Sarcopenia in Patients with CKD

*CKD Stage 3 and 4*

Figure S 131Sensitivity Analysis of the Association Between CKD Stage 3 and Sarcopenia in Patients with CKD

Figure S 132Funnel Plot of the Association Between CKD Stage 3 and Sarcopenia in Patients with CKD

Figure S 133Egger's Test of the Association Between CKD Stage 3 and Sarcopenia in Patients with CKD

Figure S 134Sensitivity Analysis of the Association Between CKD Stage 4 and Sarcopenia in Patients with CKD

Figure S 135Funnel Plot of the Association Between CKD Stage 4 and Sarcopenia in Patients with CKD

Figure S 136Egger's Test of the Association Between CKD Stage 4 and Sarcopenia in Patients with CKD

*TC(Total Cholesterol)*

Figure S 137Sensitivity Analysis of the Association Between TC and Sarcopenia in Patients with CKD

*
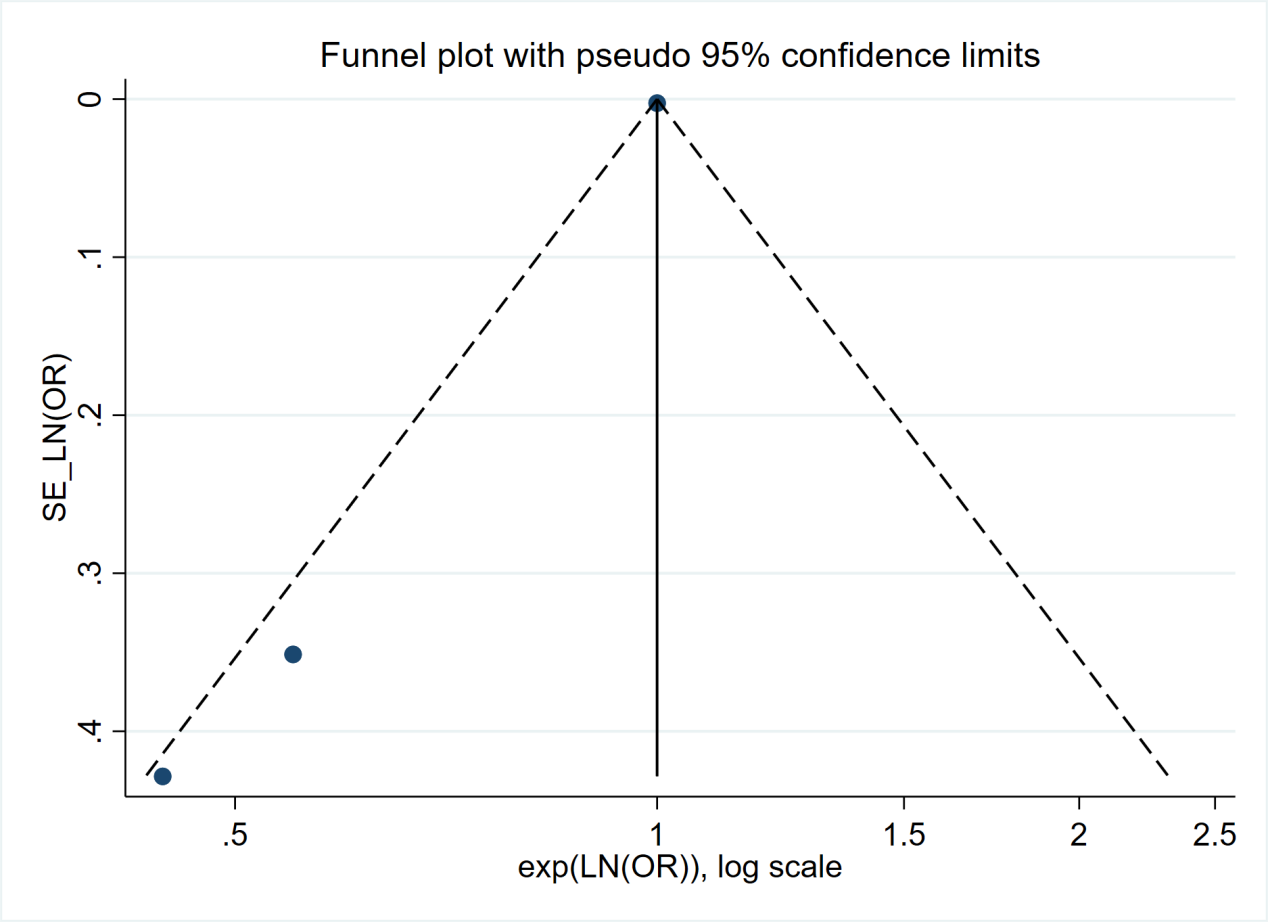
*

Figure S 138Funnel Plot of the Association Between TC and Sarcopenia in Patients with CKD

*
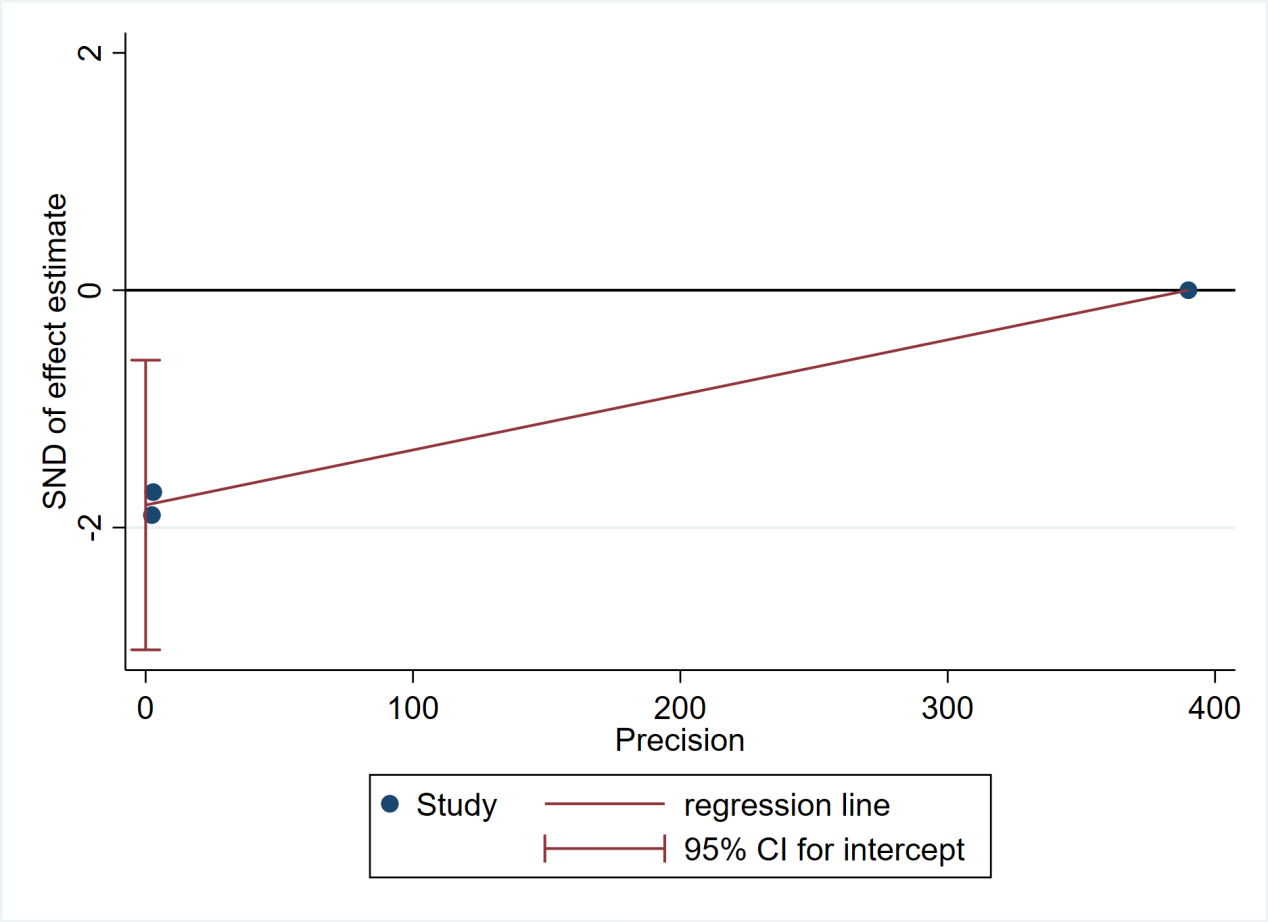
*

*
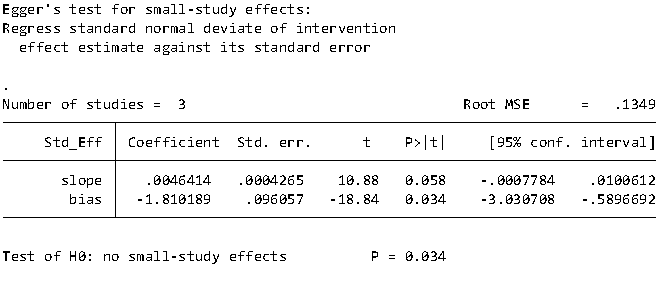
*

Figure S 139Egger's Test of the Association Between TC and Sarcopenia in Patients with CKD

*
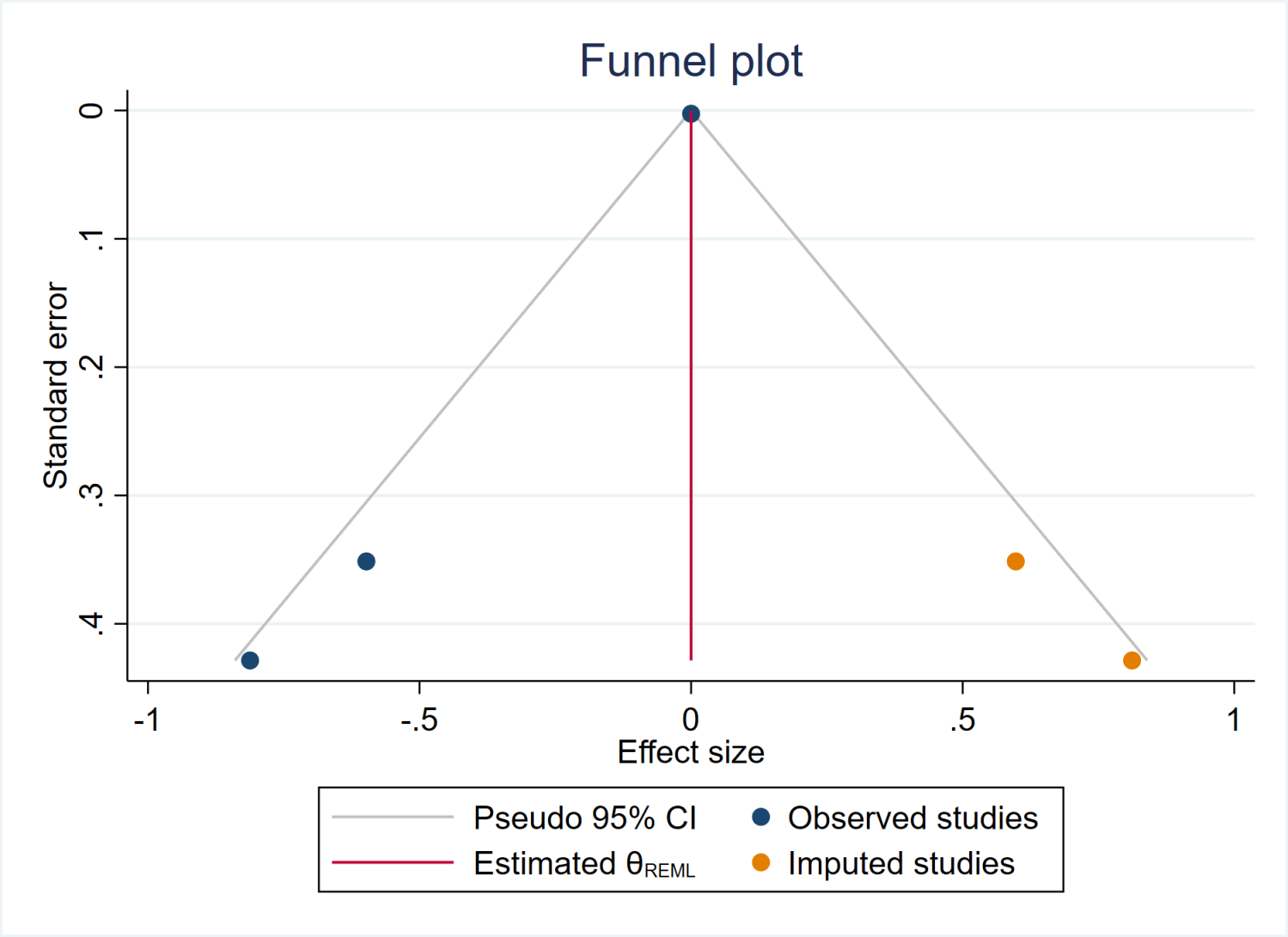

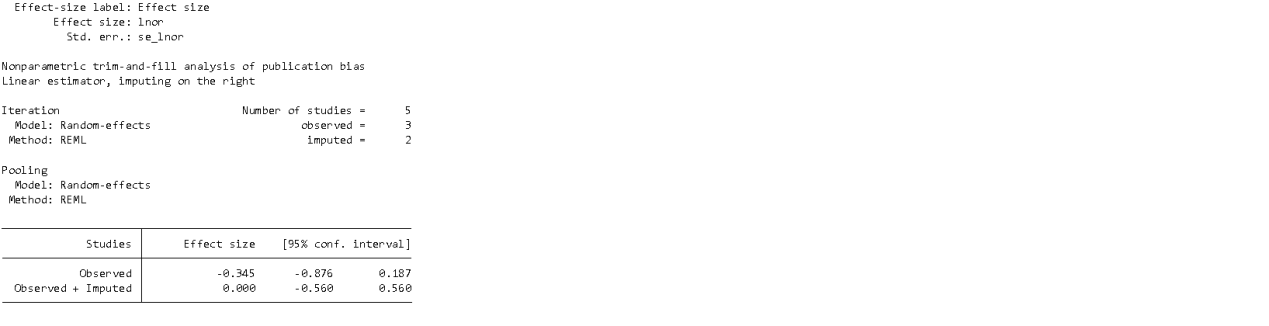
*

Figure S 140Funnel Plot after Trim and Fill Correction of the Association Between TC and Sarcopenia in Patients with CKD

*
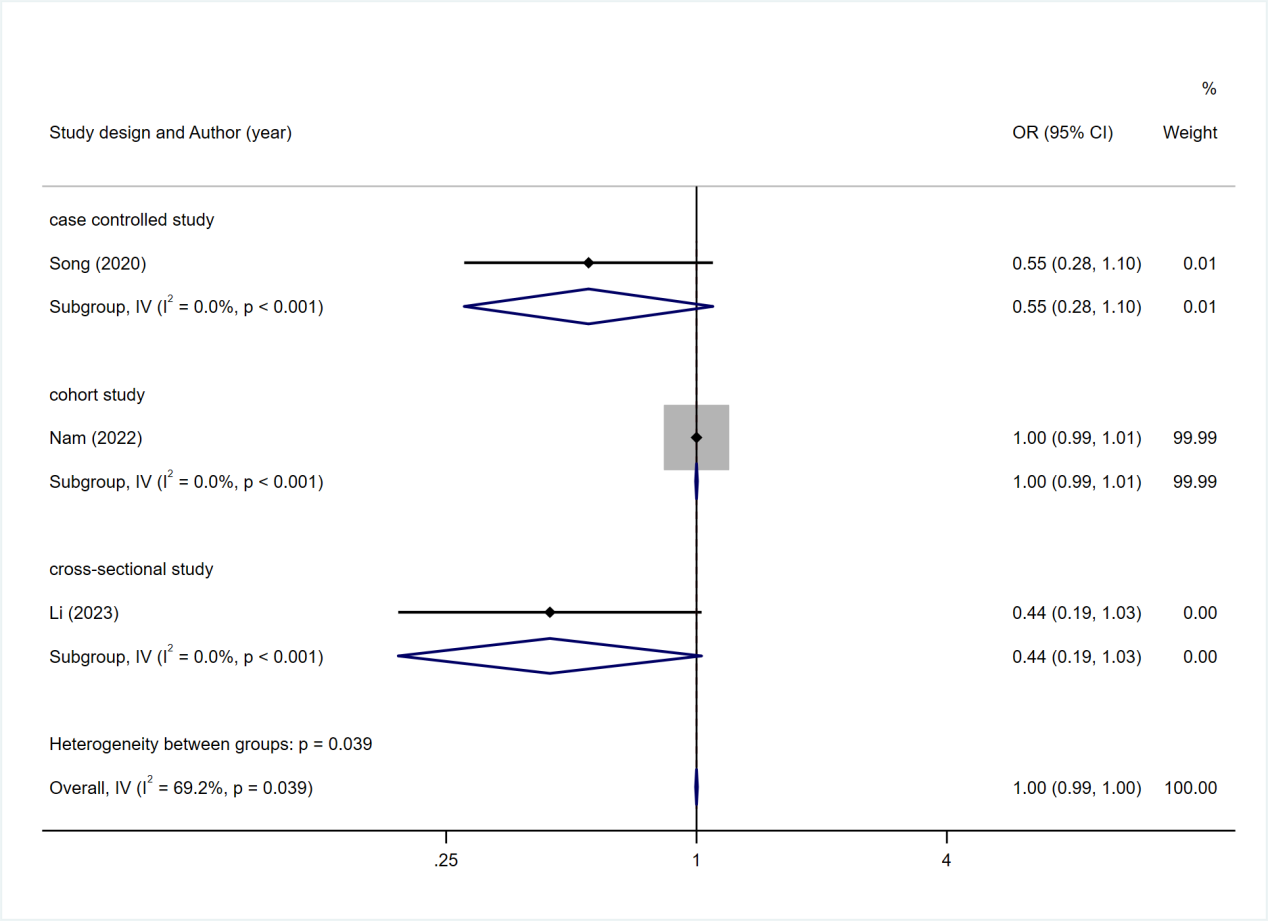
*

Figure S 141Forest Plot of the Subgroup Analysis of the Association Between TC and Sarcopenia in Patients with CKD Based on Study design

Study design is the source of heterogeneity.

*TG(Triglycerides)*

Figure S 142Sensitivity Analysis of the Association Between TG and Sarcopenia in Patients with CKD

Figure S 143Funnel Plot of the Association Between TG and Sarcopenia in Patients with CKD

Figure S 144Egger's Test of the Association Between TG and Sarcopenia in Patients with CKD

*TNF-a(Tumor Necrosis Factor-alpha)*

**

Figure S 145Sensitivity Analysis of the Association Between TNF-α and Sarcopenia in Patients with CKD

*
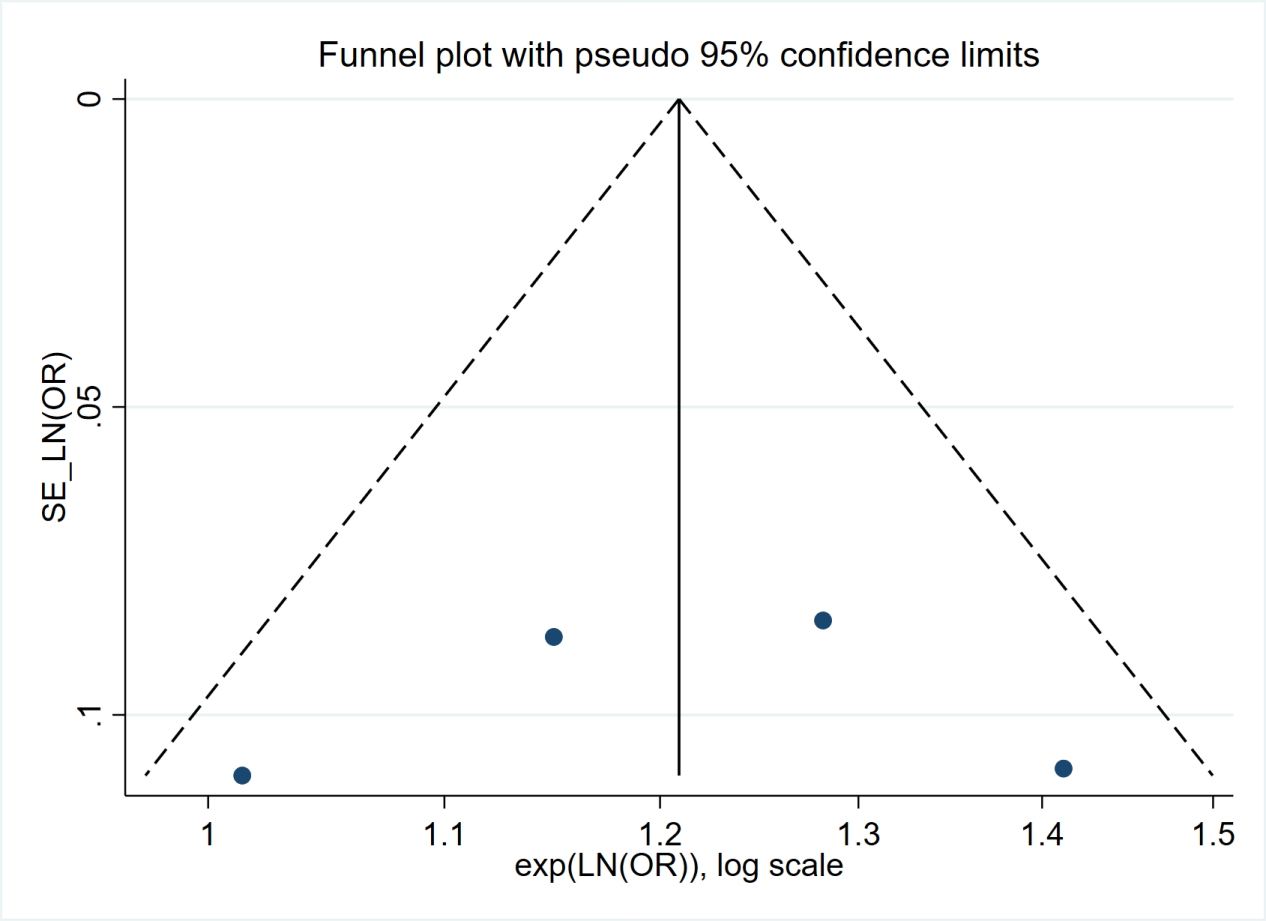
*

Figure S 146Funnel Plot of the Association Between TNF-α and Sarcopenia in Patients with CKD

*Figure 114 Funnel Plot of the Association Between TNF-α and Sarcopenia in Patients with CKD*

*
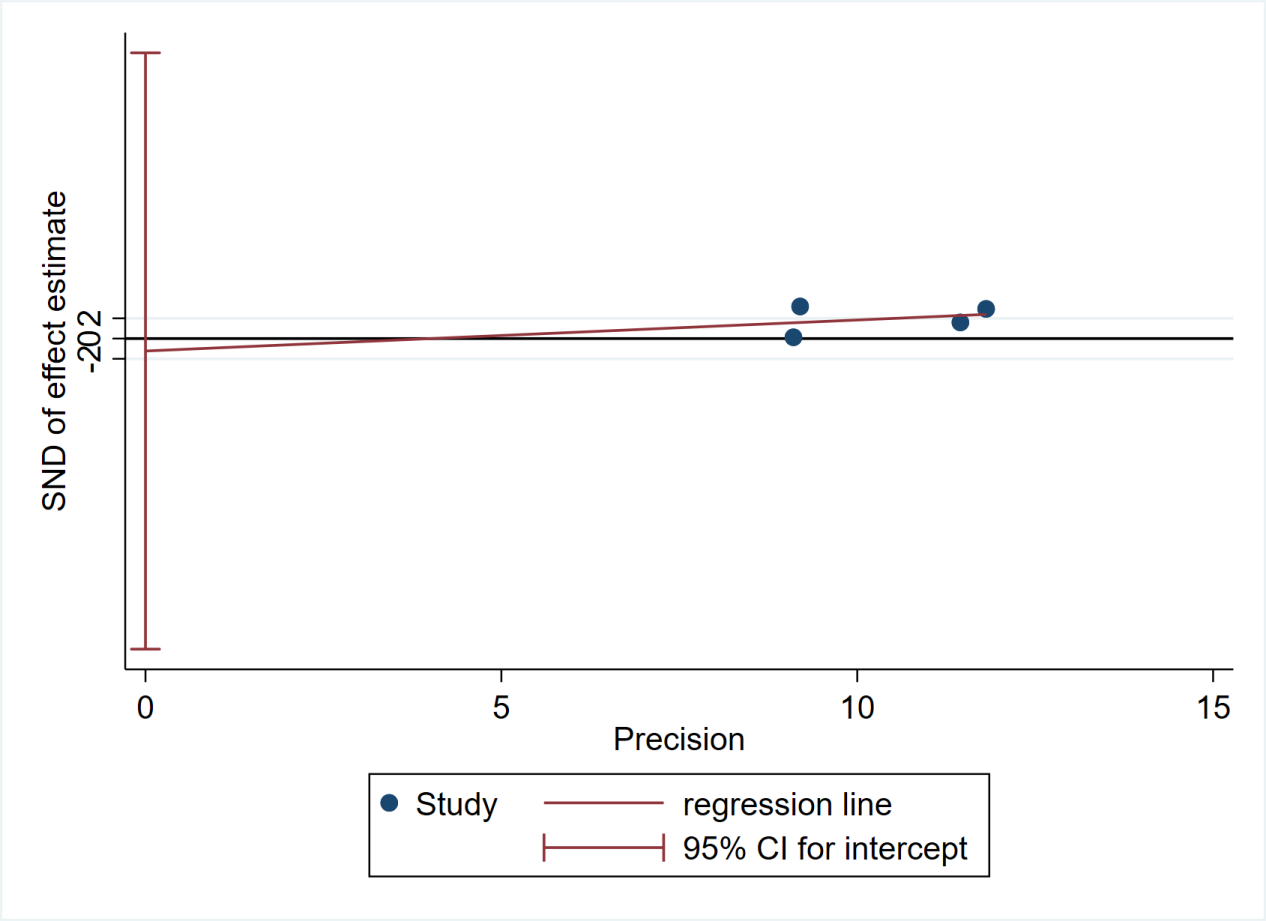
*

Figure S 147Egger's Test of the Association Between TNF-α and Sarcopenia in Patients with CKD

*Body water*

Figure S 148Sensitivity Analysis of the Association Between Body Water and Sarcopenia in Patients with CKD

Figure S 149Funnel Plot of the Association Between Body Water and Sarcopenia in Patients with CKD

Figure S 150Egger's Test of the Association Between Body Water and Sarcopenia in Patients with CKD

Figure S 151Forest Plot of the Subgroup Analysis of the Association Between Body Water and Sarcopenia in Patients with CKD Based on the Definition of sarcopenia

*Subgroup analysis revealed that the source of heterogeneity was primarily related to the definition of sarcopenia. Studies utilizing different criteria for diagnosing sarcopenia exhibited varying effect sizes, suggesting that inconsistent definitions may be a key factor contributing to the observed heterogeneity. This finding highlights the importance of using standardized criteria for sarcopenia diagnosis in future research to ensure comparability across studies.*

*urea nitrogen*

Figure S 152Sensitivity Analysis of the Association Between Urea Nitrogen and Sarcopenia in Patients with CKD

Figure S 153Funnel Plot of the Association Between Urea Nitrogen and Sarcopenia in Patients with CKD

Figure S 154Egger's Test of the Association Between Urea Nitrogen and Sarcopenia in Patients with CKD

*Uric Acid*

Figure S 155Sensitivity Analysis of the Association Between Uric Acid and Sarcopenia in Patients with CKD

Figure S 156Funnel Plot of the Association Between Uric Acid and Sarcopenia in Patients with CKD3

Figure S 157Egger's Test of the Association Between Uric Acid and Sarcopenia in Patients with CKD

IL-6(Interleukin6)

Figure S 158Sensitivity Analysis of the Association Between IL-6 and Sarcopenia in Patients with CKD

Figure S 159Funnel Plot of the Association Between IL-6 and Sarcopenia in Patients with CKD

Figure S 160Egger's Test of the Association Between IL-6 and Sarcopenia in Patients with CKD
